# Pyrroloquinoline Quinone Mitigates Type 2 Diabetes-Induced Cardiac Injury Through Mitochondrial Quality Control and Inhibition of NLRP3-Dependent Pyroptosis

**DOI:** 10.3390/metabo16050340

**Published:** 2026-05-19

**Authors:** Xue Zhang, Wei Liu, Zhijing Fu, Zhuoling Chen, Qixin Chen, Yanan Shen, Yukai Jin, Dengfeng Xu, Yin Wang, Xuefeng Qu, Yangjunna Zhang

**Affiliations:** School of Pharmacy, School of Food Science and Engineering, Hangzhou Medical College, Hangzhou 310013, China; 18137653819@163.com (X.Z.); 15762762835@163.com (W.L.); fuzhijinggq0501@hmc.edu.cn (Z.F.); czl5295@163.com (Z.C.); qixinchen163@163.com (Q.C.); shenyanan@hmc.edu.cn (Y.S.); jyk@hmc.edu.cn (Y.J.); withxu@hmc.edu.cn (D.X.); wy3333@hmc.edu.cn (Y.W.)

**Keywords:** diabetic cardiomyopathy, mitochondrial quality control, NLRP3 inflammasome, pyrroloquinoline quinone, type 2 diabetes

## Abstract

**Background**: Pyrroloquinoline quinone (PQQ), a naturally occurring redox cofactor with potent antioxidant and anti-inflammatory properties, has been shown to protect against cardiac injury. However, its therapeutic potential in diabetic cardiomyopathy (DCM) induced by Type 2 diabetes mellitus (T2DM) and the underlying mechanisms remain poorly understood. **Methods**: A T2DM mouse model was established via a high-fat diet and low-dose STZ. We investigated the cardioprotective effects of 12-week oral PQQ administration, assessing fasting blood glucose, oral glucose tolerance, cardiac function, myocardial histopathology, blood biochemistry, mitophagy, and NLRP3 inflammasome activation. In vitro experiments using AC16 cardiomyocytes exposed to palmitic acid and high glucose were also conducted. **Results**: Results showed PQQ significantly improved cardiac function, attenuated remodeling, and reduced proinflammatory cytokines in mice with T2DM, regulated key mitophagy-related proteins (Parkin, Beclin-1, LC3B-II, p62), and downregulated NLRP3 inflammasome pathway components (Caspase-1, NLRP3, IL-1β, IL-18). In vitro experiments demonstrated that PQQ reduced reactive oxygen species (ROS) production, improved mitochondrial membrane potential, promoted mitophagy, and inhibited NLRP3 inflammasome-mediated pyroptosis. **Conclusions**: PQQ alleviates DCM in mice with T2DM by improving mitochondrial quality control, promoting mitophagy, and subsequently inhibiting NLRP3 inflammasome-mediated pyroptosis, highlighting its potential as a promising therapeutic agent for T2DM-associated cardiomyopathy.

## 1. Introduction

According to data from the International Diabetes Federation, 537 million people worldwide were living with diabetes in 2021. One in ten adults has diabetes, and this number is projected to rise to 784 million by 2045. Type 2 diabetes mellitus (T2DM) is the most common form, accounting for approximately 90% of all diabetes cases globally [1]. T2DM-induced cardiomyopathy is a common cardiovascular complication that can ultimately lead to cardiac remodeling and is a major cause of death among patients with diabetes [2]. Myocardial fibrosis, cardiac hypertrophy, and microvascular damage are the main pathological characteristics of diabetic cardiomyopathy (DCM) [3]. These pathological changes can ultimately lead to cardiac dysfunction. An increasing number of studies have shown that mitochondrial quality control and inflammatory response play an important role in the pathophysiology of DCM [4]. Therefore, intervening in mitochondrial homeostasis and inflammatory signaling is an effective strategy to ameliorate the progression of DCM.

Pyrroloquinoline quinone (PQQ), a bioactive compound, plays a crucial role in maintaining human health, modulating physiological functions, and preventing various diseases [5,6,7]. Recent studies have shown that PQQ can enhance mitochondrial function, attenuate oxidative stress, and suppress neuroinflammation in the central nervous system, suggesting its therapeutic value for neurodegenerative disorders such as Alzheimer’s and Parkinson’s diseases [8,9]. It has also been recognized as the third redox cofactor discovered, following nicotinamide nucleotides and flavin nucleotides [10,11]. It is primarily synthesized by bacteria and is widely distributed in vegetables, fruits, and meats [12,13]. In humans, PQQ is obtained mainly from dietary sources, as free PQQ has been detected in vital organs including the heart, brain, liver, and lungs, as well as in plasma and breast milk [14,15]. However, humans and other mammals are unable to synthesize PQQ, and the intestinal microbiota cannot generate sufficient amounts to meet physiological requirements. Therefore, exogenous supplementation of PQQ is considered important for maintaining health and preventing disease progression. Regulatory authorities have also begun to acknowledge its potential nutritional value: PQQ has been designated as a new member of the vitamin B family in Japan [16], and it has been approved as a new resource food in both China and the European Union. Furthermore, dietary supplements containing PQQ are already available in the United States [6]. A comprehensive investigation into the protective effects of PQQ against type 2 diabetic cardiomyopathy may not only provide novel insights for the prevention and treatment of this condition but also serve as a theoretical foundation for the development of new functional foods and pharmaceutical agents.

Previous investigations have demonstrated that PQQ can potentially ameliorate impaired glucose tolerance [17], mitigate myocardial cell hypertrophy [18], and prevent cardiovascular diseases [19]. PQQ has been shown to enhance mitochondrial function, with its ortho-quinone structure serving as a key functional group. Acting as an electron acceptor and donor, this structure participates in electron transfer processes involving riboflavin, nicotinamide nucleotides, and cytochrome C within the mitochondrial respiratory complexes, thereby contributing to the maintenance of mitochondrial quality control [20]. Early PQQ supplementation has persistent long-term protective effects on developmental programming of hepatic lipotoxicity and inflammation in mice with obesity [21]. In this study, PQQ was administered to maternal mice during gestation and lactation, with offspring subsequently weaned onto a Western-style diet. Despite PQQ withdrawal at weaning, offspring exhibited sustained protection from hepatic steatosis, fibrosis, and inflammation when examined at 20 weeks of age, indicating that early PQQ exposure confers enduring metabolic benefits. However, whether PQQ exerts a protective effect on type 2 diabetic cardiomyopathy and the underlying molecular regulatory mechanisms thereof remains to be elucidated.

Mitochondrial quality control serves as a crucial strategy in the prevention and treatment of heart diseases [22]. In the context of T2DM, the mitochondrial permeability transition pore (mPTP) exhibits excessive opening, leading to mitochondrial swelling, structural alterations in mitochondrial cristae, and dysfunction of the electron transport chain, which collectively contribute to excessive reactive oxygen species (ROS) production [23,24]. Furthermore, T2DM suppresses cardiac mitophagy, impairing the timely removal of damaged mitochondria and consequently leading to elevated ROS levels. The excessive accumulation of ROS then triggers the dissociation of thioredoxin (TRX) from its binding partner, thioredoxin-interacting protein (TXNIP) [25,26]. Once released, TXNIP translocates from the mitochondria to the cytoplasm, where it interacts with NLRP3 and facilitates the activation of the NLRP3 inflammasome. Persistent activation of the nucleotide-binding oligomerization domain-like pyrin domain-containing protein 3 (NLRP3) inflammasome contributes significantly to the progression of DCM [27]. Despite evidence of PQQ’s cardioprotective effects, whether PQQ protects against type 2 diabetic cardiomyopathy remains unknown, and the role of PQQ in regulating mitophagy has not been explored. Therefore, the present study aimed to investigate whether PQQ protects against type 2 diabetic cardiomyopathy by enhancing mitophagy and suppressing NLRP3 inflammasome activation.

## 2. Materials and Methods

### 2.1. Materials and Animals

PQQ (purity > 98%) was provided by Zhucheng HaoTian Pharm Co., Ltd. (Zhucheng, China). To avoid the potential confounding effects of the estrous cycle on metabolic and inflammatory parameters, which could introduce additional variability in this initial mechanistic study, only male mice were used. Eight-week-old male C57BL/6 mice were purchased from the Animal Experiment Center of Hangzhou Medical College (Hangzhou, China). All mice were provided free access to water under controlled environmental conditions of 25 ± 2 °C temperature and 55 ± 5% relative humidity, with a 12-h light–dark cycle. All animal experimental procedures were performed in accordance with the Guidelines for Ethical Review of Animal Welfare in Experimental Animals [28] (GB/T 35892-2018, revised 2018), issued by the China Experimental Animal Standardization Technical Committee. The experimental protocols were approved by the Ethics Committee of Hangzhou Medical College (Approval No. 2021-004).

### 2.2. Mice Modeling and Grouping

After a 5-day acclimatization period, the mice were randomly divided into two groups: the high-fat diet (HFD) combined with low-dose streptozotocin (STZ)-induced type 2 diabetic group (*n* = 50), and the control group (*n* = 10). To induce type 2 diabetes [29], fifty male C57BL/6 mice were fed a high-fat diet (HFD) for three weeks and subsequently received intraperitoneal injections of a low dose of streptozotocin (STZ; Sigma, St. Louis, MO, USA) at 35 mg/kg body weight (BW). The injections were given twice, with a one-week interval, following the three-week HFD feeding period. The HFD (XTHF60; Jiangsu Xietong Pharmaceutical and Biological Engineering Co., Ltd., Nanjing, China) contained 23% protein, 27% carbohydrate, and 35% fat by mass (mass ratio, the same below). Meanwhile, another group of 10 mice served as the control group (Ctl group) and were maintained on a control diet for the same duration. These mice received intraperitoneal injections of 0.05 mol/L citrate buffer. The nutrient composition of the control diet (1010009, Xietong, China) consisted of 19% protein, 61% carbohydrates, and 5% fat. Fasting blood glucose levels were measured using a glucometer (LifeScan, Milpitas, CA, USA) on the 7th day following the final injection. Forty mice with blood glucose levels ≥ 11.1 mmol/L were considered to have successfully developed diabetes and used for subsequent experiments. The remaining 10 mice that did not meet the glycemic criteria were excluded from further analysis.

The PQQ was dissolved in deionized water. Mice with diabetes were randomly divided into four groups, with 10 mice in each group: a type 2 diabetic cardiomyopathy (DCM) group, a type 2 diabetic group treated with low-dose PQQ (DCM + PQQL), a type 2 diabetic group treated with medium-dose PQQ (DCM + PQQM), and a type 2 diabetic group treated with high-dose PQQ (DCM + PQQH). PQQ was administered to the mice via oral gavage at doses of 10, 20, and 40 mg/kg BW/day for the low-, medium-, and high-dose groups, respectively. The DCM group received the same gavage procedure with an equal volume of deionized water. The doses of PQQ were selected based on previous studies demonstrating the efficacy and safety of PQQ in rodent models [12]. All mice were administered gavage continuously once daily for 12 weeks. Body weight was measured on a weekly basis, and fasting blood glucose levels were monitored every two weeks. At the end of the 12-week period, the mice were anesthetized, and blood and heart tissues were collected for subsequent experimental analyses. For all in vivo experiments, each individual mouse was considered an independent biological replicate.

### 2.3. Fasting Blood Glucose Measurement

At the end of week 12, the mice were fasted starting at 8 a.m. for a duration of 4 h [30]. The tail tips were disinfected with 75% alcohol, and a disposable lancet was used to puncture the tail tip, with the first drop of blood being discarded. Subsequently, blood glucose levels were measured using a calibrated glucometer (LifeScan, Milpitas, CA, USA), and the readings were recorded.

### 2.4. Glucose Tolerance Test

Prior to the test, mice were fasted for 16 h with ad libitum access to water. Following the fasting period, the animals received an oral gavage of glucose solution (Sigma-Aldrich, USA) at a dosage of 2 g/kg body weight. Blood glucose levels were measured at 0, 15, 30, 60, and 120 min after glucose administration. These values were used to construct a glucose tolerance curve. The area under the curve (AUC) was calculated based on the following equation:

AUC = (0.5 × [G0 + G30] + 1.5 × [G30 + G120])/2,

where Gt denotes the blood glucose concentration at time point t (minutes).

### 2.5. Insulin Tolerance Test

For the insulin tolerance test, mice were subjected to a 4-h fast with water provided ad libitum. Following the fasting period, the animals were administered insulin (1 U/kg BW, human, MedChemExpress, Monmouth Junction, NJ, USA) via intraperitoneal injection. Blood glucose levels were measured from tail vein blood samples using a glucometer at baseline (0 min) and at 15, 30, 60, and 120 min after insulin administration. The area under the glucose curve (AUC) was calculated to assess insulin sensitivity according to the following formula:

AUC = (0.5 × [G0 + G30] + 1.5 × [G30 + G120])/2,

where Gt indicates the blood glucose level at time t (in minutes).

### 2.6. Echocardiography

One day prior to echocardiography, chest hair from the mice was removed using a depilatory cream to facilitate acoustic coupling. General anesthesia was induced by placing the animals in an anesthetic induction chamber supplied with 3% isoflurane in oxygen at a flow rate of 500 mL/min. Two-dimensional echocardiograms of mice were obtained using a small-animal imaging system (Vinno D6 Vet, VINNO Technology, Suzhou, China), and the position of the M-shaped sampling line was adjusted to obtain the corresponding M-mode curve, which was measured to obtain the left ventricular (LV) long-axis view. From these recordings, the following parameters were measured: LV end-diastolic and end-systolic internal dimensions (LVIDd, LVIDs), LV end-diastolic and end-systolic interventricular septum thickness (IVSd, IVSs), end-diastolic and end-systolic LV posterior wall thickness (LVPWd, LVPWs), ejection fraction (EF), and fractional shortening (FS).

### 2.7. Biochemical Analyses

At the end of the 12th week, mice were anesthetized via intraperitoneal injection of 2% 2,2,2-tribromoethanol (T48402; Aladdin, Shanghai, China). Blood samples were then collected from the retro-orbital plexus and centrifuged at 3000 rpm for 15 min at 4 °C to obtain serum. Serum biochemical parameters were measured using an automated biochemical analyzer (Lanyun C400, Shenzhen Landwind Industry Co., Ltd., Shenzhen, China). The measured indicators included albumin (ALB, g/L), total protein (TP, g/L), alkaline phosphatase (ALP, U/L), alanine aminotransferase (ALT, U/L), aspartate aminotransferase (AST, U/L), blood urea nitrogen (BUN, mmol/L), creatinine (CR, μmol/L), gamma-glutamyl transferase (GGT, U/L), blood glucose (GLU, mmol/L), high-density lipoprotein cholesterol (HDL-C), low-density lipoprotein cholesterol (LDL-C). The commercial assay kits were obtained from Mindray (Shenzhen, China).

### 2.8. Masson’s Trichrome Staining

Mouse heart tissues were fixed in 4% paraformaldehyde (Biosharp, Beijing Lanjieke Technology Co., Ltd., Beijing, China) for 24 h at room temperature. After fixation, the tissues were dehydrated through a graded ethanol series, cleared in xylene, and embedded in paraffin. Sections of 4 μm thickness were prepared using a rotary microtome and mounted onto glass slides. The sections were then deparaffinized and rehydrated before being stained with a Masson’s trichrome staining kit (Solarbio, Beijing, China), following the manufacturer’s instructions. Stained sections were examined under a light microscope (Olympus Corporation, Tokyo, Japan) at 200× magnification. Myocardial interstitial fibrosis was assessed by evaluating the blue-stained collagen deposits surrounding cardiomyocytes. Four randomly selected non-overlapping fields were quantified per section. Image acquisition and analysis were performed in a blinded manner.

### 2.9. Cell Culture and Treatment

AC16 human cardiomyocytes were purchased from Otwo Biotech (HTX2571, Shenzhen, China) and cultured in Dulbecco’s modified Eagle’s medium (DMEM; Gibco, Grand Island, NY, USA) containing 10% fetal bovine serum (Biological Industries, Haemek, Israel) and 1% penicillin–streptomycin (TBD, Tianjin, China) at 37 °C in an incubator under a 5% CO_2_ atmosphere. Cells were exposed to high glucose (HG), palmitic acid (PA, KC002, Psaitong, Tianjin, China), and pyrroloquinoline quinone (PQQ, D7783, Sigma) for 24 h, with nigericin (HY-127019, MedChemExpress, Shanghai, China) administered during the final two hours of the treatment period [31]. The cells were divided into four groups: control group (5.5 mmol/L glucose), HG + PA group (35 mmol/L glucose, 0.25 mmol/L PA), HG + PA + PQQ group (10 nmol/L PQQ), and HG + PA + PQQ + nigericin group (10 μg/mL nigericin). For all in vitro experiments, each independent cell culture passage or experiment was considered a biological replicate. Technical replicates were used only to ensure measurement consistency and were not included as independent data points for statistical analysis.

### 2.10. Detection of Intracellular ROS

AC16 human cardiomyocytes were seeded in six-well culture plates at a density of 1.0 × 10^6^ cells per well and allowed to adhere for 24 h under standard culture conditions (37 °C, 5% CO_2_). Cells were treated with high glucose (HG), palmitic acid (PA), and pyrroloquinoline quinone (PQQ) for 24 h. To evaluate intracellular reactive oxygen species (ROS) levels, the cells were incubated with 20 μmol/L 2′,7-dichlorofluorescin diacetate (DCFH-DA; 50101ES01, Yeasen, Shanghai, China) in serum-free medium for 45 min at 37 °C in the dark. After incubation, the cells were gently washed twice with phosphate-buffered saline (PBS) to remove excess probe, with each wash lasting for 5 min. Fluorescence images were captured immediately using a fluorescence microscope (Olympus Corporation, Tokyo, Japan) under standardized exposure conditions across all experimental groups at a magnification of 200×. ROS levels were qualitatively assessed by DCF fluorescence intensity. Four randomly selected non-overlapping fields were captured per coverslip, and three independent experiments were performed. Image acquisition and analysis were performed in a blinded manner. Uncropped original microscopy images for all immunofluorescence experiments are provided in Figure S2.

### 2.11. Mitochondrial Membrane Potential Analysis

The mitochondrial membrane potential in AC16 cells under HG, PA, and PQQ treatments was assessed using a JC-1 mitochondrial membrane potential assay kit (HY-K0601, MedChemExpress, Shanghai, China). AC16 cells were seeded in six-well culture plates at a density of 1.0 × 10^6^ cells per well and cultured for 24 h. Following this, the cells were incubated with 2 μmol/L JC-1 for 20 min in the dark at 37 °C. Subsequently, the cells were washed twice with PBS and examined under a fluorescence microscope (Olympus Corporation, Tokyo, Japan) at a magnification of ×200.

### 2.12. Pyroptosis Staining

To assess cell pyroptosis, the cells were incubated in serum-free medium containing a staining solution composed of YO-PRO-1 iodide (6 μmol/L; Y3603, Thermo Fisher Scientific, Waltham, MA, USA) and ethidium homodimer-2 (EthD-2) (2 μmol/L; E3599, Thermo Fisher Scientific, USA) for 30 min at 37 °C under dark conditions. YO-PRO-1 (green, a small molecule) is a membrane-impermeable yet pyroptosis-permeable dye that selectively labels cells with damaged membranes, serving as an early indicator of pyroptosis. In contrast, EthD-2 (red, a larger molecule) is impermeable to both intact and pyroptosis-compromised membranes and only enters cells at later stages of severe membrane disruption. Cells were then stained with Hoechst 33258 (5 μg/mL; Yeasen, Shanghai, China) for 10 min and images were captured using fluorescence microscopy (Olympus Corporation, Tokyo, Japan) at a magnification of ×200. The degree of pyroptosis was qualitatively assessed by the percentage of positive cells. Four randomly selected non-overlapping fields were analyzed per coverslip, and three independent replicates were performed. Image acquisition and analysis were performed in a blinded manner.

### 2.13. Western Blotting Analysis

Total proteins were extracted from cardiac left ventricular tissues using RIPA lysis buffer (AR0102, Boster Biological Technology, Wuhan, China) containing 1% protease (Boster, Wuhan, China) and phosphatase inhibitors (Boster, Wuhan, China). The concentration of the extracted proteins was determined using a BCA kit (Tiangen, Beijing, China). Equal amounts of protein were separated by 8–15% SDS-PAGE and transferred to nitrocellulose (NC) membranes (BioTrace, Courbevoie, France). After blocking the NC membrane with 5% skim milk for 2 h at room temperature, the membranes were incubated with primary antibody overnight at 4 °C. The following primary antibodies were used: Atrial natriuretic peptide (ANP, ab262703, Abcam, UK), Brain natriuretic peptide (BNP, ab236101, Abcam, UK), Collagen I (14695-1-AP, Proteintech, Wuhan, China), Collagen III (abs131560, Abcam, Cambridge, UK), Parkin (Prk8, Cell Signaling Technology, Danvers, MA, USA), Beclin-1 (3738S, Cell Signaling Technology, Danvers, MA, USA), LC3BI/II (E7X4S, Cell Signaling Technology, Danvers, MA, USA), p62 (5114S, Cell Signaling Technology, Danvers, MA, USA), NLRP3 (BA3677, Boster Biological Technology, Wuhan, China), Caspase-1 (E2Z1C, Cell Signaling Technology, Danvers, MA, USA), IL-1β (3A6, Cell Signaling Technology, Danvers, MA, USA), IL-18 (E8P50, Cell Signaling Technology, Danvers, MA, USA), and GAPDH (BK7021, Bioker, Hangzhou, China). After incubation, the membranes were washed three times with phosphate-buffered saline (PBS)-Tween 20, and then were incubated with fluorescently labeled secondary antibody (LI-COR Biosciences, Lincoln, NE, USA) for 1 h at room temperature in the dark. The Western blotting data were normalized with GAPDH. Results were captured and analyzed using the Odyssey Imaging System (LI-COR Biosciences). For Western blot analysis, each biological replicate originated from the heart tissue of a different mouse. Full uncropped original Western blot images are provided in Figure S1.

### 2.14. Immunofluorescence

AC16 cells were cultured in a six-well plate at a density of 1.0 × 10^6^ cells per well. After being washed three times with PBS, the cells were fixed with 0.5 mL of 4% paraformaldehyde fixing solution for 15 min. Then, a permeabilization treatment was carried out with 0.5% Triton X-100. Subsequently, the cells were blocked with 5% BSA at room temperature for 1 h and washed with PBS. After that, the cells were incubated with the primary antibody at 4 °C overnight. The following primary antibodies were used: Parkin (HA722952, Huaan, Hangzhou, China), Beclin-1 (HA721216, Huaan, Hangzhou, China), PINK1 (23274-1-AP, Proteintech, Wuhan, China), p62 (HA721171, Huaan, Hangzhou, China), NLRP3 (ET1610-93, Huaan, Hangzhou, China), Caspase-1 cut (HA722222, Huaan, Hangzhou, China), IL-1β (ET1701-39, Huaan, Hangzhou, China), and ASC (ab309497, Abcam, UK). The following day, the cells were incubated with Alexa Fluor^®^ 594 fluorescent secondary antibody (ab150080, Abcam, UK) at room temperature for 2 h. Subsequently, Hoechst 33258 (40730ES03, Yeasen, China) was added after dilution with PBS to a final concentration of 5 μg/mL for a duration of 5 min. Fluorescence images were captured using a fluorescence microscope (Olympus Corporation, Tokyo, Japan) at 200× magnification. Changes in protein expression were qualitatively assessed by fluorescence intensity. Four randomly selected non-overlapping fields were captured per coverslip using a fluorescence microscope, and three independent experiments were performed. Image acquisition and analysis were performed in a blinded manner.

### 2.15. Statistical Analysis

The variables were initially assessed for normal distribution using the Shapiro–Wilk test and homogeneity of variances using Levene’s test in SPSS 22.0 software (Version 22.0; IBM, Armonk, NY, USA). Subsequently, if the variables exhibited normal distribution and comparable variances, they underwent analysis of variance (one-way ANOVA), followed by Tukey’s post hoc test for multiple comparisons. In cases where the variables did not demonstrate normal distribution or had unequal variances, Tamhane’s test was employed. The statistical data generated from the presented results were expressed as the mean ± standard deviation (SD) [32]. *p* < 0.05 was considered statistically significant.

## 3. Results

### 3.1. PQQ Effectively Alleviates Abnormalities in Organ Weight, Body Weight, and Blood Glucose Levels in Type 2 Diabetic Mice

**Figure 1:**
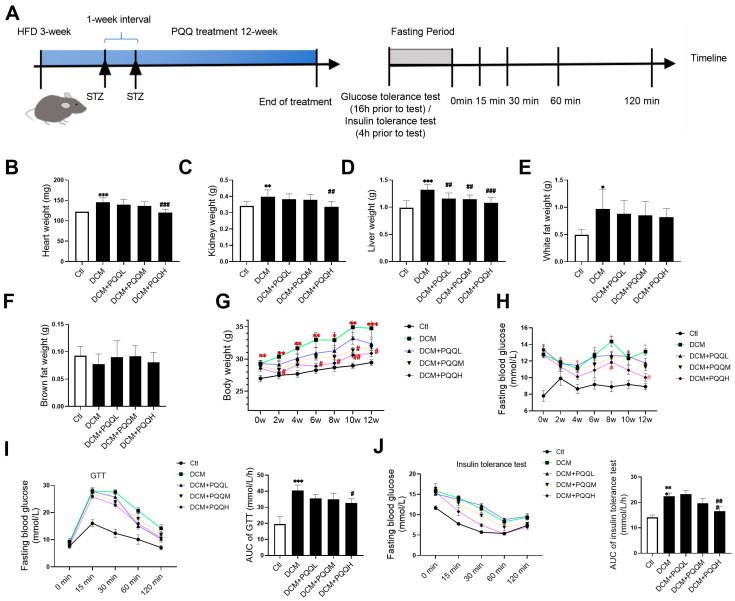
PQQ effectively alleviates abnormalities in organ weight, body weight, and blood glucose levels in mice with type 2 diabetes. (**A**) Schematic timeline of the experimental protocol. The (**B**) heart weight, (**C**) kidney weight, (**D**) liver weight, (**E**) white fat weight, (**F**) brown fat weight, and (**G**) body weight of mice after 12 weeks of PQQ treatment. (**H**) The time variation in fasting blood glucose concentration with 12 weeks of PQQ treatment. (**I**) Glucose tolerance and the area under the glucose tolerance curve following PQQ treatment; (**J**) Insulin resistance and the area under the insulin resistance curve following PQQ treatment. Data were expressed as mean ± SD (*n* = 10 mice per group, * *p* < 0.05, ** *p* < 0.01, *** *p* < 0.001 vs. Ctl; ^#^ *p* < 0.05, ^##^ *p* < 0.01, ^###^ *p* < 0.001 vs. DCM).

Before PQQ treatment (week 0), no significant differences in body weight or fasting blood glucose levels were observed among the four groups of mice with diabetes, confirming successful randomization. Following 12 weeks of continuous intragastric administration of varying doses of PQQ to mice with type 2 diabetes (Figure 1A–E), the heart, kidney, and liver weights in the DCM group were significantly higher than those in the Ctl group (*p* < 0.01 or *p* < 0.001). However, PQQ intervention, particularly in the DCM + PQQH group, partially attenuated this organ weight gain. With respect to adipose tissue, the DCM group exhibited a significant increase in white fat content (*p* < 0.05). Nevertheless, the effects of PQQ treatment on adipose tissue were not statistically significant. Body weight monitoring results (Figure 1F) showed that the DCM group had significantly higher body weight gain compared to the control group. With increasing PQQ dosage, the trend in body weight gain gradually normalized and approached that of the Ctl group. Notably, the DCM + PQQH group exhibited significantly lower body weight from the 6th to the 12th week (*p* < 0.05, *p* < 0.01). During the 12-week treatment period, no mortality or obvious behavioral abnormalities were observed in any of the PQQ-treated groups. All mice appeared healthy and active.

Fasting blood glucose monitoring (Figure 1G) demonstrated that blood glucose levels remained stable in the Ctl group, whereas they were significantly elevated in the DCM group (*p* < 0.05). Following PQQ treatment, blood glucose levels decreased, with the high-dose group showing significant reductions at the 8th and 12th weeks (*p* < 0.05). In the glucose tolerance test (GTT) and insulin tolerance test (ITT) (Figure 1H,I), the DCM group exhibited impaired glucose tolerance and reduced insulin sensitivity, as evidenced by significantly higher area under the curve (AUC) values compared to the Ctl group (*p* < 0.001). Following PQQ administration, both glucose clearance and insulin response were improved, with the DCM + PQQH group showing significantly lower AUC values than the DCM group (*p* < 0.05 or *p* < 0.001). Collectively, these findings indicate that PQQ can partially mitigate organ hypertrophy, ameliorate body weight and glycemic abnormalities, and enhance glucose tolerance and insulin sensitivity, thereby contributing to the restoration of metabolic homeostasis in mice with type 2 diabetes.

### 3.2. PQQ Ameliorates Cardiac Functional Impairments in Type 2 Diabetic Mice

**Figure 2:**
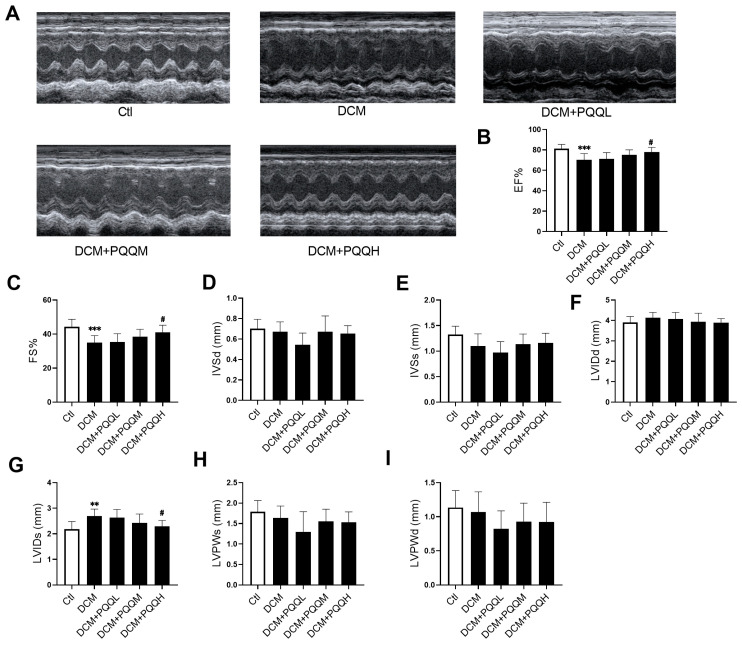
PQQ ameliorates cardiac functional impairments in mice with type 2 diabetes. (**A**) Representative images of mice cardiac echocardiography; (**B**) Ejection fraction (EF), (**C**) Fractional shortening (FS), (**D**) left ventricular end-diastolic septal thickness (IVSd), (**E**) left ventricular end-systolic septal thickness (IVSs), (**F**) left ventricular end-diastole internal dimension (LVIDd), (**G**) left ventricular end-systolic internal dimension (LVIDs), (**H**) left ventricular end-systolic posterior wall thickness (LVPWs), and (**I**) left ventricular end-diastole posterior wall thickness (LVPWd) were evaluated by echocardiography in different treatment groups. Data were expressed as mean ± SD (*n* = 10 mice per group, ** *p* < 0.01, *** *p* < 0.001 vs. Ctl; ^#^ *p* < 0.05 vs. DCM).

To assess the impact of PQQ on cardiac function in mice with type 2 diabetes, echocardiographic analysis was performed (Figure 2). The results showed that the DCM group exhibited significant structural and functional impairments, characterized by increased left ventricular end-systolic diameter (LVIDs, *p* < 0.01) and reduced ejection fraction (EF%) and fractional shortening (FS%, *p* < 0.001). Following PQQ administration, improvements in both cardiac structure and function were observed to varying extents, with the high-dose group (DCM + PQQH) showing the most pronounced beneficial effects. Collectively, these findings indicate that PQQ effectively mitigates cardiac structural abnormalities and dysfunction in mice with type 2 diabetes.

### 3.3. Effects of PQQ on Myocardial Pathological Alterations and Biomarkers of Cardiac Fibrosis and Hypertrophy in Mice with Type 2 Diabetes

**Figure 3:**
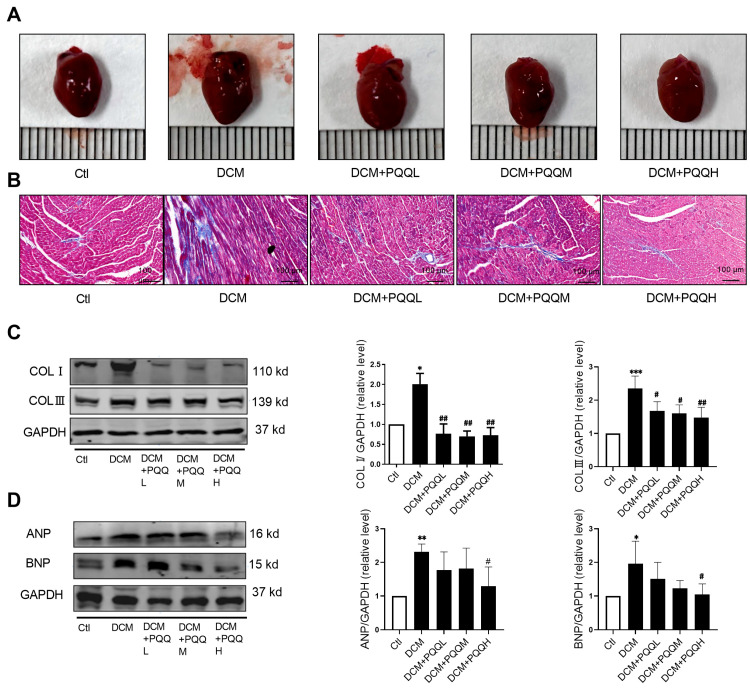
Effects of PQQ on myocardial pathological alterations and biomarkers of cardiac fibrosis and hypertrophy in mice with type 2 diabetes. (**A**) Cardiac morphological changes among different treatment groups. (**B**) Representative Masson’s trichrome staining of interstitial fibrosis in different groups; magnification 200×. (**C**) Type I collagen (COL-I) and type III collagen (COL-III) of mouse hearts were detected by Western blotting in different groups. (**D**) Atrial natriuretic peptide (ANP) and brain natriuretic peptide (BNP) of mouse hearts were detected by Western blotting in different groups. Data were expressed as mean ± SD (*n* = 4 per group, * *p* < 0.05, ** *p* < 0.01, *** *p* < 0.001 vs. Ctl; ^#^ *p* < 0.05, ^##^ *p* < 0.01 vs. DCM).

To further elucidate the protective effects of PQQ against T2DM-associated cardiac injury, the present study utilized gross morphological examination, Masson’s trichrome staining, and Western blot analysis to assess the myocardial and expression of key biomarker proteins (Figure 3). Gross morphological observation (Figure 3A) revealed that hearts from the DCM group exhibited increased size, whereas PQQ-treated groups—particularly the high-dose group (DCM + PQQH)—displayed reduced heart size. Histological analysis (Figure 3B) demonstrated that myocardial architecture in the Ctl group was well-preserved, with tightly packed and regularly arranged cardiomyocytes, and no evidence of collagen deposition, representing normal cardiac morphology. In contrast, the DCM group exhibited marked pathological alterations characterized by extensive interstitial fibrosis. Following PQQ administration, these pathological changes were significantly ameliorated.

Western blot analysis (Figure 3C,D) revealed that the expression levels of fibrosis-associated proteins Collagen I and Collagen III were significantly elevated in the DCM group (*p* < 0.05 or *p* < 0.001), but markedly reduced after PQQ treatment. Furthermore, the cardiac hypertrophy biomarkers ANP and BNP were significantly upregulated in the DCM group (*p* < 0.05, *p* < 0.01), but were notably reduced in the high-dose PQQ treatment group (*p* < 0.05). Collectively, these findings suggest that PQQ exerts cardioprotective effects in mice with T2DM by attenuating myocardial structural damage, suppressing interstitial fibrosis, and myocardial hypertrophy.

### 3.4. Effects of PQQ on Blood Biochemical Parameters in Type 2 Diabetic Mice

**Figure 4:**
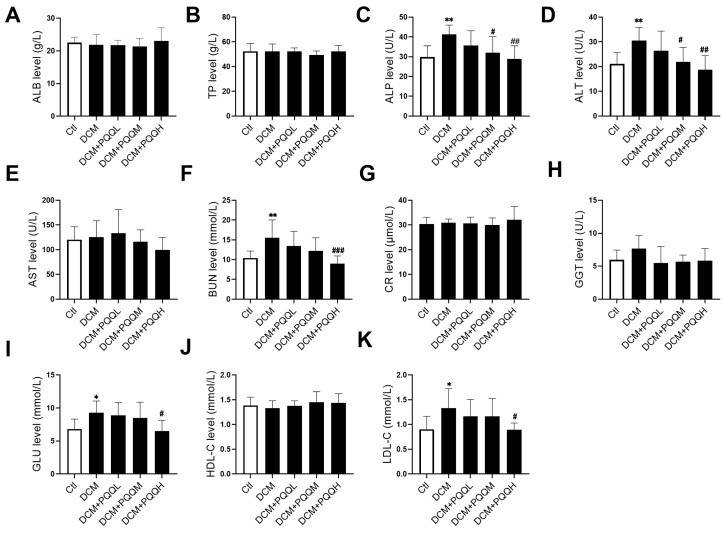
Effects of PQQ on blood biochemical parameters in mice with type 2 diabetes. The effects of PQQ intervention were clarified by determining the variations in the levels and contents of (**A**) albumin (ALB), (**B**) total protein (TP), (**C**) alkaline phosphatase (ALP), (**D**) alanine transaminase (ALT), (**E**) aspartate transaminase (AST), (**F**) blood urea nitrogen (BUN), (**G**) creatinine (CR), (**H**) gamma-glutamyl transferase (GGT), (**I**) glucose (GLU), (**J**) high-density lipoprotein cholesterol (HDL-C), and (**K**) low-density lipoprotein cholesterol (LDL-C) in different groups. Data were expressed as mean ± SD (*n* = 10 per group, * *p* < 0.05, ** *p* < 0.01 vs. Ctl; ^#^ *p* < 0.05, ^##^ *p* < 0.01, ^###^ *p* < 0.001 vs. DCM).

To evaluate the regulatory effects of PQQ on blood biochemical parameters in a T2DM mouse model, serum biochemical profiles were analyzed following 12 weeks of daily oral administration (Figure 4). The results showed that alkaline phosphatase (ALP) and alanine aminotransferase (ALT) levels in the DCM group were significantly elevated compared to the control group (*p* < 0.01), indicating potential hepatic injury. Following treatment with 20 mg/kg BW/day and 40 mg/kg BW/day of PQQ, both ALP and ALT levels were markedly reduced (*p* < 0.05 or *p* < 0.01), suggesting that PQQ exerts a protective effect on liver function. With respect to renal function, blood urea nitrogen (BUN) level was significantly increased in the DCM group compared to the control group (*p* < 0.01), indicating impaired kidney function. High-dose PQQ treatment significantly reduced BUN levels (*p* < 0.001), suggesting a potential renoprotective effect of PQQ. In terms of metabolic parameters, the DCM group exhibited significantly elevated blood glucose (GLU) level (*p* < 0.05), which was significantly lowered after 40 mg/kg BW/day PQQ administration (*p* < 0.05), confirming its beneficial role in glycemic control. Lipid profile analysis revealed that low-density lipoprotein cholesterol (LDL-C) level was increased in the DCM group compared to the control (*p* < 0.05). High-dose PQQ treatment significantly reduced LDL-C level (*p* < 0.05), indicating that PQQ may help alleviate lipid dysregulation in mice with T2DM.

### 3.5. The Dual Regulatory Effects of PQQ on Mitophagy and the NLRP3 Inflammasome Pathway in T2DM-Induced Diabetic Cardiomyopathy

**Figure 5:**
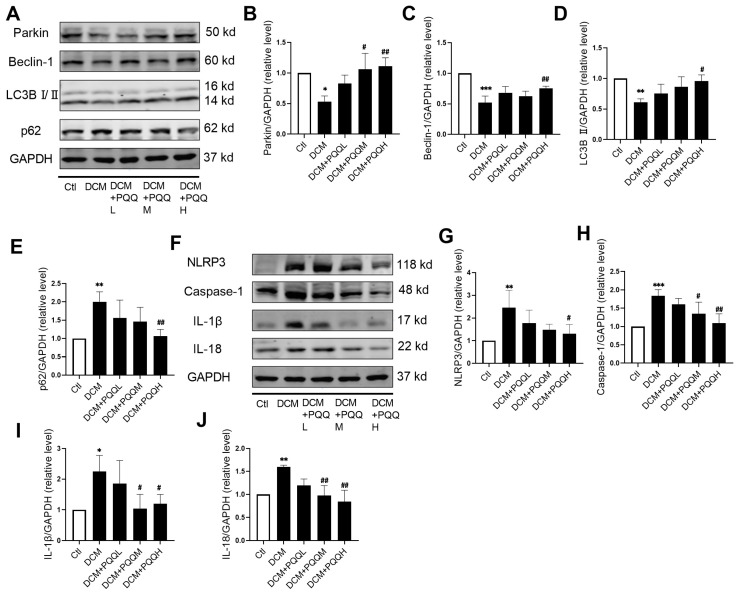
The dual regulatory effects of PQQ on mitophagy and the NLRP3 inflammasome pathway in T2DM-induced diabetic cardiomyopathy. (**A**) The protein expression levels of Parkin, Beclin-1, LC3B, and p62 in different groups were detected by Western blot analysis. Variations in the levels of (**B**) Parkin, (**C**) Beclin-1, (**D**) LC3B, and (**E**) p62 proteins indicate the ameliorative effect of PQQ intervention. (**F**) The protein expression levels of NLRP3, Caspase-1, IL-1β, and IL-18 in different groups were detected by Western blot analysis. Variations in the levels of (**G**) NLRP3, (**H**) Caspase-1, (**I**) IL-1β, and (**J**) IL-18 proteins indicate the ameliorative effect of PQQ intervention. Data were expressed as mean ± SD (*n* = 3–4 per group, * *p* < 0.05, ** *p* < 0.01, *** *p* < 0.001 vs. Ctl; ^#^ *p* < 0.05, ^##^ *p* < 0.01 vs. DCM).

To investigate whether PQQ ameliorates T2DM-associated cardiac injury through modulation of mitophagy, the expression levels of mitophagy-related proteins, including Parkin, Beclin-1, LC3B I/II, and p62, were assessed in this study (Figure 5A–E). Compared with the control group, the expression levels of Parkin, Beclin-1, and LC3B II were significantly downregulated in the DCM group, indicating impaired mitophagy and disruption of mitochondrial quality control. In contrast, p62 expression was markedly upregulated in the DCM group, further reflecting reduced autophagic activity. Following PQQ treatment, the high-dose group exhibited significant upregulation of Parkin, Beclin-1, and LC3B II, along with a notable decrease in p62 expression, indicating a trend toward restoration of mitophagy function.

To further explore the regulatory effects of PQQ on the NLRP3 inflammasome pathway, the expression levels of key components, including NLRP3, Caspase-1, IL-1β, and IL-18, were evaluated (Figure 5F–J). The results revealed that the expression of NLRP3, Caspase-1, IL-1β, and IL-18 was significantly increased in the DCM group induced by T2DM, indicating activation of the NLRP3 inflammasome and enhanced maturation of inflammatory mediators. In contrast, treatment with 20 mg/kg BW/day and 40 mg/kg BW/day of PQQ resulted in a marked reduction in the expression levels of these proteins.

### 3.6. The Effects of PQQ on Mitochondrial Quality Control and Pyroptosis of Myocardial Cells

**Figure 6:**
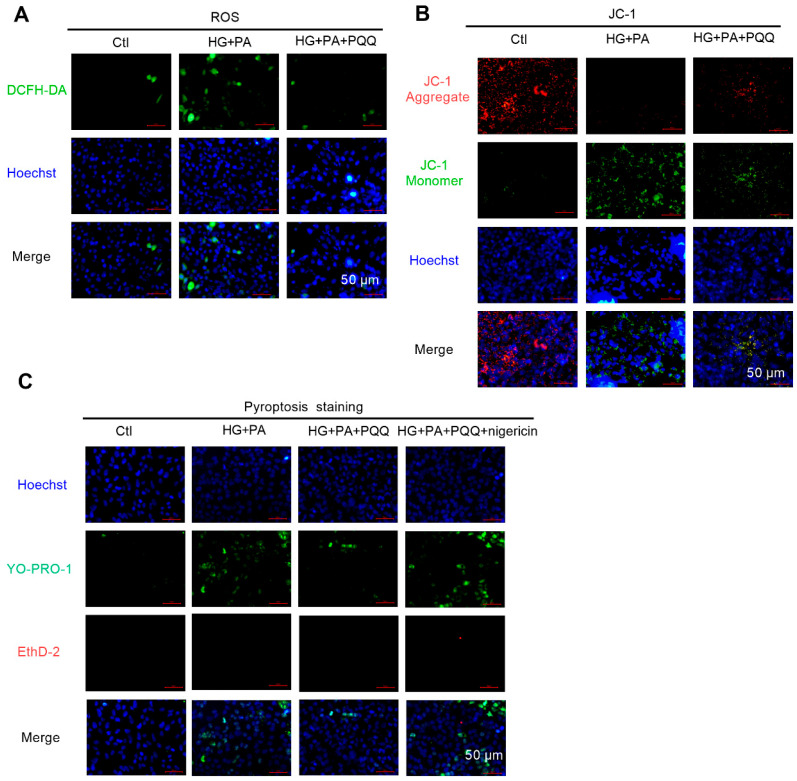
The effects of PQQ on mitochondrial quality control and pyroptosis of myocardial cells. (**A**) Intracellular reactive oxygen species (ROS) levels and (**B**) mitochondrial membrane potential levels in AC16 cells were measured using the DCFH-DA detection kit and the JC-1 mitochondrial membrane potential detection kit; magnification 200×; *n* = 3 per group. (**C**) Pyroptosis staining of AC16 cells was measured using YO-PRO-1 and EthD-2 dye; magnification 200×; *n* = 3 per group.

To evaluate the regulatory effects of PQQ on T2DM-associated oxidative stress of myocardial cells, this study employed the DCFH-DA fluorescence assay to measure ROS levels in AC16 cells (Figure 6A). The results demonstrated that HG + PA treatment significantly enhanced ROS production, as evidenced by a marked increase in green fluorescence intensity, indicating exacerbated oxidative stress. Following 10 nmol/L PQQ intervention, ROS levels were significantly reduced, and the fluorescence intensity was attenuated, suggesting that PQQ effectively mitigates oxidative stress damage. Mitochondrial membrane potential (ΔΨm) was further assessed using the JC-1 fluorescence probe (Figure 6B). ΔΨm was significantly decreased in the HG + PA group, indicating mitochondrial dysfunction and a strong association with excessive ROS generation. Notably, PQQ treatment significantly restored ΔΨm levels, effectively attenuating the membrane potential decline induced by high glucose and high lipid conditions.

To investigate the regulatory effects of PQQ on pyroptosis induced by high glucose and high lipid conditions, cellular pyroptosis was assessed via staining analysis (Figure 6C). The results revealed that in the HG + PA group, YO-PRO-1 fluorescence (green) was significantly increased. YO-PRO-1, a small, membrane-impermeable but pyroptosis-permeable dye, selectively labels cells with compromised membranes and serves as an early indicator of pyroptosis. Compared with the HG + PA group, PQQ treatment effectively reduced cellular pyroptosis. However, upon treatment with nigericin, an NLRP3 agonist, the green fluorescence intensity increased again, indicating that the NLRP3 activator nigericin reverses the inhibitory effects of PQQ. In summary, PQQ significantly reduces excessive ROS production, improves mitochondrial membrane potential dysfunction, and alleviates pyroptosis by inhibiting NLRP3 inflammasome activation under high glucose and high lipid conditions.

### 3.7. The Effect of PQQ on Mitophagy and Pyroptosis Pathway of AC16 Cells Under High Glucose and High Lipid Conditions

**Figure 7:**
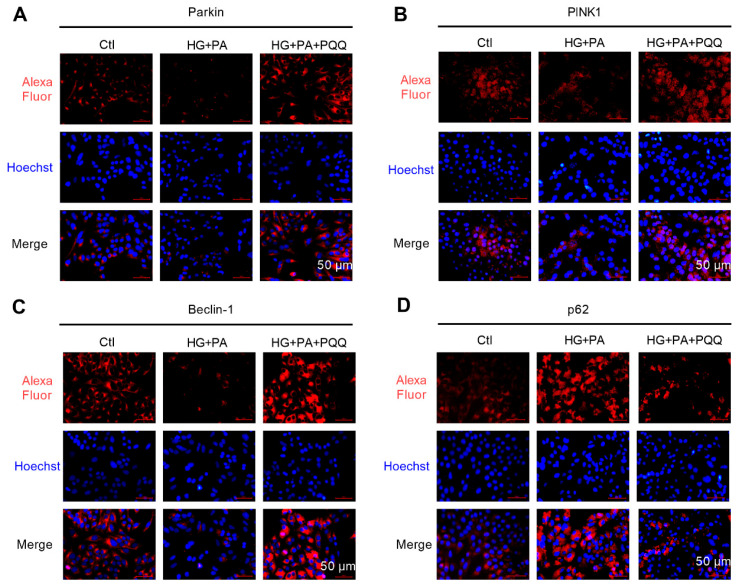
The effect of PQQ on mitophagy pathway of AC16 cells under high glucose and high lipid conditions. The protein expression levels of (**A**) Parkin, (**B**) PINK1, (**C**) Beclin-1, and (**D**) p62 in PQQ + HG + PA-treated AC16 cardiomyocytes were detected by immunofluorescence; magnification 200×; *n* = 3 per group.

**Figure 8:**
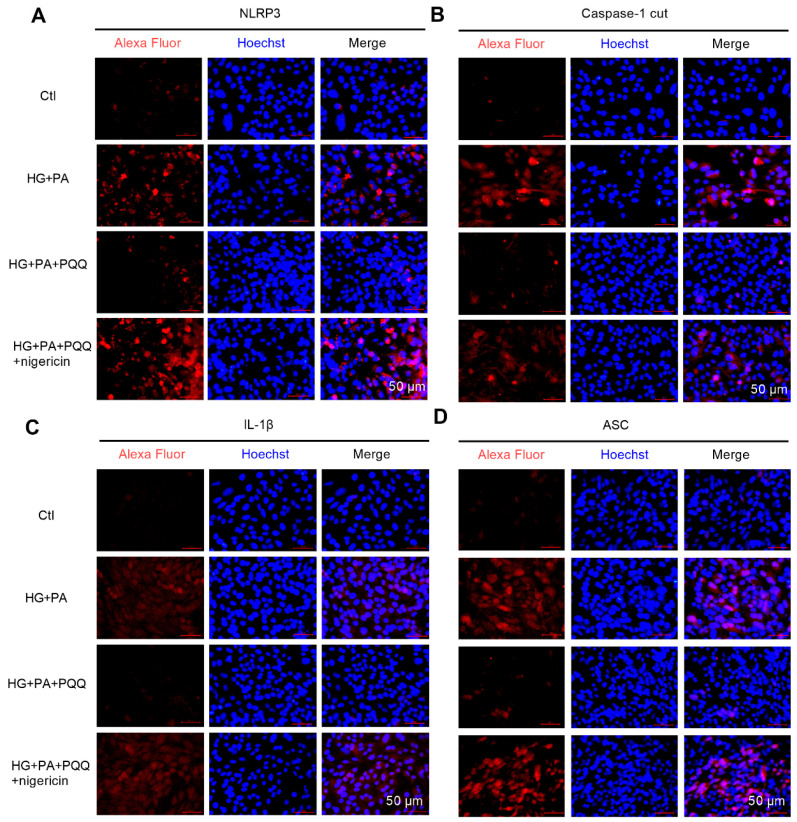
The effect of PQQ on pyroptosis pathway of AC16 cells under high glucose and high lipid conditions. The protein expression levels of (**A**) NLRP3, (**B**) Caspase-1 cut, (**C**) IL-1β, and (**D**) ASC in PQQ + HG + PA-treated AC16 cardiomyocytes were detected by immunofluorescence; magnification 200×; *n* = 3 per group.

To investigate the effects of PQQ on mitophagy and NLRP3 inflammasome signaling pathways under high glucose and high lipid conditions at the cellular level, this study utilized immunofluorescence staining to assess the expression levels of key proteins involved in mitophagy and pyroptosis (Figure 7 and Figure 8). The results demonstrated that HG + PA treatment significantly reduced the red fluorescence signals of PINK1, Parkin, and Beclin-1 in AC16 cells, whereas the fluorescence intensities of p62, NLRP3, Caspase-1 cut, IL-1β, and IL-18 were markedly increased. These findings suggest that mitophagy was impaired and accompanied by activation of the NLRP3 inflammasome. Following 10 nmol/L PQQ intervention, these alterations were notably ameliorated. However, co-administration of an NLRP3 agonist abrogated the protective effects of PQQ on the inflammasome pathway. Collectively, these findings demonstrate that PQQ mitigates HG + PA-induced cellular damage by enhancing the mitochondrial autophagy pathway and inhibiting the NLRP3-mediated pyroptosis in cardiomyocytes.

## 4. Discussion

In this study, we demonstrated that pyrroloquinoline quinone (PQQ), a naturally occurring redox cofactor, provides significant protection against diabetic cardiomyopathy (DCM) induced by type 2 diabetes mellitus (T2DM). Using a murine model combining a high-fat diet with low-dose streptozotocin, we showed that long-term oral PQQ treatment improved glucose tolerance, preserved cardiac function, and mitigated structural remodeling characterized by fibrosis and hypertrophy. PQQ enhanced mitophagy through the activation of PINK1/Parkin signaling, promoted the turnover of damaged mitochondria, and suppressed NLRP3 inflammasome activation, leading to reduced oxidative stress and decreased pyroptosis. In vitro experiments in AC16 human cardiomyocytes further validated these findings by demonstrating that PQQ alleviates lipotoxic and glucotoxic stress, restores mitochondrial homeostasis, and blunts inflammatory responses. Collectively, these results identify PQQ as a dual-action modulator of mitochondrial quality control and NLRP3 inflammasome signaling, underscoring its translational potential for T2DM-related cardiac disease.

PQQ has been shown to possess a favorable safety profile. In a toxicological evaluation conducted by our collaborating laboratory, the acute oral toxicity test in SD rats demonstrated an LD50 greater than 3.69 g/kg BW. In a 90-day repeated oral toxicity study, no treatment-related adverse effects were observed at a dose of 0.4 g/kg BW/day, suggesting a relatively wide safety margin for potential therapeutic applications. The functions and toxicological characteristics of PQQ further support our investigation into its potential role in cardiometabolic disorders.

PQQ has been studied in various pathological conditions, where its beneficial actions are largely attributed to the regulation of mitochondrial homeostasis, oxidative stress, and inflammatory signaling. In neurological disorders, PQQ protects against Parkinson’s disease by stimulating mitochondrial biogenesis via the CREB/PGC-1α pathway, thereby enhancing SH-SY5Y cells’ survival [33]. PQQ could ameliorate dysregulated lipid metabolism and restore spermatogenic function in mice with HFD-induced obesity. These effects were associated with the inhibition of proprotein convertase subtilisin/kexin type 9 (PCSK9), suppression of NLRP3 inflammasome activity, and upregulation of endogenous antioxidant enzymes, collectively enhancing mitochondrial turnover and facilitating testosterone biosynthesis in Leydig cells [34]. Similarly, in human renal tubular epithelial cell injury, PQQ preserves mitochondrial function and reduces ROS generation through the Keap1/Nrf2 signaling pathway [35]. These findings highlight PQQ as a versatile cytoprotective agent that converges on conserved pathways of mitochondrial quality control and inflammation regulation across multiple organ systems.

Our data highlight the central role of mitochondrial dysfunction and sterile inflammation in the pathogenesis of DCM and demonstrate that PQQ simultaneously targets both processes. By enhancing PINK1/Parkin-mediated mitophagy, PQQ facilitated the clearance of dysfunctional mitochondria, which otherwise serve as a persistent source of ROS and energetic failure. In parallel, PQQ attenuated the NLRP3-Caspase-1-IL-1β/IL-18 signaling axis, thereby reducing pyroptotic cell death and the propagation of inflammatory injury. These dual effects suggest that PQQ interrupts the self-perpetuating cycle whereby mitochondrial damage drives inflammasome activation, which in turn exacerbates cardiomyocyte dysfunction and remodeling. This integrated regulation provides a strong mechanistic rationale for the observed improvements in myocardial structure and function.

In the management of T2DM and its associated cardiovascular complications, SGLT2 inhibitors and GLP-1 receptor agonists have emerged as key therapeutic agents with well-documented cardiovascular benefits [36,37]. For instance, the EMPA-REG OUTCOME trial demonstrated that patients treated with empagliflozin experienced significantly lower rates of all-cause mortality, cardiovascular mortality, and hospitalization for heart failure compared to those receiving placebo [38]. PQQ has shown potential as an adjuvant treatment and preventive strategy for heart diseases associated with T2DM. PQQ may serve as a preventive therapeutic strategy for early intervention in patients with T2DM who have not yet developed significant cardiac dysfunction but are already exhibiting metabolic imbalances, oxidative stress, or mild myocardial remodeling, thereby delaying or preventing the progression of DCM. PQQ can be used as an adjunct to clinical therapeutic drugs. In patients receiving SGLT2 inhibitors or GLP-1 receptor agonists who exhibit limited improvement in cardiac function or who already have established myocardial lesions, the addition of PQQ may further enhance myocardial structural and functional outcomes through dual mechanisms involving the promotion of mitochondrial autophagy and the inhibition of the NLRP3 inflammasome.

Several limitations of this study should be acknowledged. This study only included male mice to avoid physiological variability caused by the estrous cycle, which is a common design in preliminary mechanistic studies. Nevertheless, sex differences exist in the pathogenesis of diabetic cardiomyopathy and drug responsiveness, so our findings may not be directly generalizable to female mice with diabetes. Relatedly, we did not adopt genetic strategies such as gene knockout, siRNA knockdown or plasmid overexpression to strictly verify the causal roles of PINK1/Parkin-dependent mitophagy and NLRP3 inflammasome signaling. Although the combination of our phenotypic and molecular findings and nigericin intervention experiments supports the proposed mechanism, further genetic validation is still required. In addition, we only detected the static expression of mitophagy-related proteins rather than dynamic autophagic flux, and did not directly verify the expression and translocation of TXNIP, leaving this key node of the regulatory cascade for future investigation.

This study also has certain translational and interpretative limitations. Although PQQ has been detected in multiple human vital organs, detailed oral pharmacokinetic profiles and long-term safety evidence in humans are still lacking. The human equivalent dose of our highest PQQ intervention exceeds the current nutritional supplement standard, and clinical studies are needed to determine the optimal safe therapeutic dose for DCM patients. Moreover, in vivo findings cannot completely distinguish direct cardiac protection from indirect systemic metabolic improvement; while in vitro data confirm the direct protective effect of PQQ on cardiomyocytes, further research is required to differentiate the relative contribution of local cardiac action and whole-body metabolic regulation. Despite the above limitations, our work still provides valuable mechanistic insights into the cardioprotective effects of PQQ in male mice with type 2 diabetes.

## 5. Conclusions

In summary, this study identifies PQQ as a promising therapeutic candidate for diabetic cardiomyopathy by enhancing mitochondrial quality control and suppressing NLRP3 inflammasome-mediated pyroptosis. Unlike current glucose-lowering drugs with cardiovascular benefit, PQQ acts directly at the level of mitochondrial and inflammatory signaling, addressing key pathogenic drivers of DCM. These findings provide both mechanistic insights and translational promise, supporting the future exploration of PQQ either as a stand-alone intervention or as an adjunct to established antidiabetic therapies in order to reduce the burden of diabetes-associated heart injury.

## Data Availability

The datasets used and/or analyzed during the current study are available from the corresponding author upon reasonable request.

## Supplementary Materials

The following supporting information can be downloaded at: https://www.mdpi.com/article/10.3390/metabo16050340/s1. Figure S1: Uncropped Western blot images. Figure S2: Uncropped microscopy images.
