# Supplementary material for: Pyrroloquinoline Quinone Mitigates Type 2 Diabetes-Induced Cardiac Injury Through Mitochondrial Quality Control and Inhibition of NLRP3-Dependent Pyroptosis
Source: Metabolites. 2026 May 19;16(5):340. doi: 10.3390/metabo16050340 (PMC13209680; doi:10.3390/metabo16050340)
Supplement: Supplementary file 1 [file metabolites-16-00340-s001.zip › Figure S1 Uncropped western blot images.pdf]

Figure3(1)

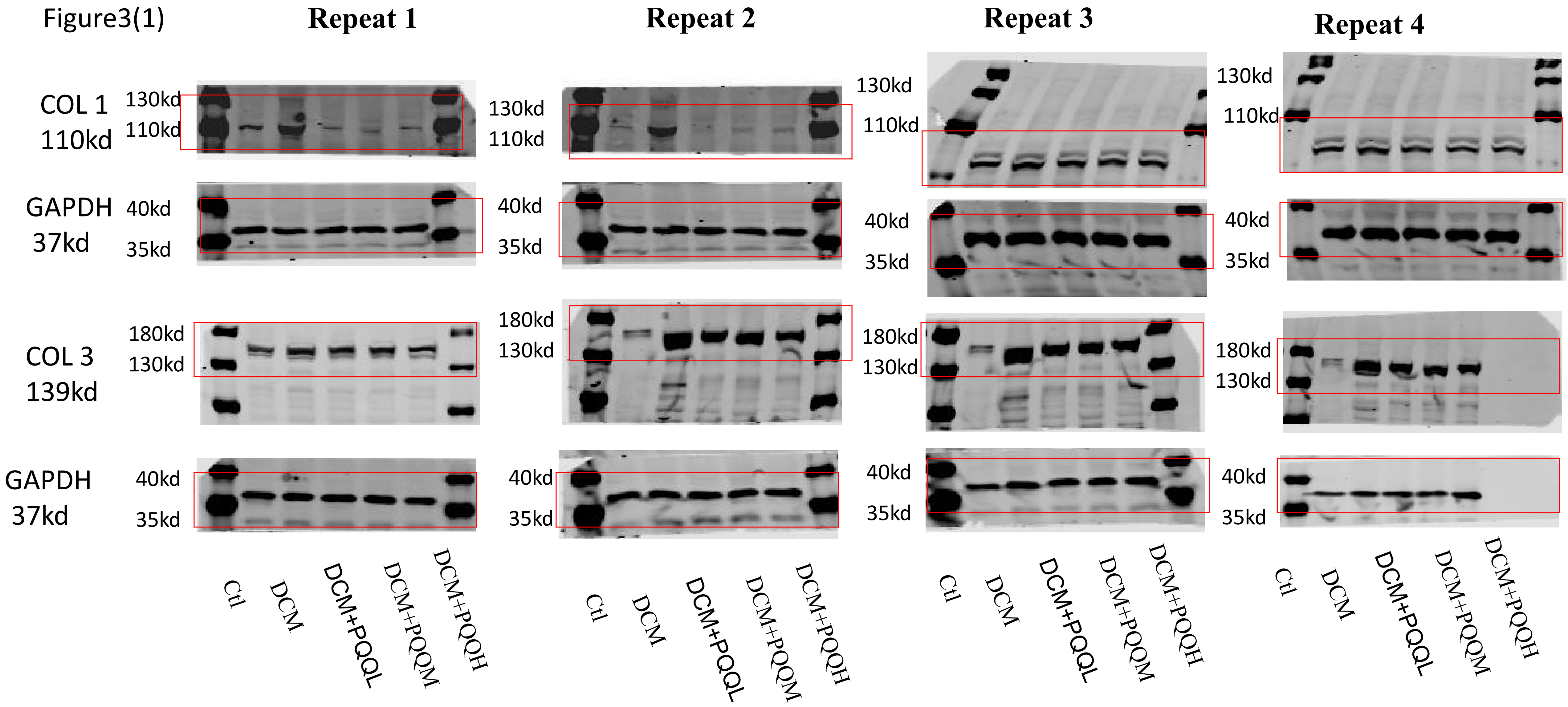
Repeat 1
Repeat 2
Repeat 3
Repeat 4
COL 1
110kd
GAPDH
37kd
COL 3
139kd
GAPDH
37kd
130kd
110kd
40kd
35kd
180kd
130kd
Ctl
DCM
DCM+PQQL
DCM+PQQM
DCM+PQQH

Figure3(2)

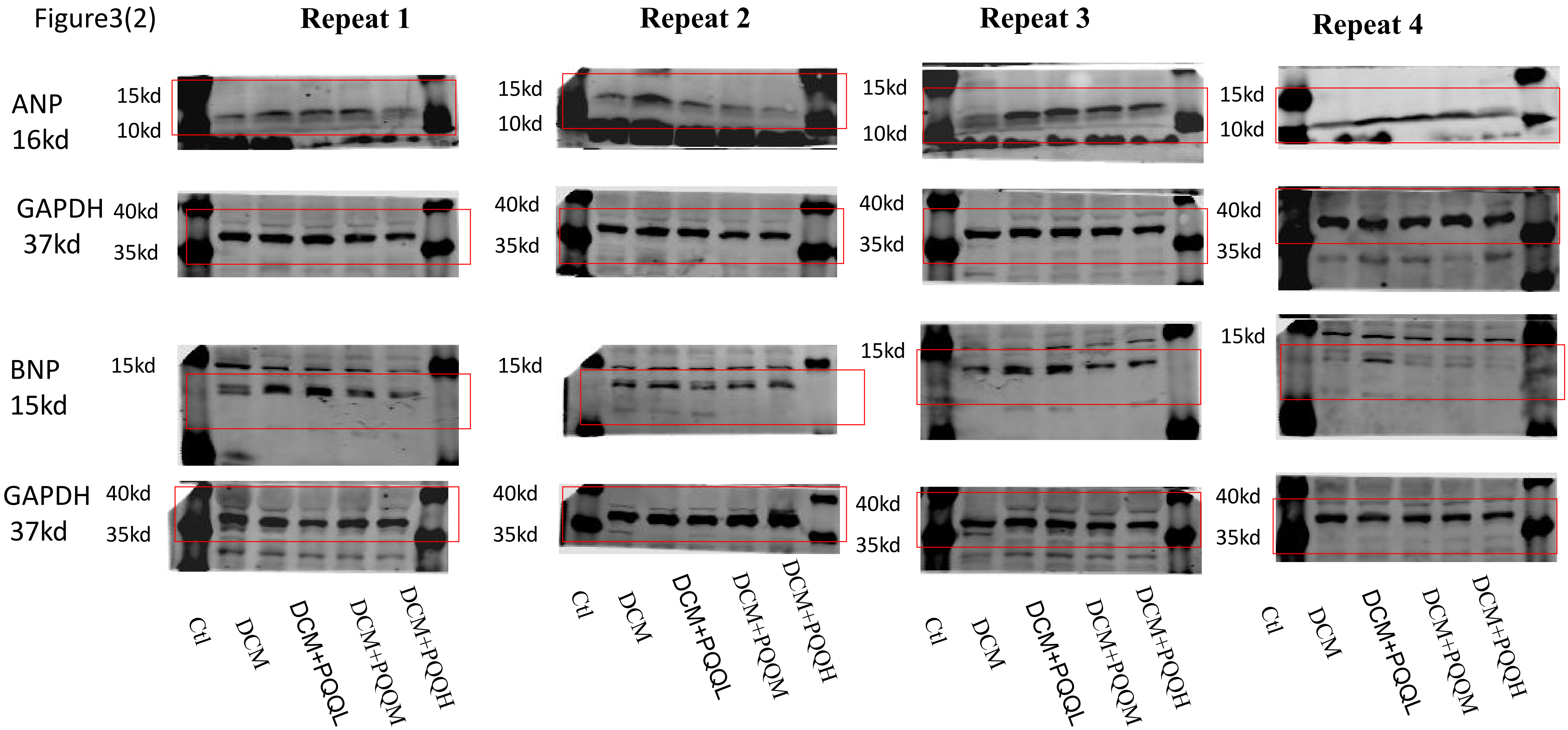
Repeat 1
Repeat 2
Repeat 3
Repeat 4
ANP
16kd
GAPDH
37kd
BNP
15kd
GAPDH
37kd
15kd
10kd
40kd
35kd
Ctl
DCM
DCM+PQQL
DCM+PQQM
DCM+PQQH

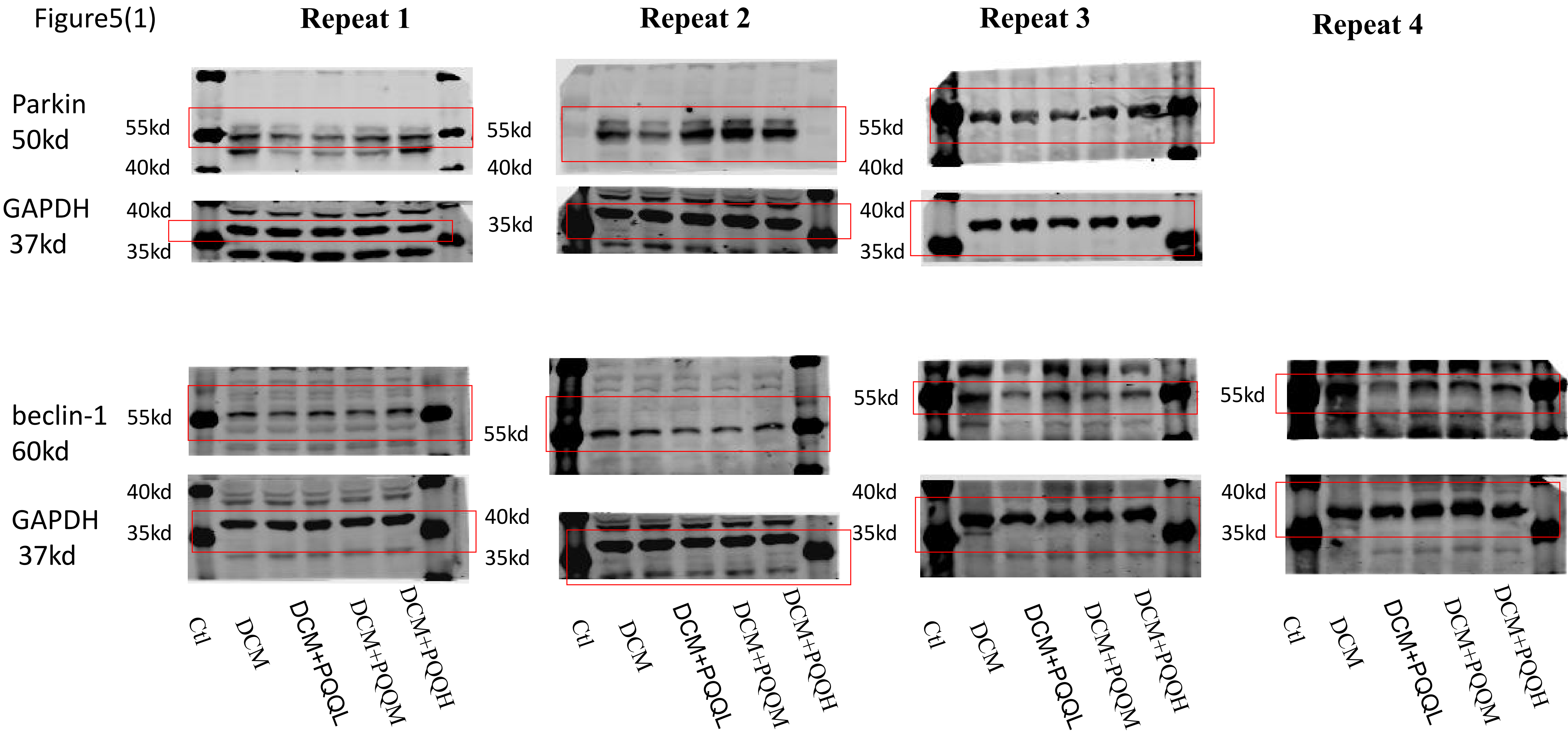

Figure5(1)
Repeat 1
Repeat 2
Repeat 3
Repeat 4
Parkin
50kd
GAPDH
37kd
beclin-1
60kd
GAPDH
37kd
55kd
40kd
35kd
Ctl
DCM
DCM+PQQL
DCM+PQQM
DCM+PQQH

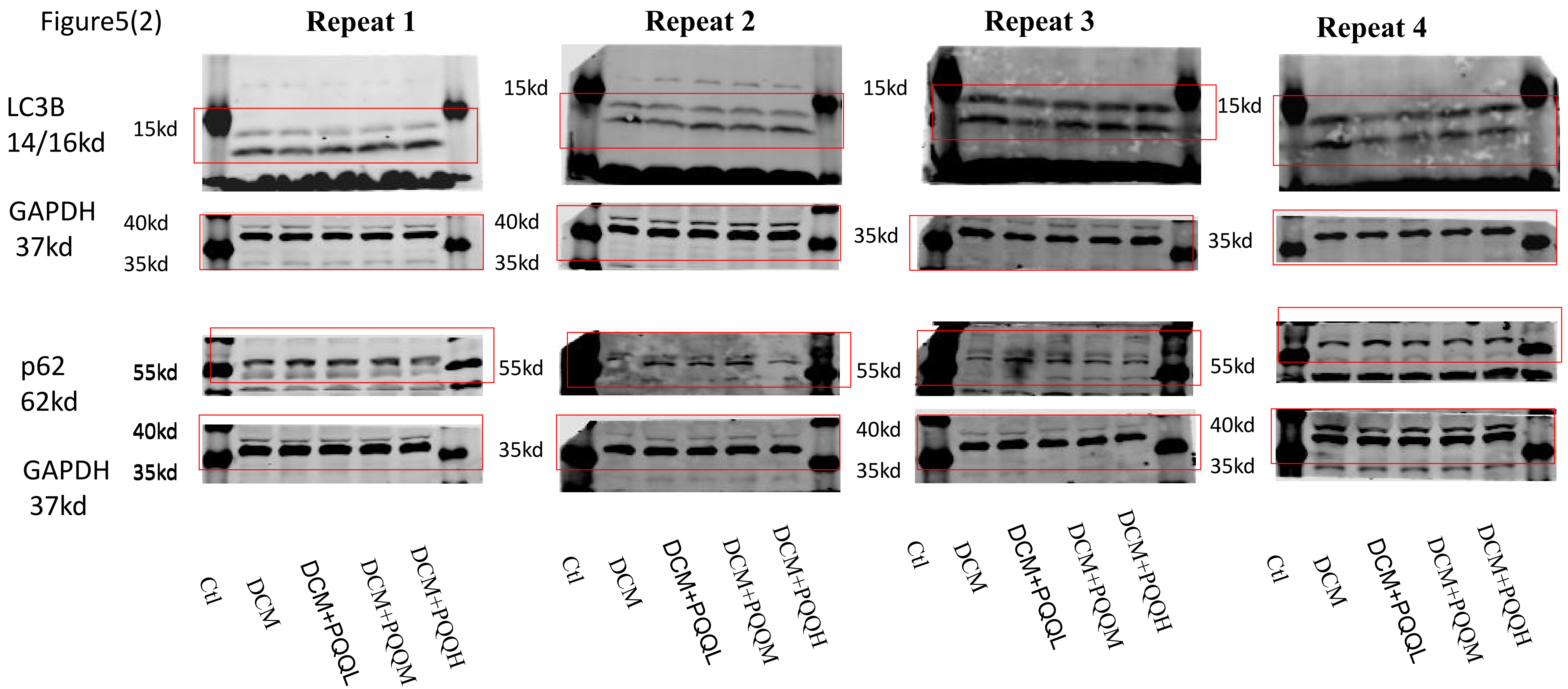
Figure5(2)
Repeat 1
Repeat 2
Repeat 3
Repeat 4
LC3B
14/16kd
GAPDH
37kd
p62
62kd
GAPDH
37kd
15kd
40kd
35kd
55kd
Ctl
DCM
DCM+PQQL
DCM+PQQM
DCM+PQQH

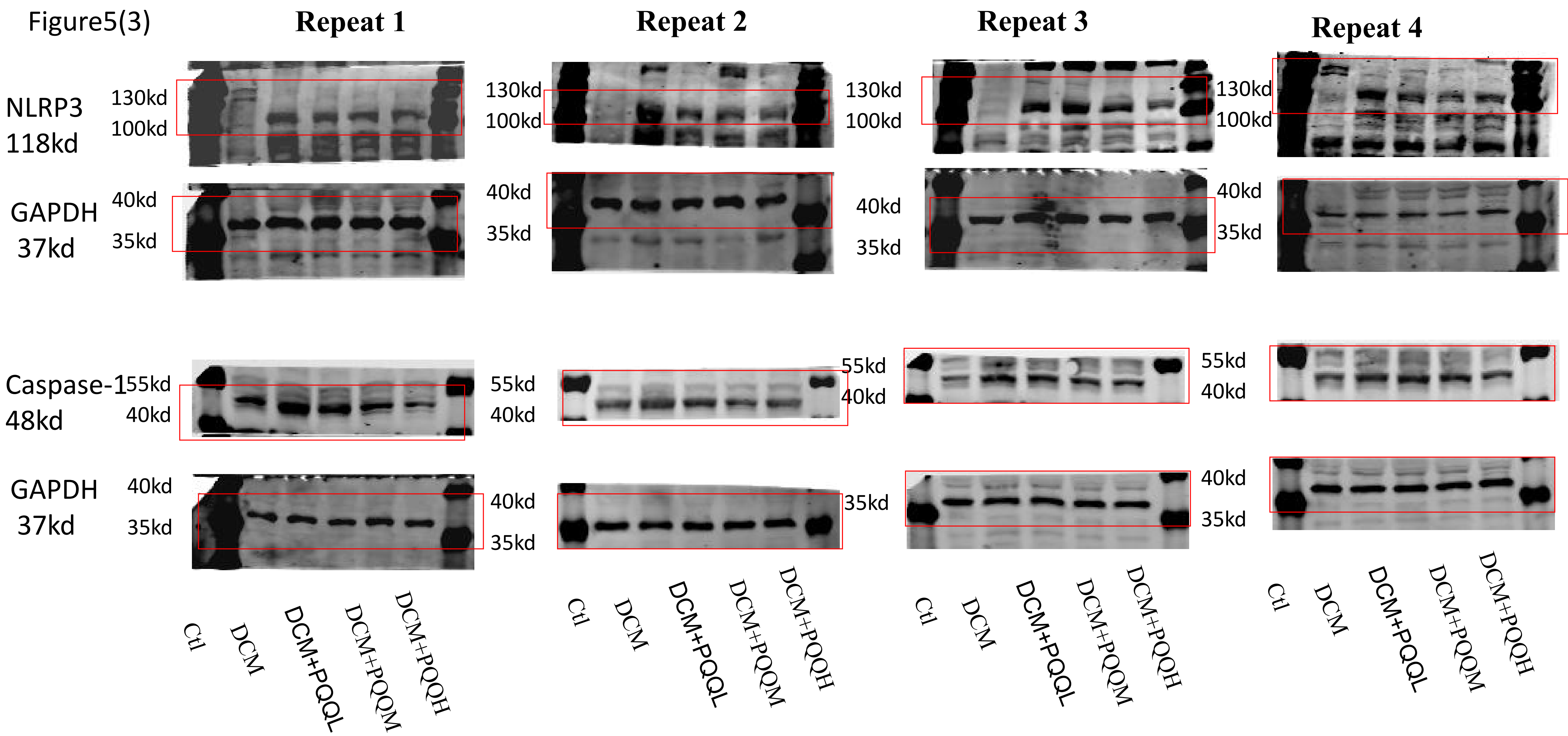

Figure5(3)
Repeat 1
Repeat 2
Repeat 3
Repeat 4
NLRP3
118kd
130kd
100kd
GAPDH
37kd
40kd
35kd
Caspase-1
48kd
55kd
40kd
GAPDH
37kd
40kd
35kd
Ctl
DCM
DCM+PQQL
DCM+PQQM
DCM+PQQH

Figure5(4)

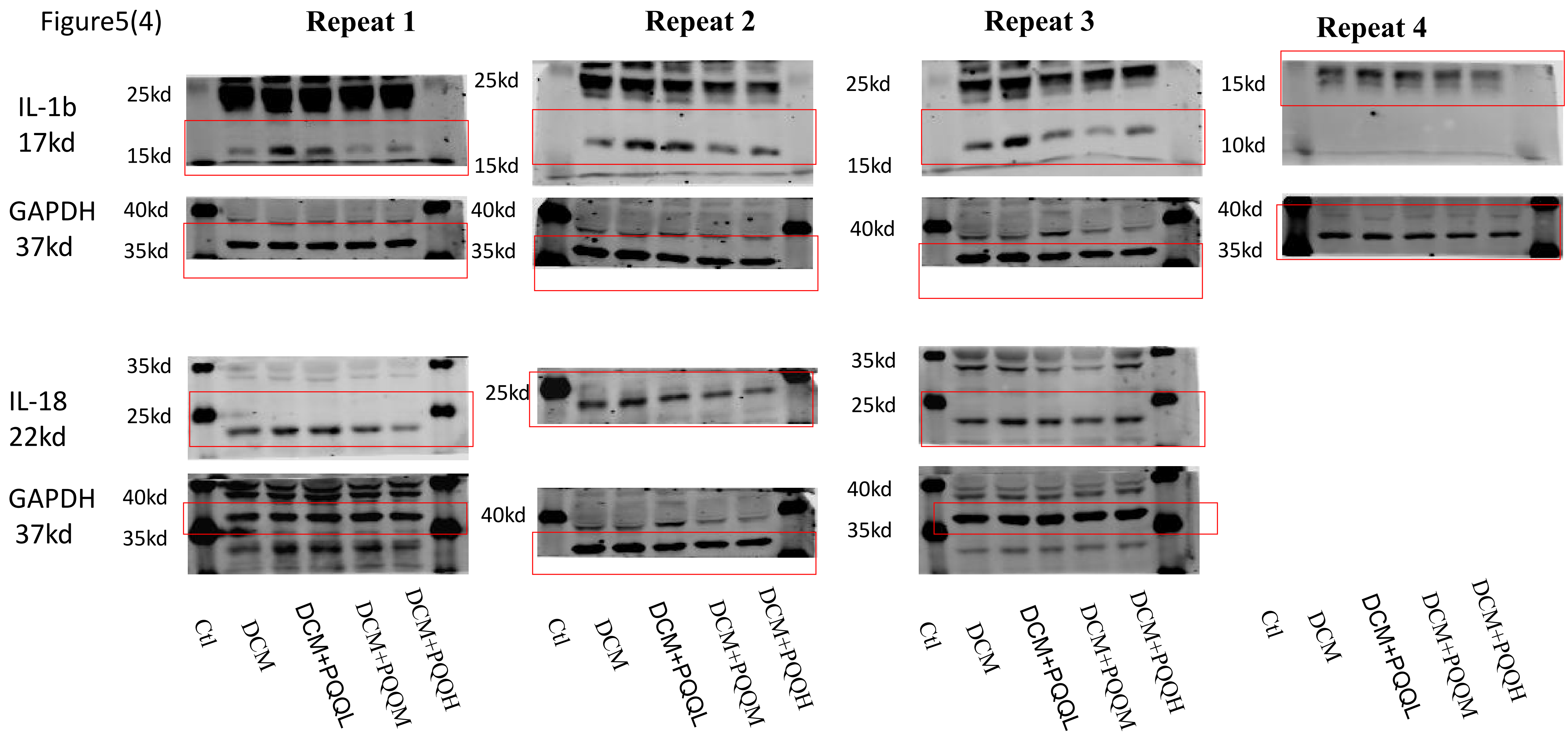
Repeat 1
Repeat 2
Repeat 3
Repeat 4
IL-1b
17kd
25kd
15kd
10kd
GAPDH
37kd
40kd
35kd
IL-18
22kd
Ctl
DCM
DCM+PQQL
DCM+PQQM
DCM+PQQH
