# Supplementary figures and images for: Pyrroloquinoline Quinone Mitigates Type 2 Diabetes-Induced Cardiac Injury Through Mitochondrial Quality Control and Inhibition of NLRP3-Dependent Pyroptosis (part 2 of 2)
Source: Metabolites. 2026 May 19;16(5):340. doi: 10.3390/metabo16050340 (PMC13209680; doi:10.3390/metabo16050340)

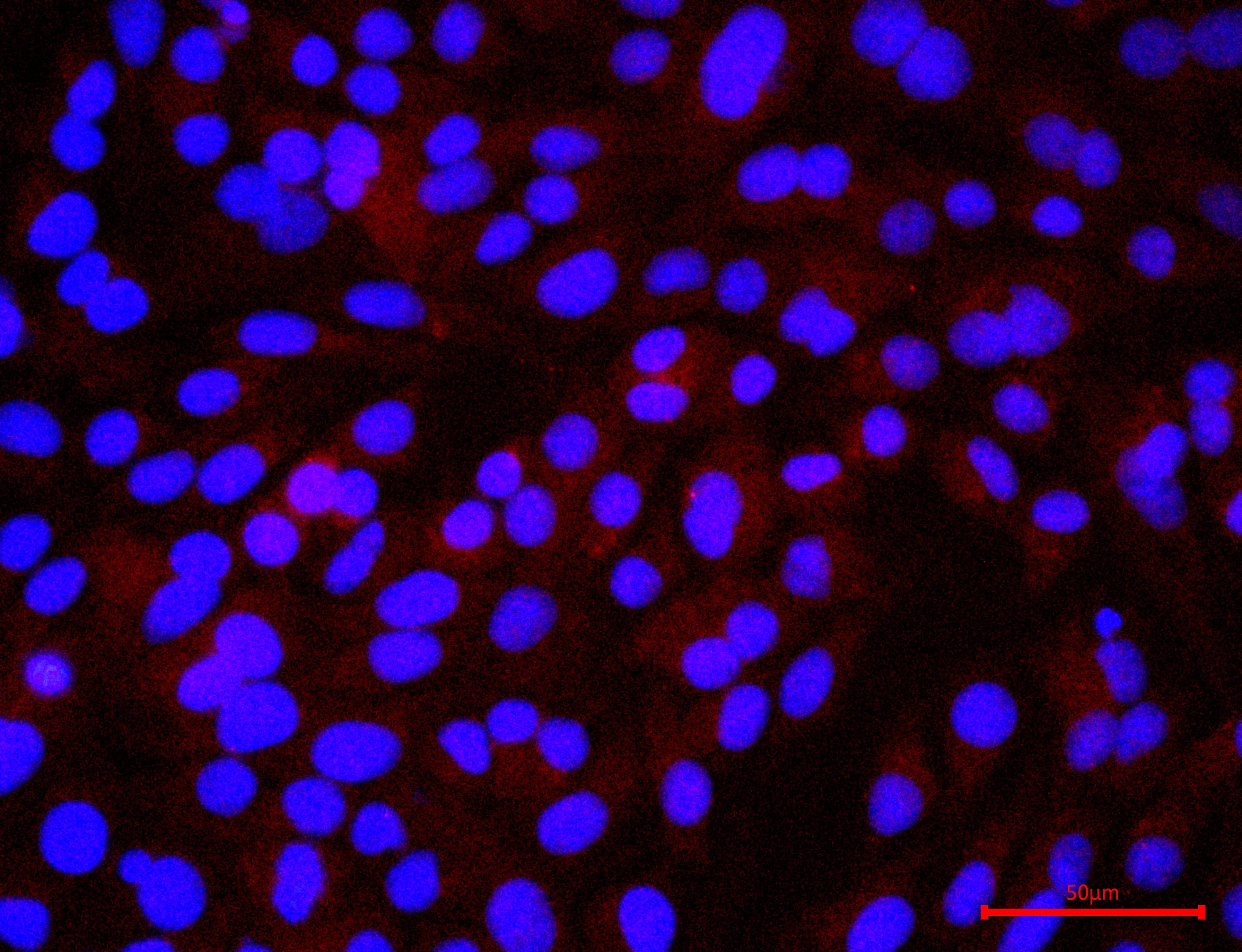

Supplement: Supplementary file 1 [file metabolites-16-00340-s001.zip › Figure S2 Uncropped microscopy images/Figure8/IL-1β/Nmerge1(1).jpg]

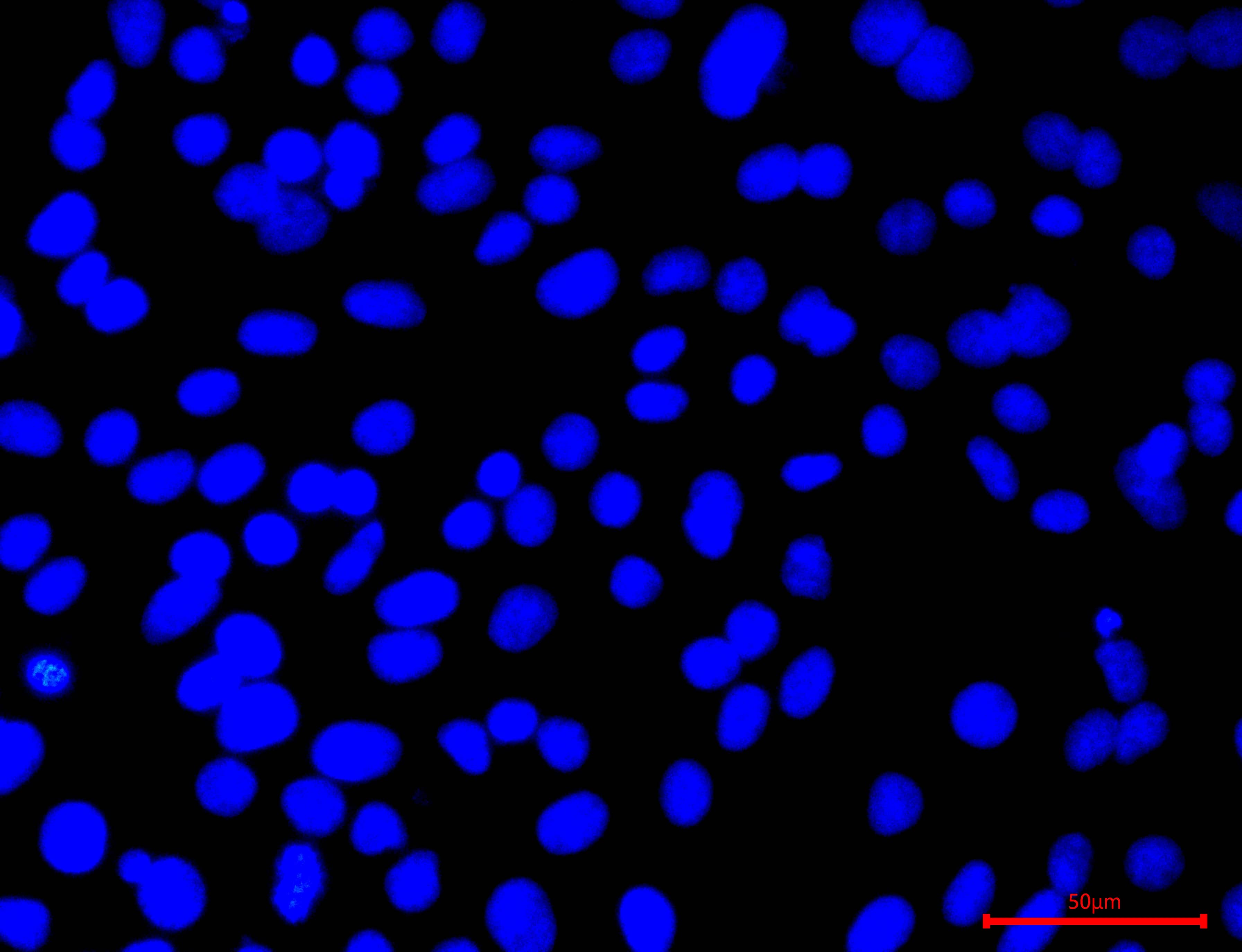

Supplement: Supplementary file 1 [file metabolites-16-00340-s001.zip › Figure S2 Uncropped microscopy images/Figure8/IL-1β/N核1(1).jpg]

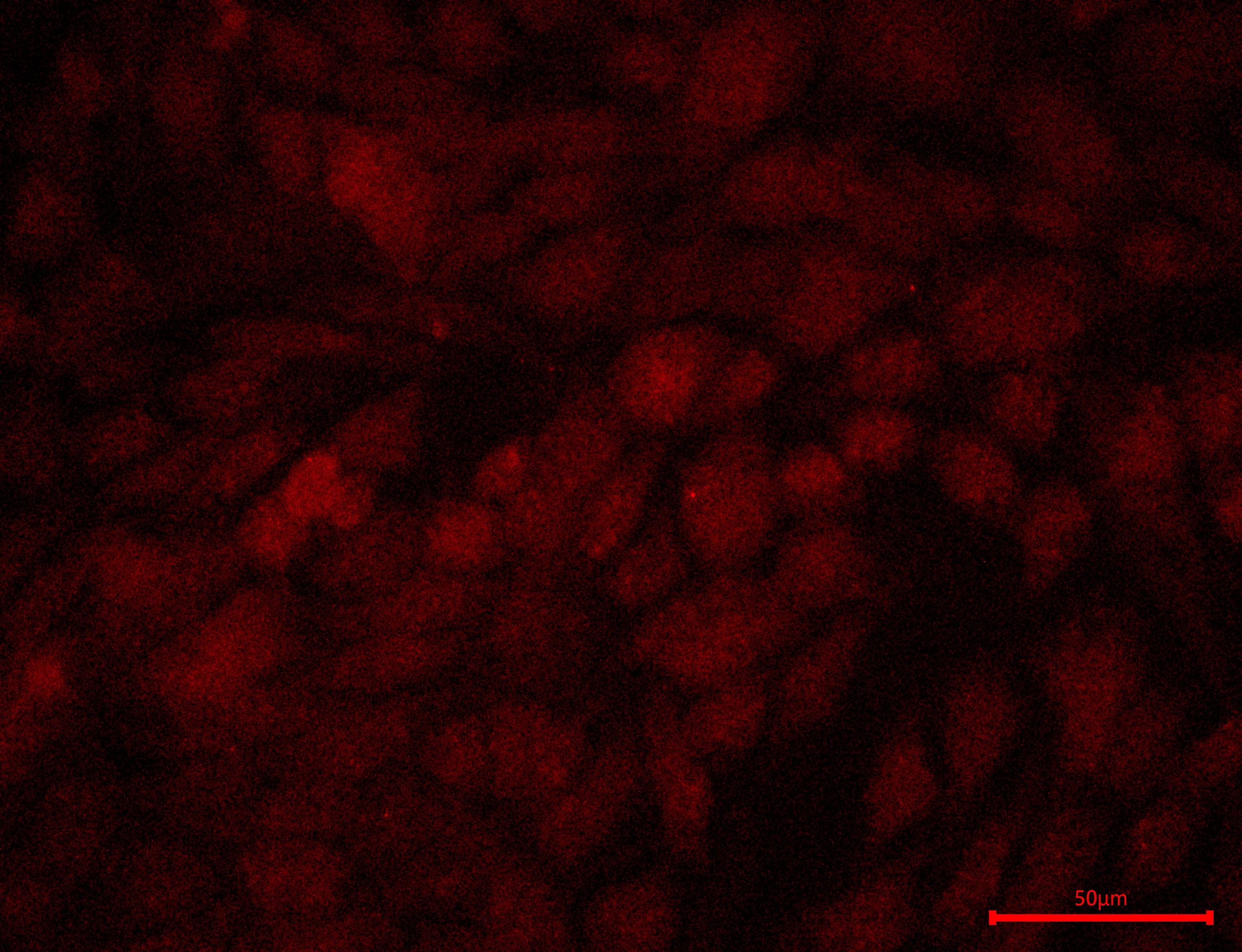

Supplement: Supplementary file 1 [file metabolites-16-00340-s001.zip › Figure S2 Uncropped microscopy images/Figure8/IL-1β/N红1(1).jpg]

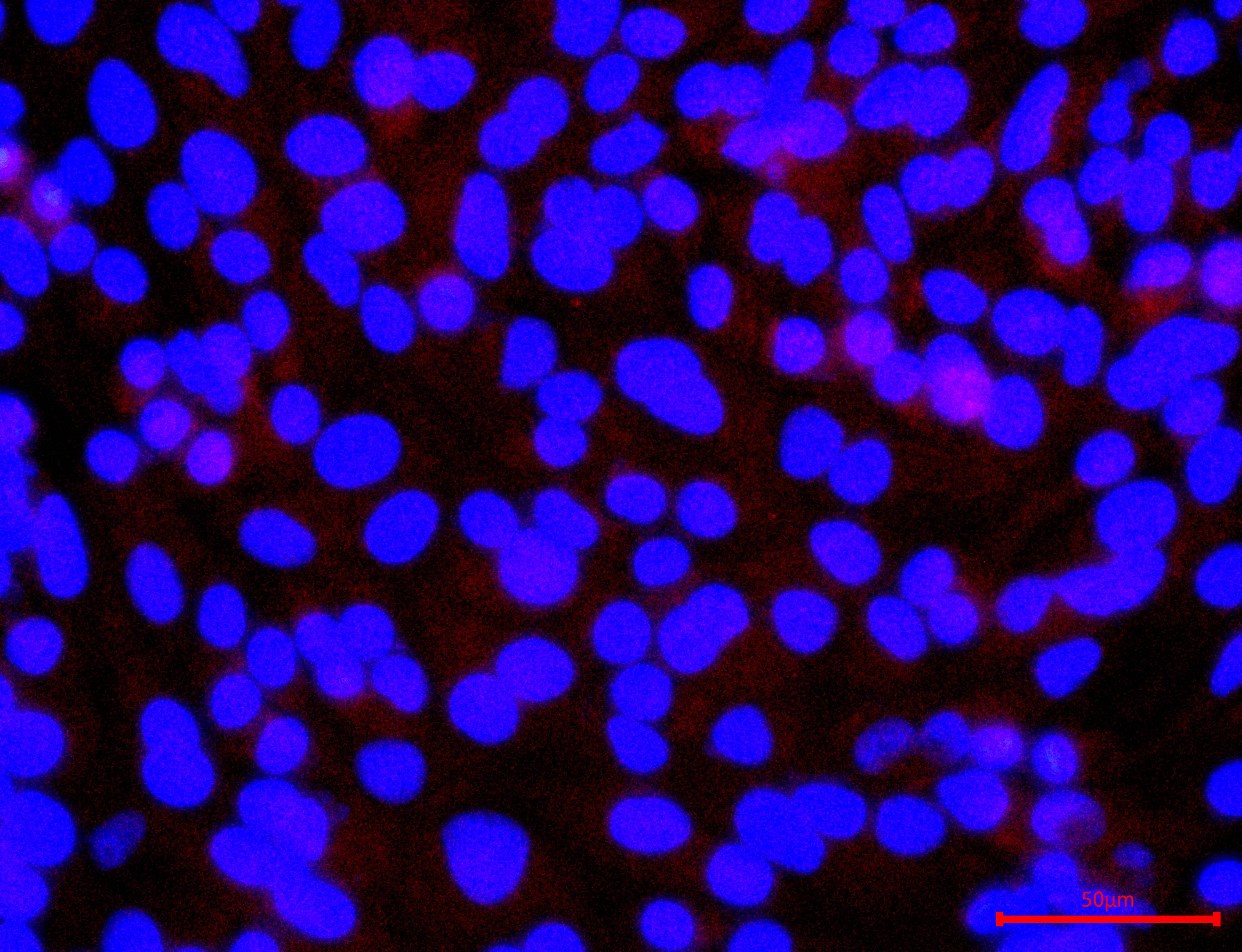

Supplement: Supplementary file 1 [file metabolites-16-00340-s001.zip › Figure S2 Uncropped microscopy images/Figure8/IL-1β/PAmerge1(1).jpg]

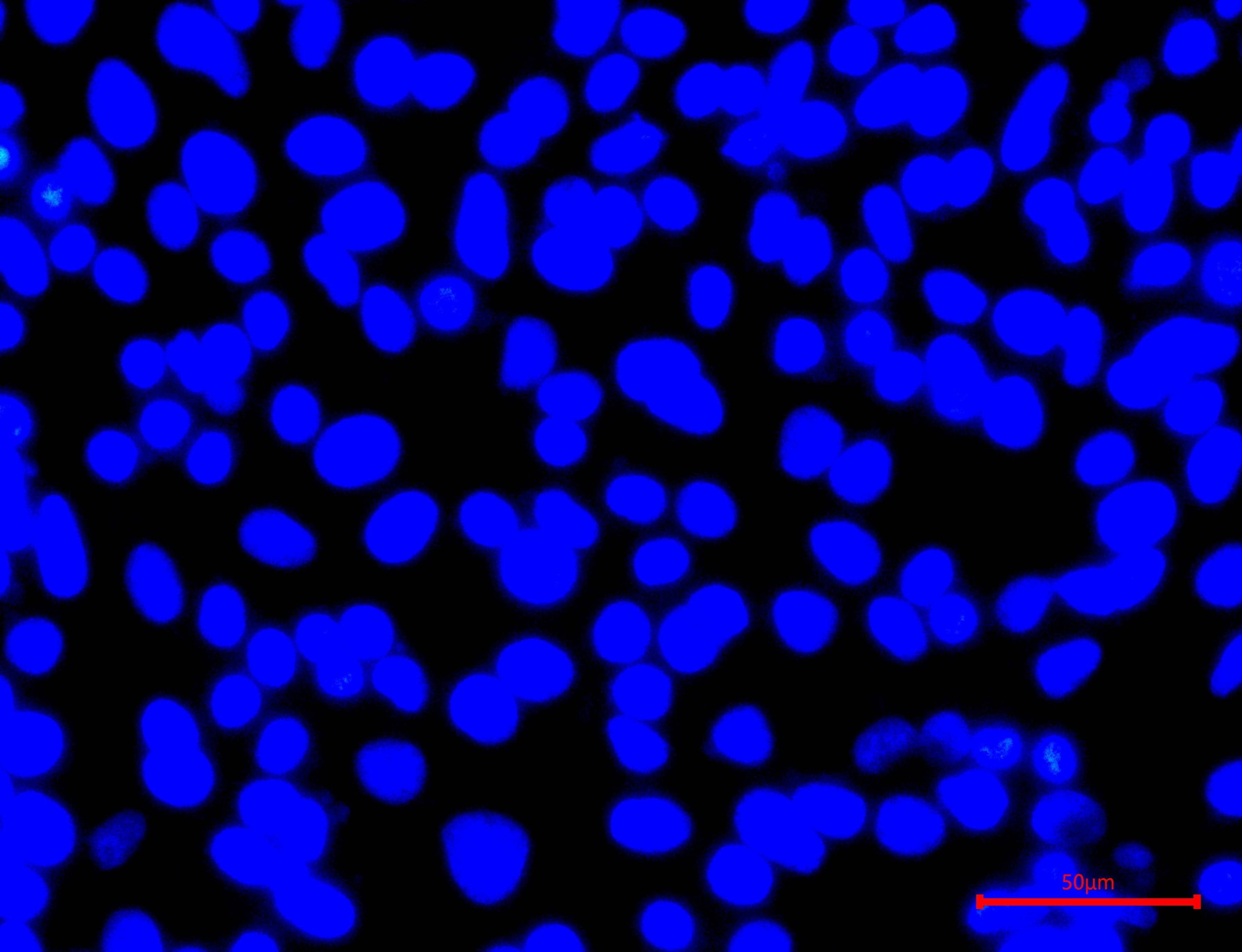

Supplement: Supplementary file 1 [file metabolites-16-00340-s001.zip › Figure S2 Uncropped microscopy images/Figure8/IL-1β/PA核1(1).jpg]

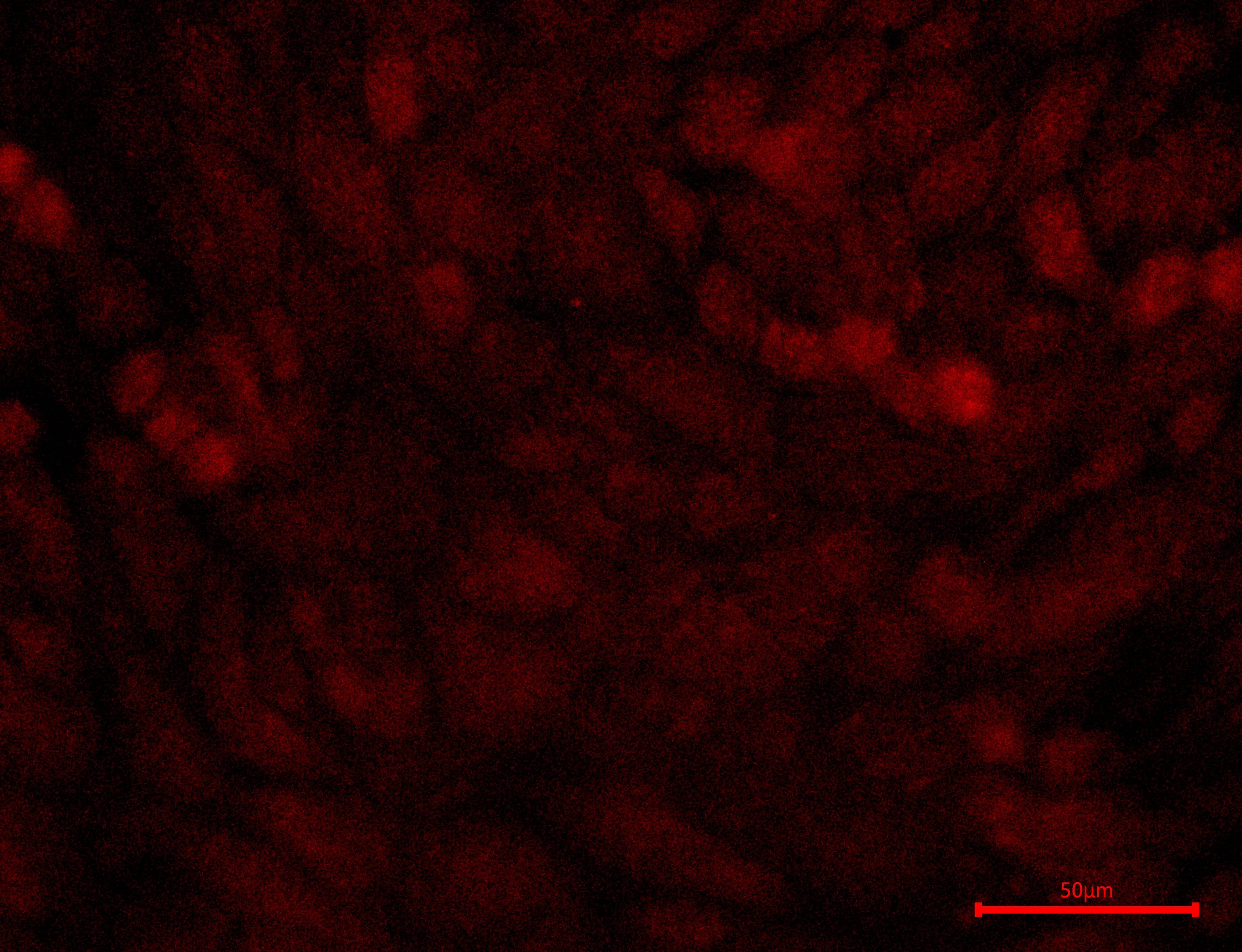

Supplement: Supplementary file 1 [file metabolites-16-00340-s001.zip › Figure S2 Uncropped microscopy images/Figure8/IL-1β/PA红1(1).jpg]

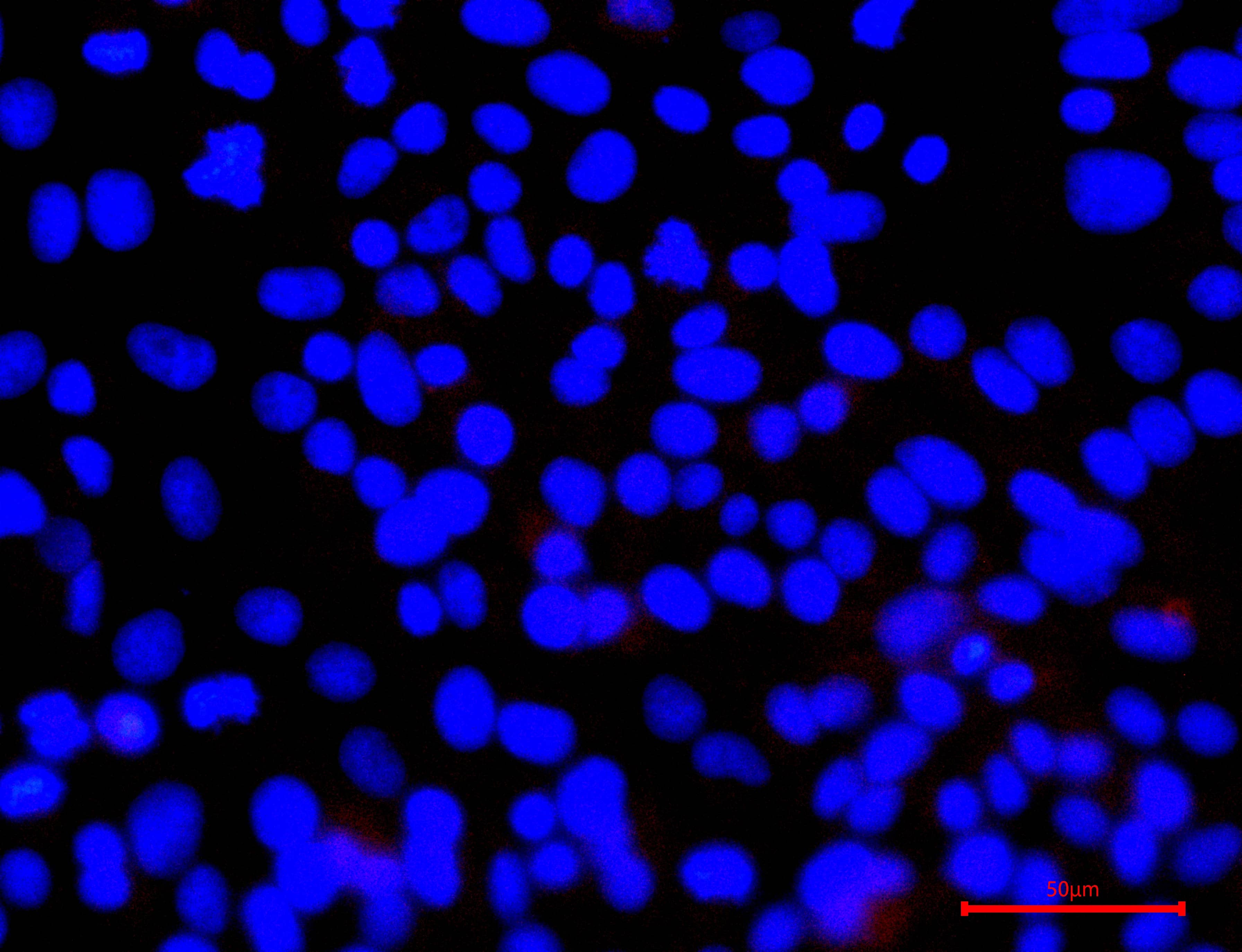

Supplement: Supplementary file 1 [file metabolites-16-00340-s001.zip › Figure S2 Uncropped microscopy images/Figure8/IL-1β/PQQmerge1(1).jpg]

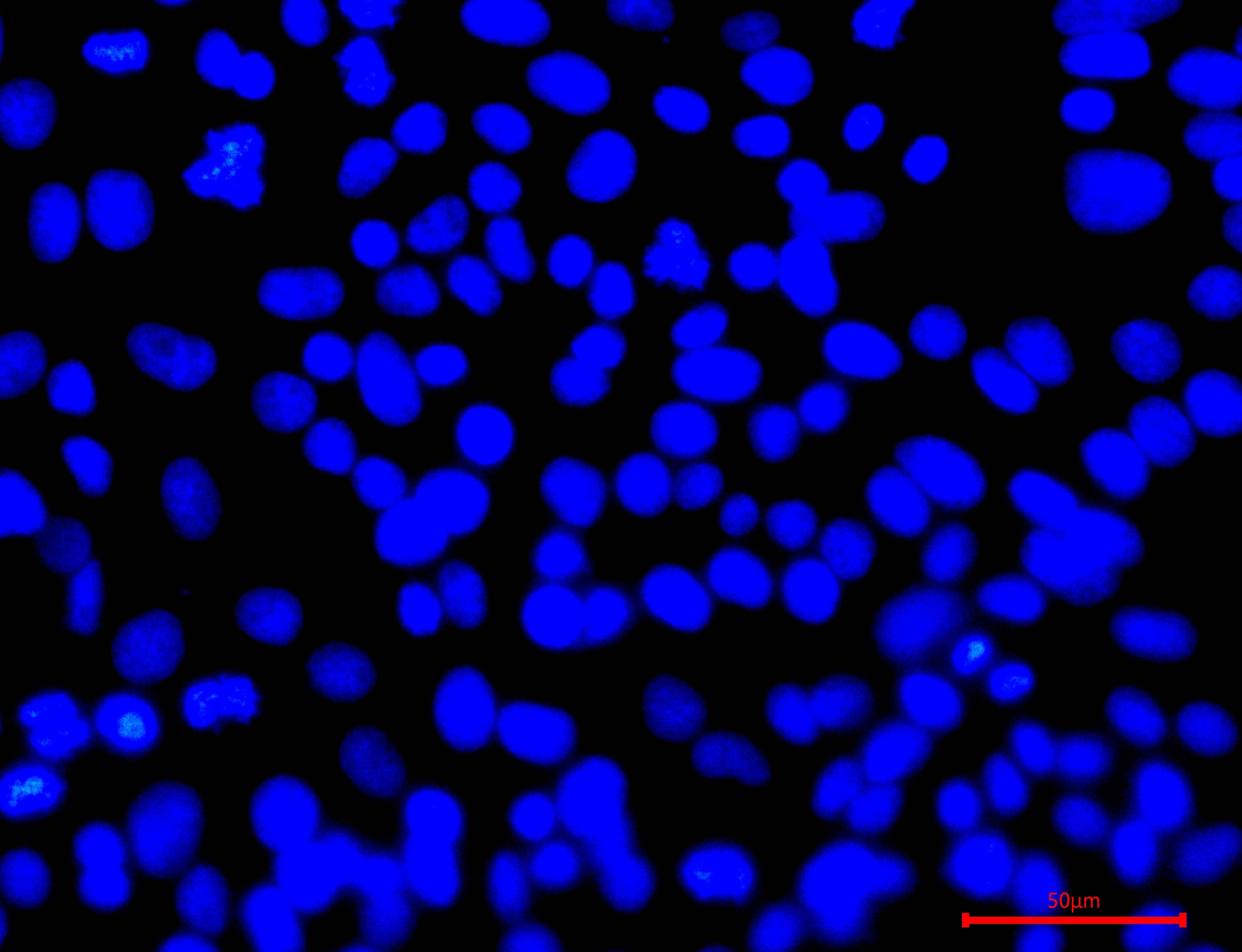

Supplement: Supplementary file 1 [file metabolites-16-00340-s001.zip › Figure S2 Uncropped microscopy images/Figure8/IL-1β/PQQ核1(1).jpg]

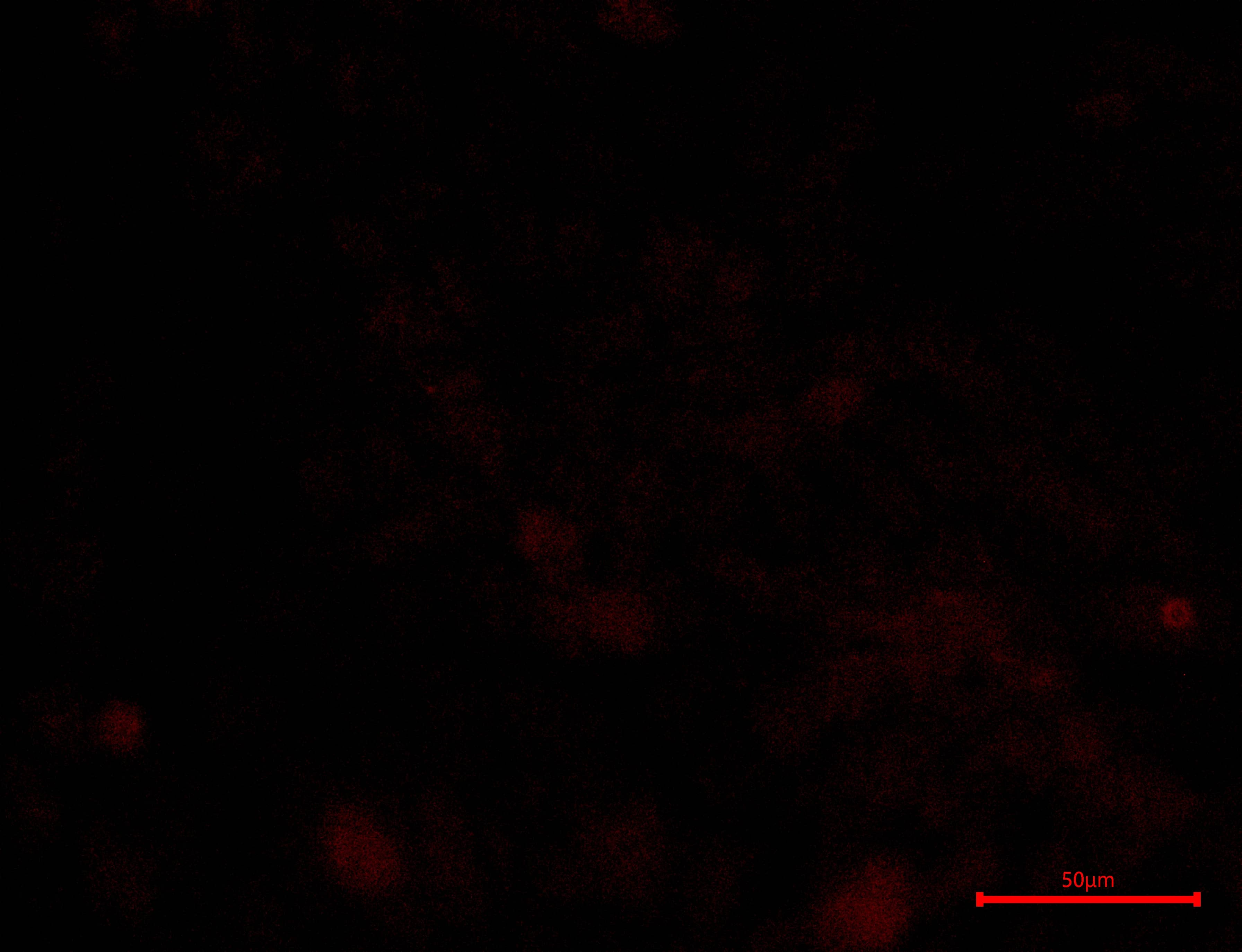

Supplement: Supplementary file 1 [file metabolites-16-00340-s001.zip › Figure S2 Uncropped microscopy images/Figure8/IL-1β/PQQ红1(1).jpg]

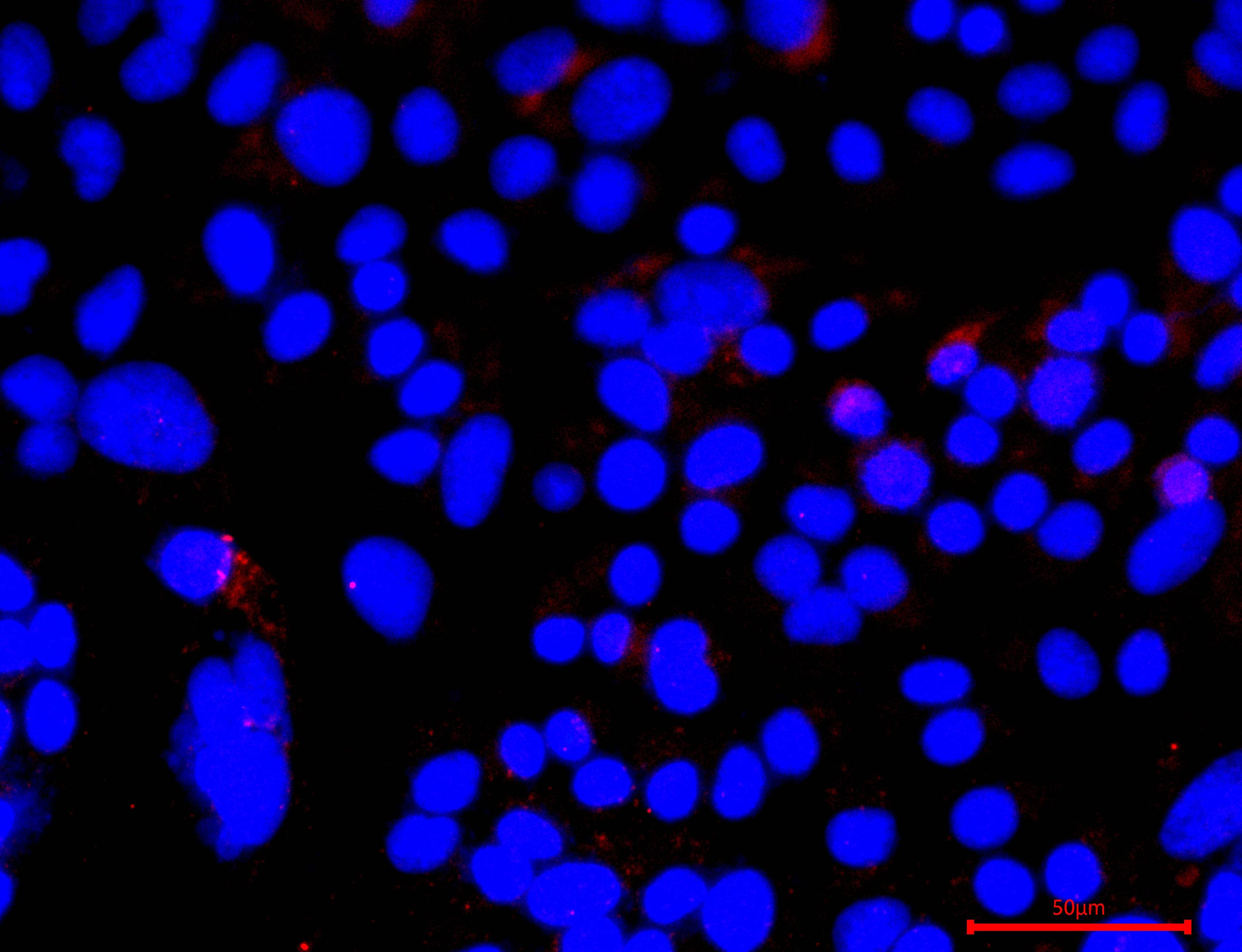

Supplement: Supplementary file 1 [file metabolites-16-00340-s001.zip › Figure S2 Uncropped microscopy images/Figure8/NLRP3/CTLmerge2(1).jpg]

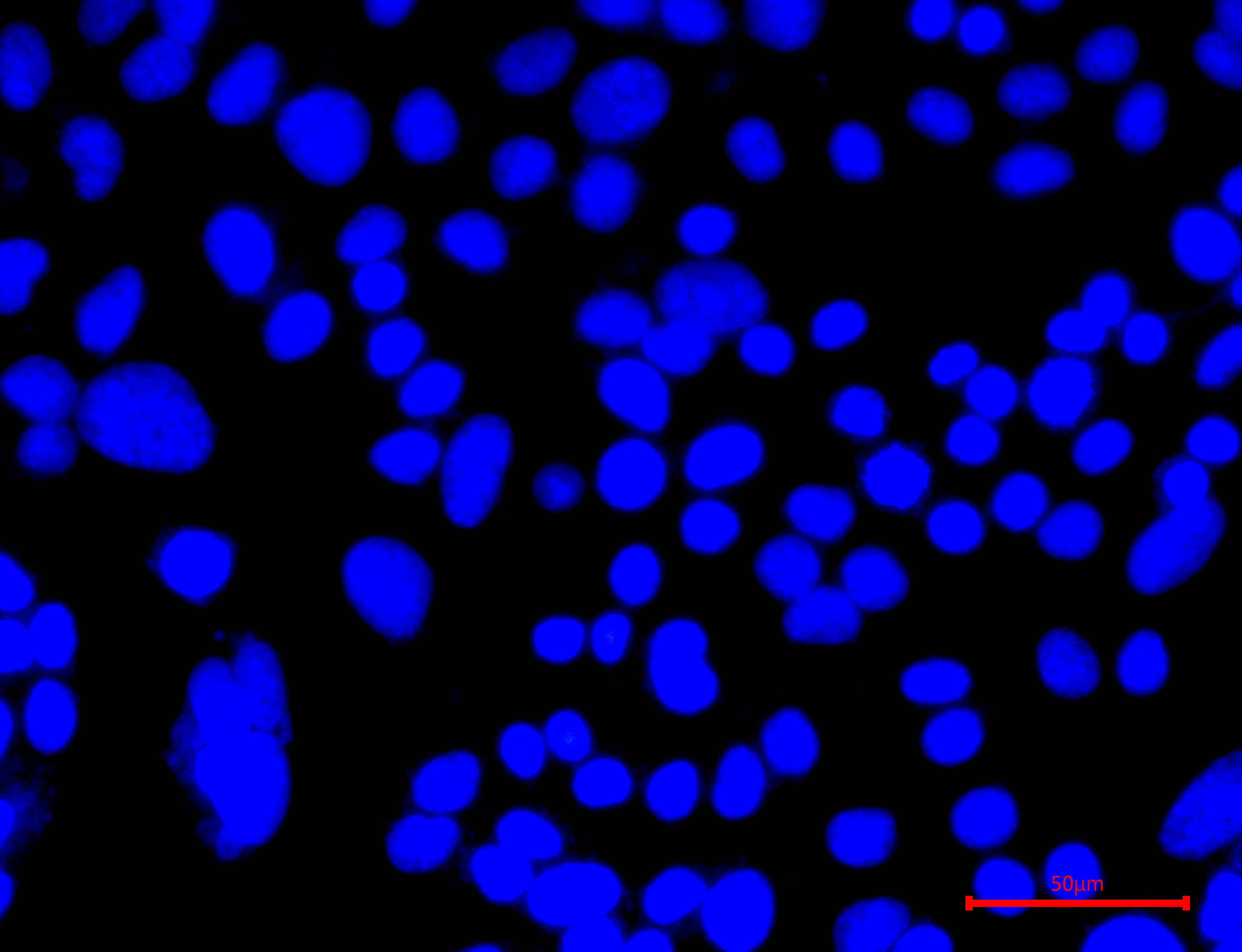

Supplement: Supplementary file 1 [file metabolites-16-00340-s001.zip › Figure S2 Uncropped microscopy images/Figure8/NLRP3/CTL核2(1).jpg]

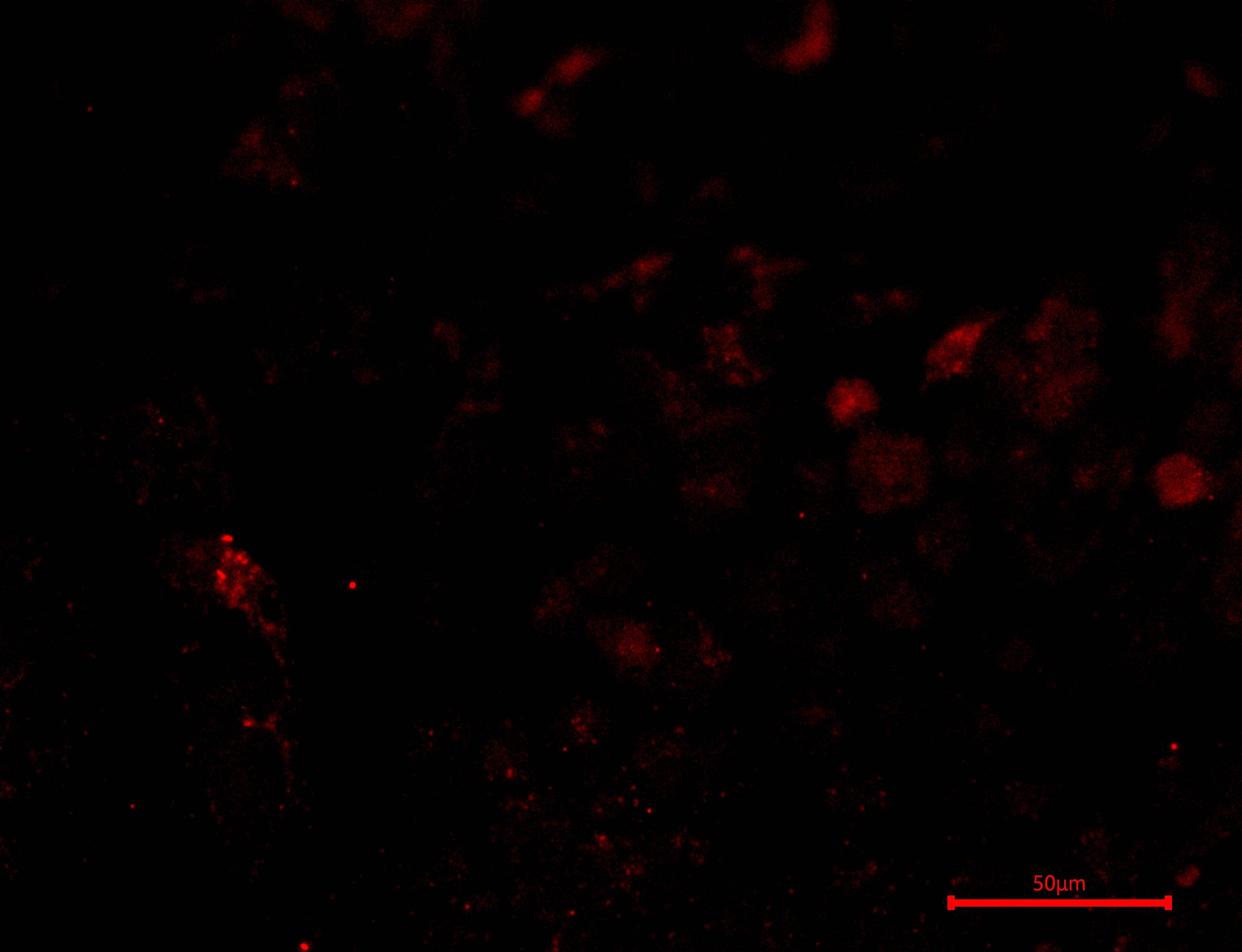

Supplement: Supplementary file 1 [file metabolites-16-00340-s001.zip › Figure S2 Uncropped microscopy images/Figure8/NLRP3/CTL红2(1).jpg]

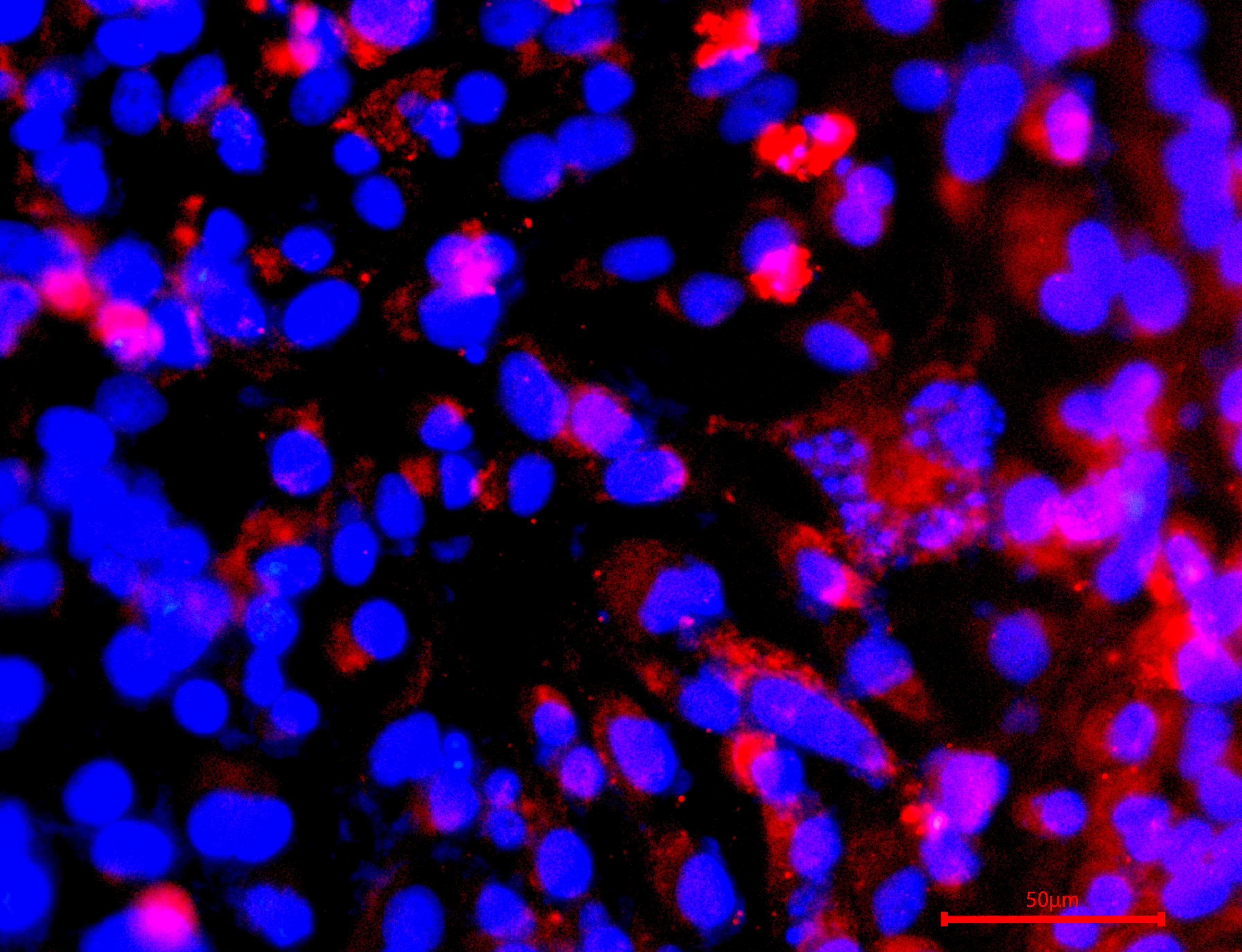

Supplement: Supplementary file 1 [file metabolites-16-00340-s001.zip › Figure S2 Uncropped microscopy images/Figure8/NLRP3/Nmerge1(1).jpg]

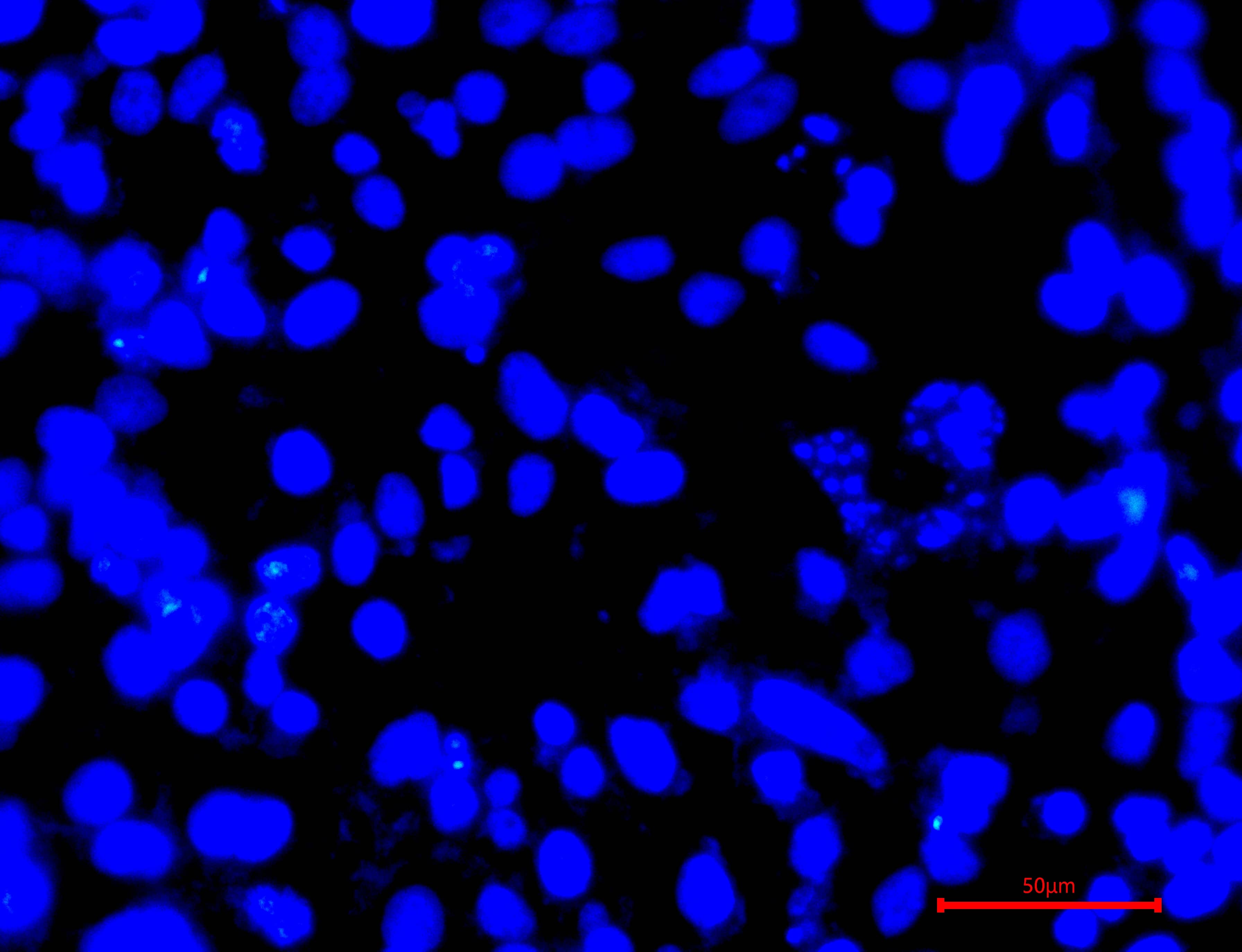

Supplement: Supplementary file 1 [file metabolites-16-00340-s001.zip › Figure S2 Uncropped microscopy images/Figure8/NLRP3/N核1(1).jpg]

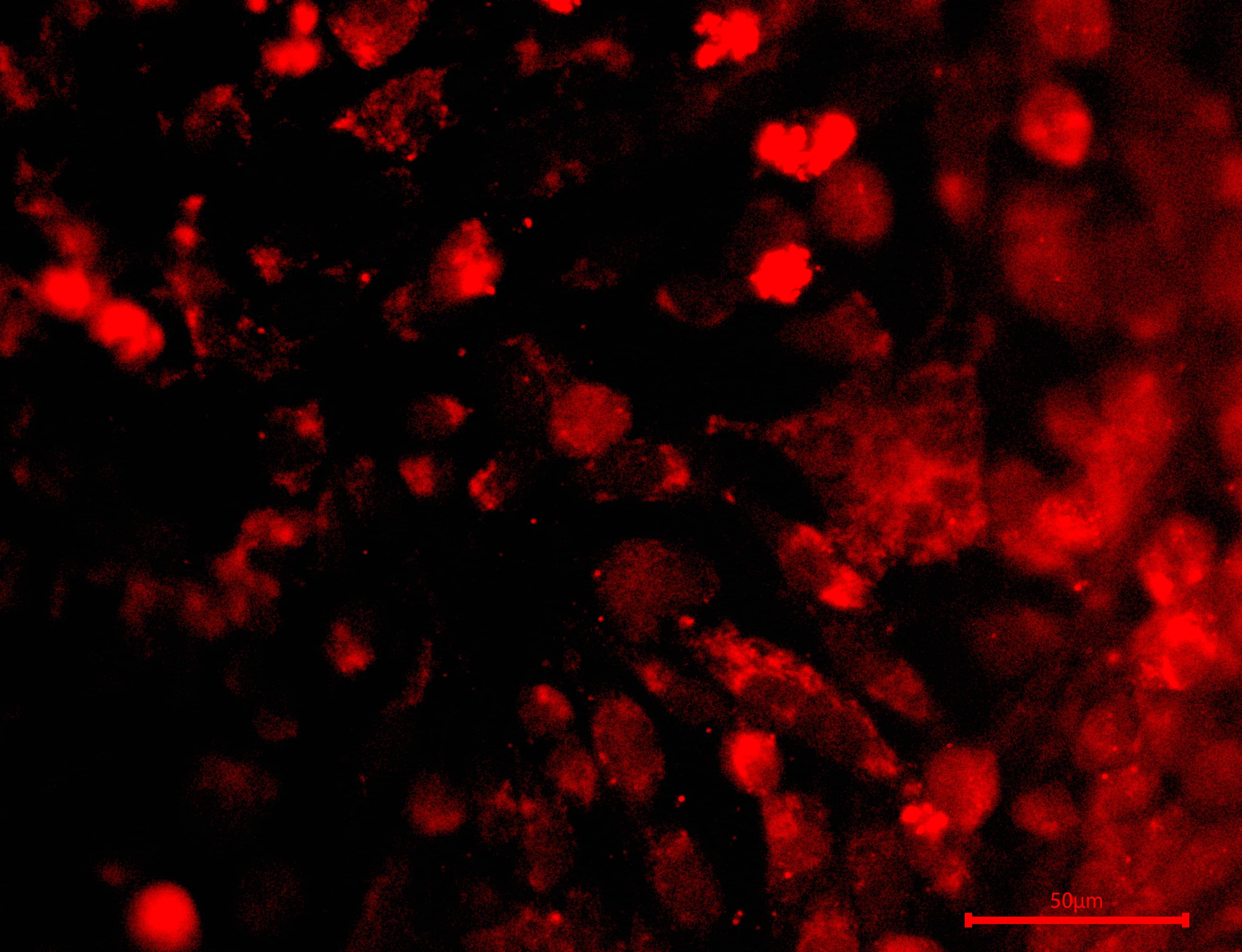

Supplement: Supplementary file 1 [file metabolites-16-00340-s001.zip › Figure S2 Uncropped microscopy images/Figure8/NLRP3/N红1(1).jpg]

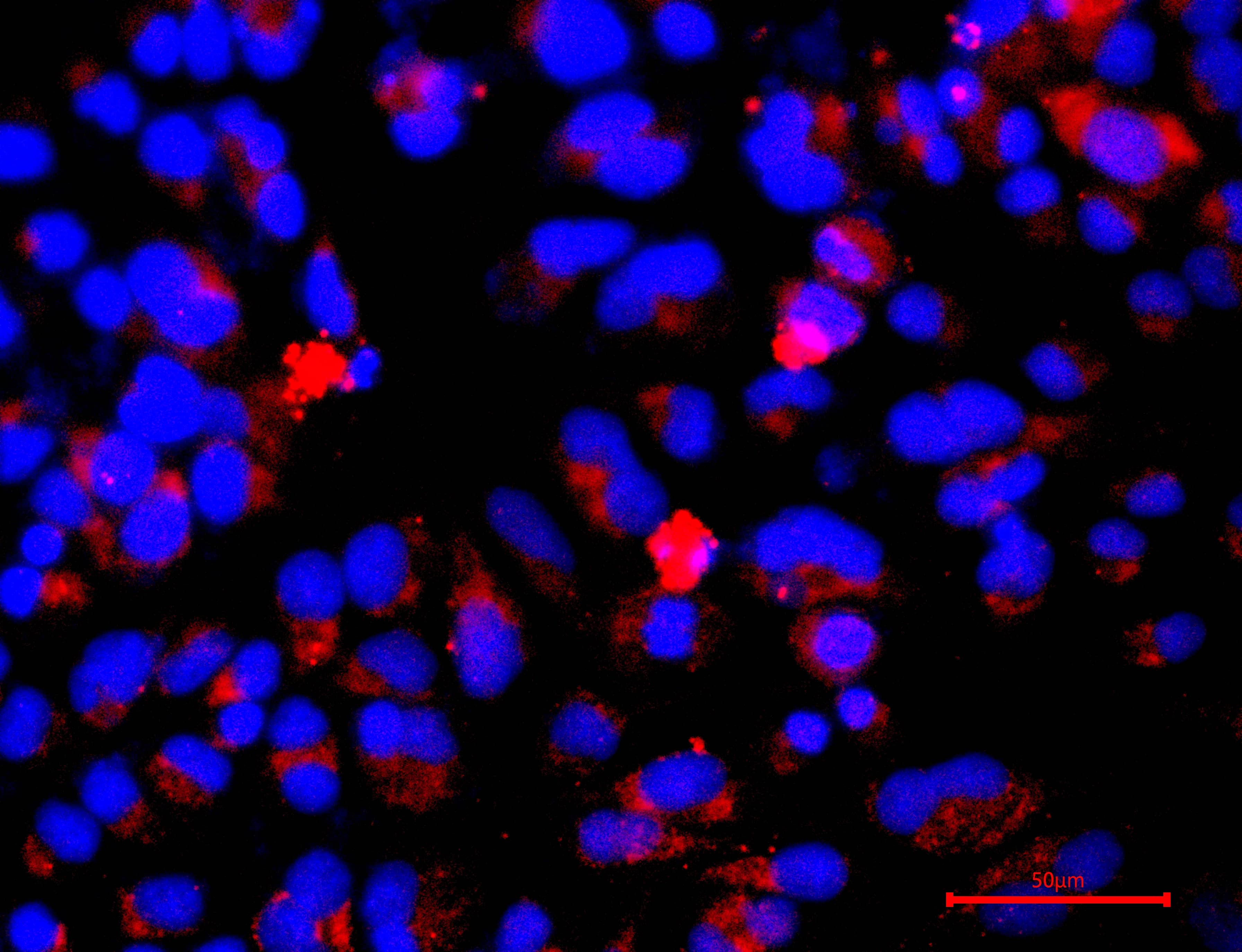

Supplement: Supplementary file 1 [file metabolites-16-00340-s001.zip › Figure S2 Uncropped microscopy images/Figure8/NLRP3/PAmerge2(1).jpg]

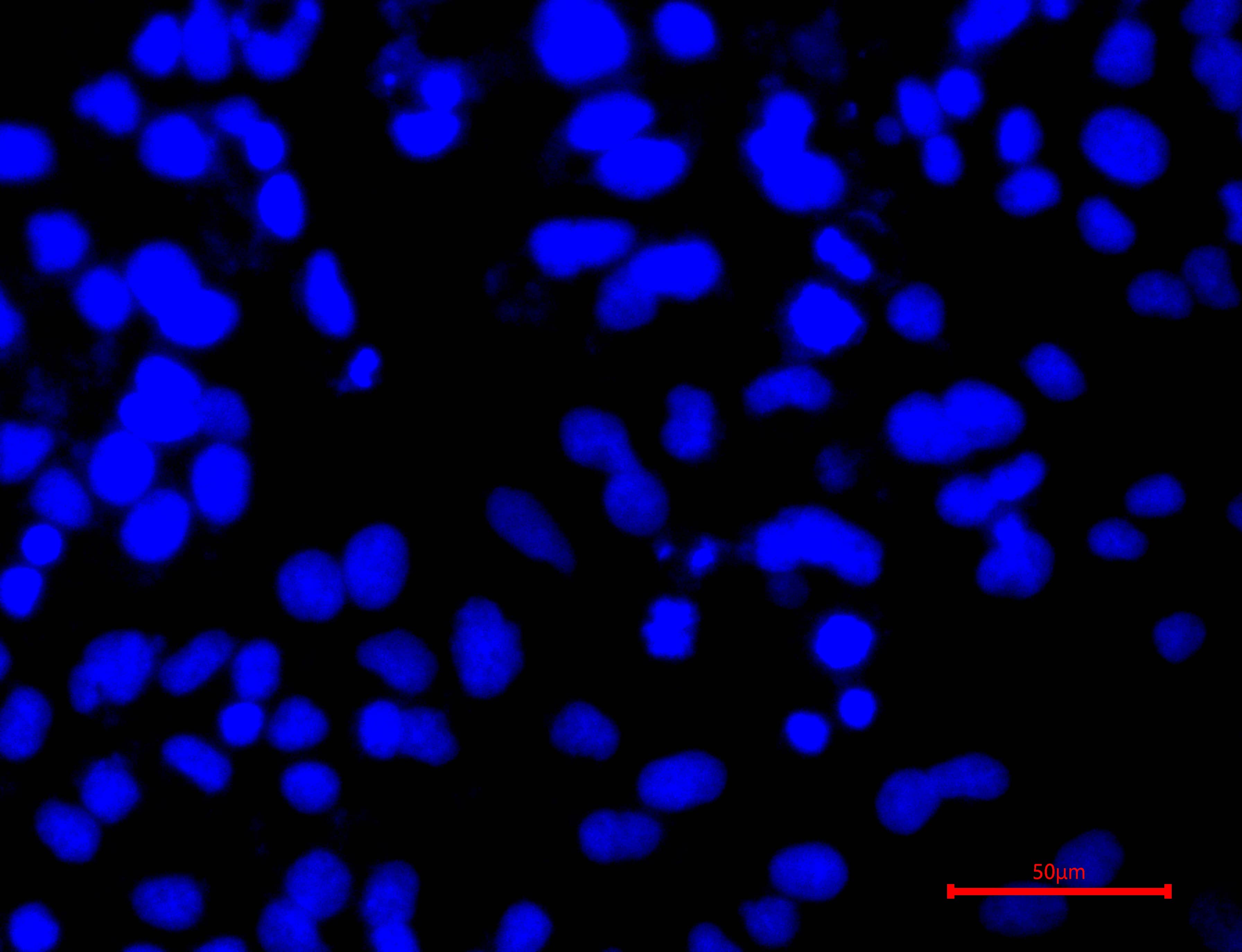

Supplement: Supplementary file 1 [file metabolites-16-00340-s001.zip › Figure S2 Uncropped microscopy images/Figure8/NLRP3/PA核2(1).jpg]

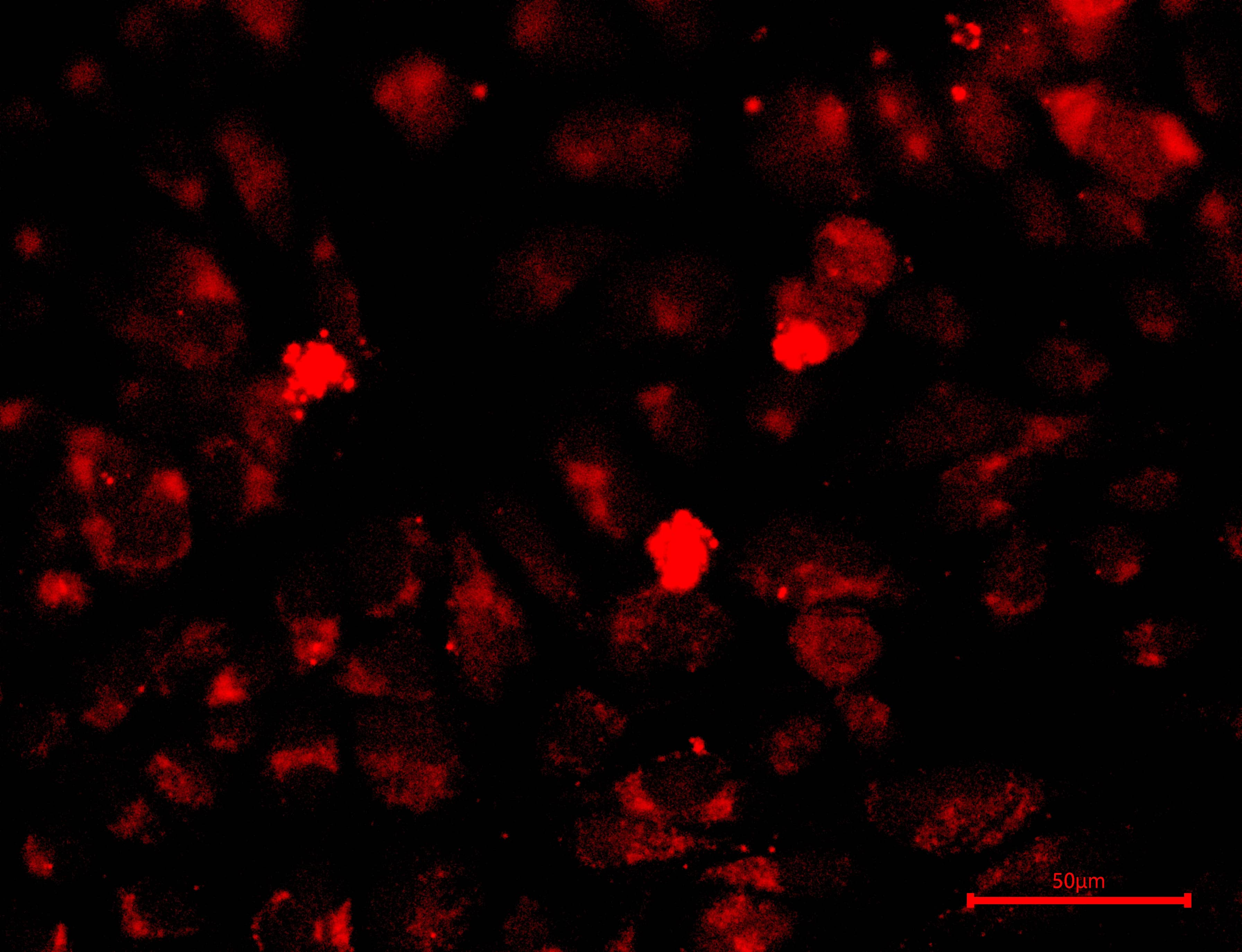

Supplement: Supplementary file 1 [file metabolites-16-00340-s001.zip › Figure S2 Uncropped microscopy images/Figure8/NLRP3/PA红2(1).jpg]

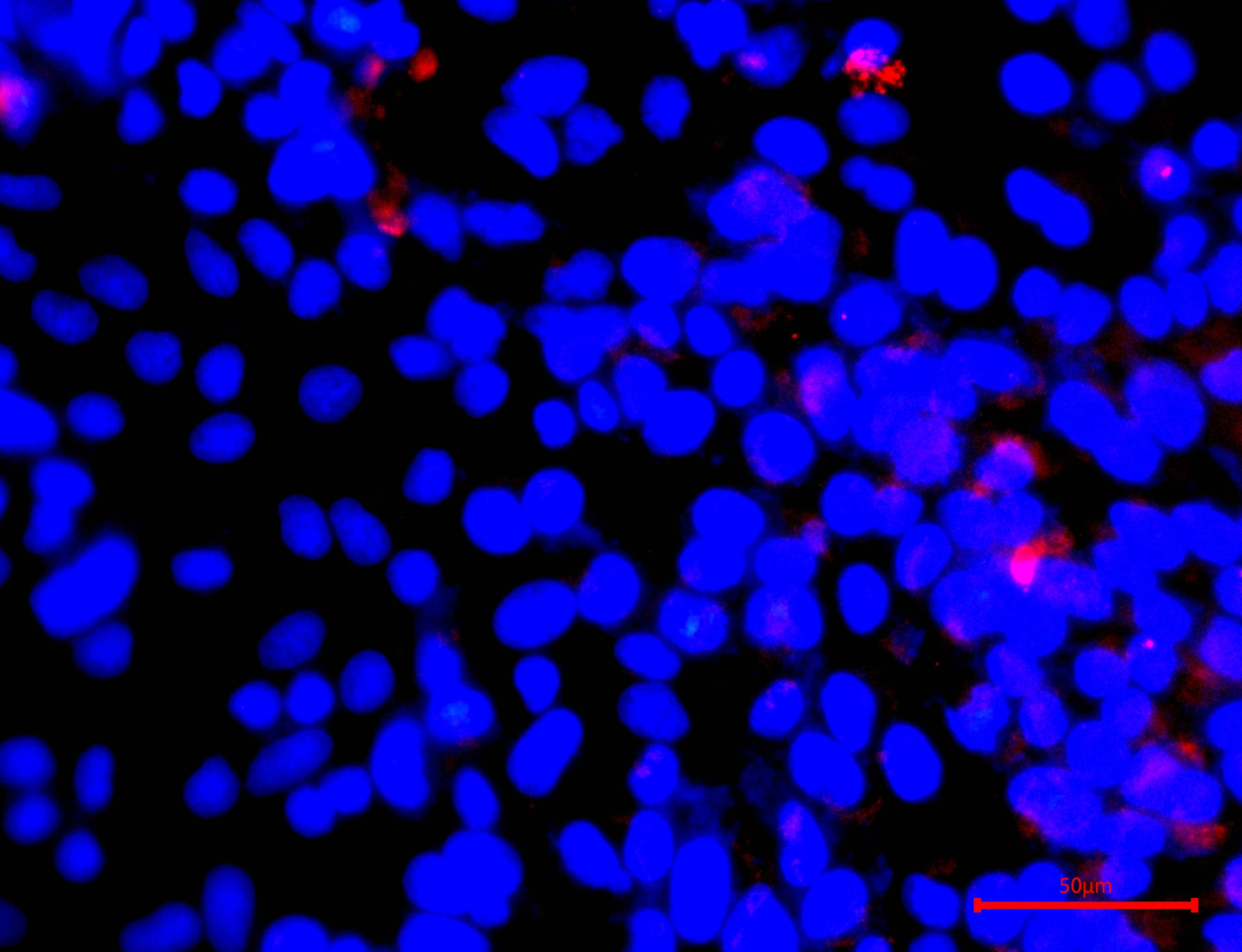

Supplement: Supplementary file 1 [file metabolites-16-00340-s001.zip › Figure S2 Uncropped microscopy images/Figure8/NLRP3/PQQmerge1(1).jpg]

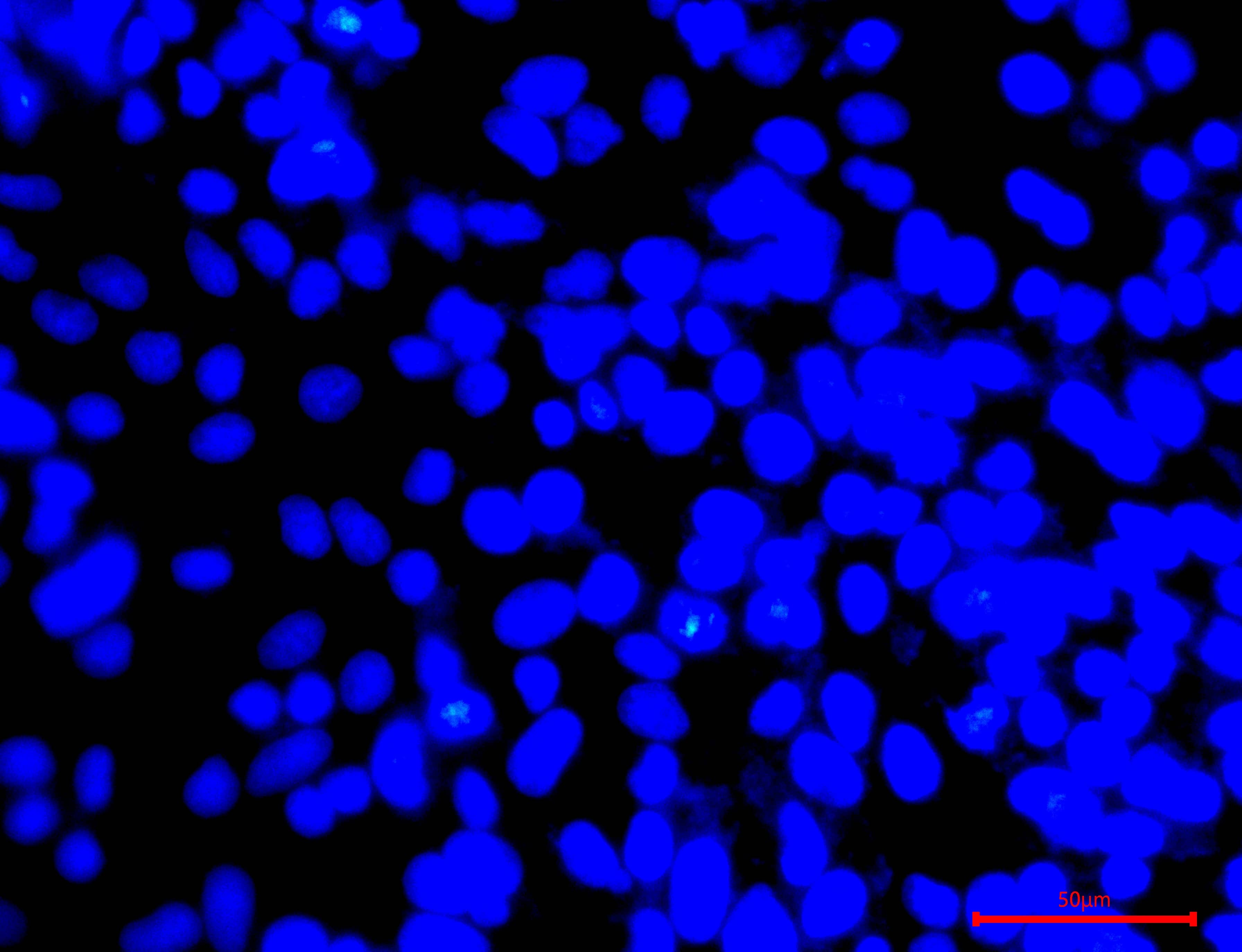

Supplement: Supplementary file 1 [file metabolites-16-00340-s001.zip › Figure S2 Uncropped microscopy images/Figure8/NLRP3/PQQ核1(1).jpg]

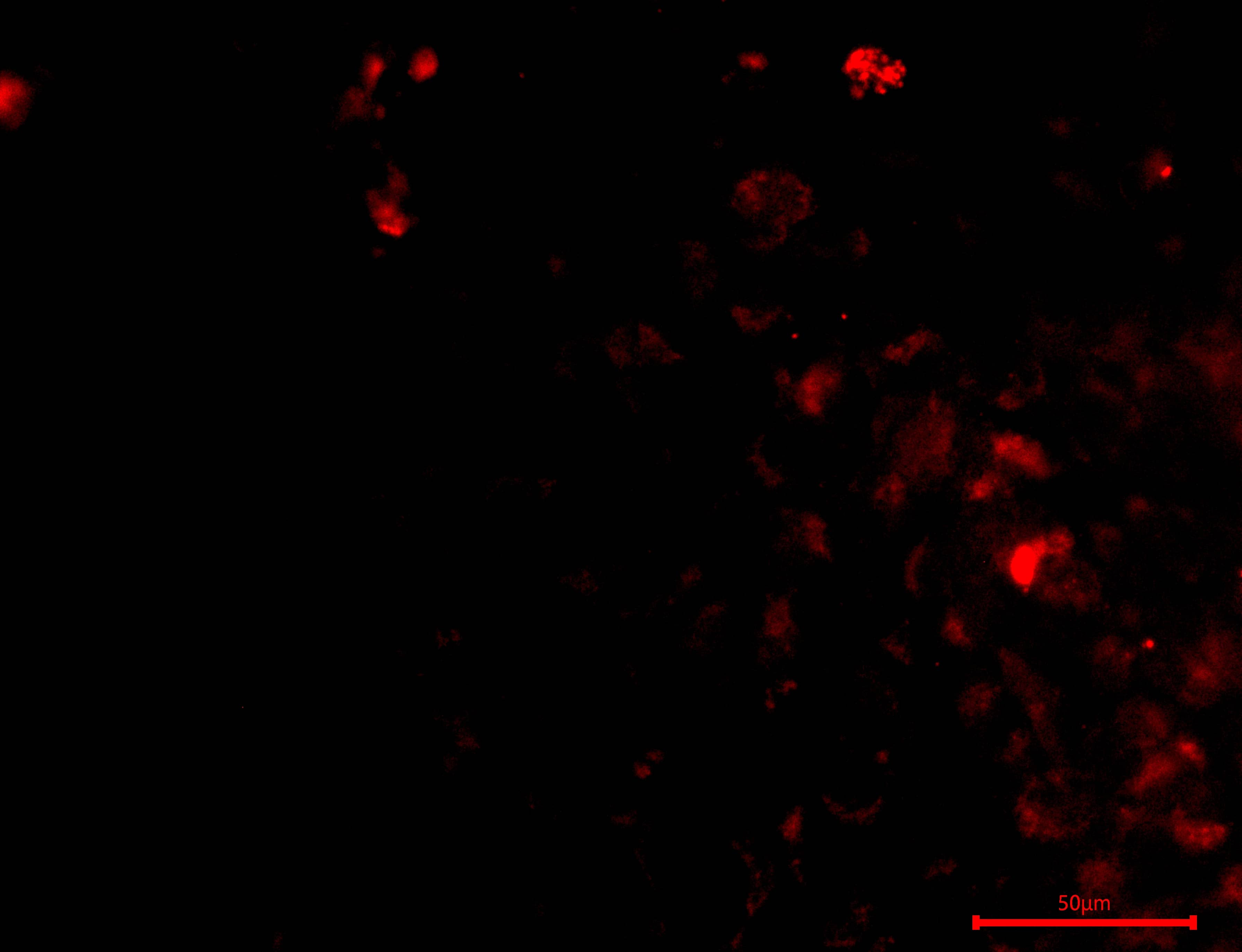

Supplement: Supplementary file 1 [file metabolites-16-00340-s001.zip › Figure S2 Uncropped microscopy images/Figure8/NLRP3/PQQ红1(1).jpg]
