# Supplementary figures and images for: Pyrroloquinoline Quinone Mitigates Type 2 Diabetes-Induced Cardiac Injury Through Mitochondrial Quality Control and Inhibition of NLRP3-Dependent Pyroptosis (part 1 of 2)
Source: Metabolites. 2026 May 19;16(5):340. doi: 10.3390/metabo16050340 (PMC13209680; doi:10.3390/metabo16050340)

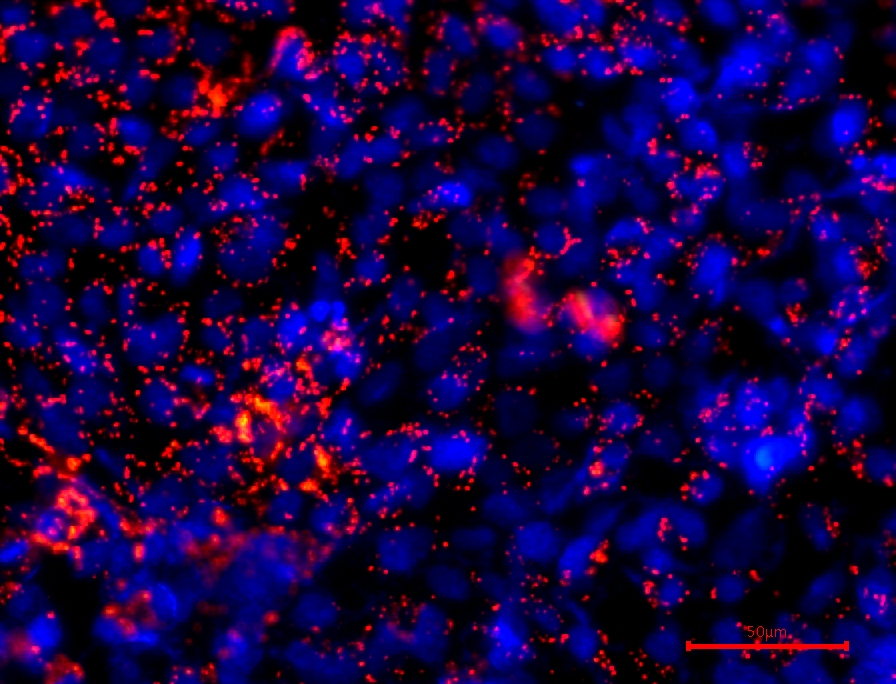

Supplement: Supplementary file 1 [file metabolites-16-00340-s001.zip › Figure S2 Uncropped microscopy images/Figure6/JC-1/CTL/CTL merge5.jpg]

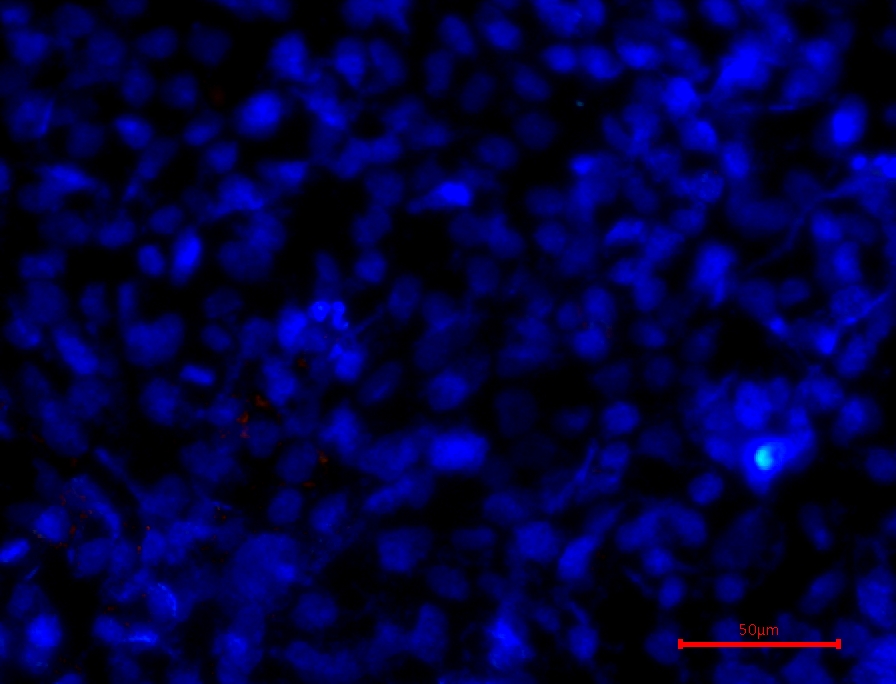

Supplement: Supplementary file 1 [file metabolites-16-00340-s001.zip › Figure S2 Uncropped microscopy images/Figure6/JC-1/CTL/CTL核5.jpg]

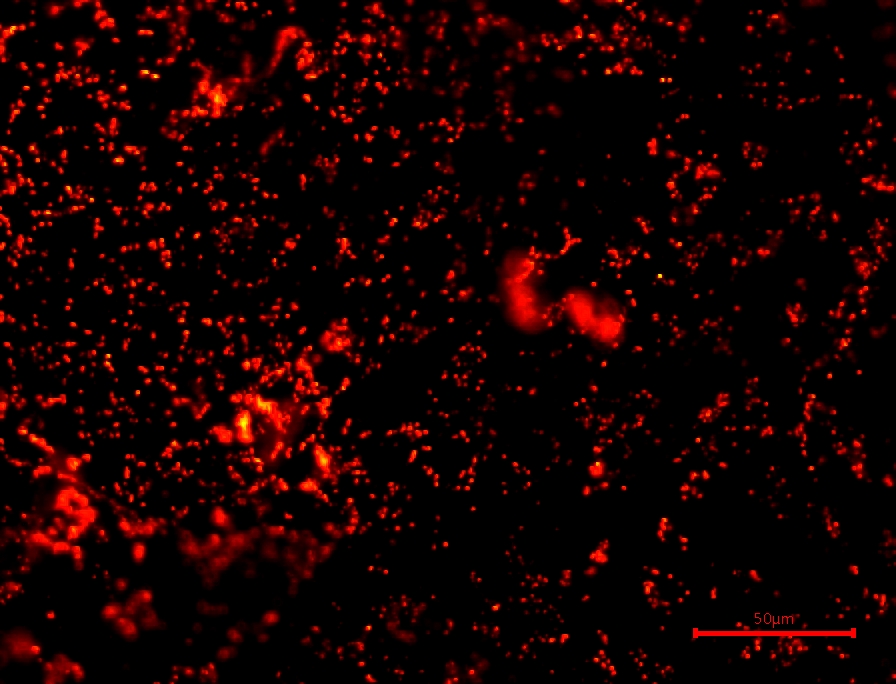

Supplement: Supplementary file 1 [file metabolites-16-00340-s001.zip › Figure S2 Uncropped microscopy images/Figure6/JC-1/CTL/CTL红5.jpg]

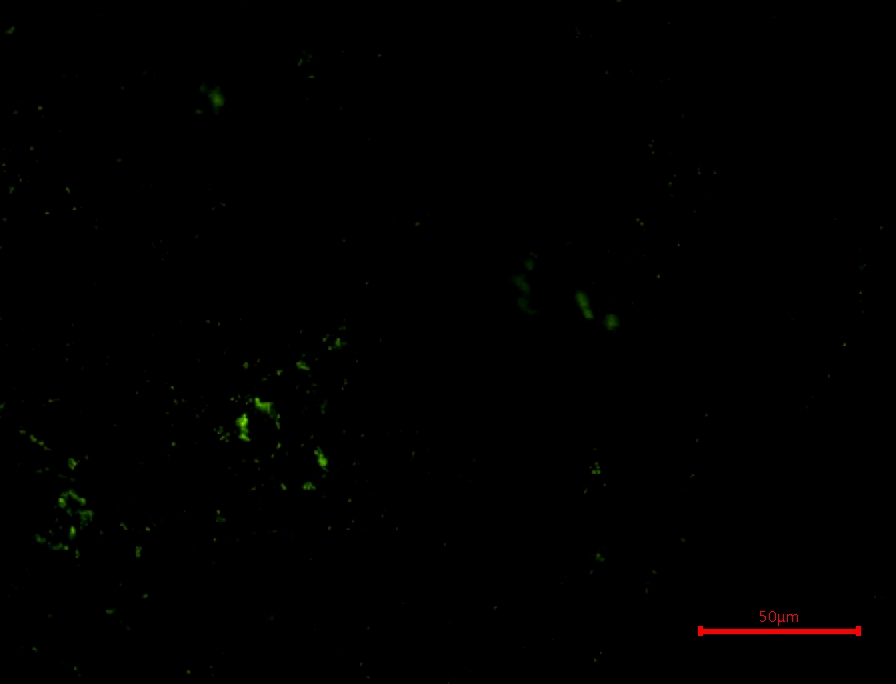

Supplement: Supplementary file 1 [file metabolites-16-00340-s001.zip › Figure S2 Uncropped microscopy images/Figure6/JC-1/CTL/CTL绿5.jpg]

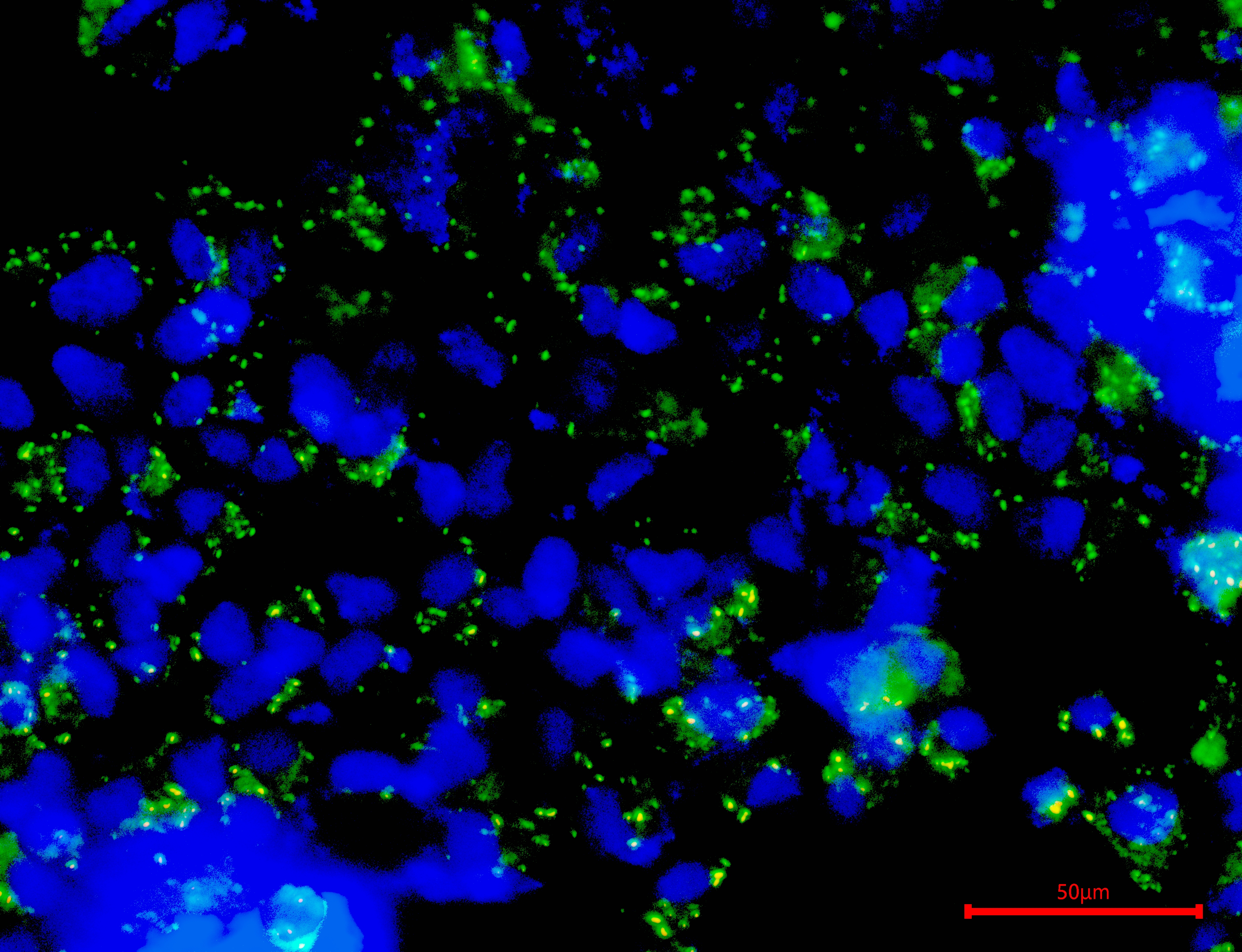

Supplement: Supplementary file 1 [file metabolites-16-00340-s001.zip › Figure S2 Uncropped microscopy images/Figure6/JC-1/PA/PA merge 1.jpg]

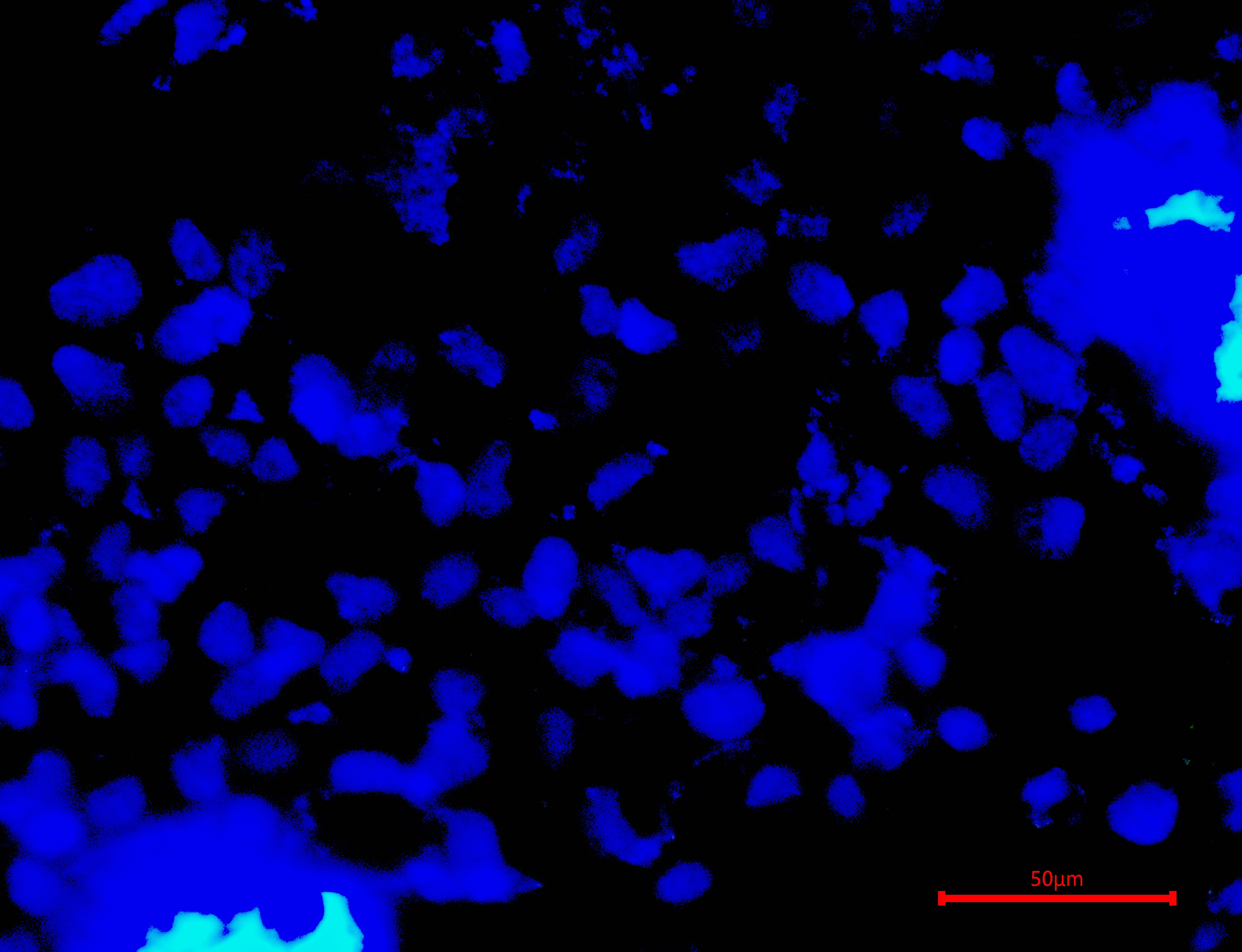

Supplement: Supplementary file 1 [file metabolites-16-00340-s001.zip › Figure S2 Uncropped microscopy images/Figure6/JC-1/PA/PA核1.jpg]

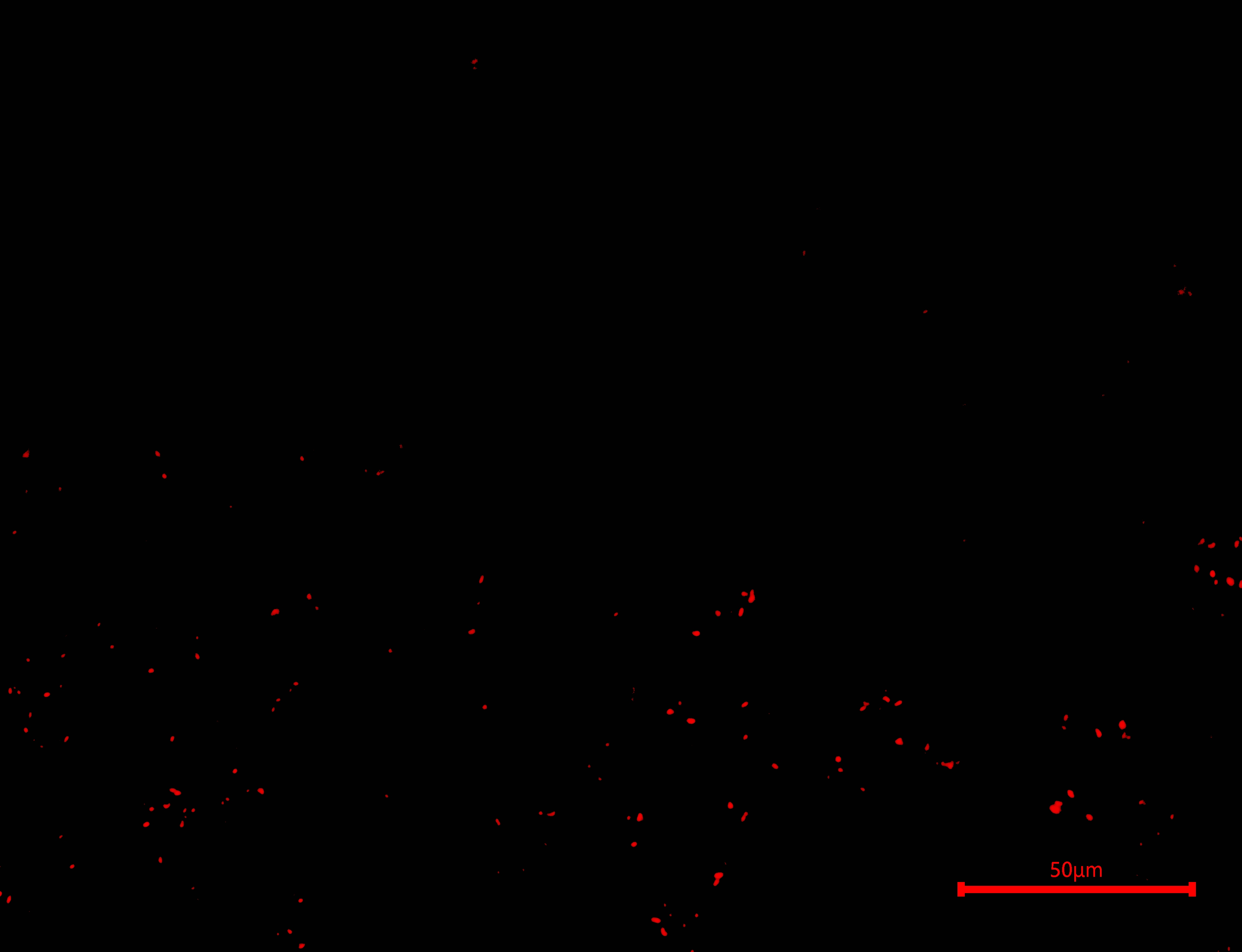

Supplement: Supplementary file 1 [file metabolites-16-00340-s001.zip › Figure S2 Uncropped microscopy images/Figure6/JC-1/PA/PA红1.jpg]

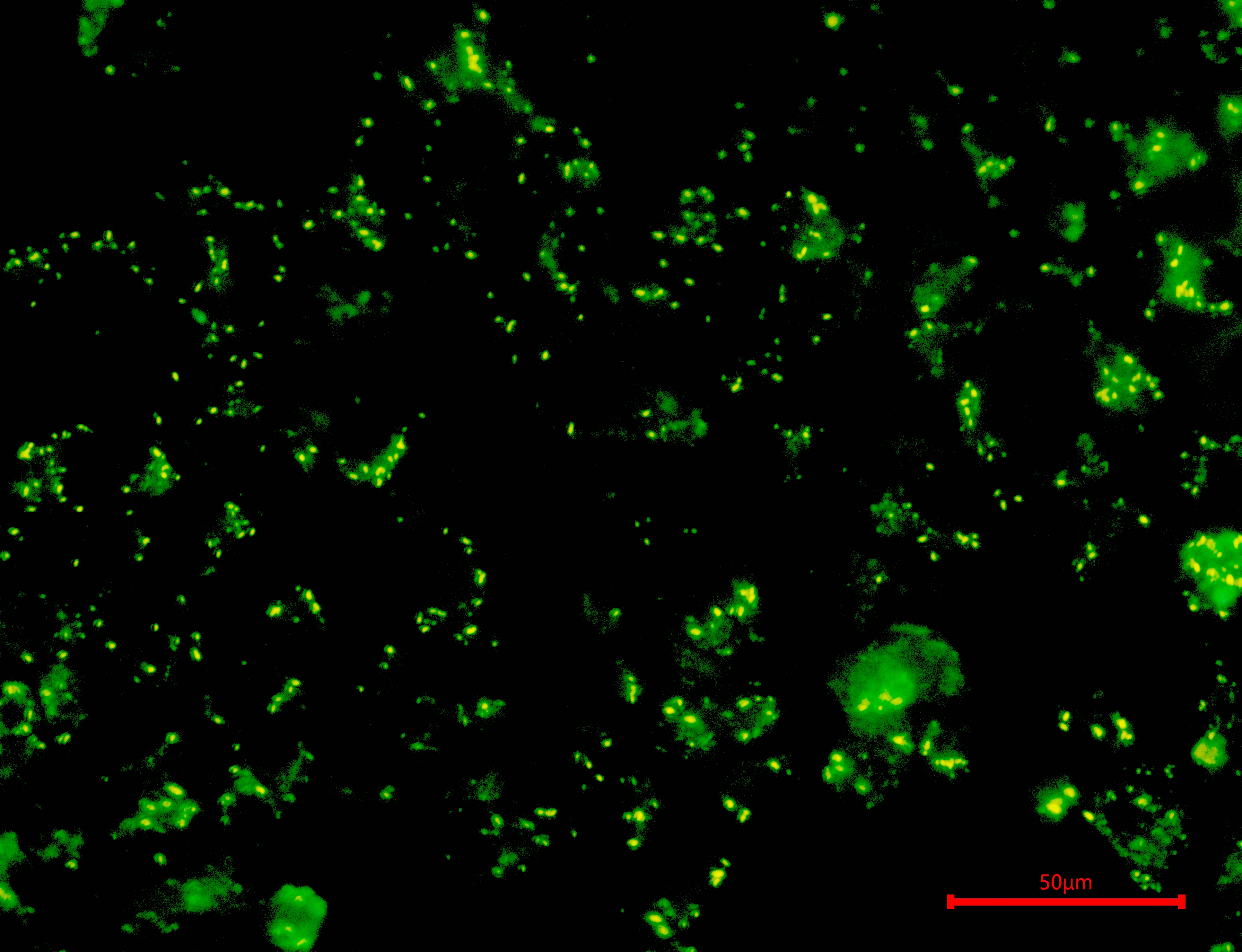

Supplement: Supplementary file 1 [file metabolites-16-00340-s001.zip › Figure S2 Uncropped microscopy images/Figure6/JC-1/PA/PA绿1.jpg]

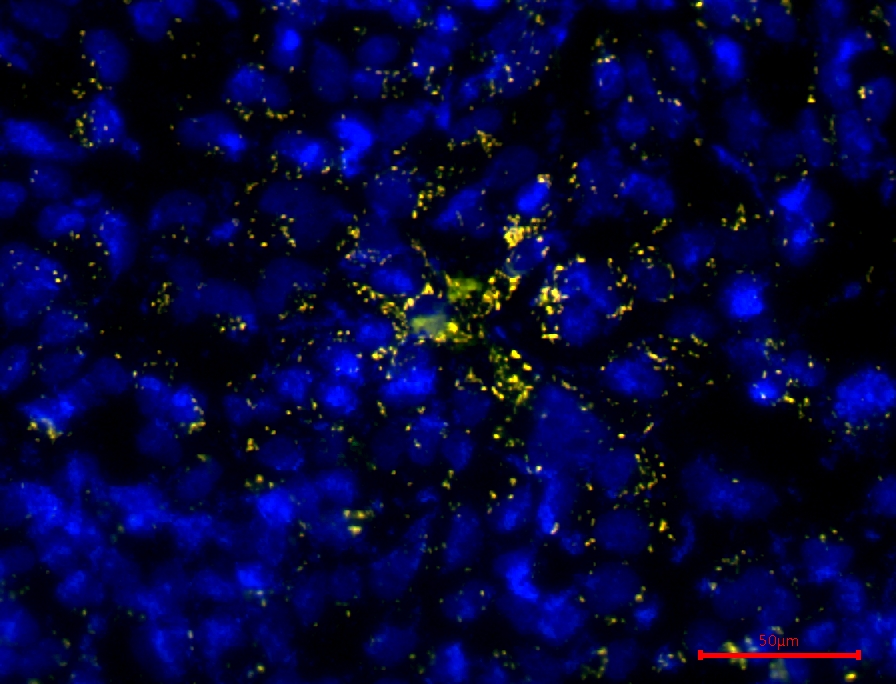

Supplement: Supplementary file 1 [file metabolites-16-00340-s001.zip › Figure S2 Uncropped microscopy images/Figure6/JC-1/PQQ/PQQ merge3.jpg]

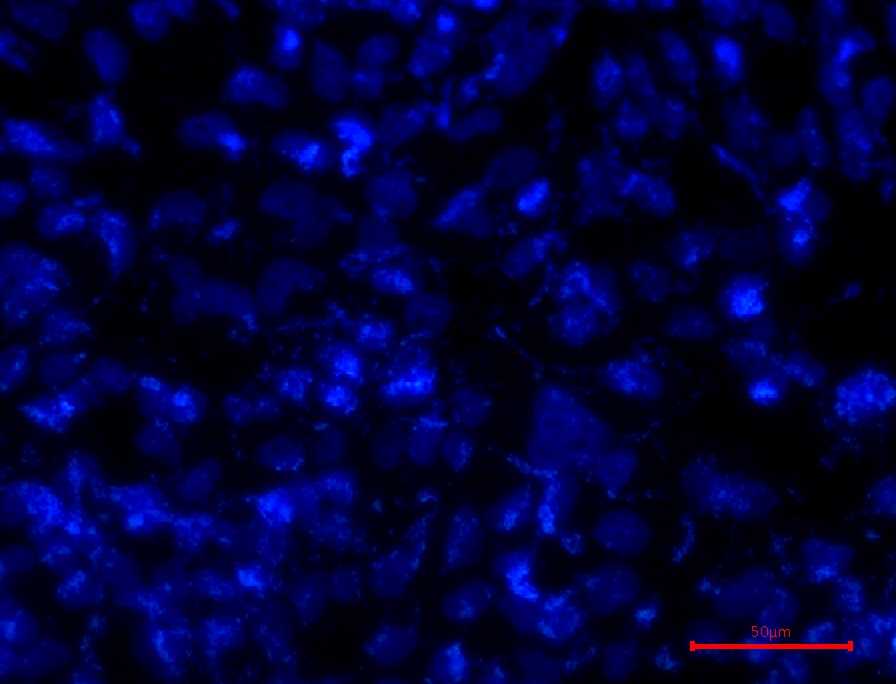

Supplement: Supplementary file 1 [file metabolites-16-00340-s001.zip › Figure S2 Uncropped microscopy images/Figure6/JC-1/PQQ/PQQ核3.jpg]

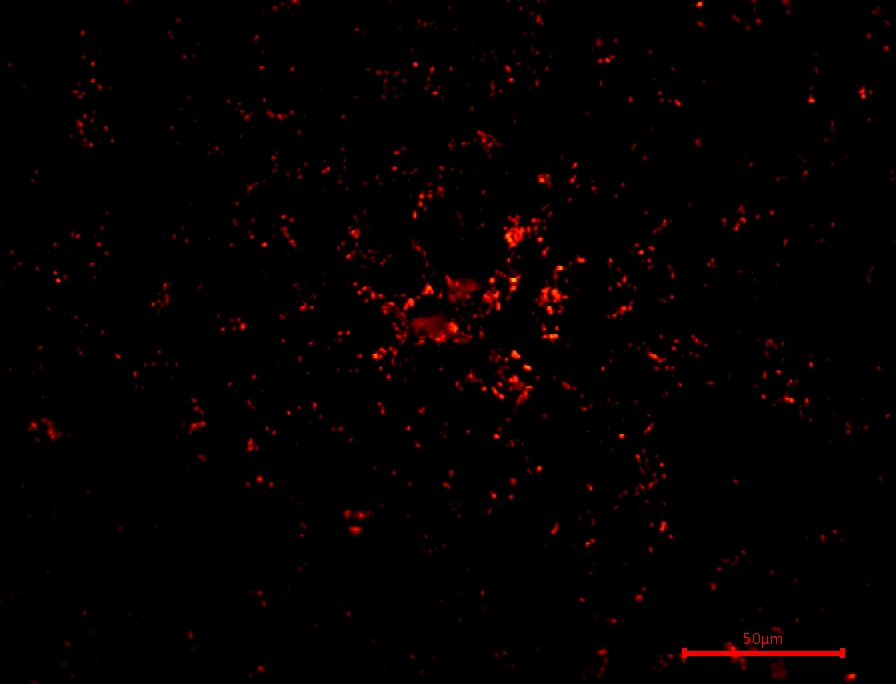

Supplement: Supplementary file 1 [file metabolites-16-00340-s001.zip › Figure S2 Uncropped microscopy images/Figure6/JC-1/PQQ/PQQ红3.jpg]

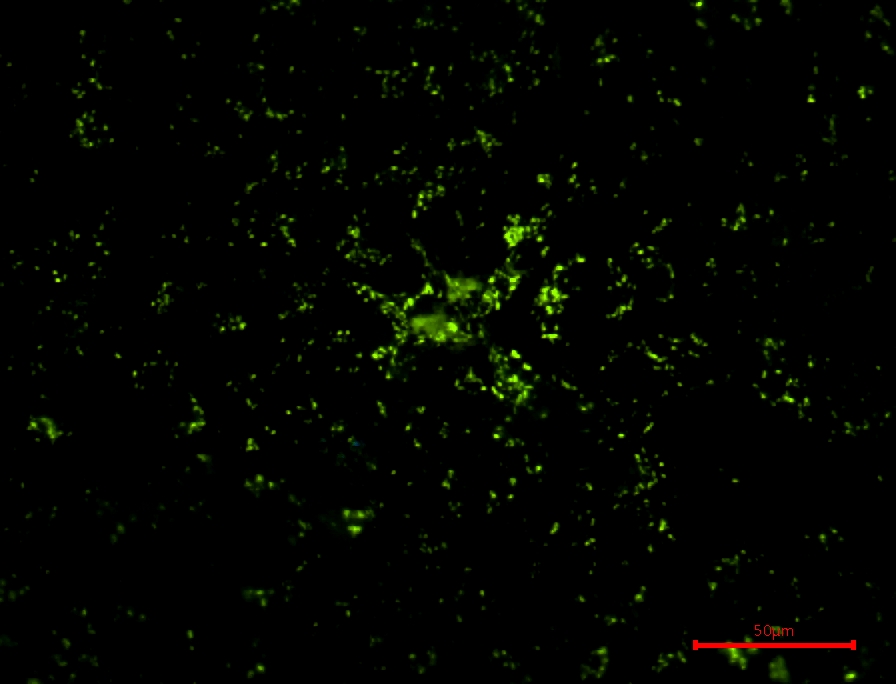

Supplement: Supplementary file 1 [file metabolites-16-00340-s001.zip › Figure S2 Uncropped microscopy images/Figure6/JC-1/PQQ/PQQ绿3.jpg]

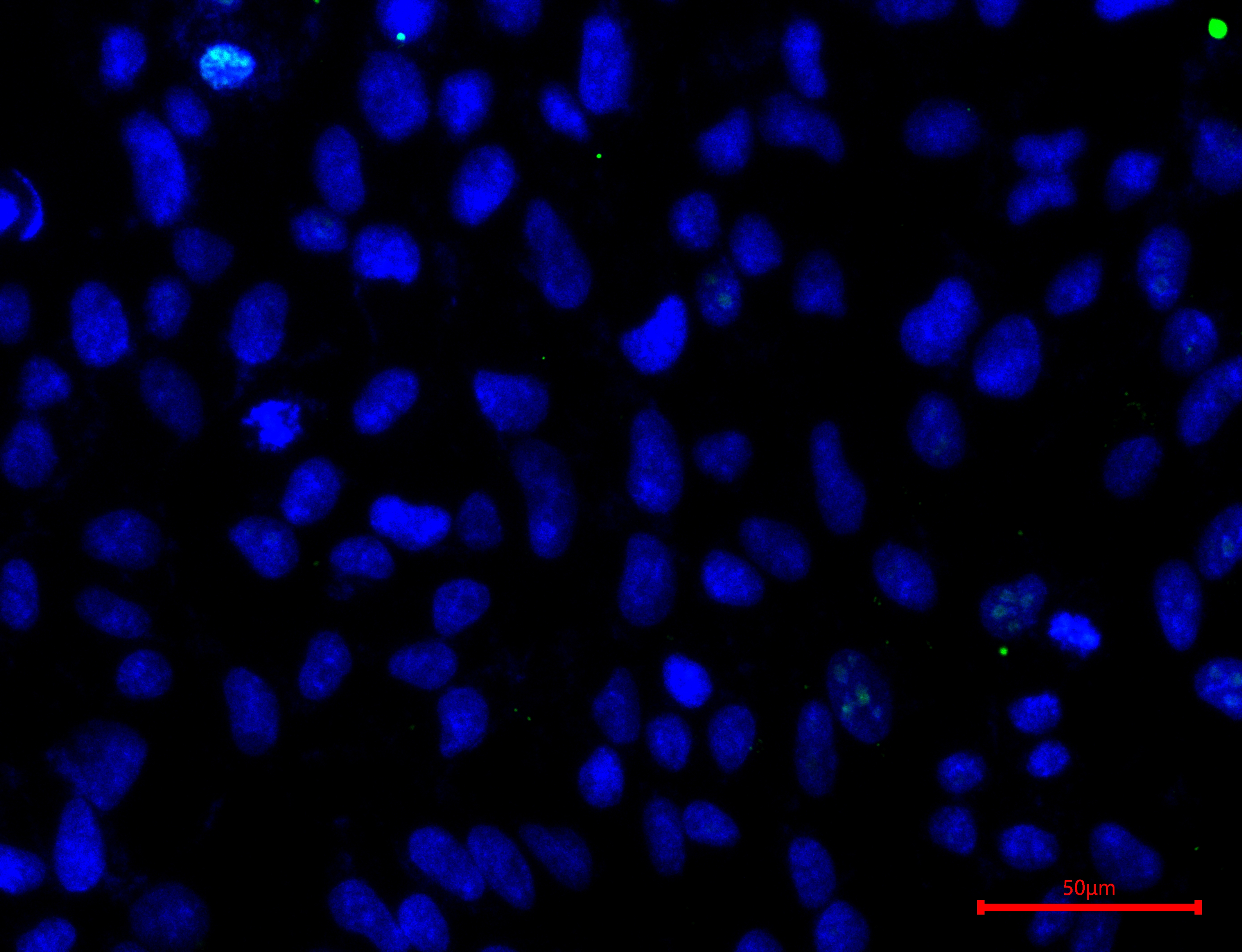

Supplement: Supplementary file 1 [file metabolites-16-00340-s001.zip › Figure S2 Uncropped microscopy images/Figure6/pyroptosis/CTL/CTLmerge2.jpg]

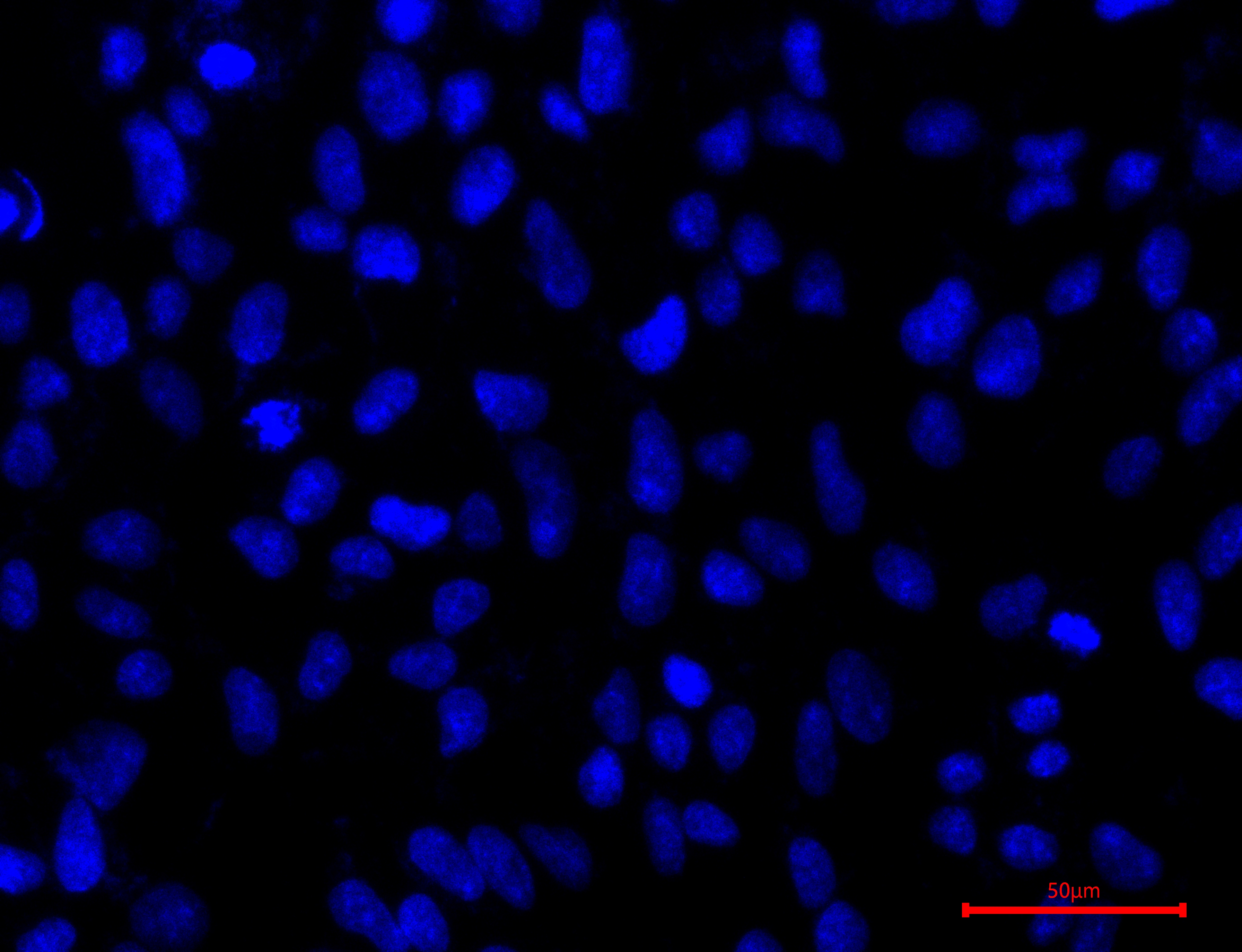

Supplement: Supplementary file 1 [file metabolites-16-00340-s001.zip › Figure S2 Uncropped microscopy images/Figure6/pyroptosis/CTL/CTL核2.jpg]

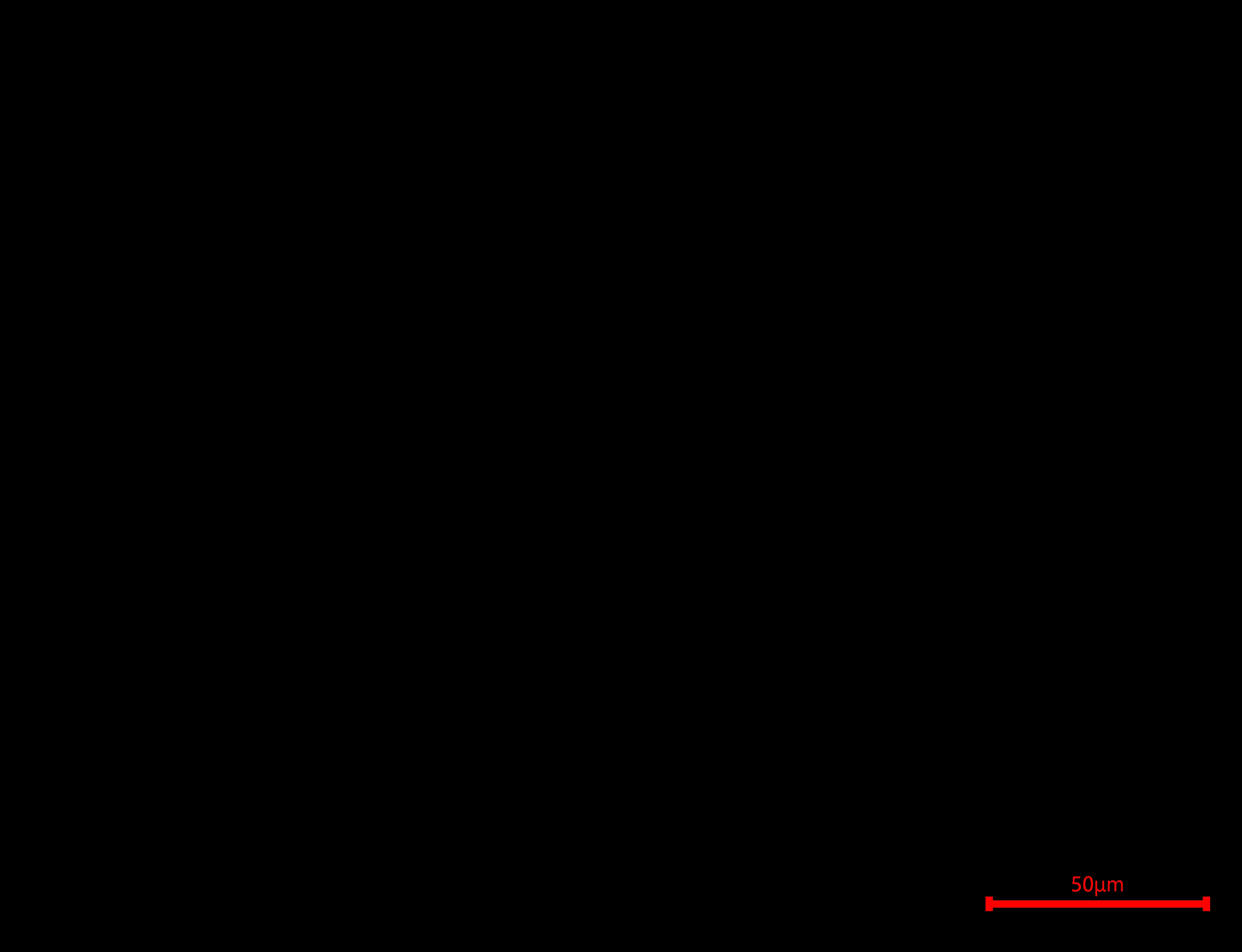

Supplement: Supplementary file 1 [file metabolites-16-00340-s001.zip › Figure S2 Uncropped microscopy images/Figure6/pyroptosis/CTL/CTL红2.jpg]

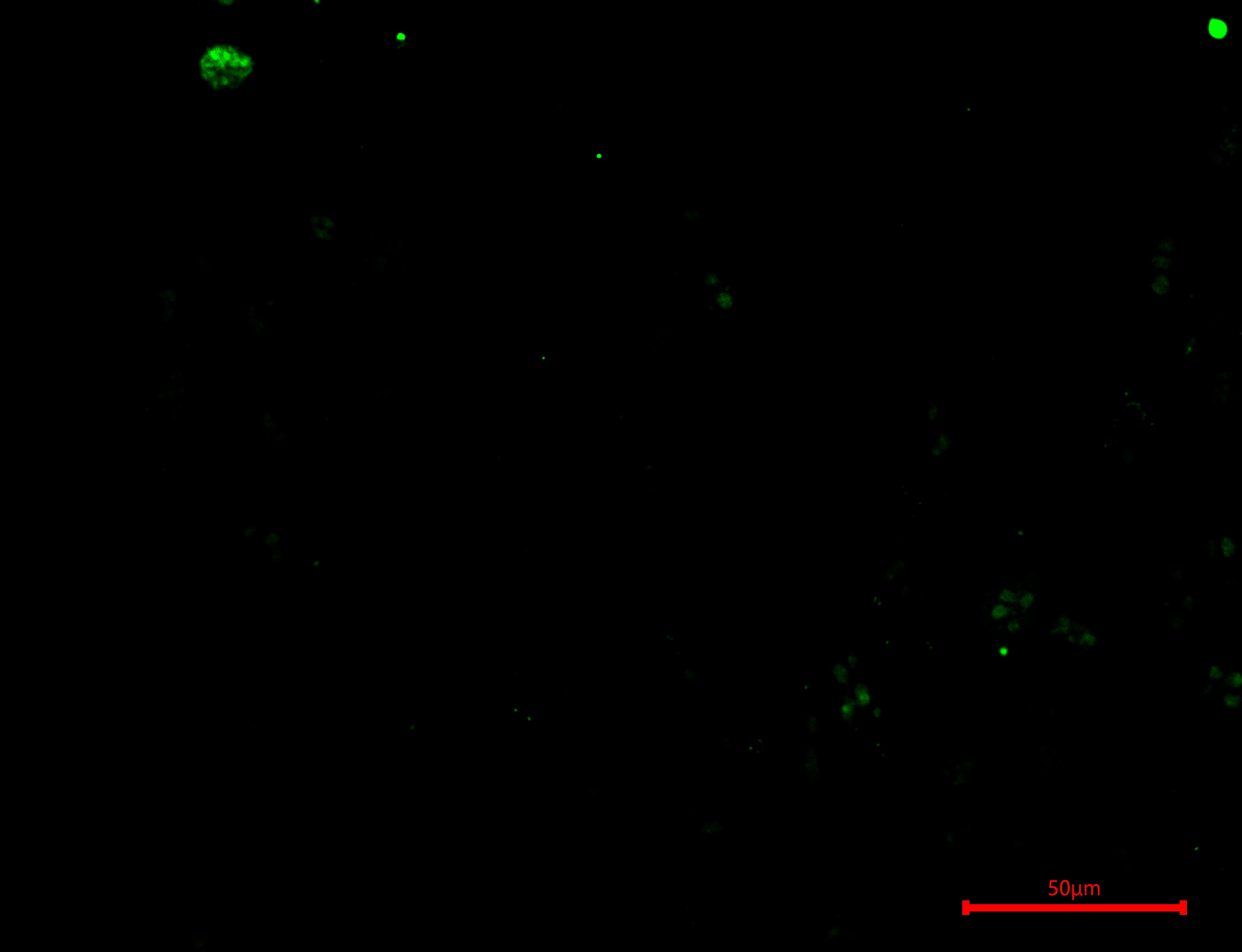

Supplement: Supplementary file 1 [file metabolites-16-00340-s001.zip › Figure S2 Uncropped microscopy images/Figure6/pyroptosis/CTL/CTL绿2.jpg]

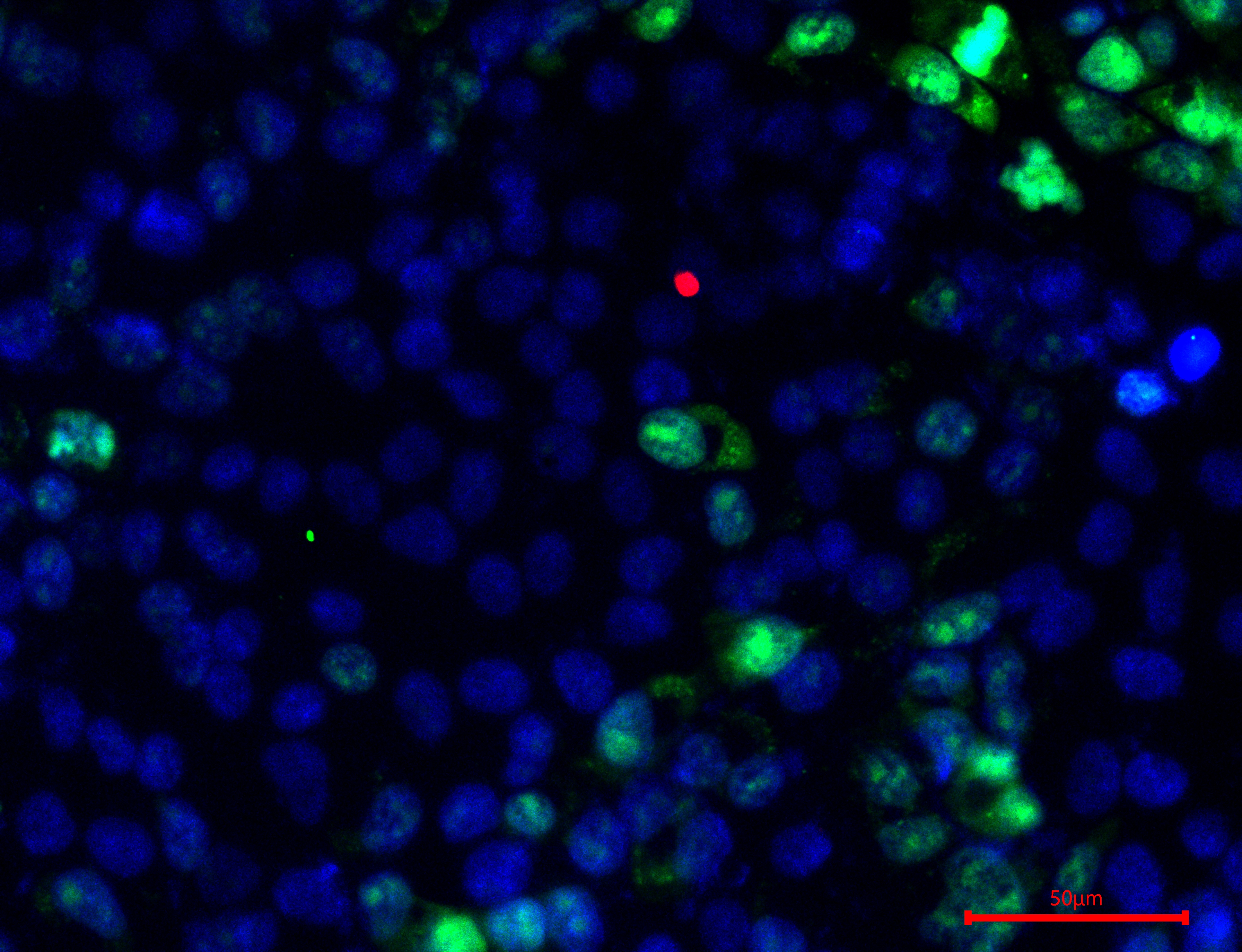

Supplement: Supplementary file 1 [file metabolites-16-00340-s001.zip › Figure S2 Uncropped microscopy images/Figure6/pyroptosis/nigericin/Nmerge1.jpg]

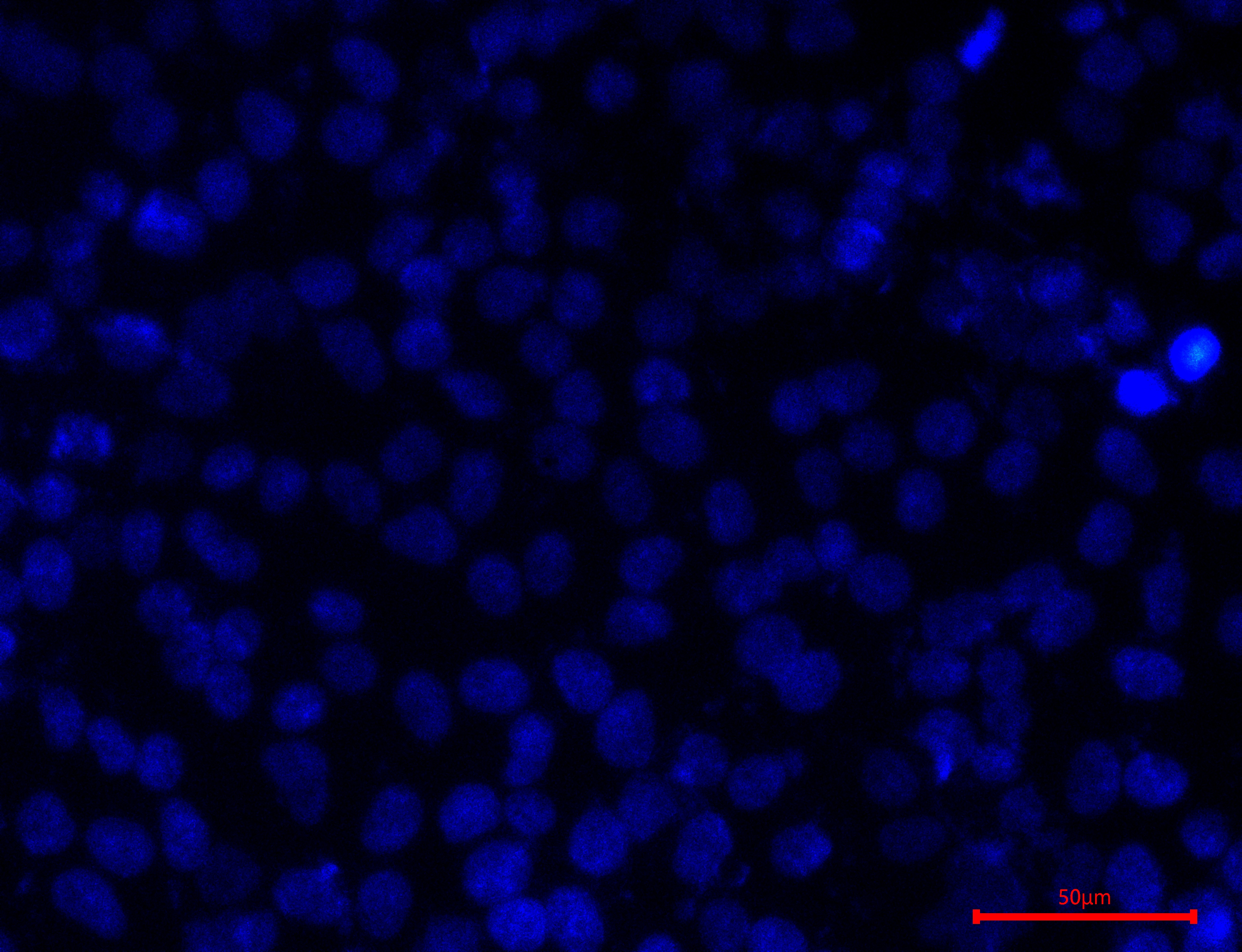

Supplement: Supplementary file 1 [file metabolites-16-00340-s001.zip › Figure S2 Uncropped microscopy images/Figure6/pyroptosis/nigericin/N核1.jpg]

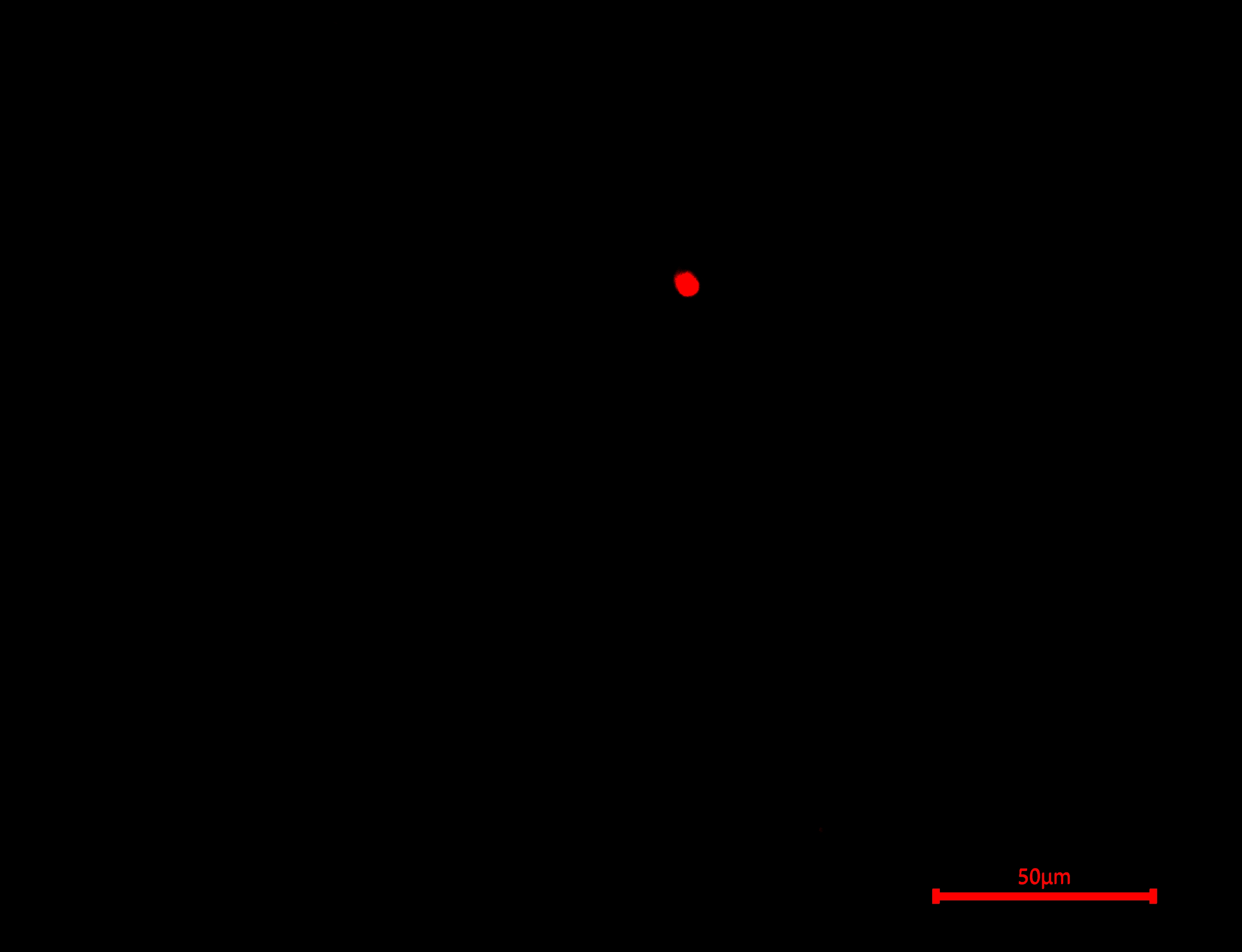

Supplement: Supplementary file 1 [file metabolites-16-00340-s001.zip › Figure S2 Uncropped microscopy images/Figure6/pyroptosis/nigericin/N红1.jpg]

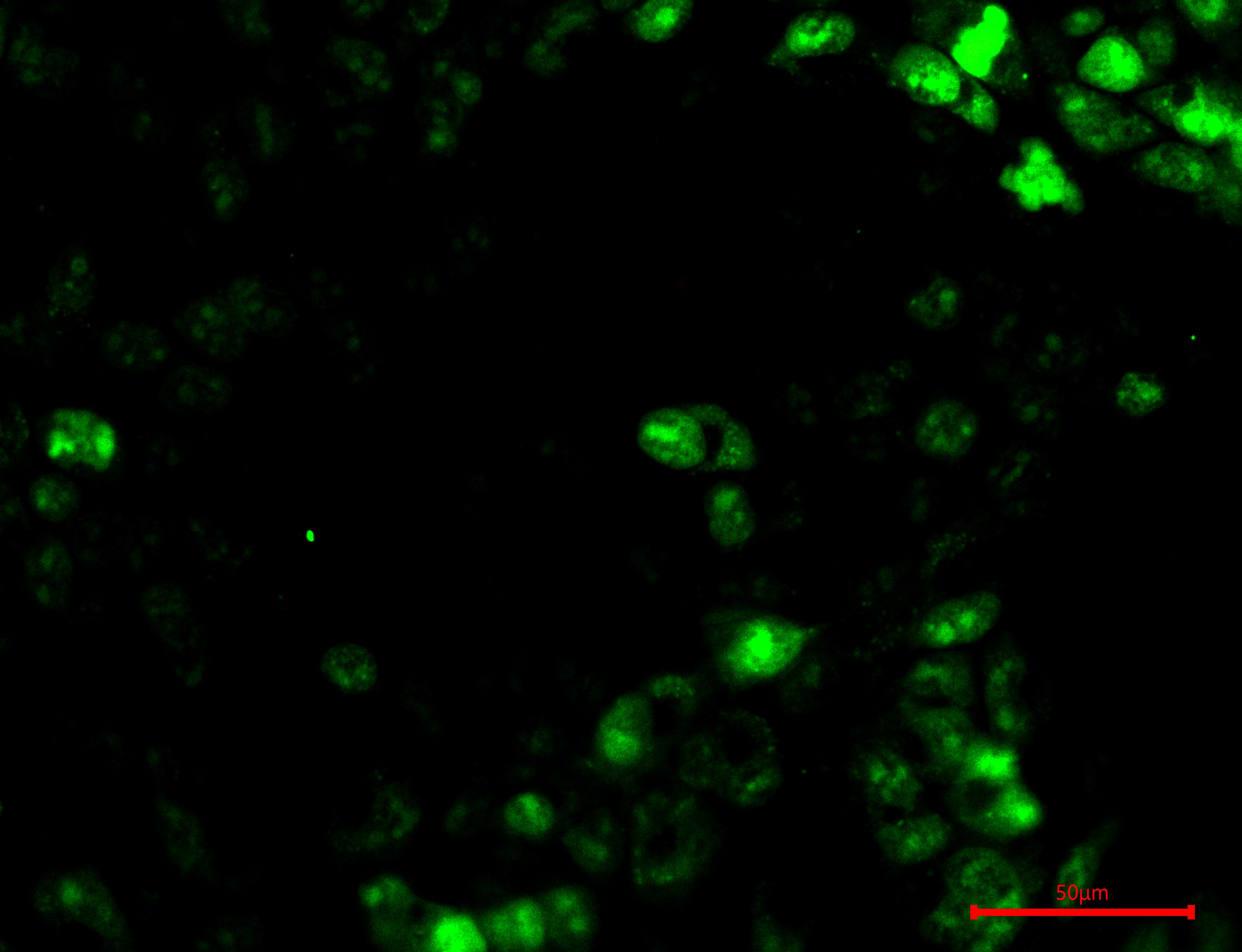

Supplement: Supplementary file 1 [file metabolites-16-00340-s001.zip › Figure S2 Uncropped microscopy images/Figure6/pyroptosis/nigericin/N绿1.jpg]

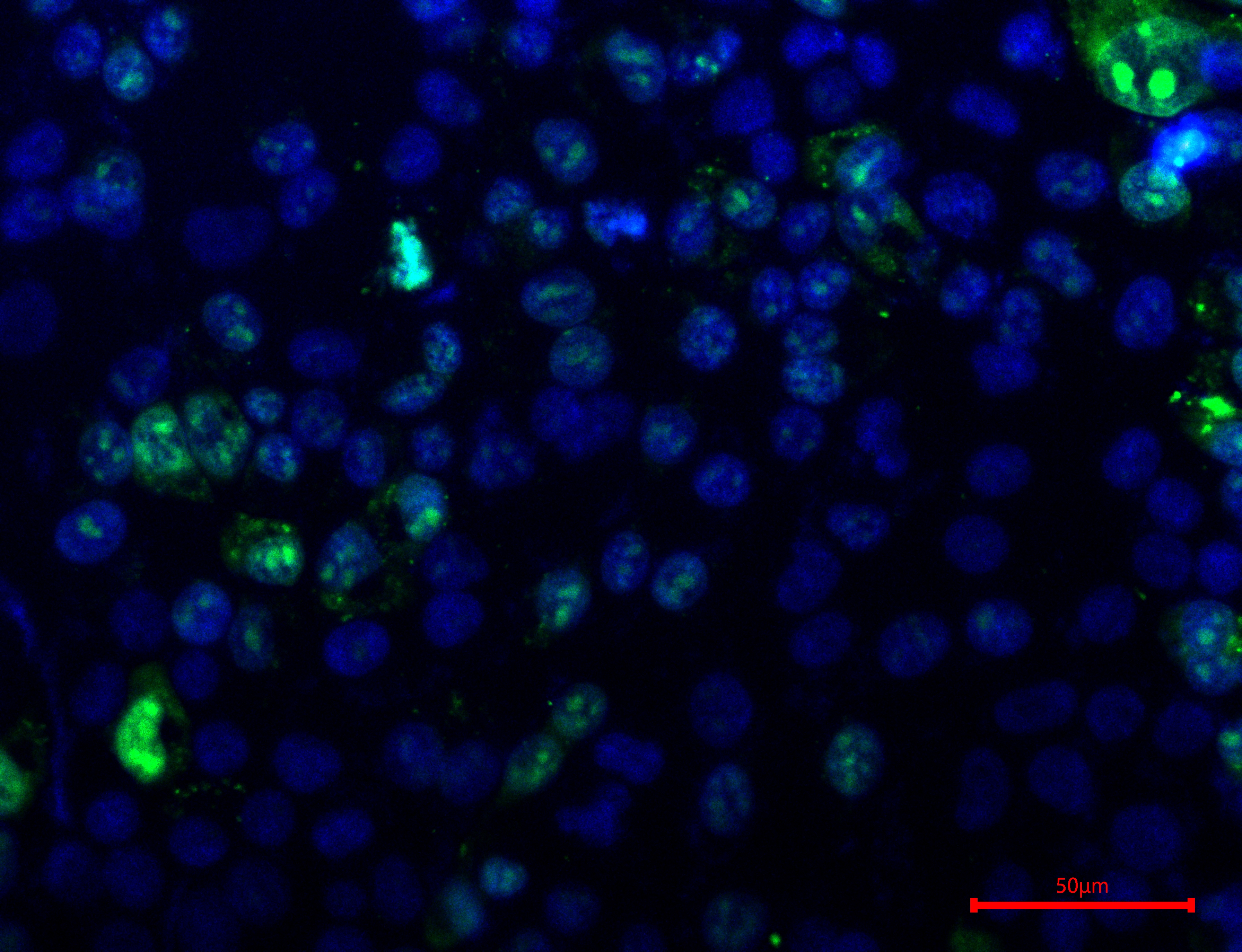

Supplement: Supplementary file 1 [file metabolites-16-00340-s001.zip › Figure S2 Uncropped microscopy images/Figure6/pyroptosis/PA/PAmerge1.jpg]

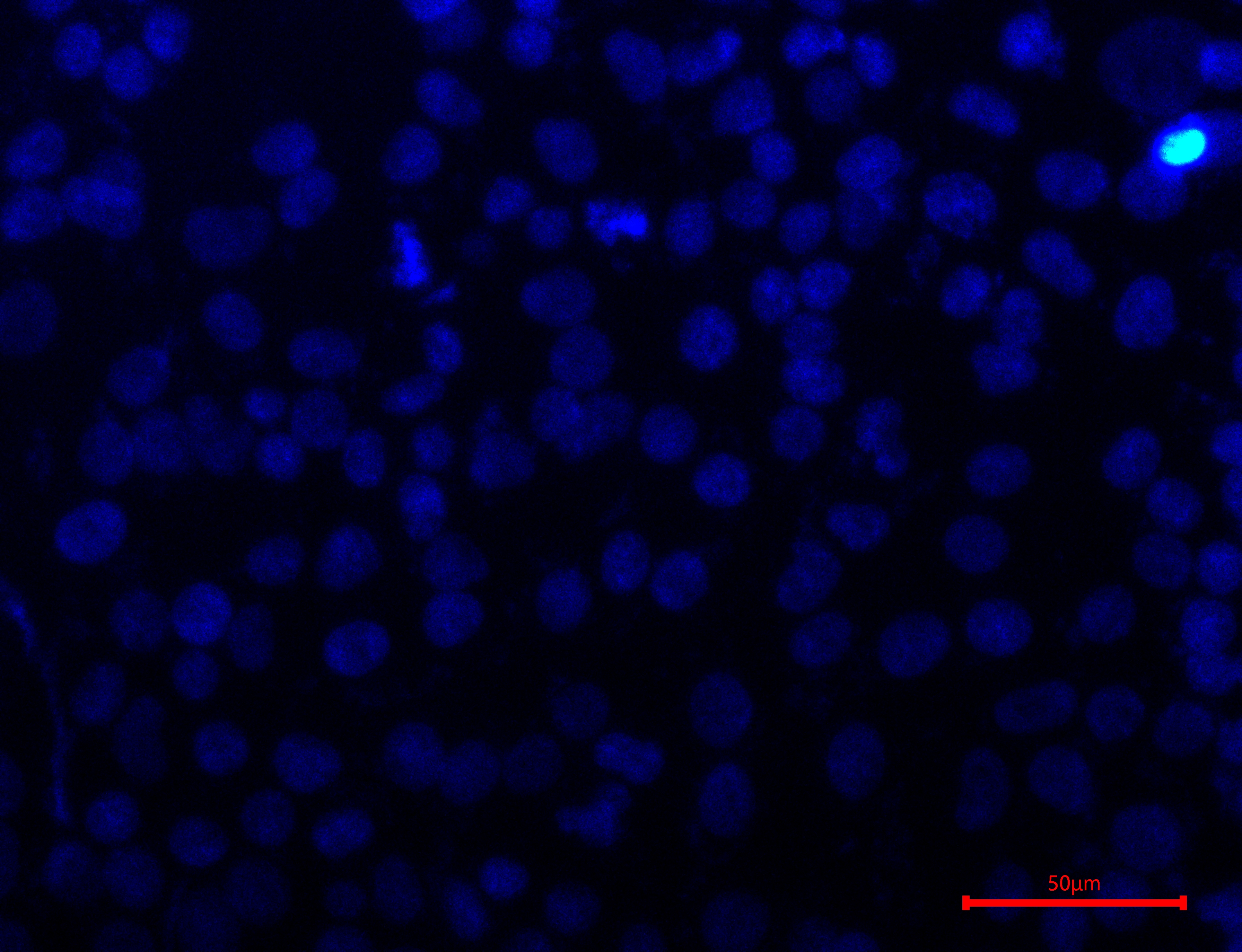

Supplement: Supplementary file 1 [file metabolites-16-00340-s001.zip › Figure S2 Uncropped microscopy images/Figure6/pyroptosis/PA/PA核1.jpg]

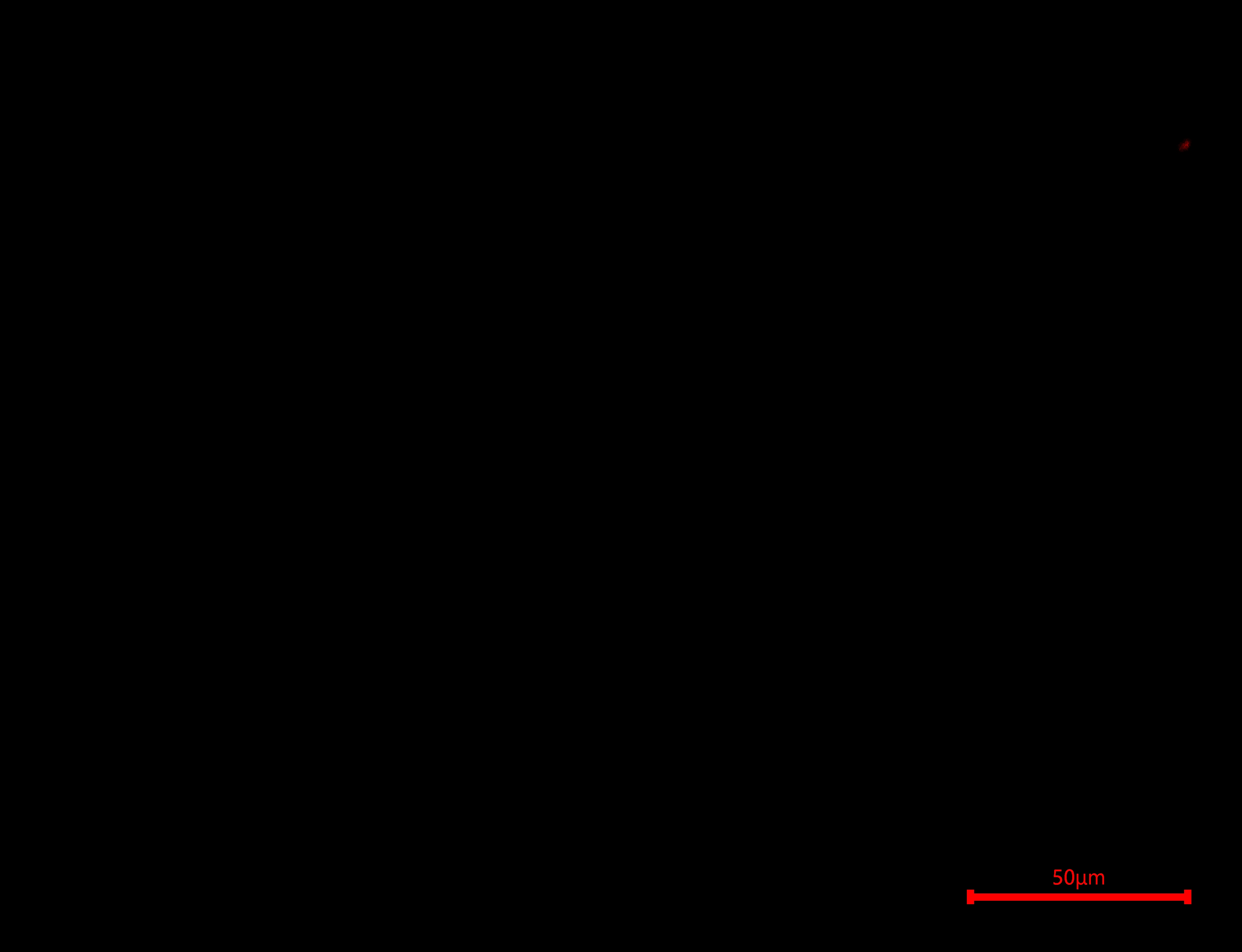

Supplement: Supplementary file 1 [file metabolites-16-00340-s001.zip › Figure S2 Uncropped microscopy images/Figure6/pyroptosis/PA/PA红1.jpg]

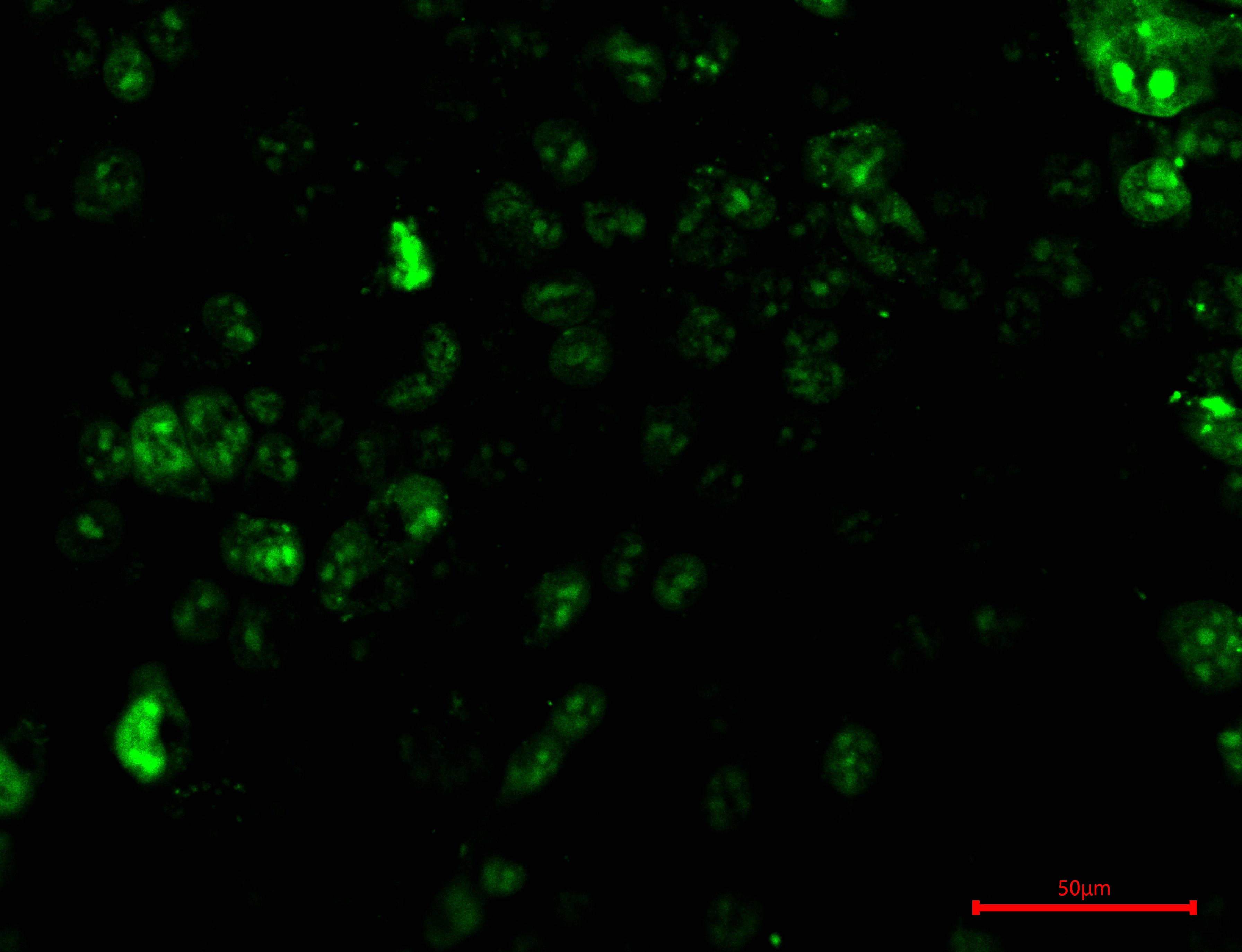

Supplement: Supplementary file 1 [file metabolites-16-00340-s001.zip › Figure S2 Uncropped microscopy images/Figure6/pyroptosis/PA/PA绿1.jpg]

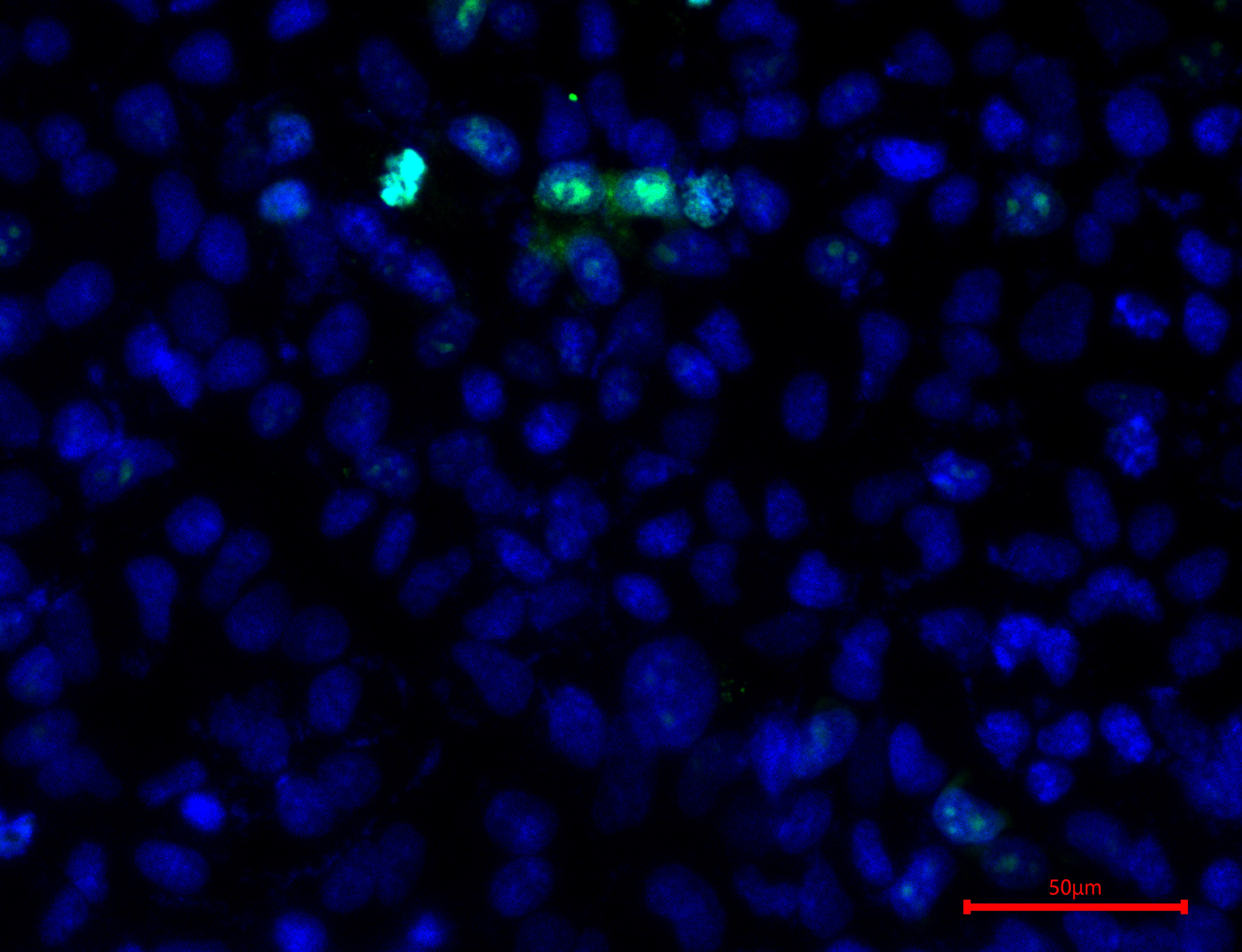

Supplement: Supplementary file 1 [file metabolites-16-00340-s001.zip › Figure S2 Uncropped microscopy images/Figure6/pyroptosis/PQQ/PQQmerge3.jpg]

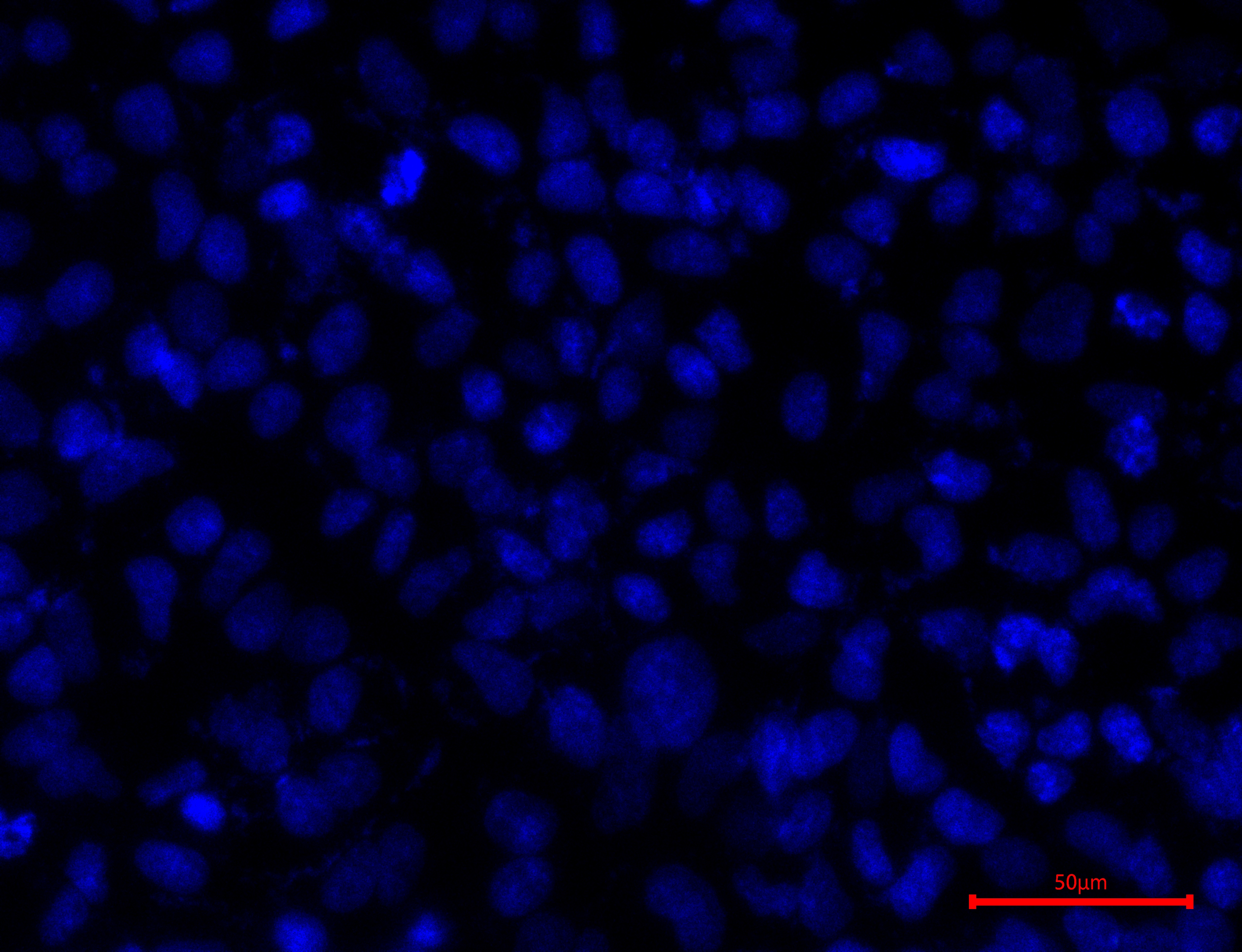

Supplement: Supplementary file 1 [file metabolites-16-00340-s001.zip › Figure S2 Uncropped microscopy images/Figure6/pyroptosis/PQQ/PQQ核3.jpg]

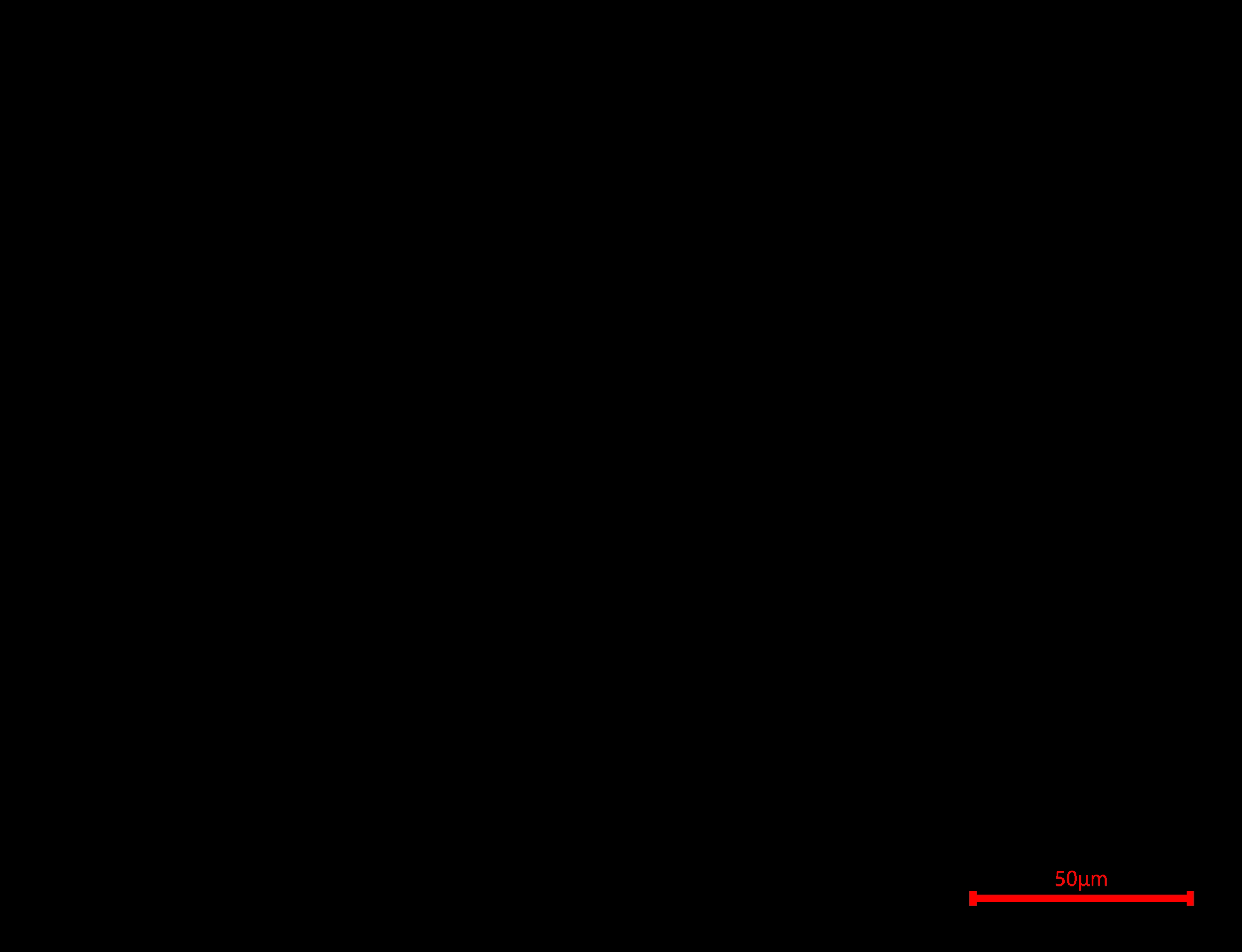

Supplement: Supplementary file 1 [file metabolites-16-00340-s001.zip › Figure S2 Uncropped microscopy images/Figure6/pyroptosis/PQQ/PQQ红3.jpg]

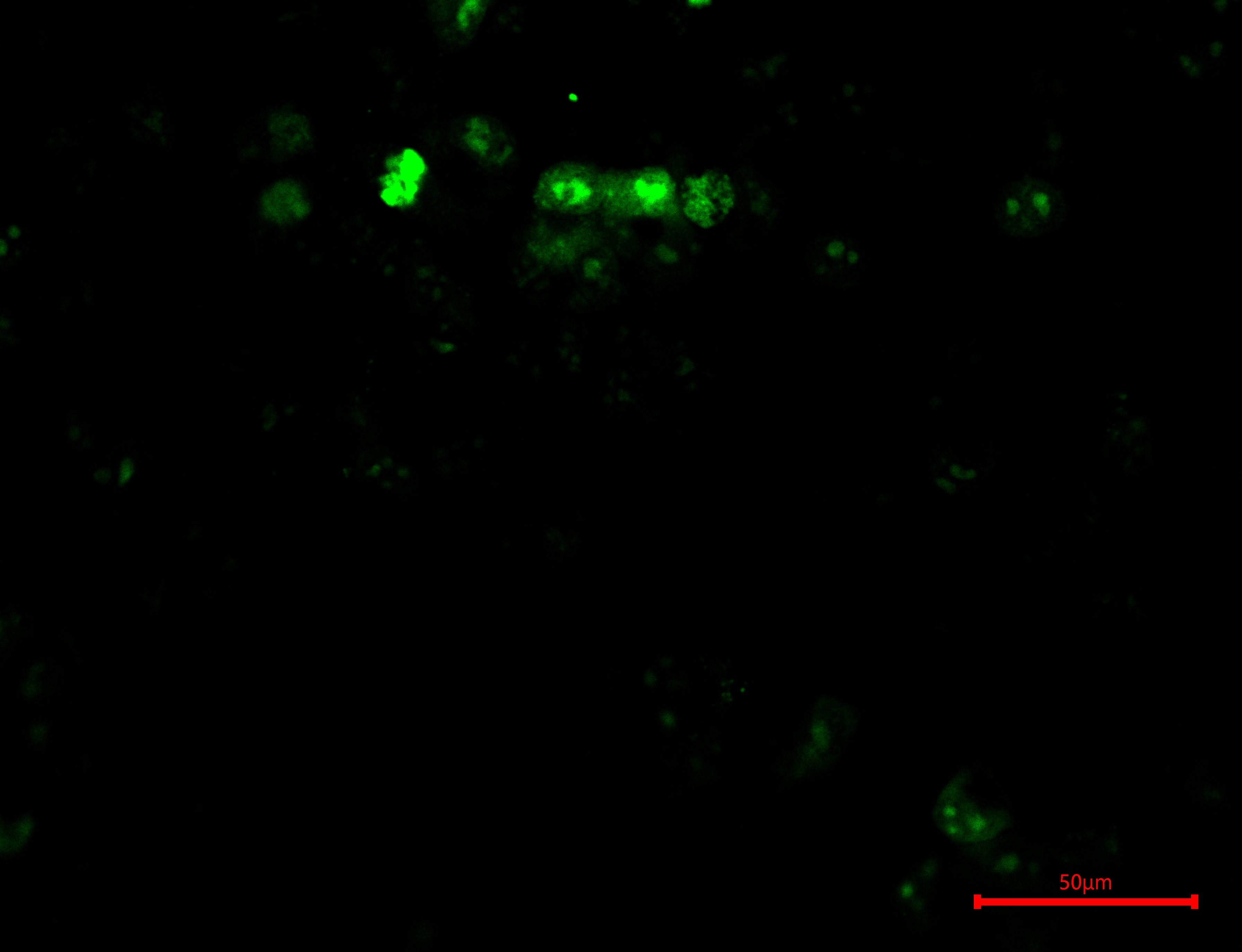

Supplement: Supplementary file 1 [file metabolites-16-00340-s001.zip › Figure S2 Uncropped microscopy images/Figure6/pyroptosis/PQQ/PQQ绿3.jpg]

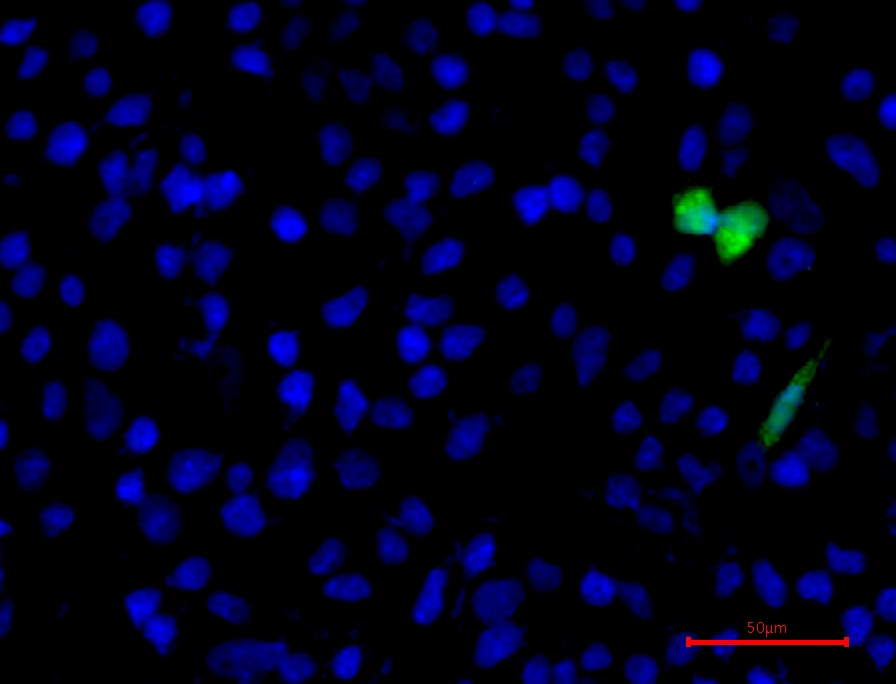

Supplement: Supplementary file 1 [file metabolites-16-00340-s001.zip › Figure S2 Uncropped microscopy images/Figure6/ROS/ctl/CTL merge6.jpg]

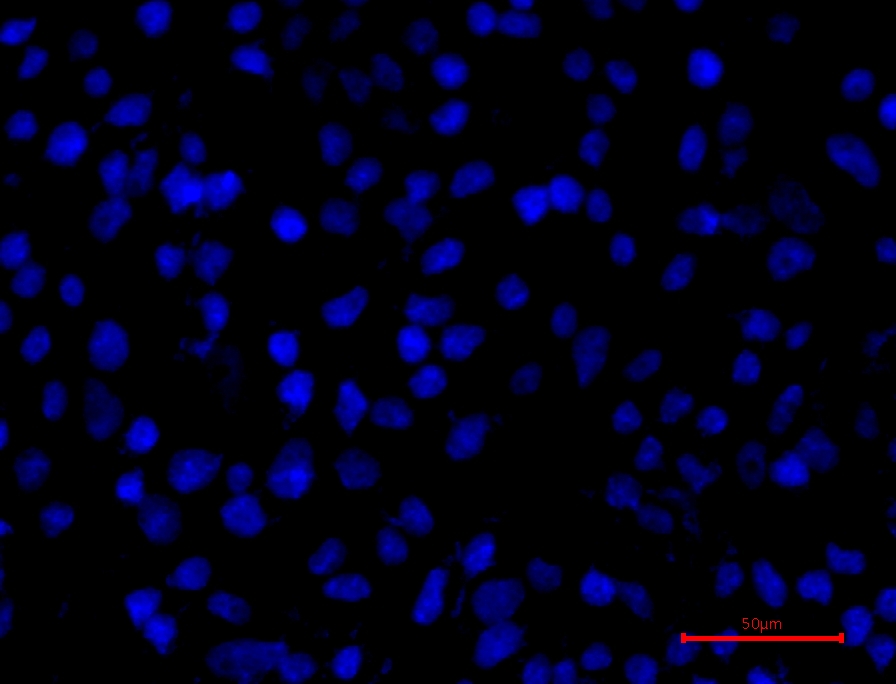

Supplement: Supplementary file 1 [file metabolites-16-00340-s001.zip › Figure S2 Uncropped microscopy images/Figure6/ROS/ctl/CTL核6.jpg]

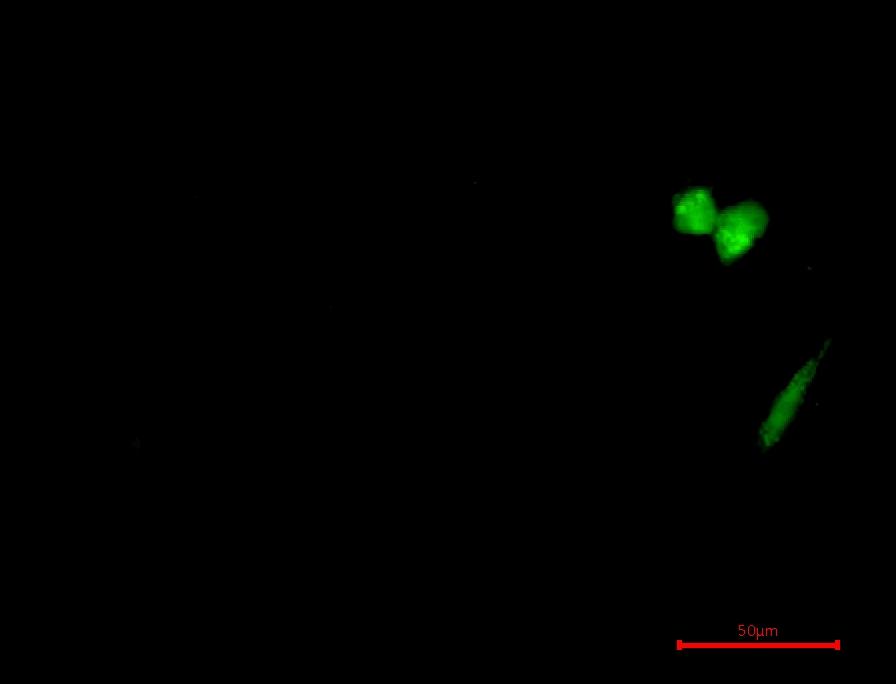

Supplement: Supplementary file 1 [file metabolites-16-00340-s001.zip › Figure S2 Uncropped microscopy images/Figure6/ROS/ctl/CTL绿6.jpg]

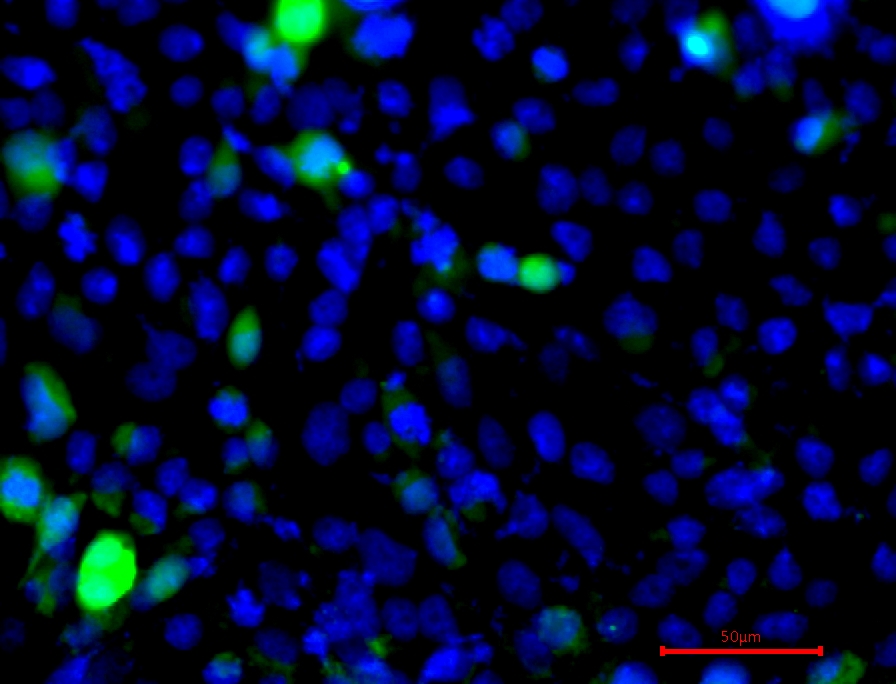

Supplement: Supplementary file 1 [file metabolites-16-00340-s001.zip › Figure S2 Uncropped microscopy images/Figure6/ROS/PA/PA merge1.jpg]

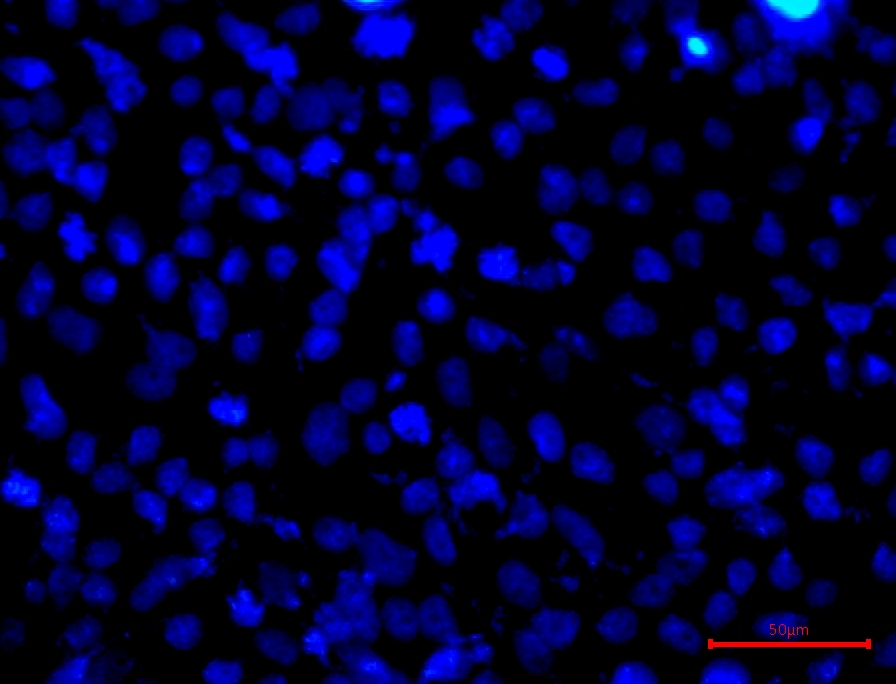

Supplement: Supplementary file 1 [file metabolites-16-00340-s001.zip › Figure S2 Uncropped microscopy images/Figure6/ROS/PA/PA核1.jpg]

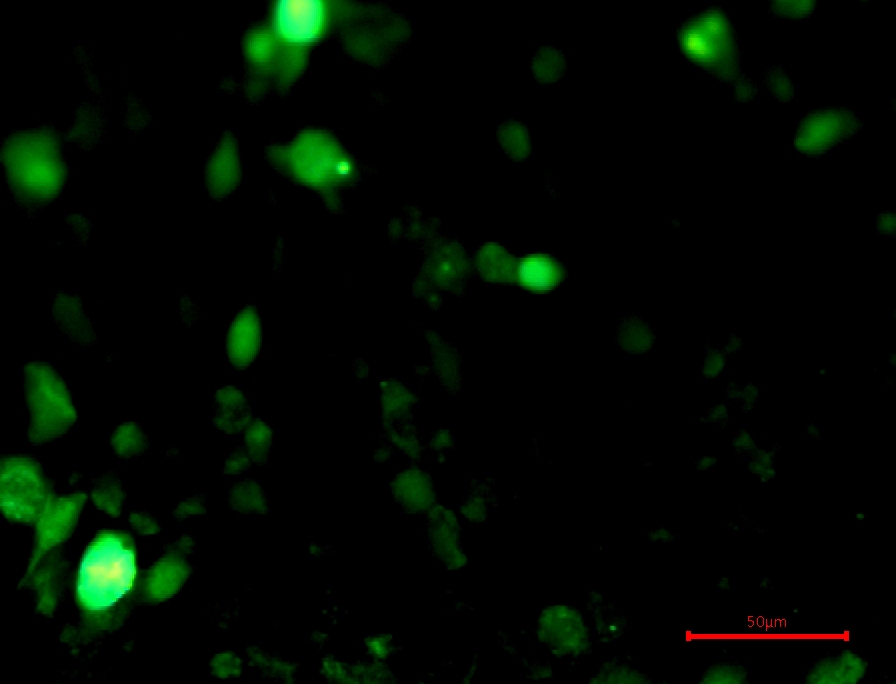

Supplement: Supplementary file 1 [file metabolites-16-00340-s001.zip › Figure S2 Uncropped microscopy images/Figure6/ROS/PA/PA绿1.jpg]

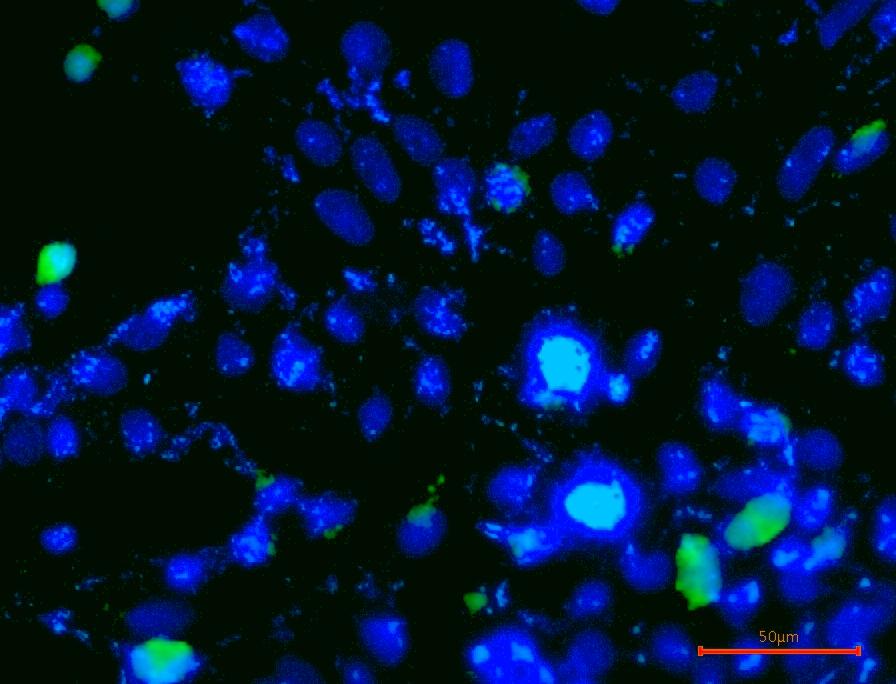

Supplement: Supplementary file 1 [file metabolites-16-00340-s001.zip › Figure S2 Uncropped microscopy images/Figure6/ROS/PQQ/PQQ merge1.jpg]

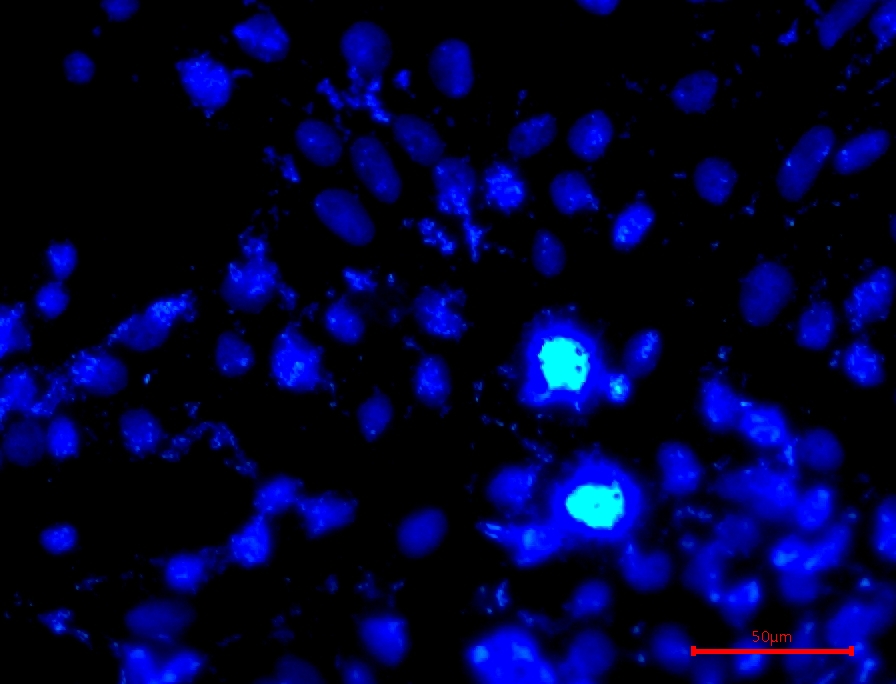

Supplement: Supplementary file 1 [file metabolites-16-00340-s001.zip › Figure S2 Uncropped microscopy images/Figure6/ROS/PQQ/PQQ核1.jpg]

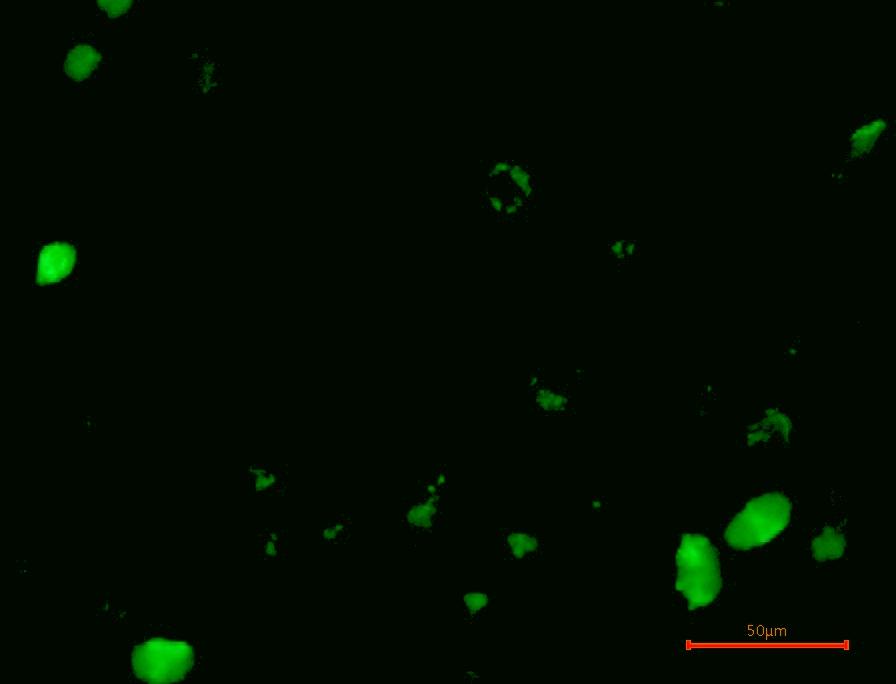

Supplement: Supplementary file 1 [file metabolites-16-00340-s001.zip › Figure S2 Uncropped microscopy images/Figure6/ROS/PQQ/PQQ绿1.jpg]

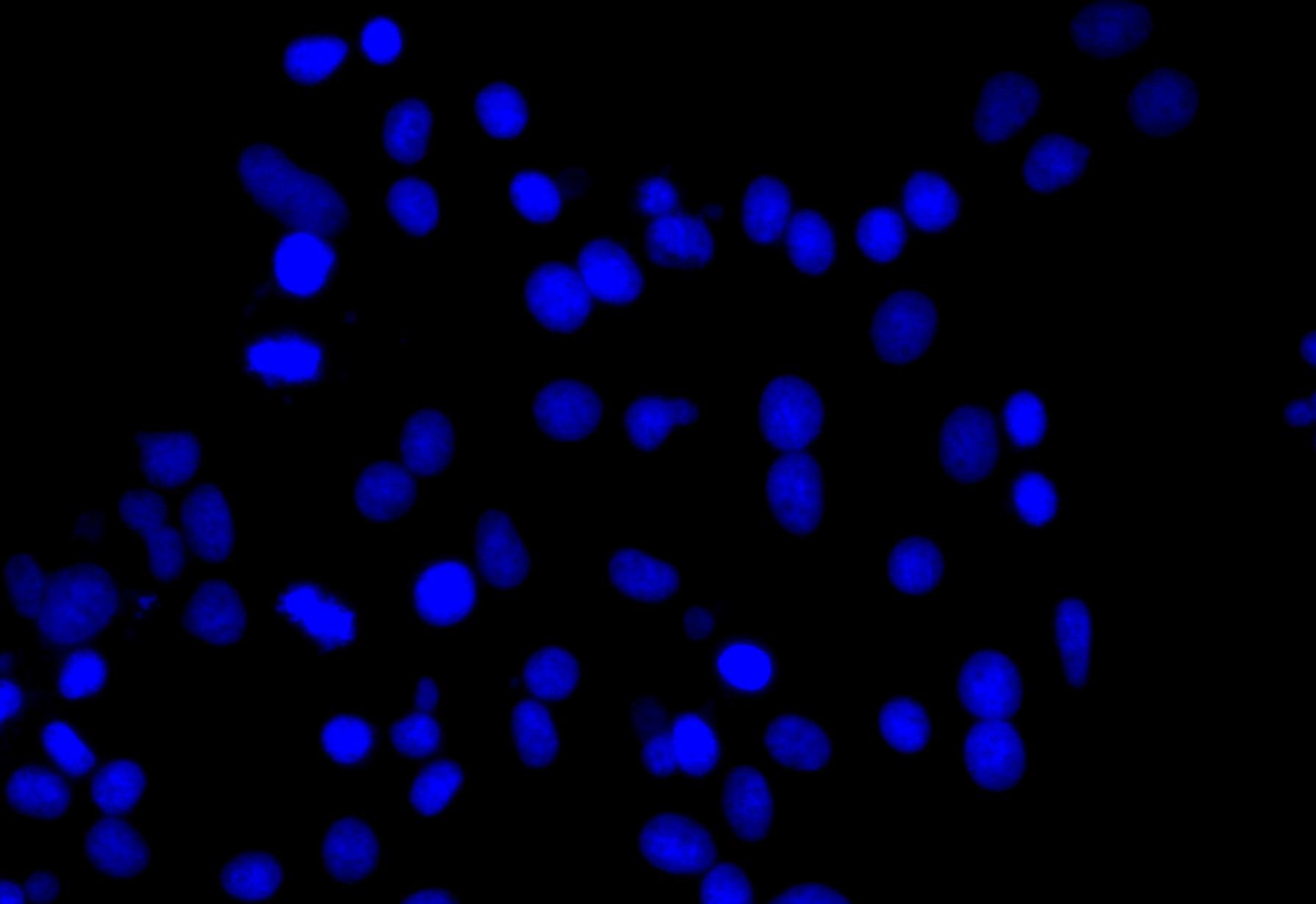

Supplement: Supplementary file 1 [file metabolites-16-00340-s001.zip › Figure S2 Uncropped microscopy images/Figure7/Beclin/Beclin-1 PA+PQQ1.1.png]

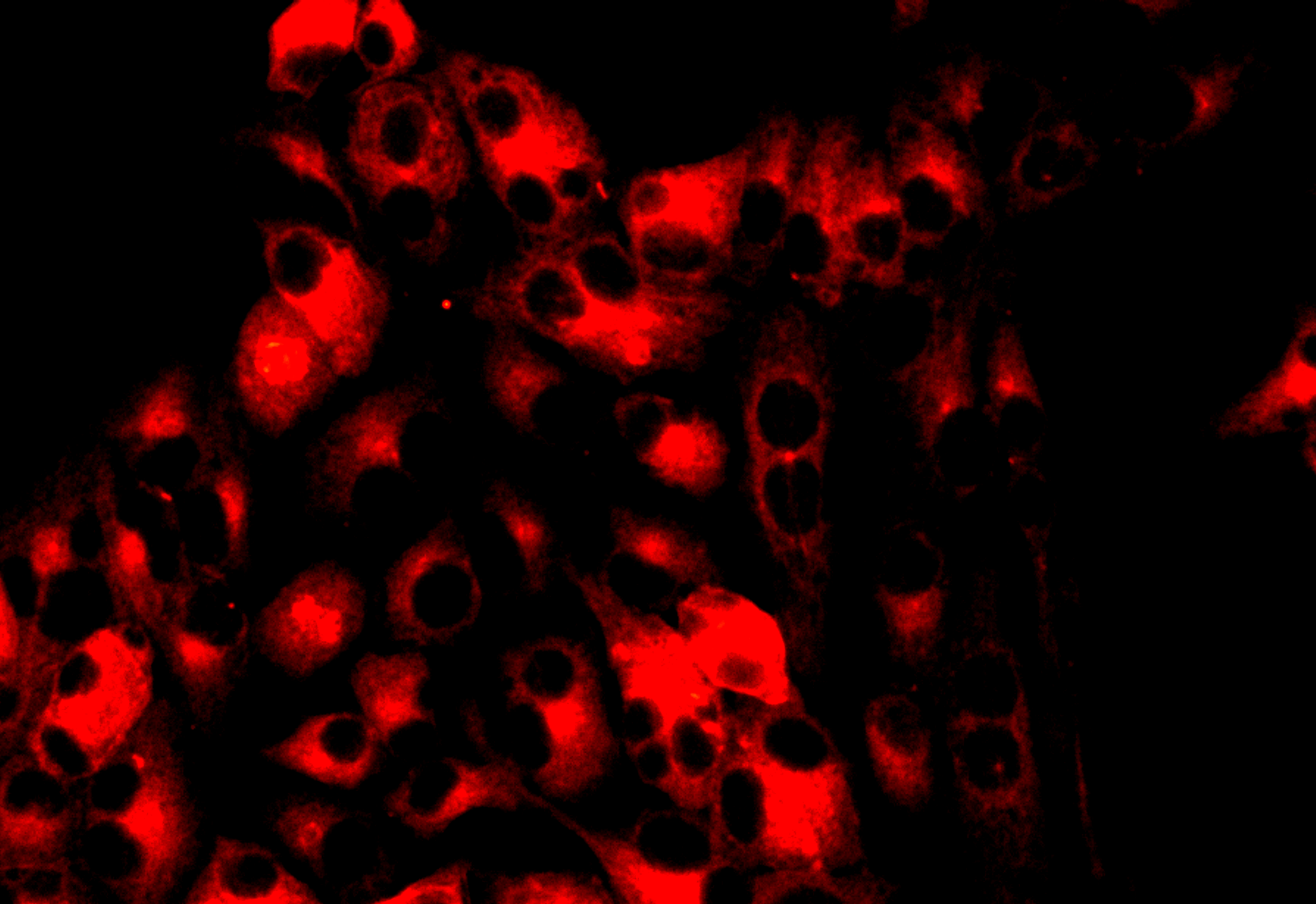

Supplement: Supplementary file 1 [file metabolites-16-00340-s001.zip › Figure S2 Uncropped microscopy images/Figure7/Beclin(1)/Beclin-1 PA+PQQ1.2.png]

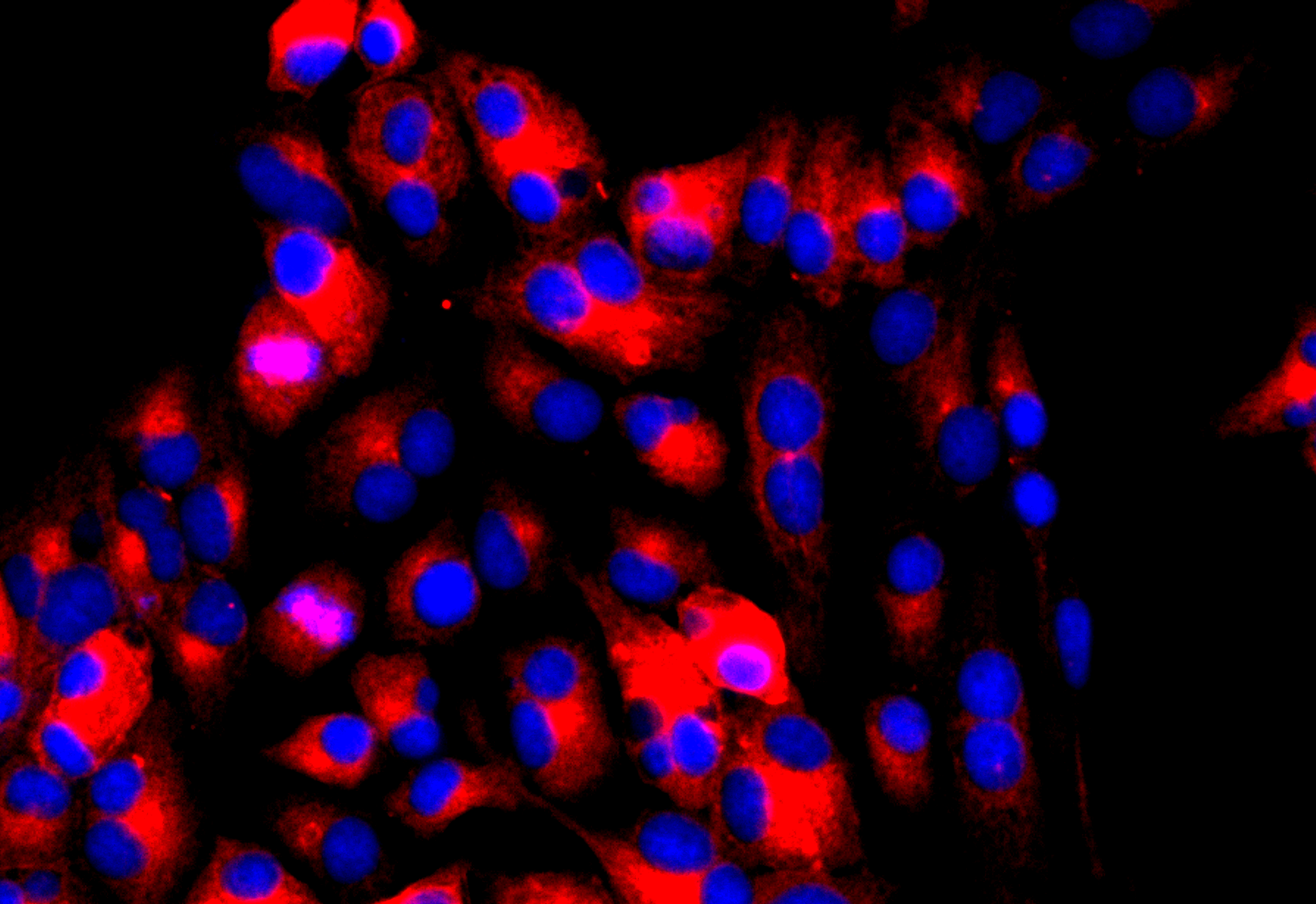

Supplement: Supplementary file 1 [file metabolites-16-00340-s001.zip › Figure S2 Uncropped microscopy images/Figure7/Beclin(1)/Beclin-1 PA+PQQ1.3Merge.png]

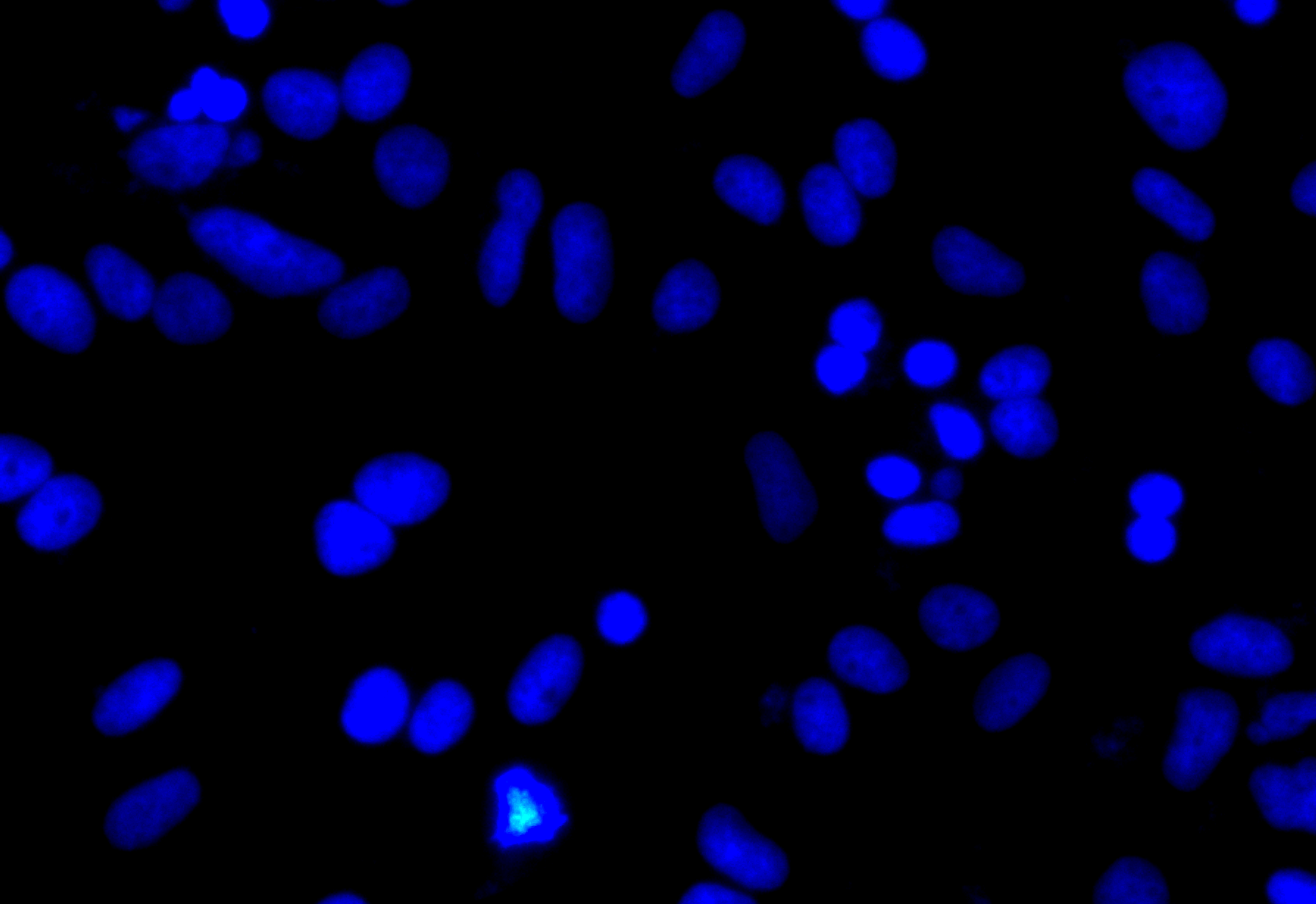

Supplement: Supplementary file 1 [file metabolites-16-00340-s001.zip › Figure S2 Uncropped microscopy images/Figure7/Beclin(1)/Beclin-1 PA3.1.png]

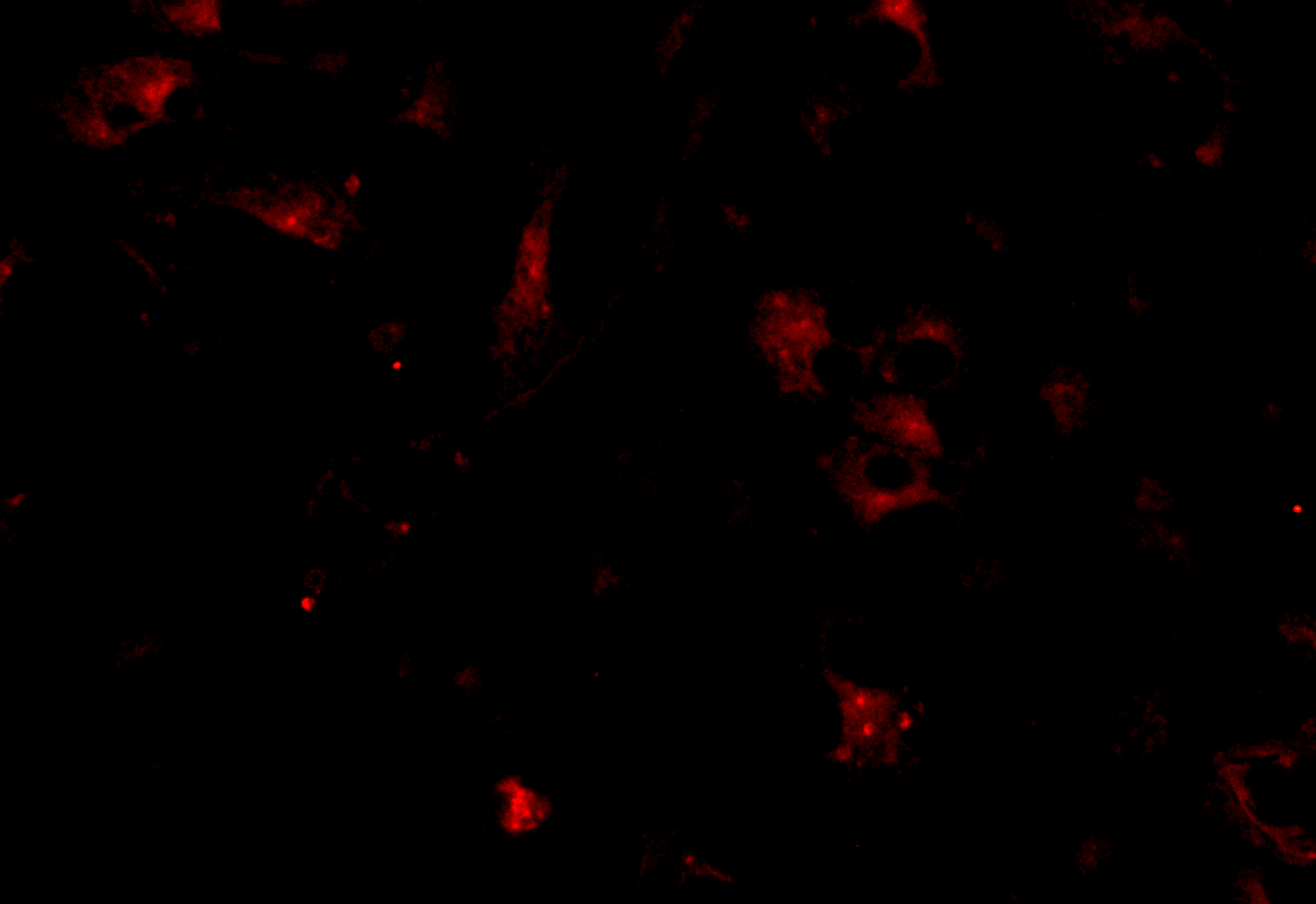

Supplement: Supplementary file 1 [file metabolites-16-00340-s001.zip › Figure S2 Uncropped microscopy images/Figure7/Beclin(1)/Beclin-1 PA3.2.png]

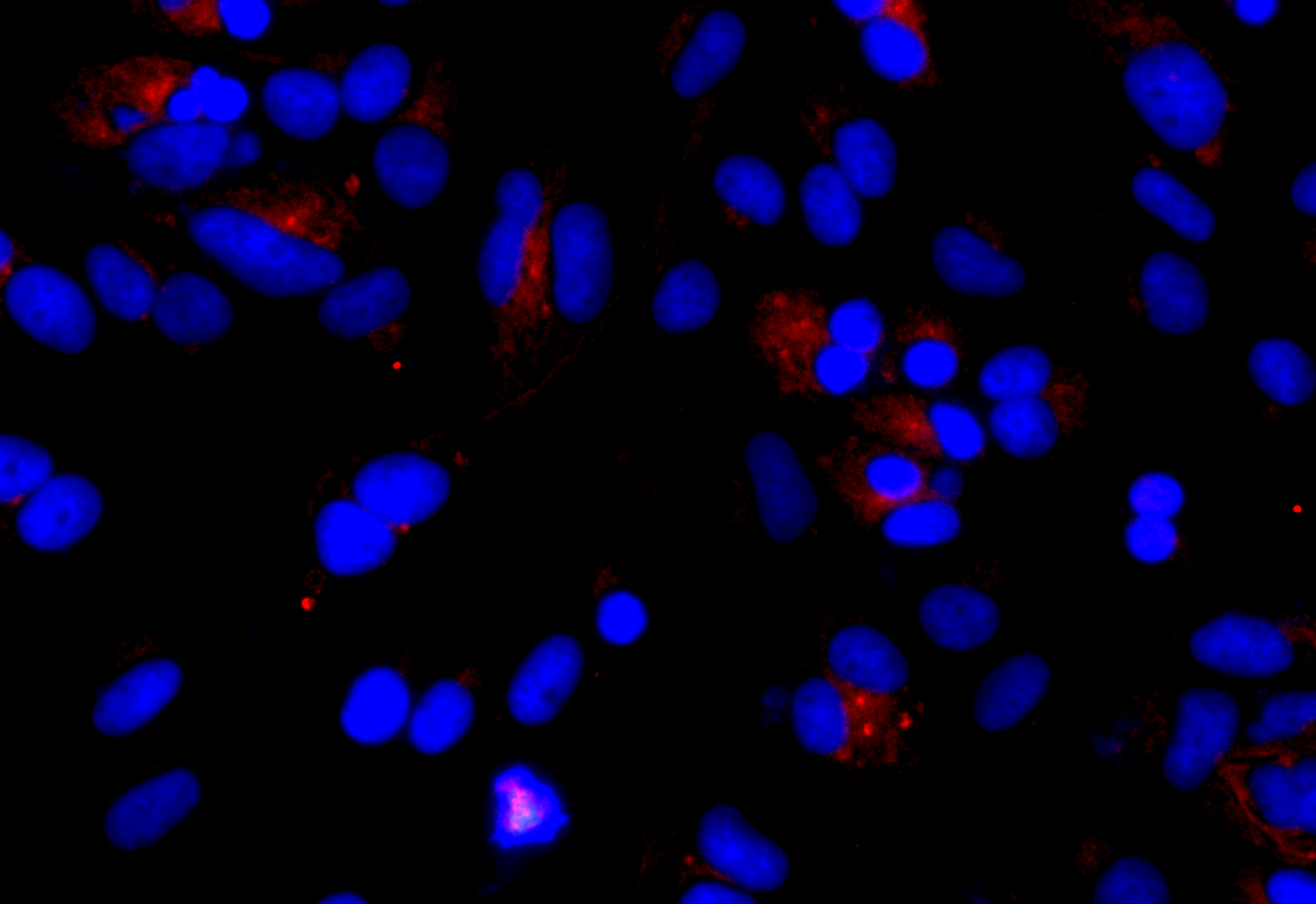

Supplement: Supplementary file 1 [file metabolites-16-00340-s001.zip › Figure S2 Uncropped microscopy images/Figure7/Beclin(1)/Beclin-1 PA3.3Merge.png]

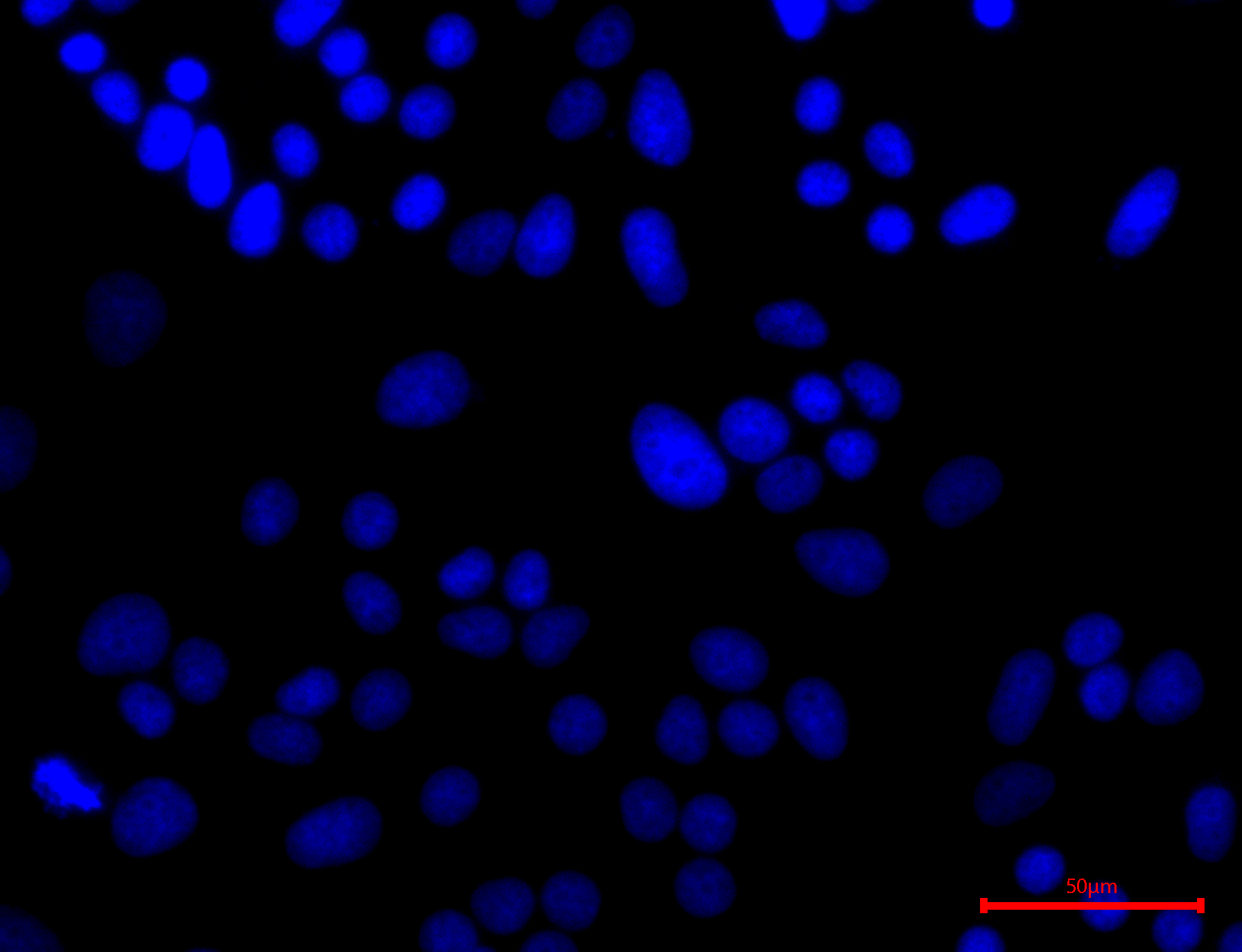

Supplement: Supplementary file 1 [file metabolites-16-00340-s001.zip › Figure S2 Uncropped microscopy images/Figure7/Beclin(1)/1Beclin-1 Ctl3.1.tif]

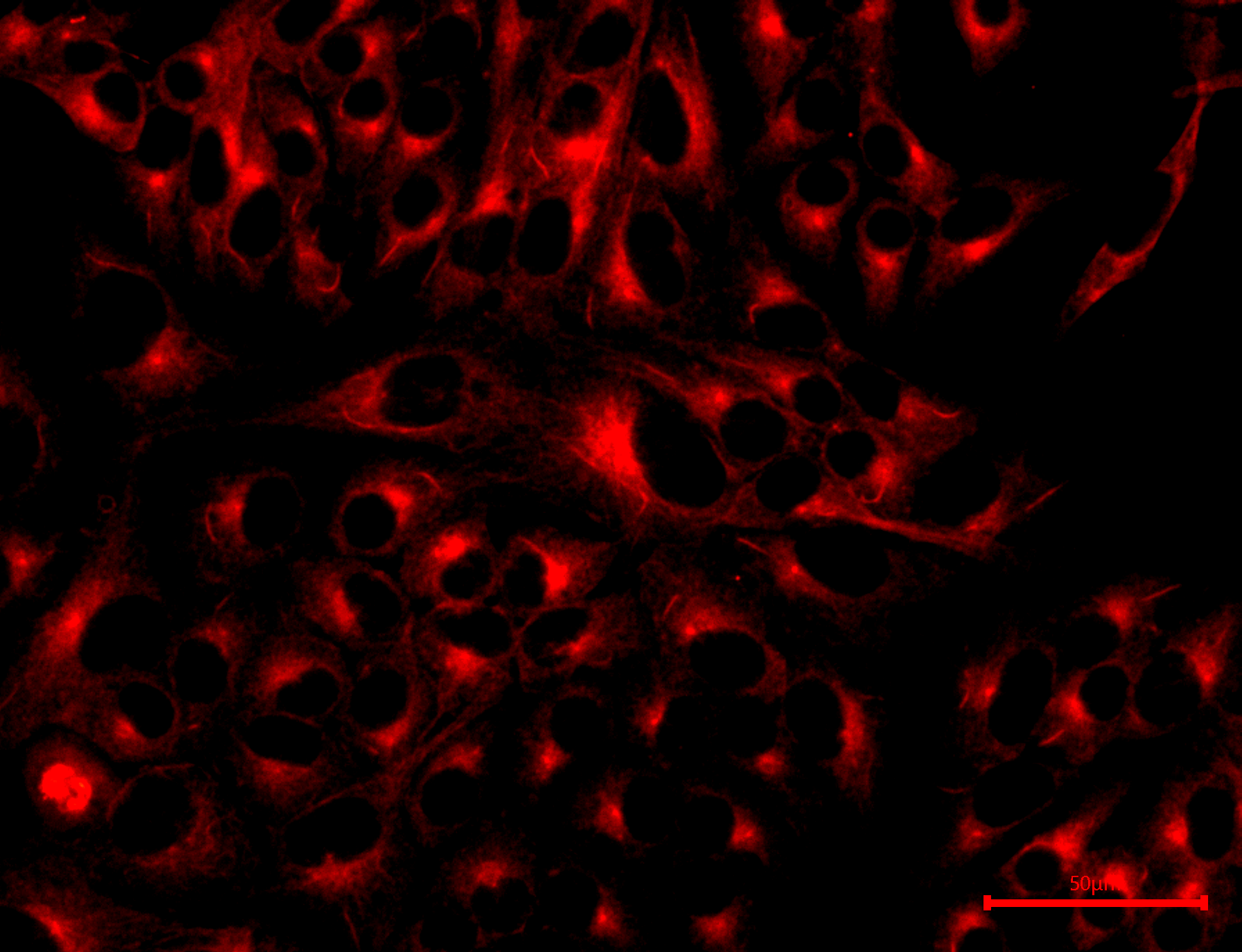

Supplement: Supplementary file 1 [file metabolites-16-00340-s001.zip › Figure S2 Uncropped microscopy images/Figure7/Beclin(1)/1Beclin-1 Ctl3.2.tif]

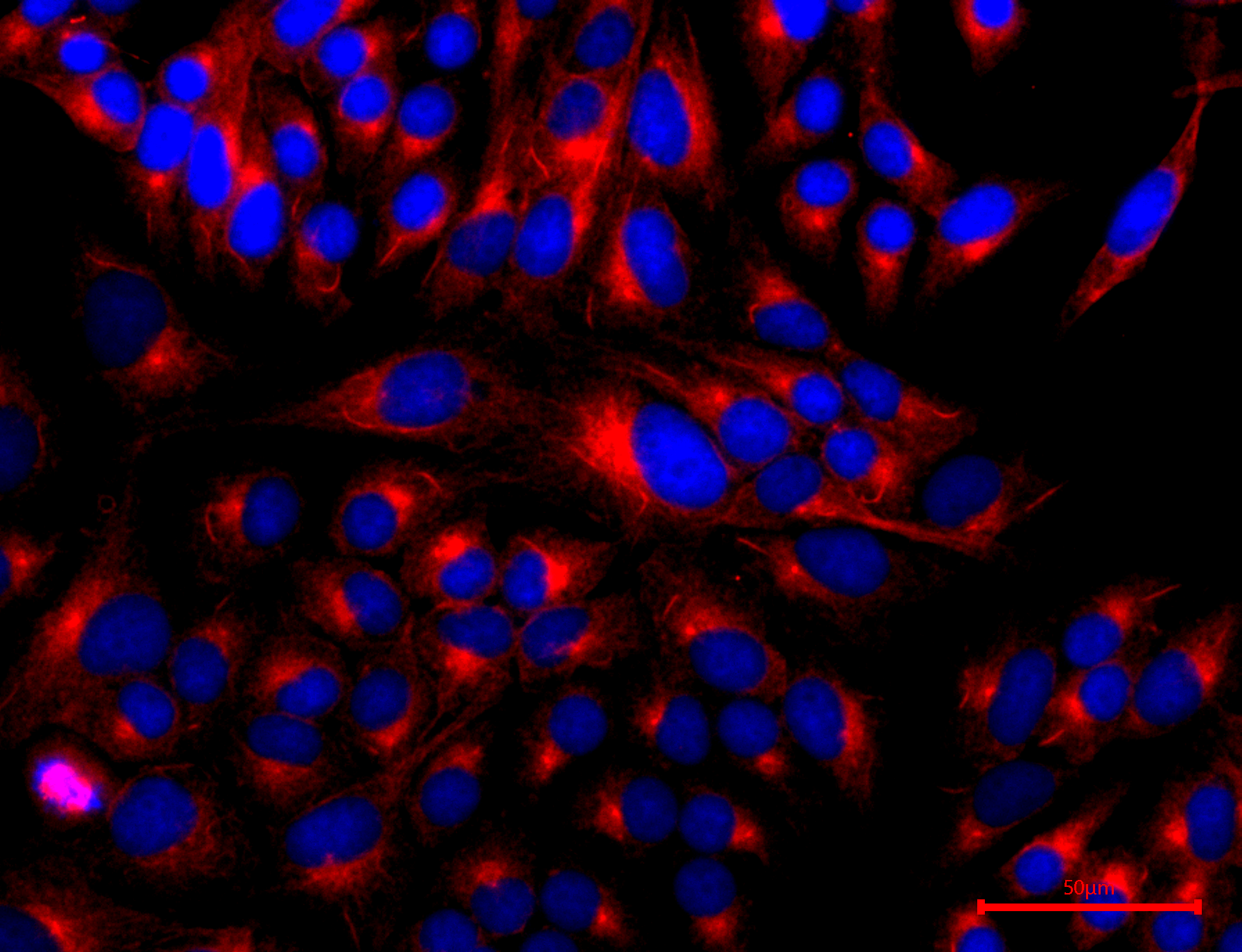

Supplement: Supplementary file 1 [file metabolites-16-00340-s001.zip › Figure S2 Uncropped microscopy images/Figure7/Beclin(1)/1Beclin-1 Ctl3.3Merge.tif]

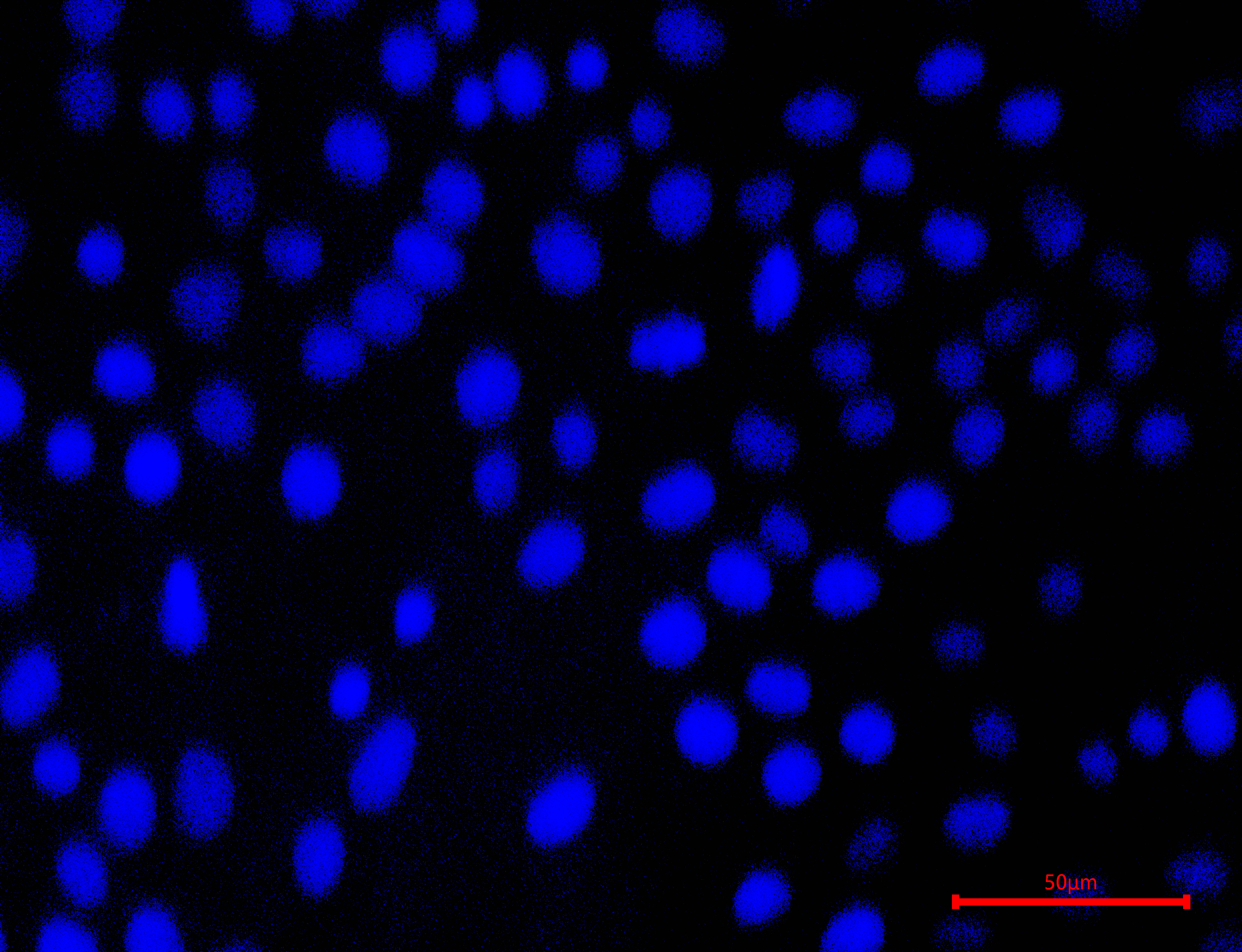

Supplement: Supplementary file 1 [file metabolites-16-00340-s001.zip › Figure S2 Uncropped microscopy images/Figure7/p62/1p62 CTL3.1.tif]

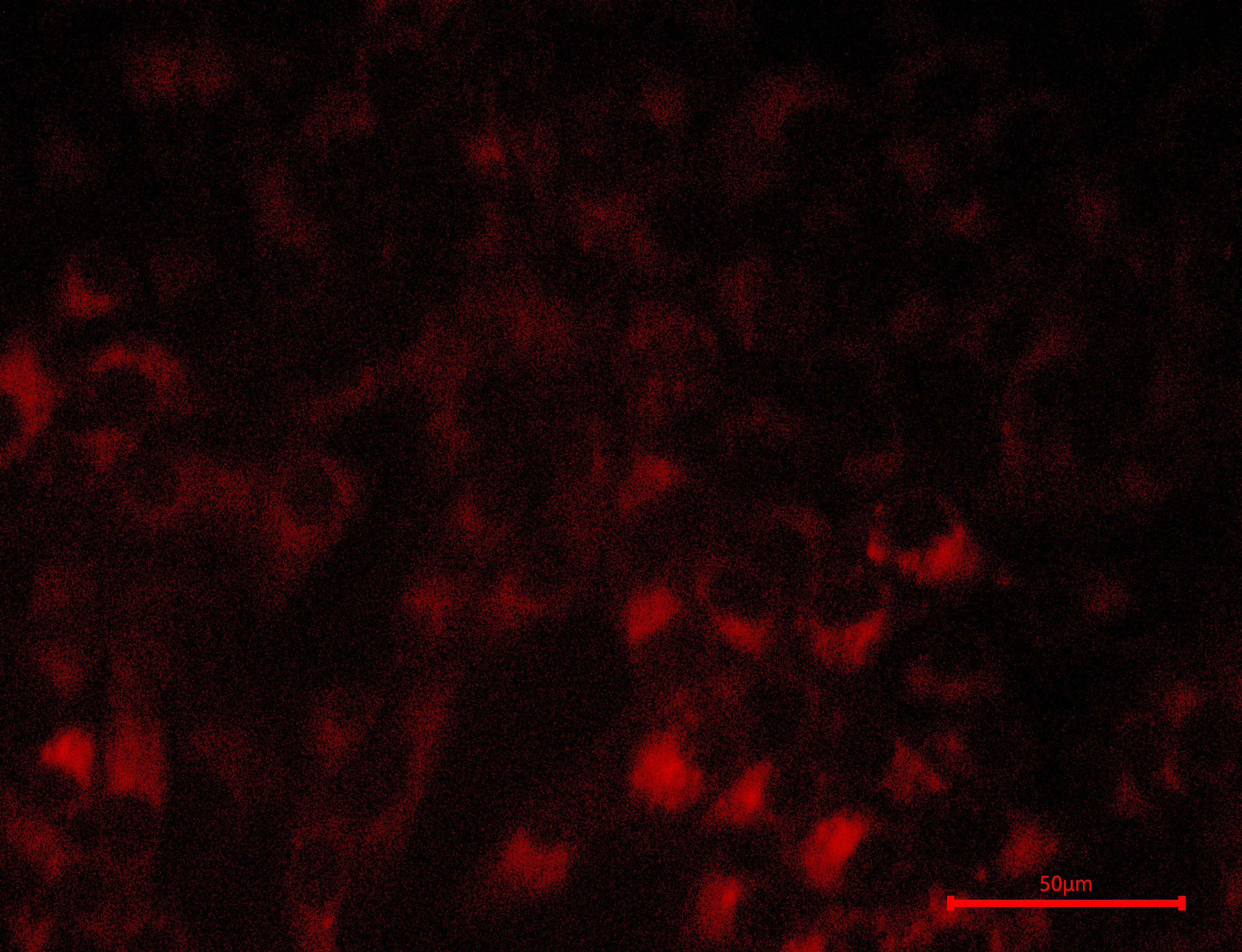

Supplement: Supplementary file 1 [file metabolites-16-00340-s001.zip › Figure S2 Uncropped microscopy images/Figure7/p62/1p62 CTL3.2.tif]

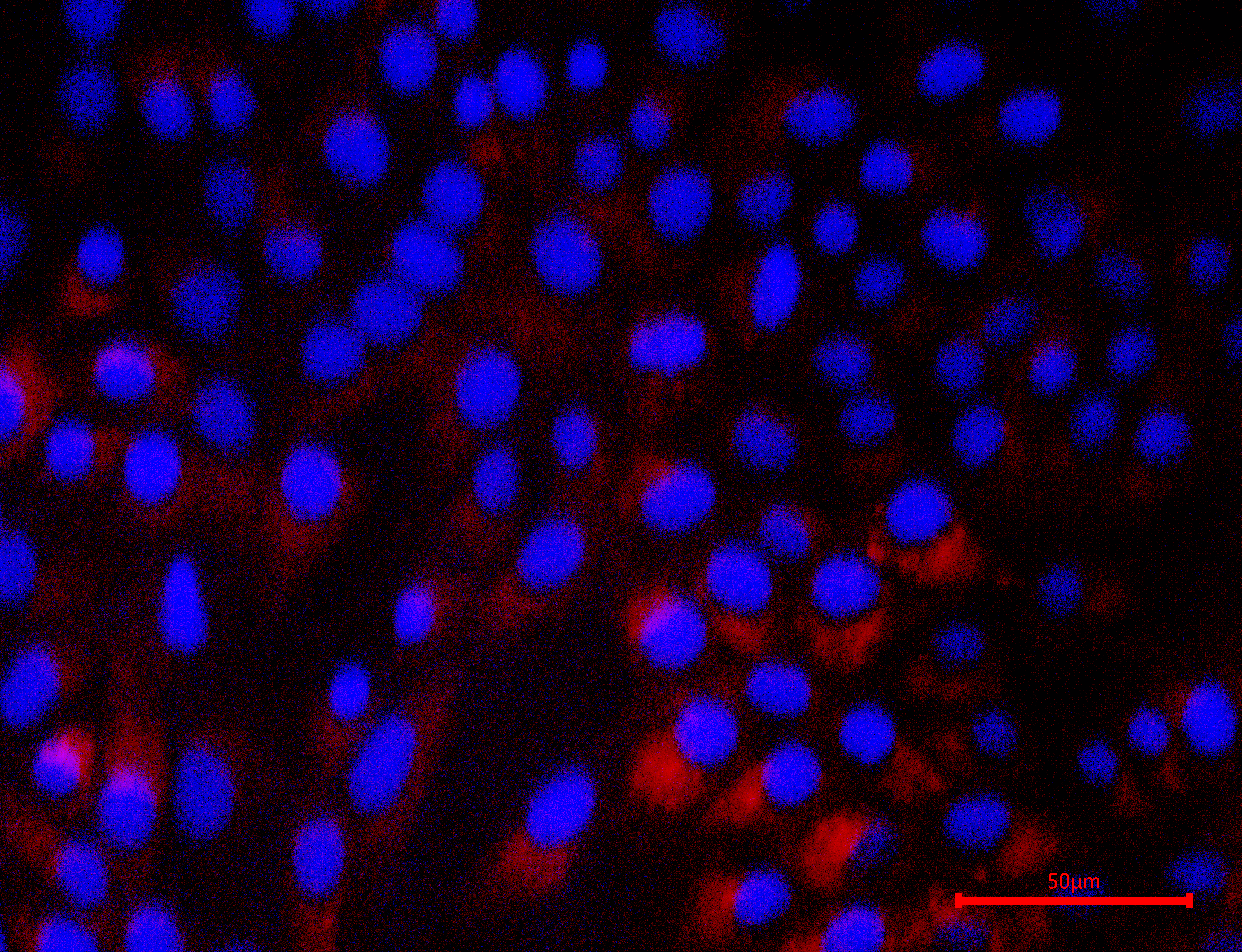

Supplement: Supplementary file 1 [file metabolites-16-00340-s001.zip › Figure S2 Uncropped microscopy images/Figure7/p62/1p62 CTL3.3Merge.tif]

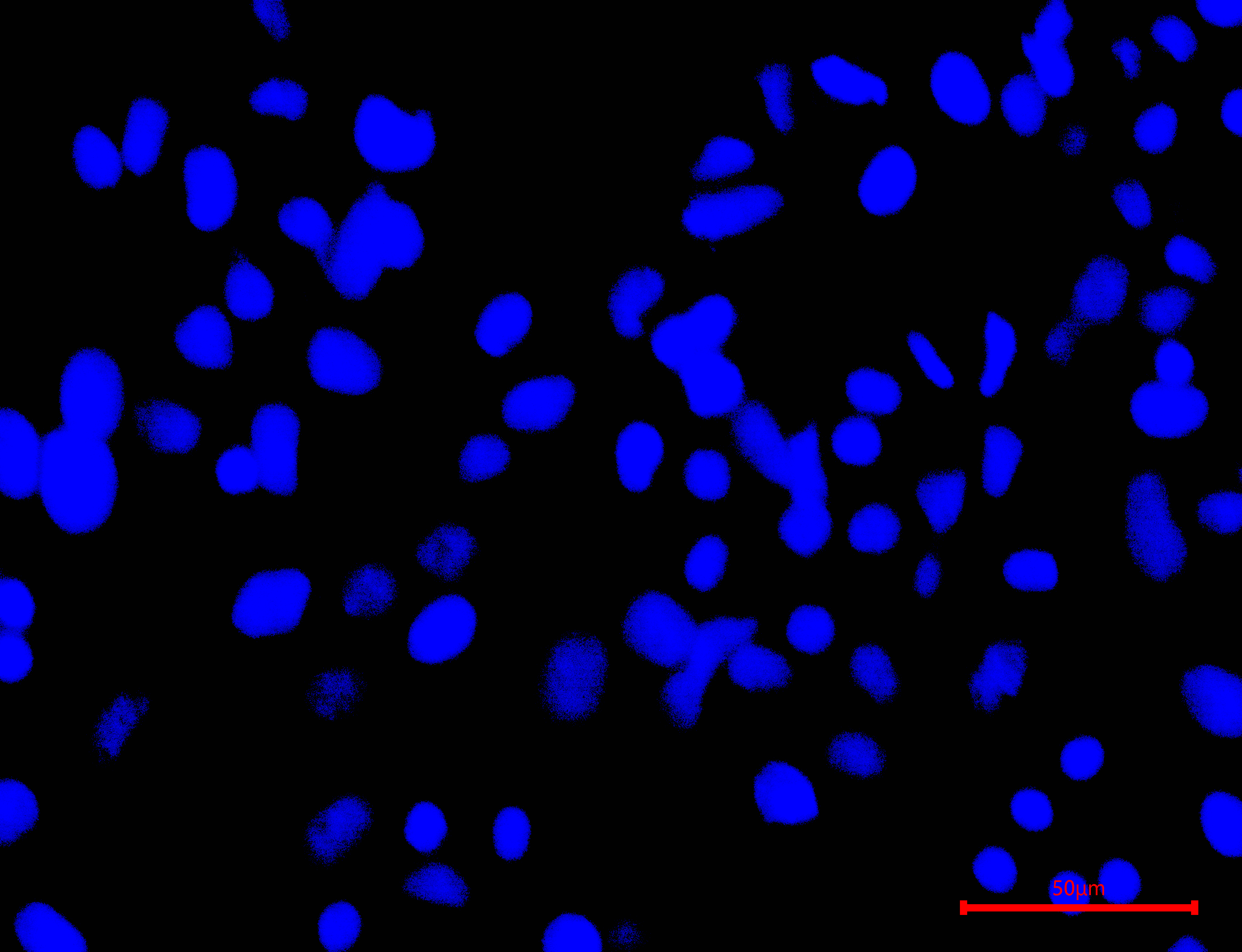

Supplement: Supplementary file 1 [file metabolites-16-00340-s001.zip › Figure S2 Uncropped microscopy images/Figure7/p62/2p62 PA1.1.tif]

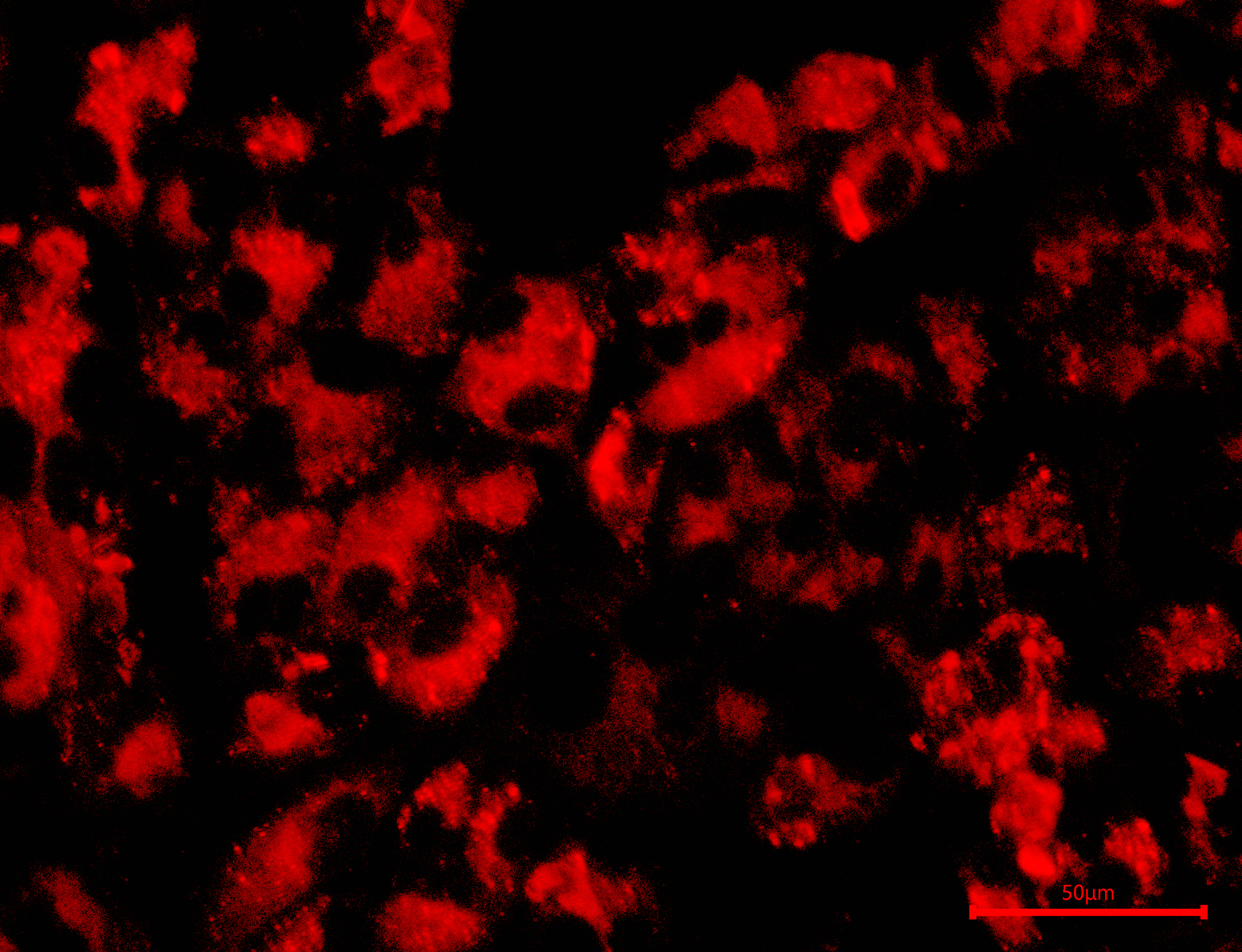

Supplement: Supplementary file 1 [file metabolites-16-00340-s001.zip › Figure S2 Uncropped microscopy images/Figure7/p62/2p62 PA1.2.tif]

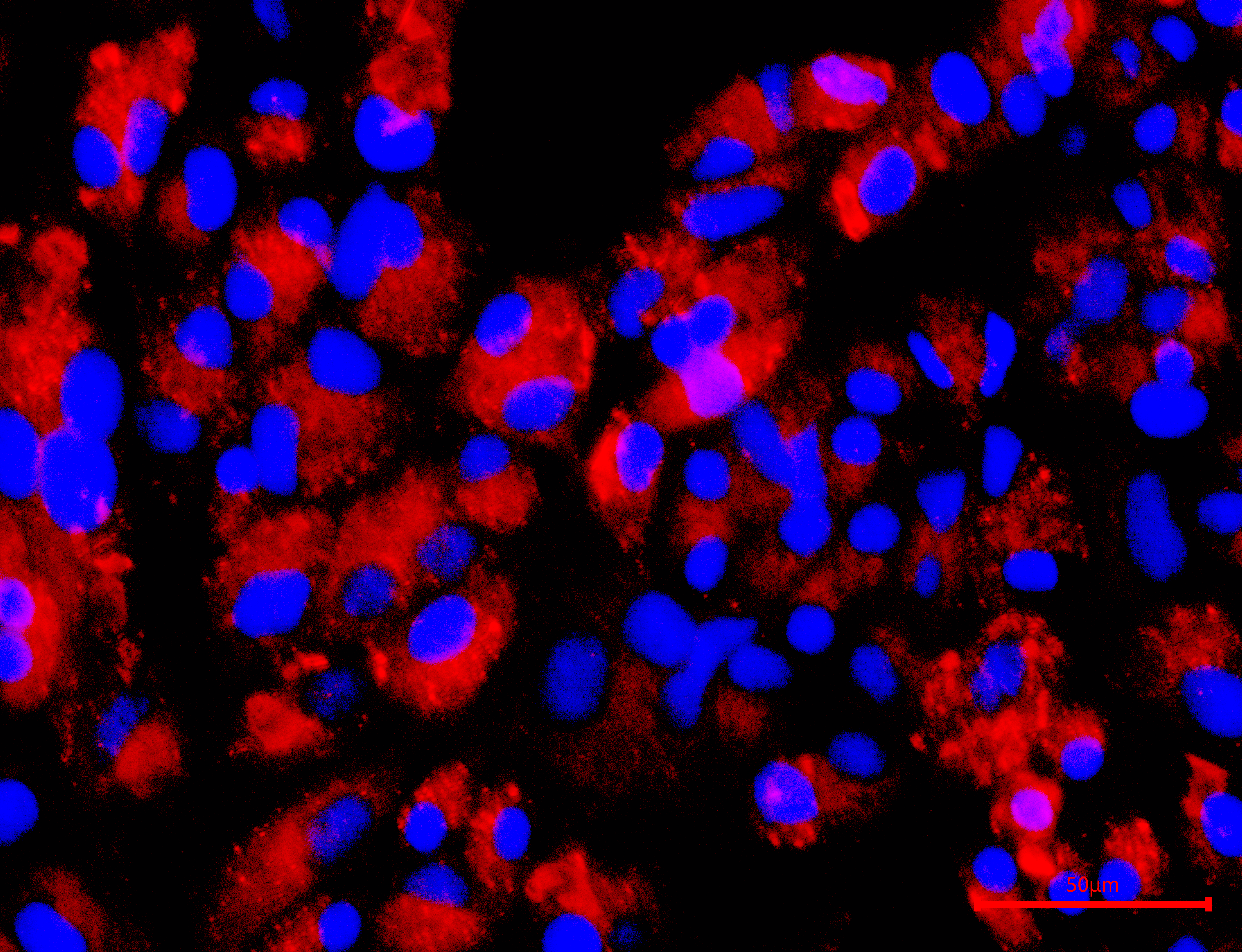

Supplement: Supplementary file 1 [file metabolites-16-00340-s001.zip › Figure S2 Uncropped microscopy images/Figure7/p62/2p62 PA1.3Merge.tif]

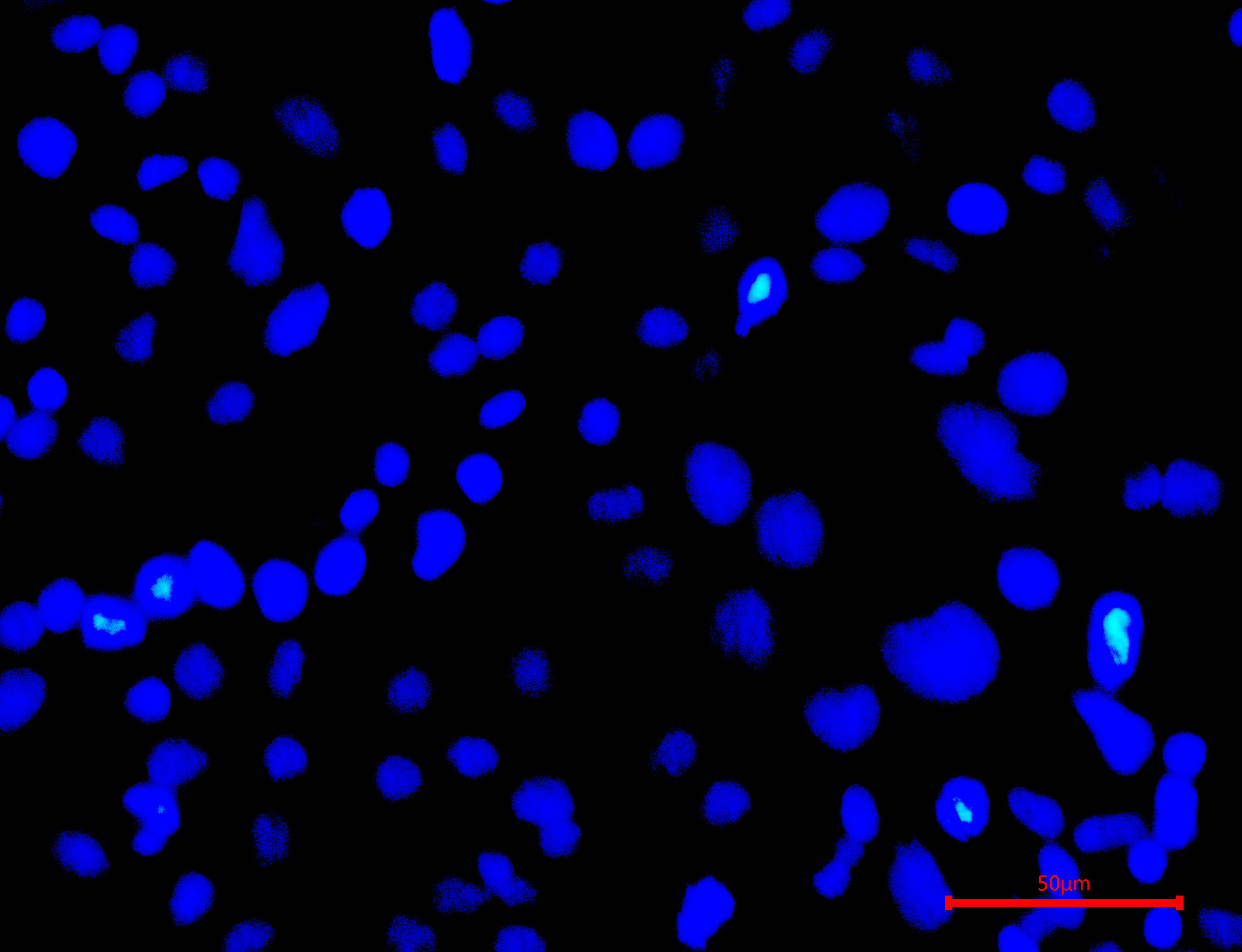

Supplement: Supplementary file 1 [file metabolites-16-00340-s001.zip › Figure S2 Uncropped microscopy images/Figure7/p62/3p62 PA+PQQ2.1.tif]

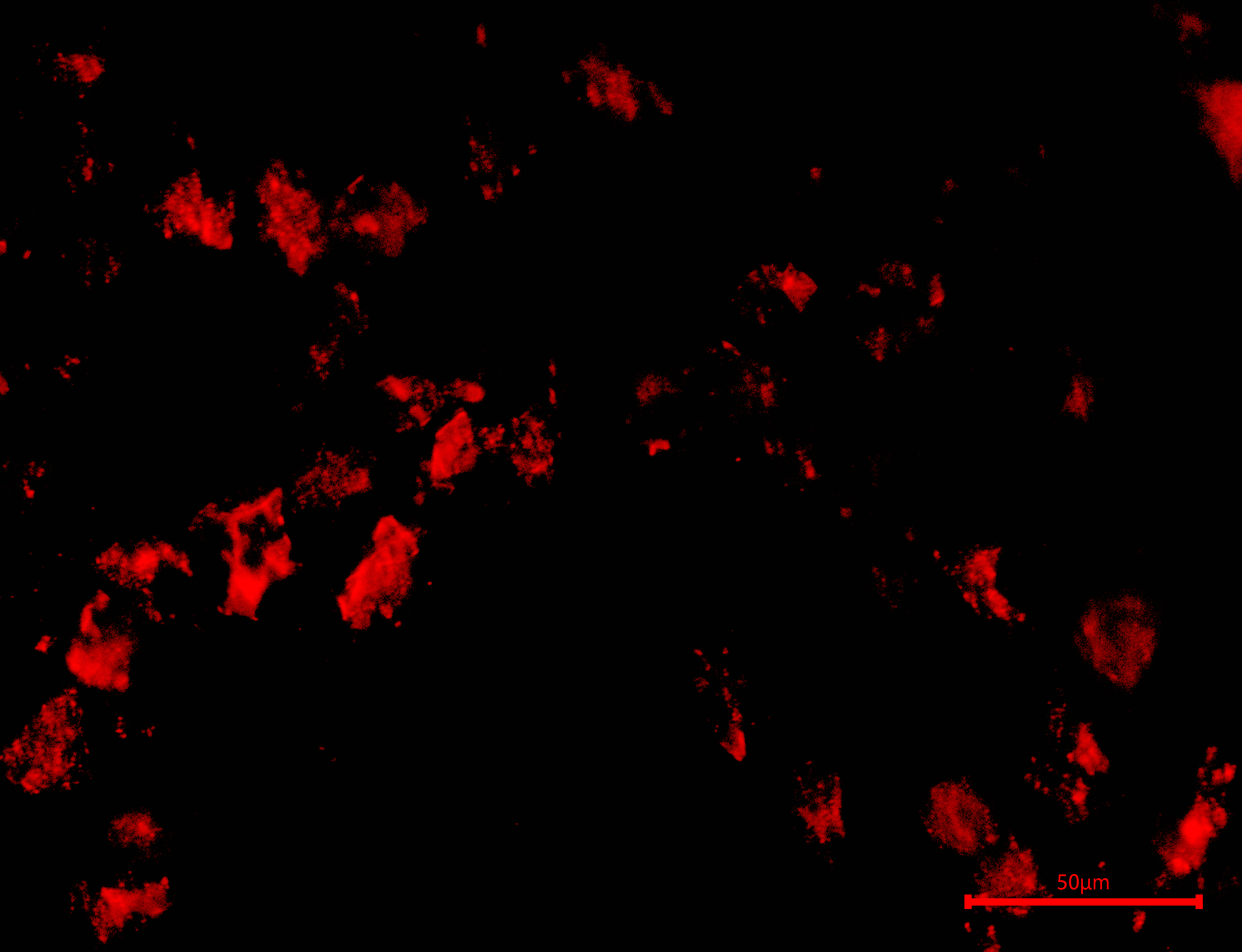

Supplement: Supplementary file 1 [file metabolites-16-00340-s001.zip › Figure S2 Uncropped microscopy images/Figure7/p62/3p62 PA+PQQ2.2.tif]

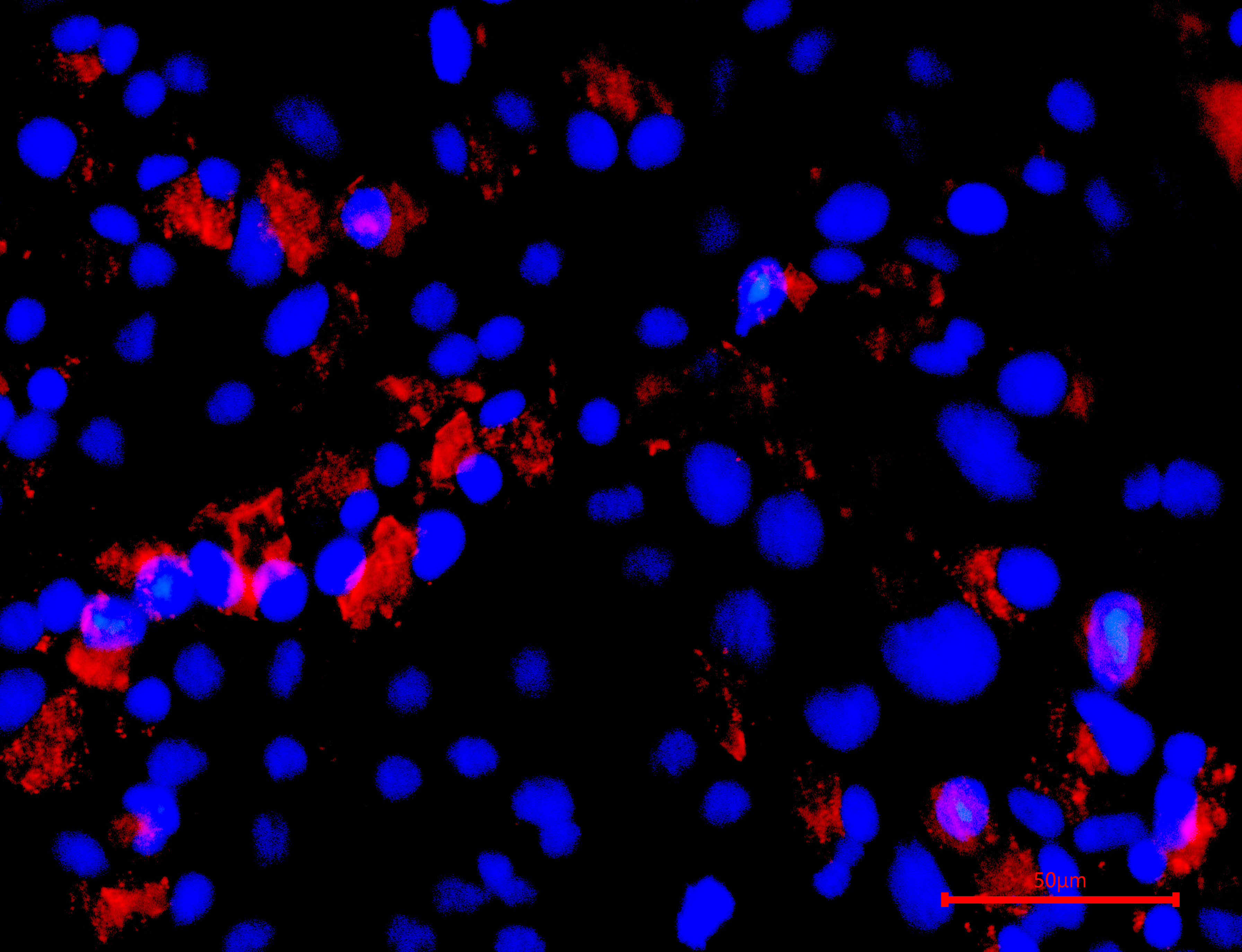

Supplement: Supplementary file 1 [file metabolites-16-00340-s001.zip › Figure S2 Uncropped microscopy images/Figure7/p62/3p62 PA+PQQ2.3Merge.tif]

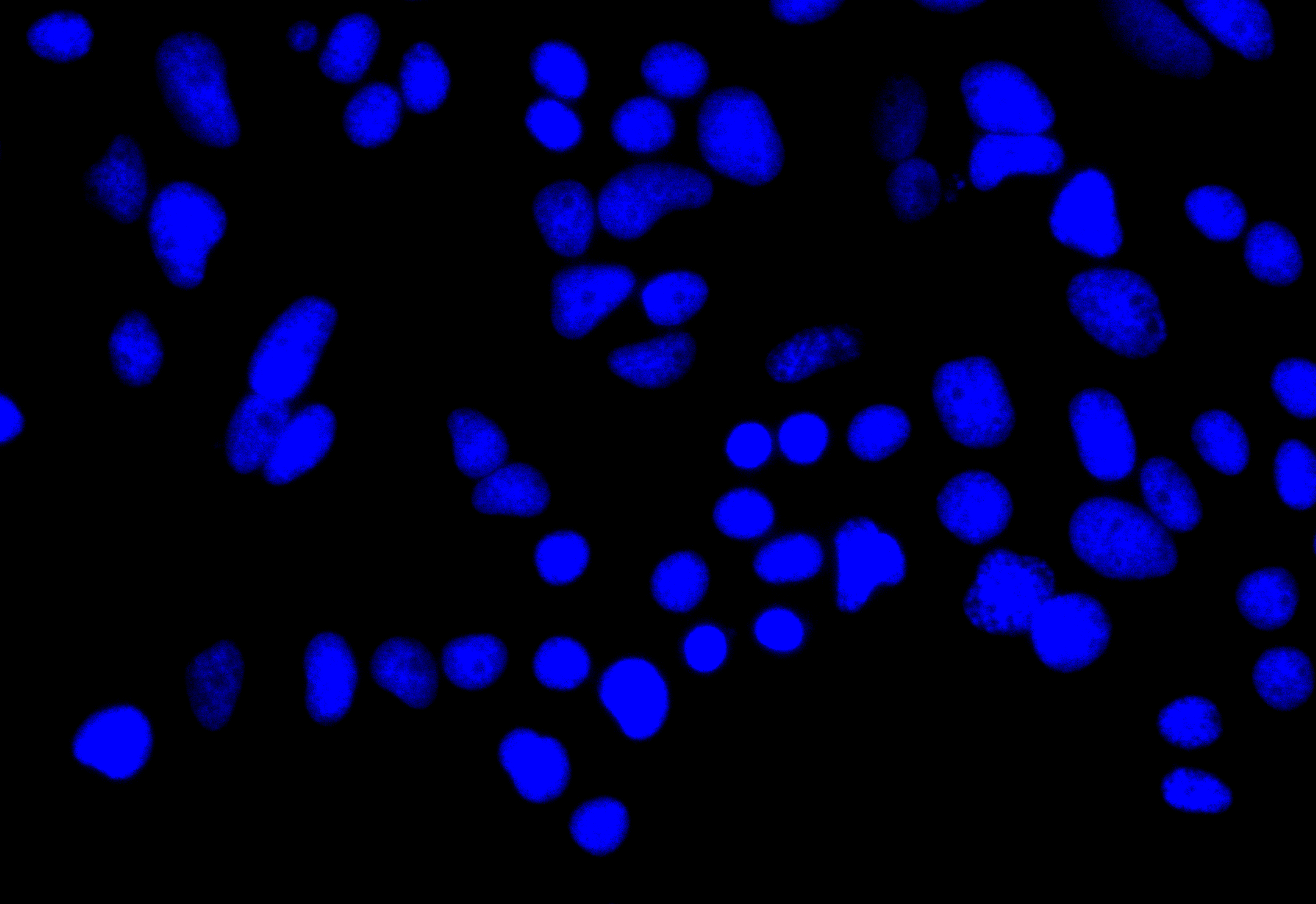

Supplement: Supplementary file 1 [file metabolites-16-00340-s001.zip › Figure S2 Uncropped microscopy images/Figure7/Parkin/1Parkin Ctl 2.1.png]

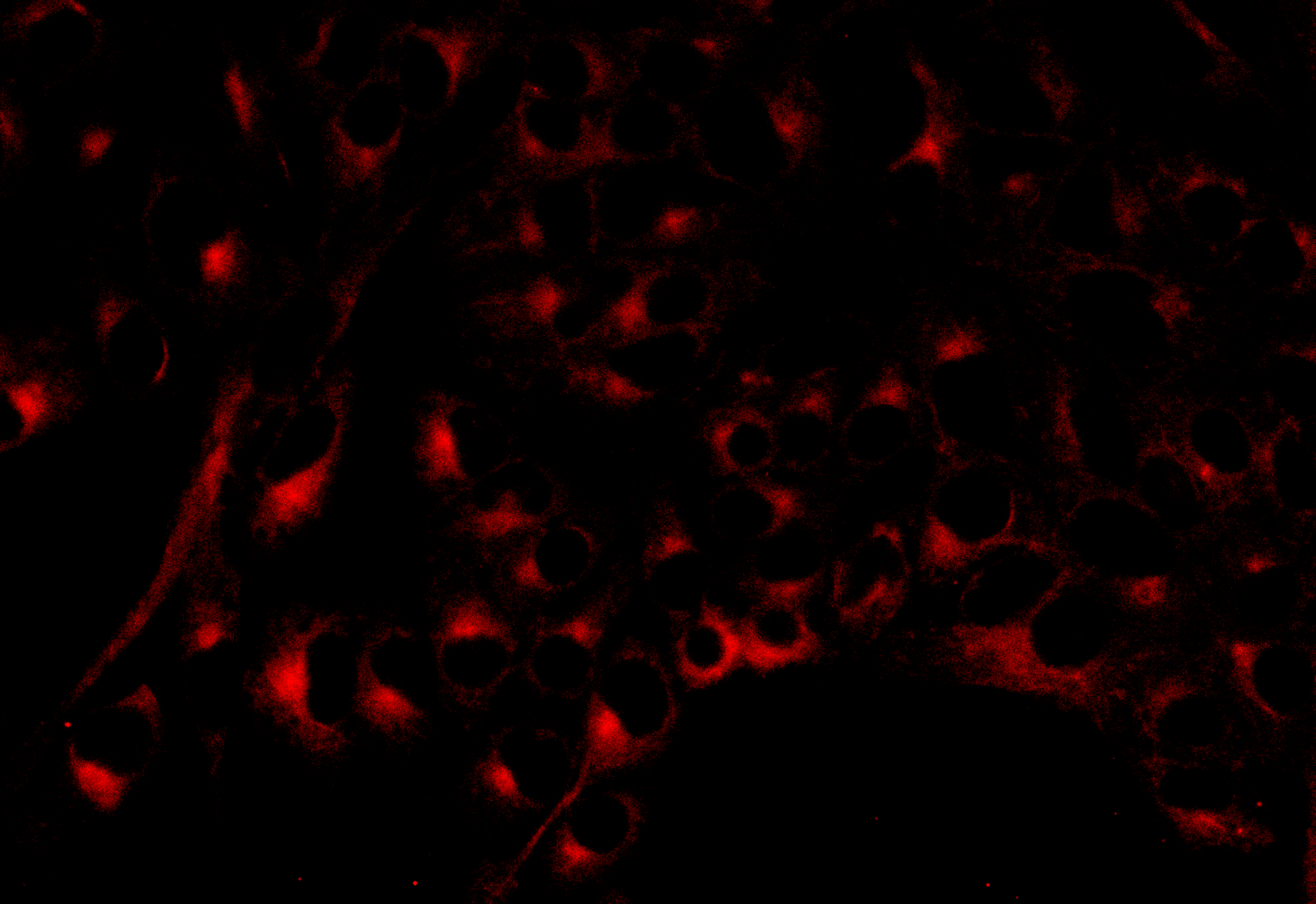

Supplement: Supplementary file 1 [file metabolites-16-00340-s001.zip › Figure S2 Uncropped microscopy images/Figure7/Parkin/1Parkin Ctl 2.2.png]

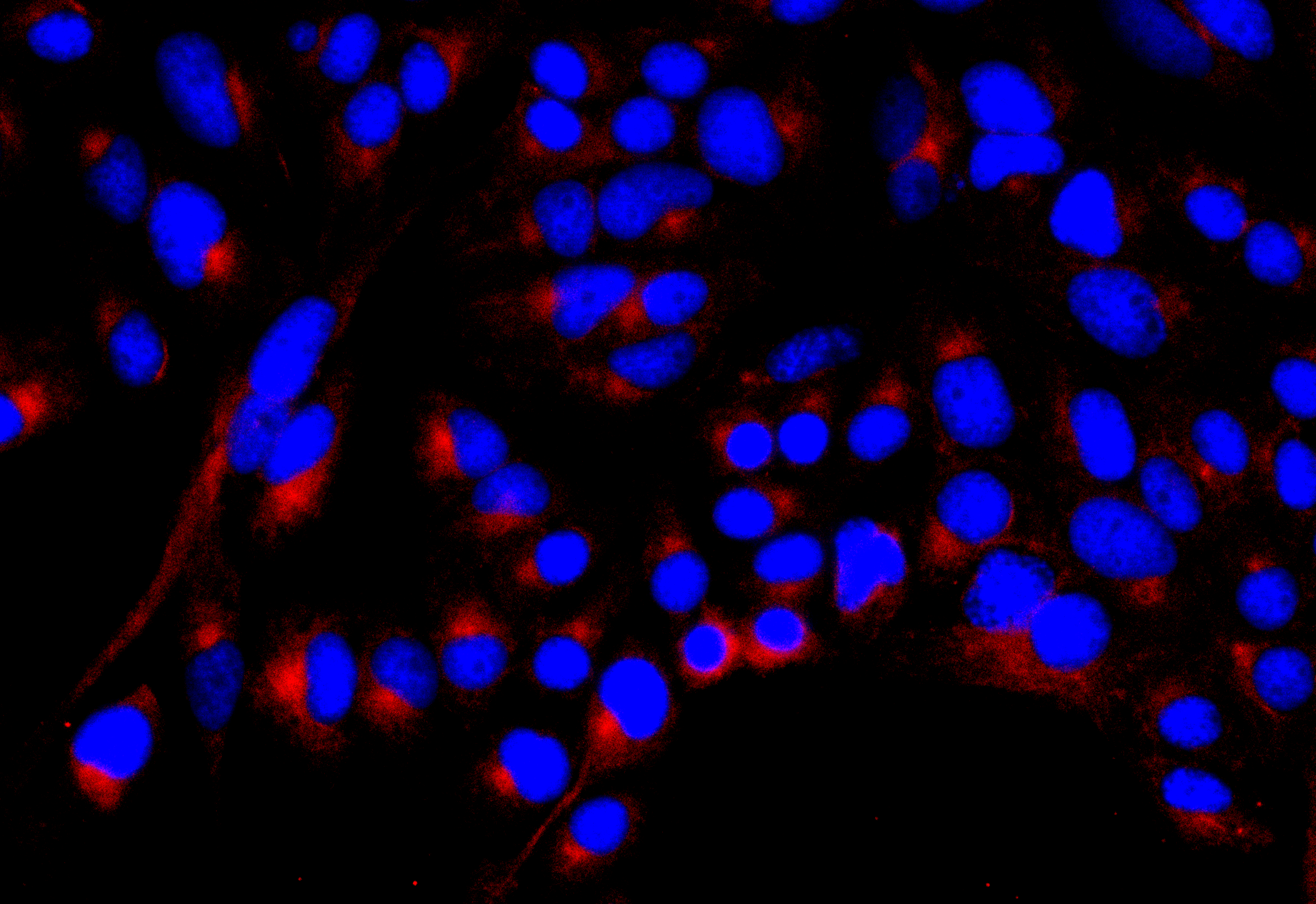

Supplement: Supplementary file 1 [file metabolites-16-00340-s001.zip › Figure S2 Uncropped microscopy images/Figure7/Parkin/1Parkin Ctl 2.3Merge.png]

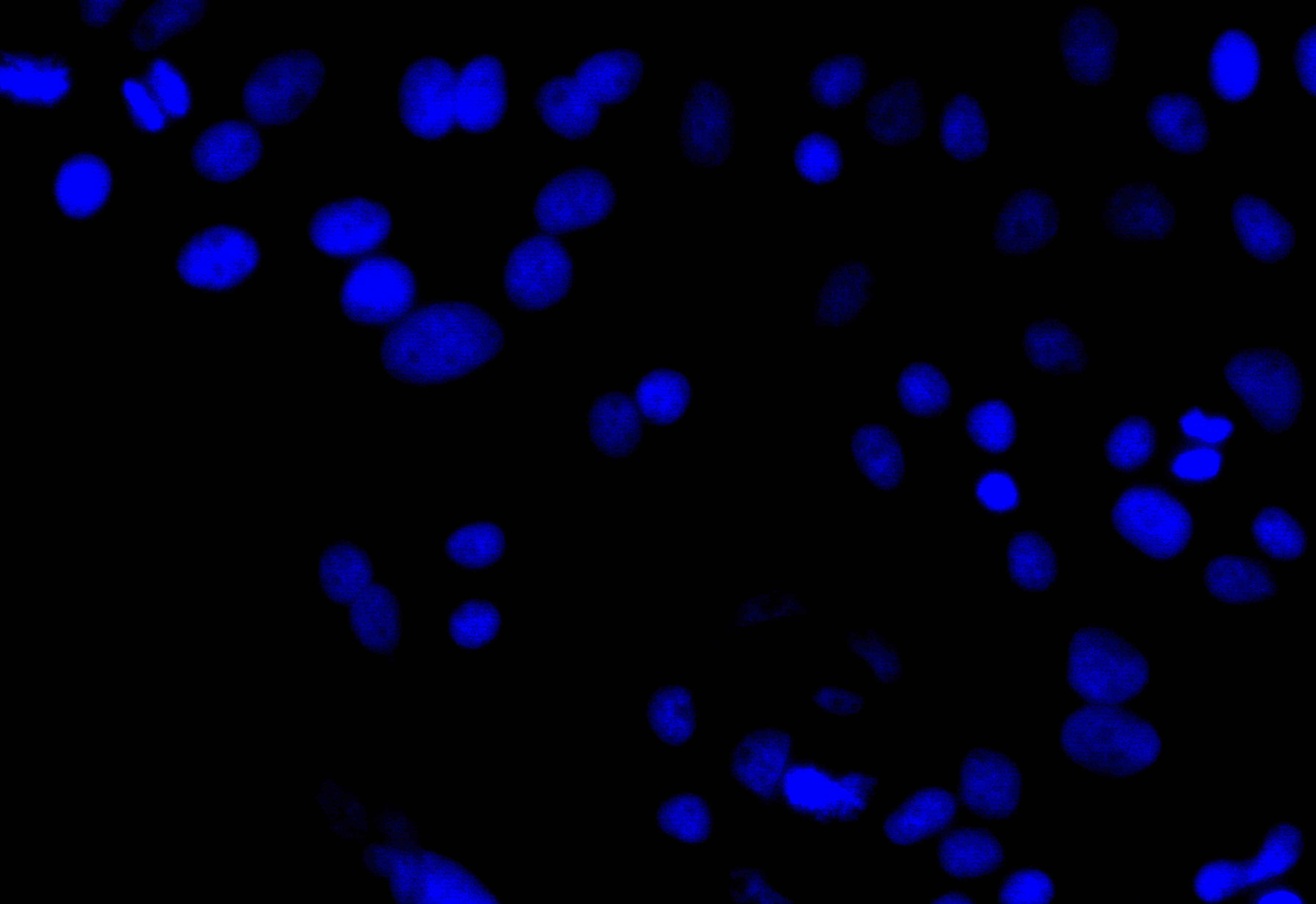

Supplement: Supplementary file 1 [file metabolites-16-00340-s001.zip › Figure S2 Uncropped microscopy images/Figure7/Parkin/2Parkin PA3.1.png]

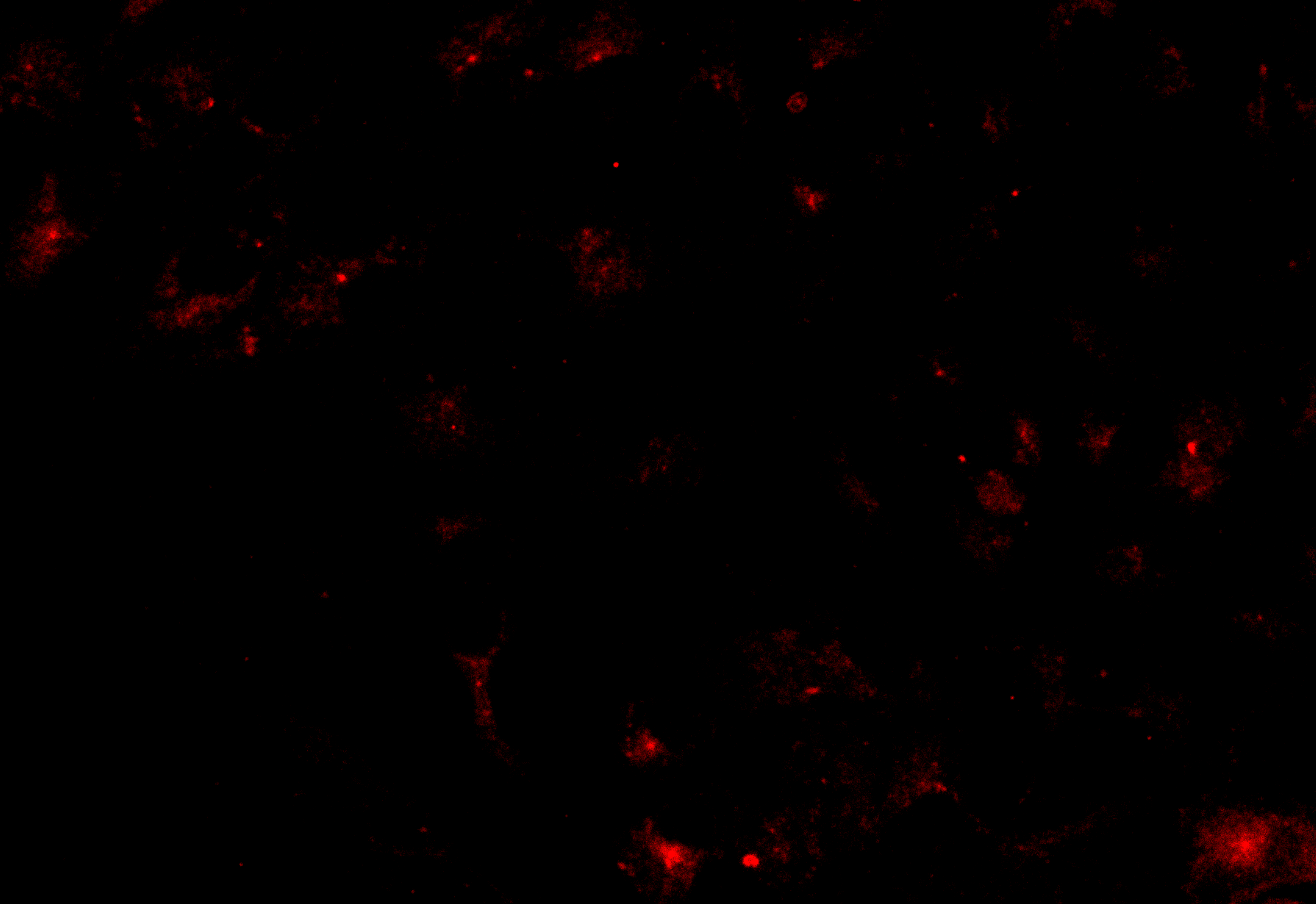

Supplement: Supplementary file 1 [file metabolites-16-00340-s001.zip › Figure S2 Uncropped microscopy images/Figure7/Parkin/2Parkin PA3.2.png]

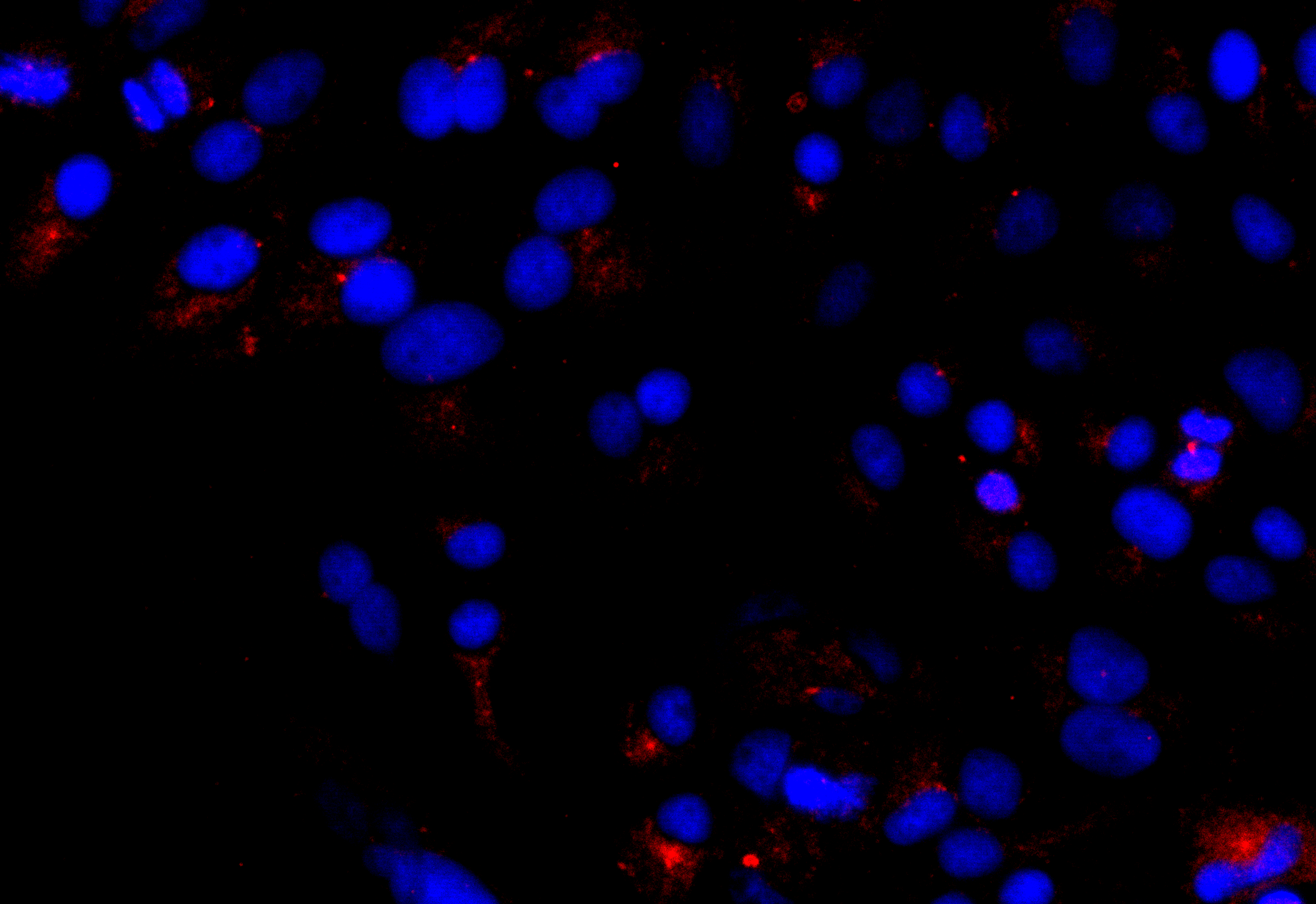

Supplement: Supplementary file 1 [file metabolites-16-00340-s001.zip › Figure S2 Uncropped microscopy images/Figure7/Parkin/2Parkin PA3.3Merge.png]

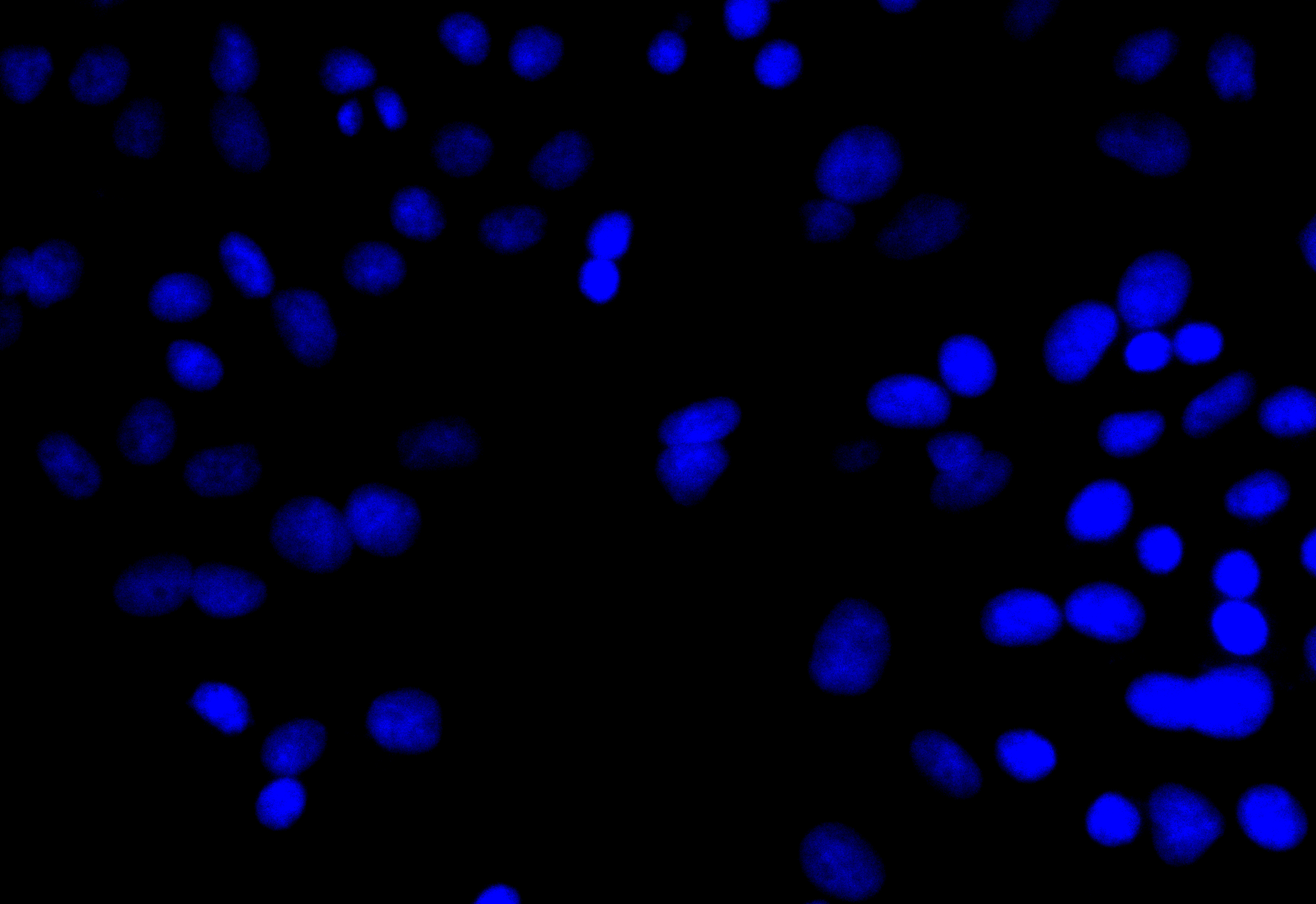

Supplement: Supplementary file 1 [file metabolites-16-00340-s001.zip › Figure S2 Uncropped microscopy images/Figure7/Parkin/3Parkin PQQ1.1.png]

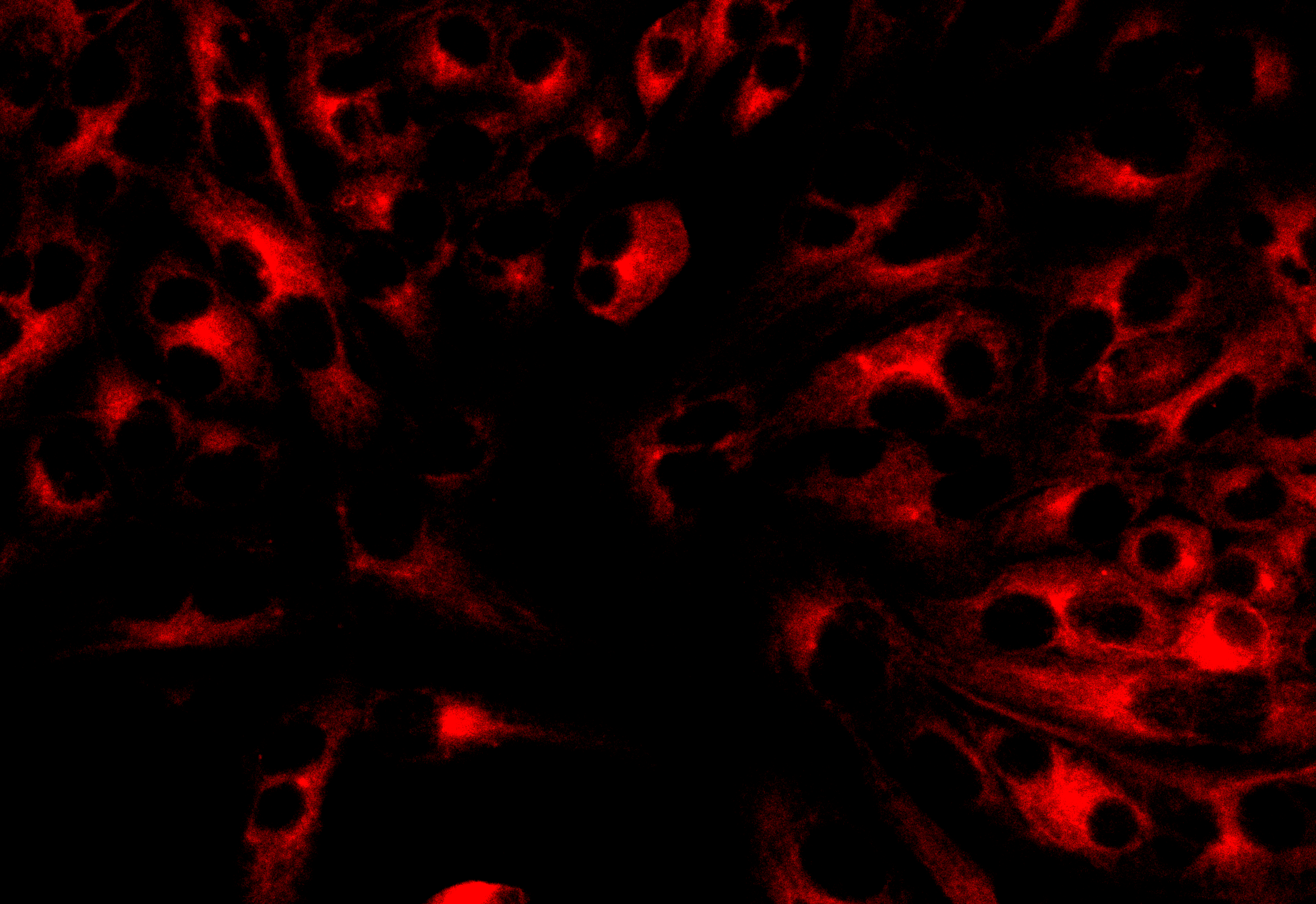

Supplement: Supplementary file 1 [file metabolites-16-00340-s001.zip › Figure S2 Uncropped microscopy images/Figure7/Parkin/3Parkin PQQ1.2.png]

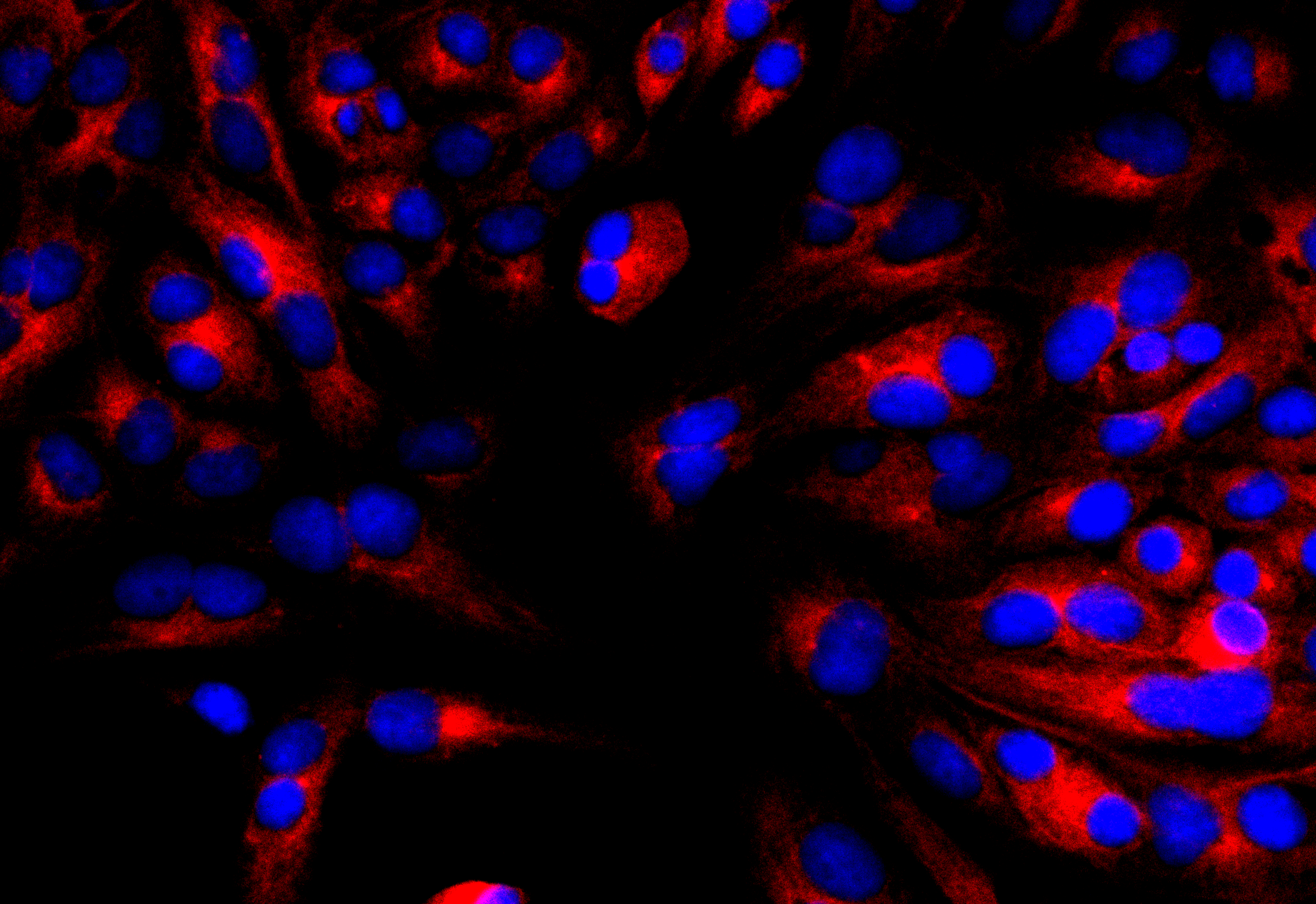

Supplement: Supplementary file 1 [file metabolites-16-00340-s001.zip › Figure S2 Uncropped microscopy images/Figure7/Parkin/3Parkin PQQ1.3.png]

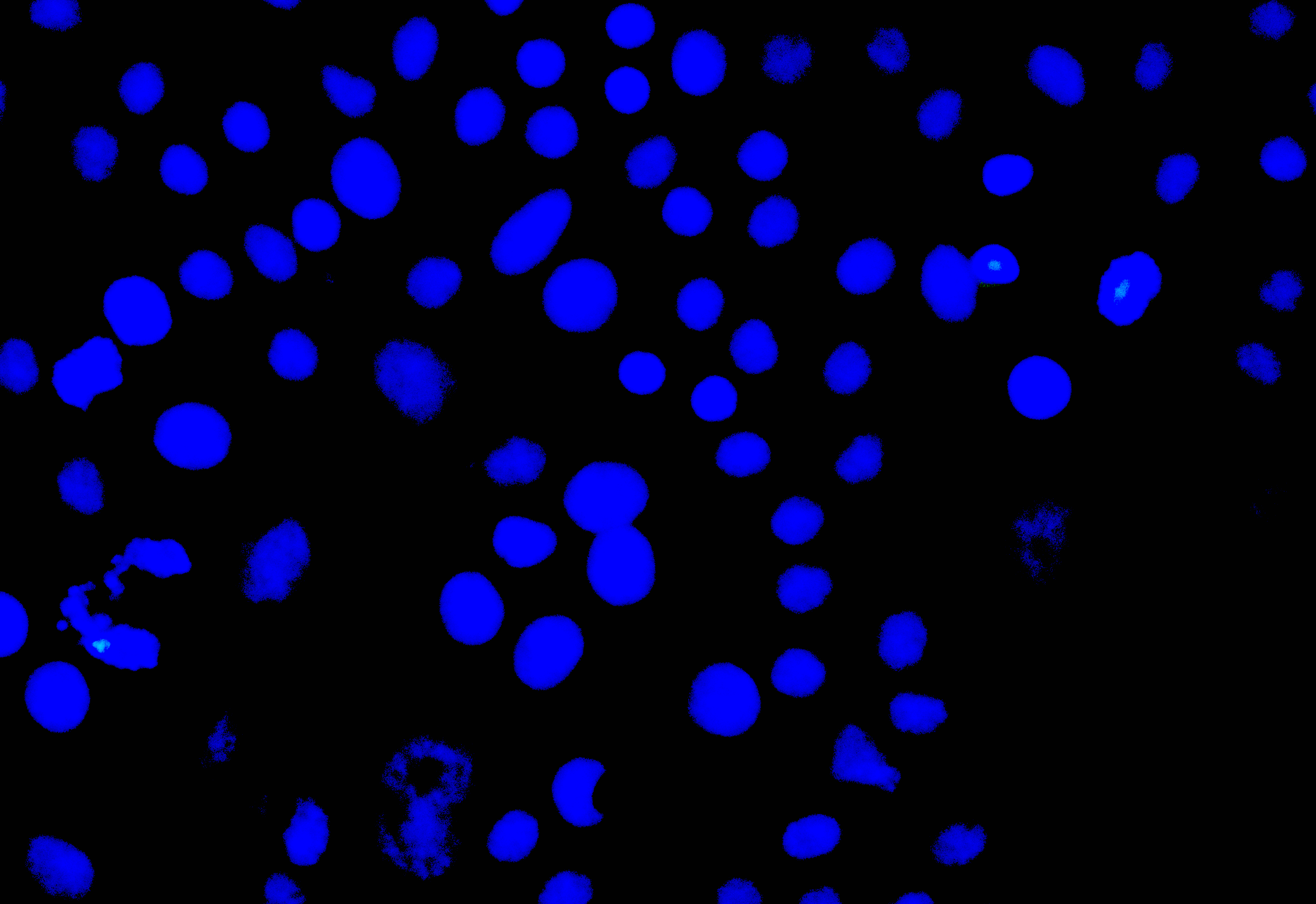

Supplement: Supplementary file 1 [file metabolites-16-00340-s001.zip › Figure S2 Uncropped microscopy images/Figure7/PINK1/PINK1 CTL2.1.png]

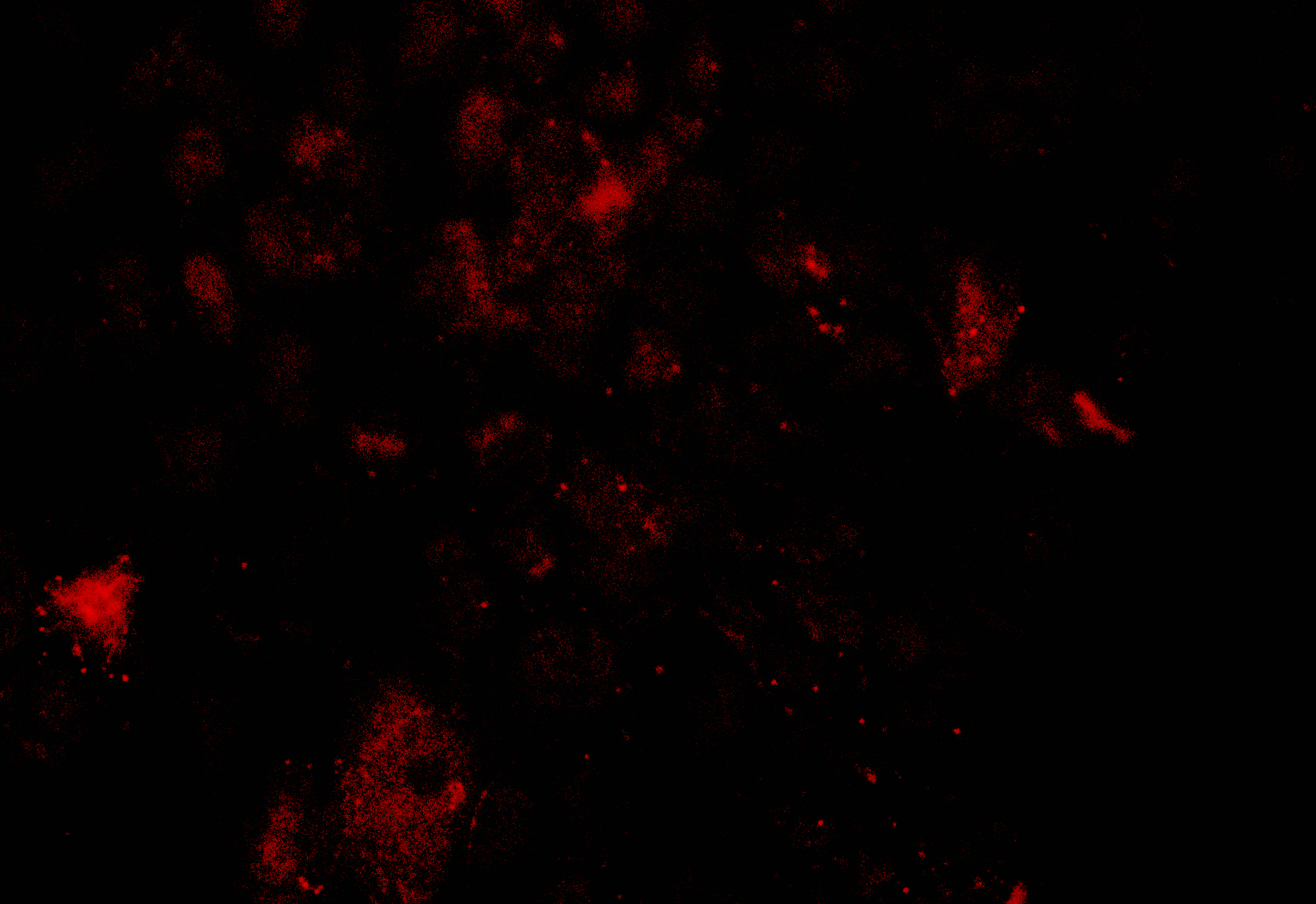

Supplement: Supplementary file 1 [file metabolites-16-00340-s001.zip › Figure S2 Uncropped microscopy images/Figure7/PINK1/PINK1 CTL2.2.png]

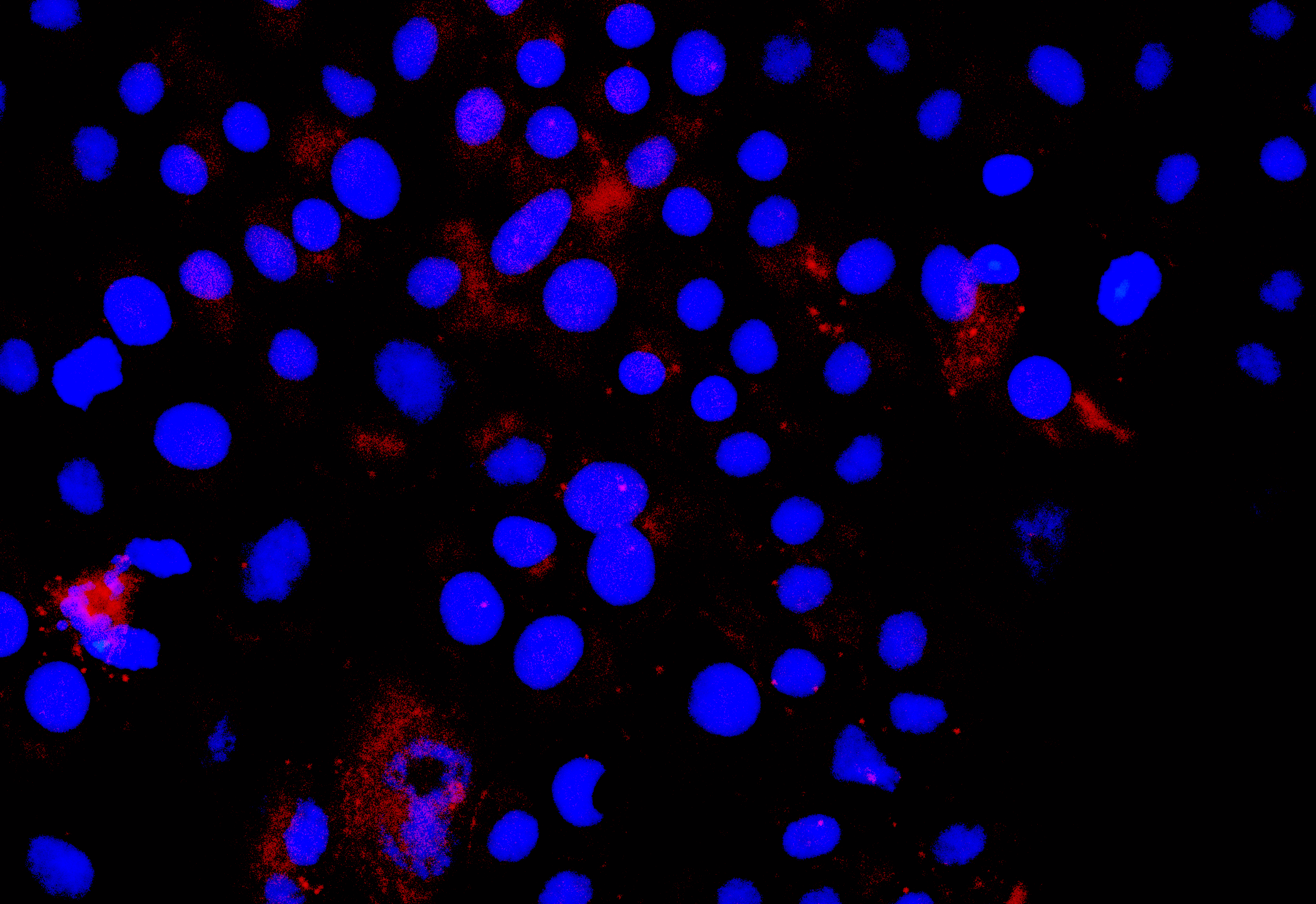

Supplement: Supplementary file 1 [file metabolites-16-00340-s001.zip › Figure S2 Uncropped microscopy images/Figure7/PINK1/PINK1 CTL2.3Merge.png]

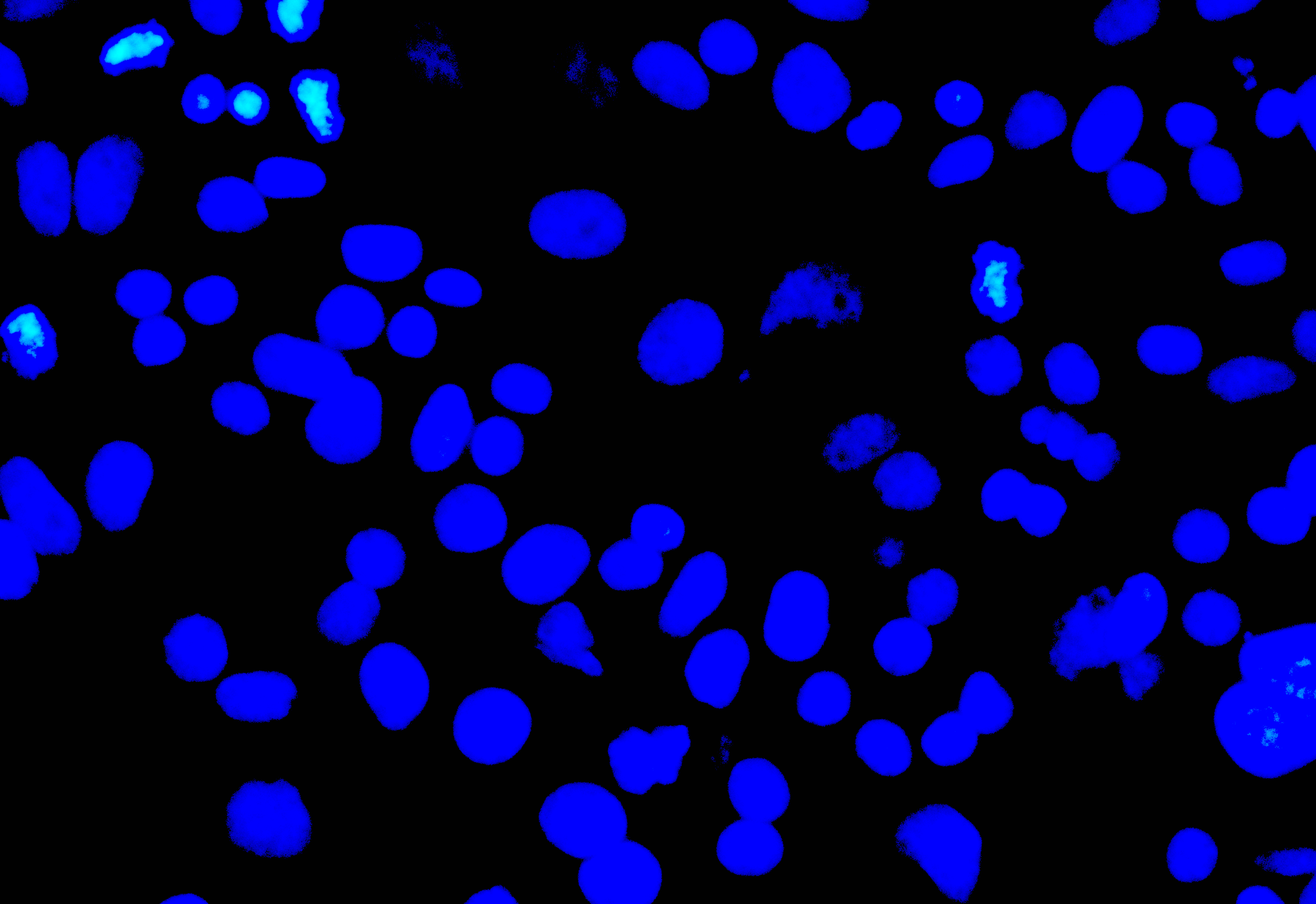

Supplement: Supplementary file 1 [file metabolites-16-00340-s001.zip › Figure S2 Uncropped microscopy images/Figure7/PINK1/PINK1 PA2.1.png]

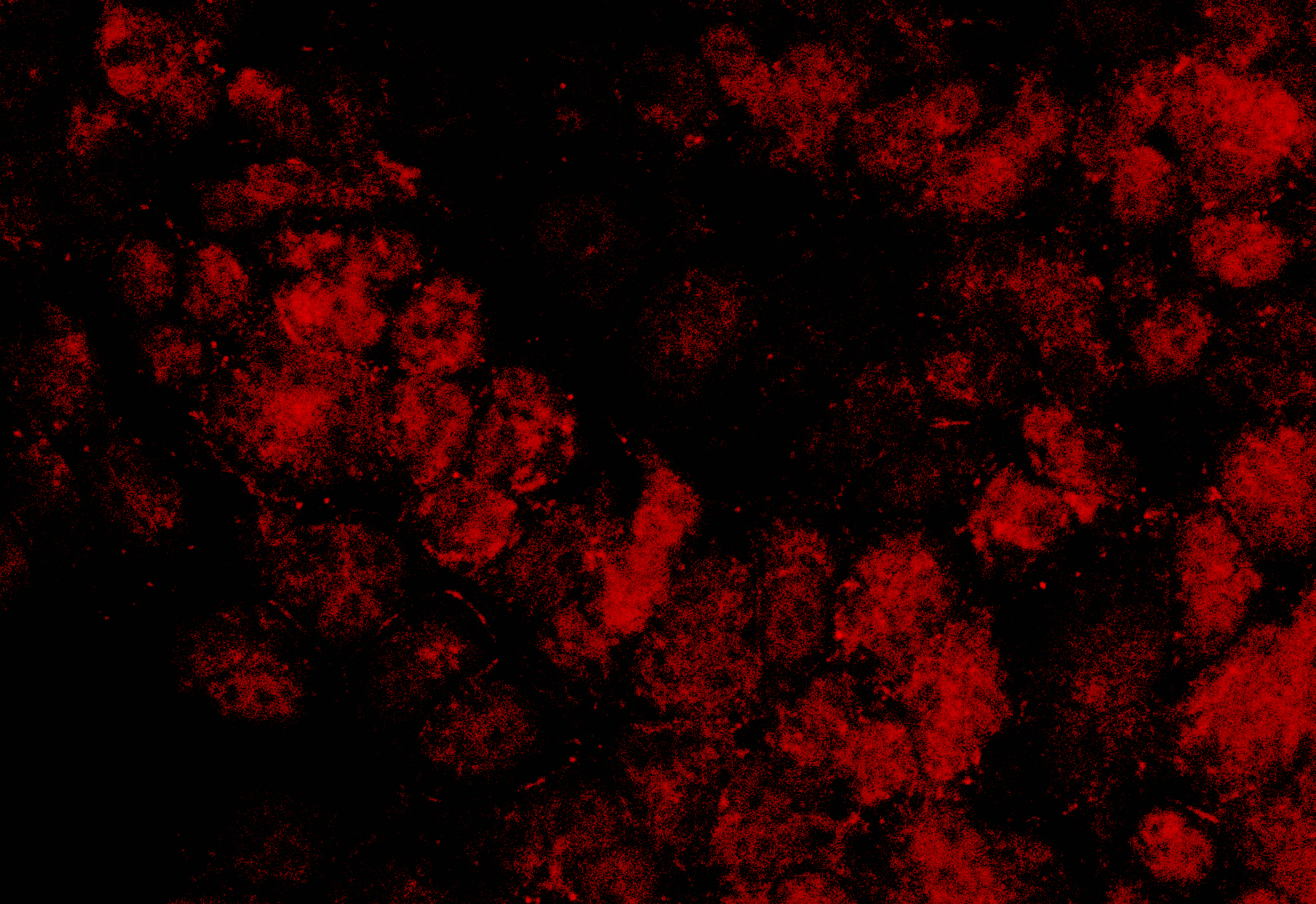

Supplement: Supplementary file 1 [file metabolites-16-00340-s001.zip › Figure S2 Uncropped microscopy images/Figure7/PINK1/PINK1 PA2.2.png]

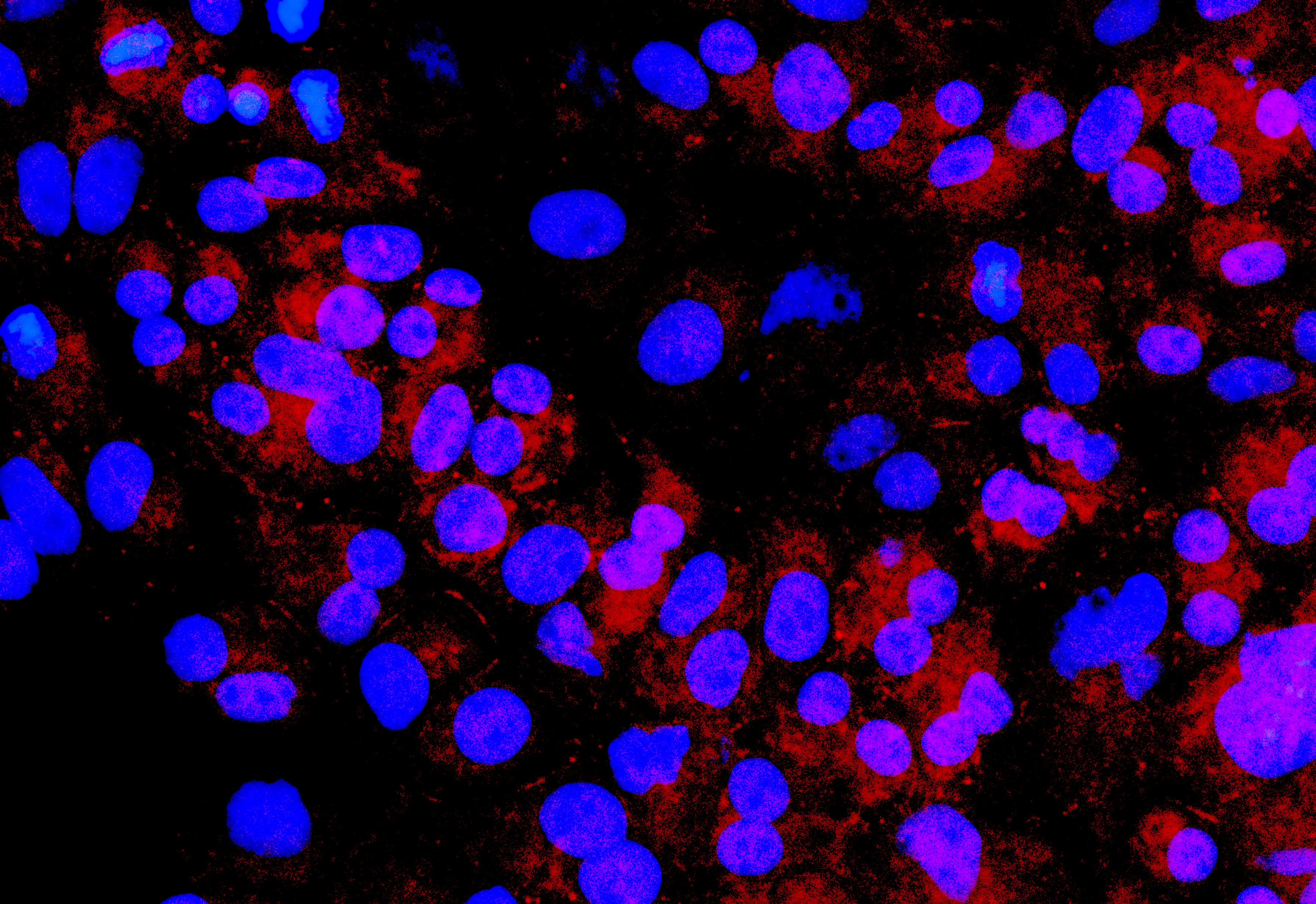

Supplement: Supplementary file 1 [file metabolites-16-00340-s001.zip › Figure S2 Uncropped microscopy images/Figure7/PINK1/PINK1 PA2.3Merge.png]

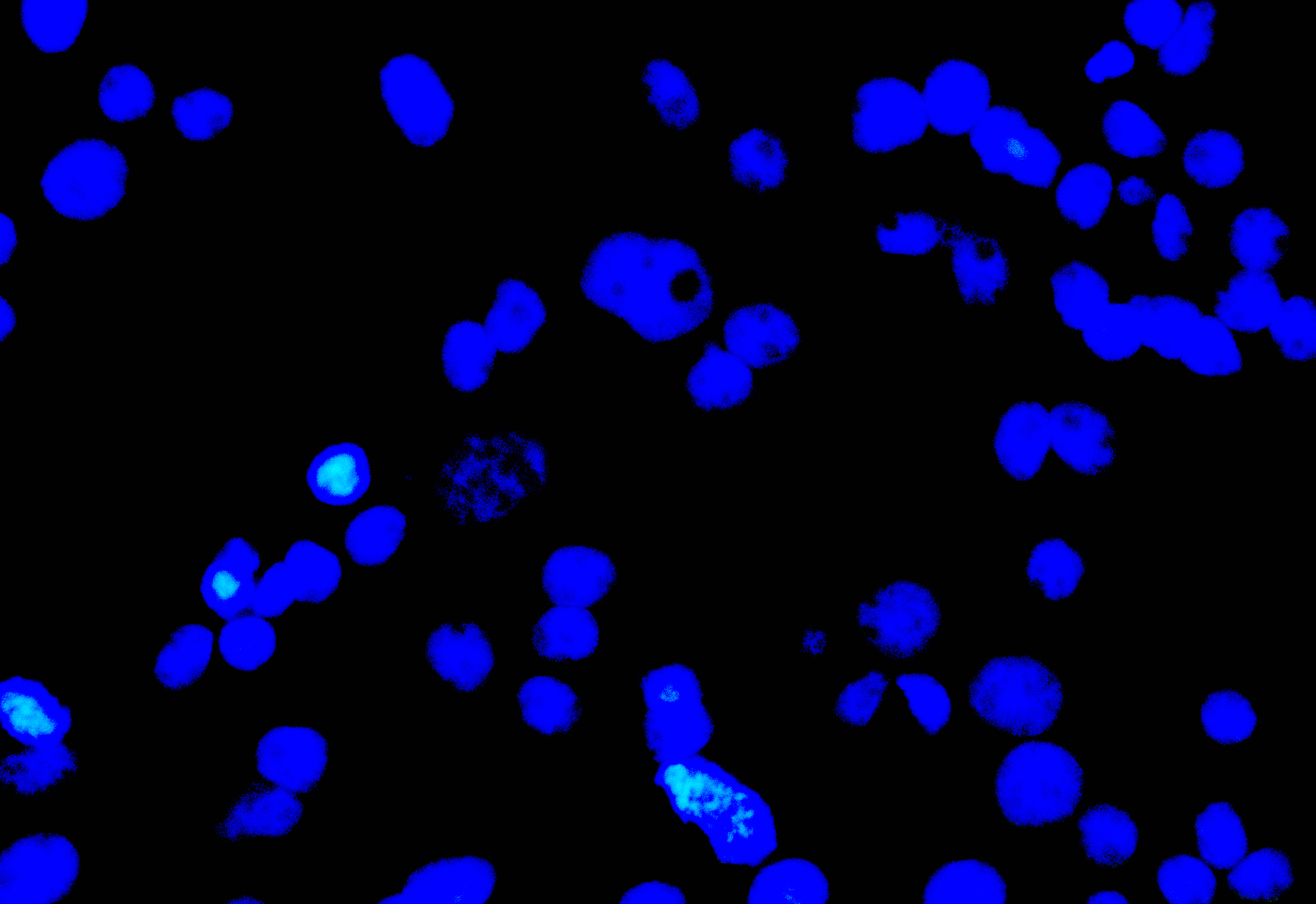

Supplement: Supplementary file 1 [file metabolites-16-00340-s001.zip › Figure S2 Uncropped microscopy images/Figure7/PINK1/PINK1 PQQ1.1.png]

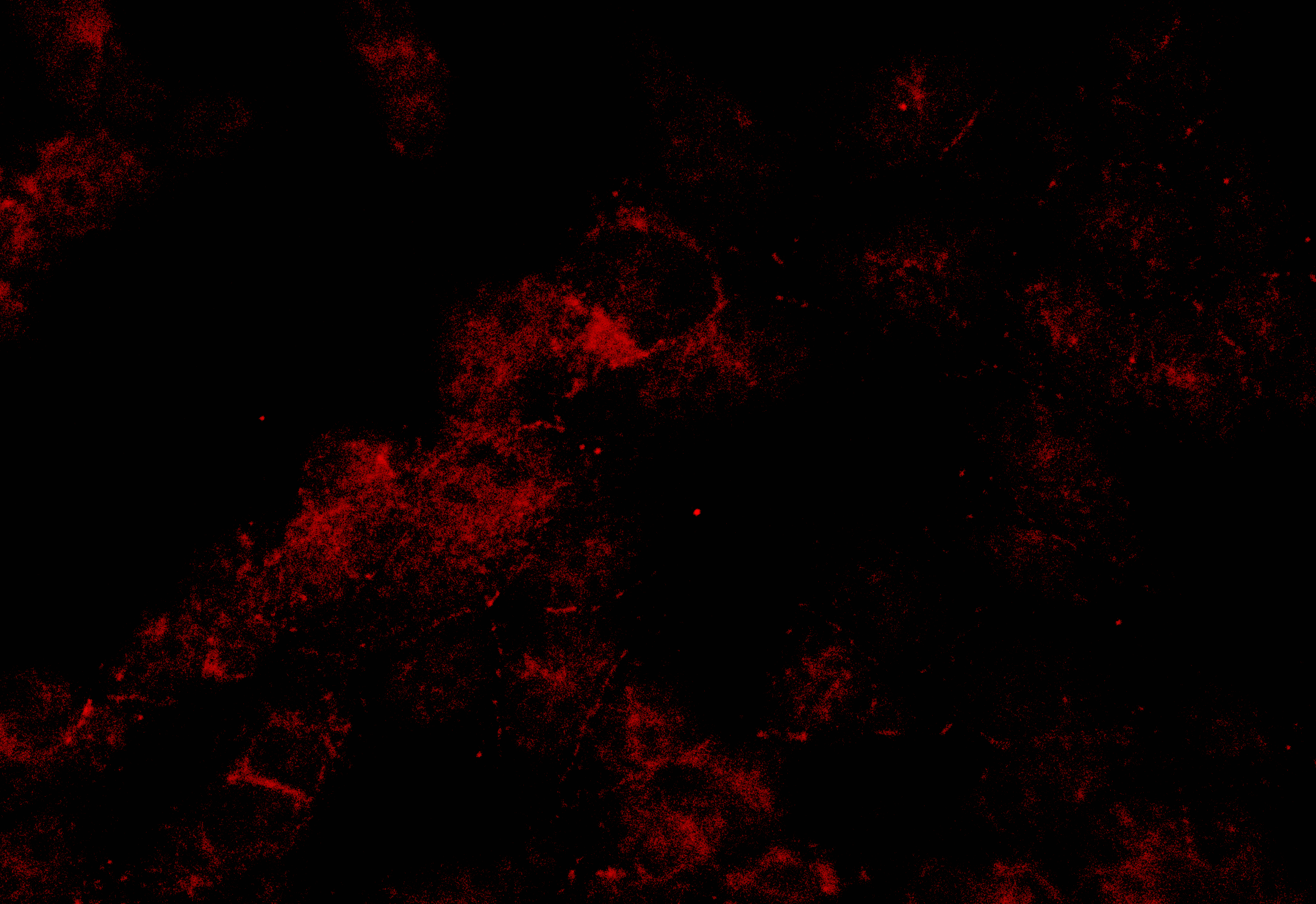

Supplement: Supplementary file 1 [file metabolites-16-00340-s001.zip › Figure S2 Uncropped microscopy images/Figure7/PINK1/PINK1 PQQ1.2.png]

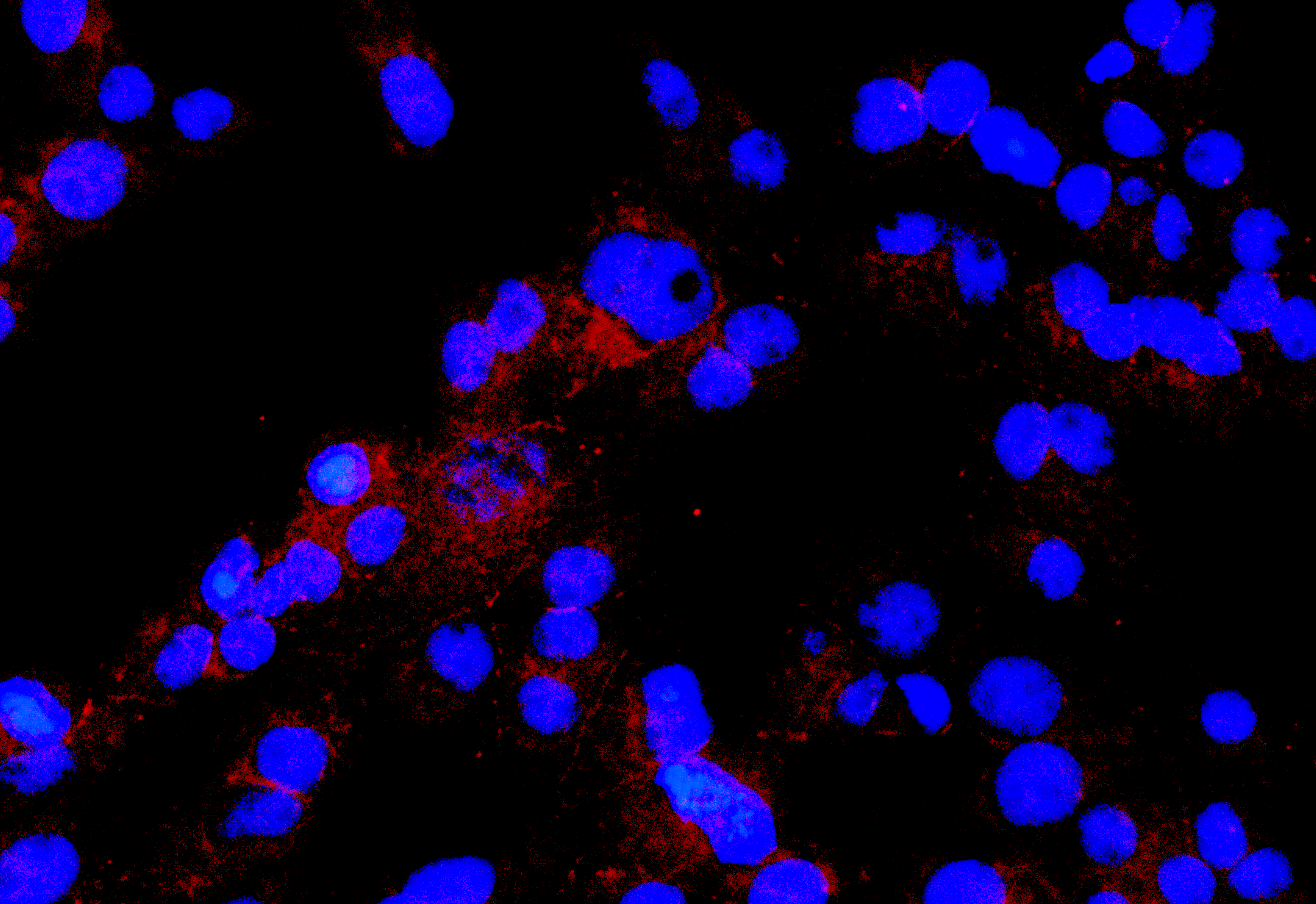

Supplement: Supplementary file 1 [file metabolites-16-00340-s001.zip › Figure S2 Uncropped microscopy images/Figure7/PINK1/PINK1 PQQ1.3Merge.png]

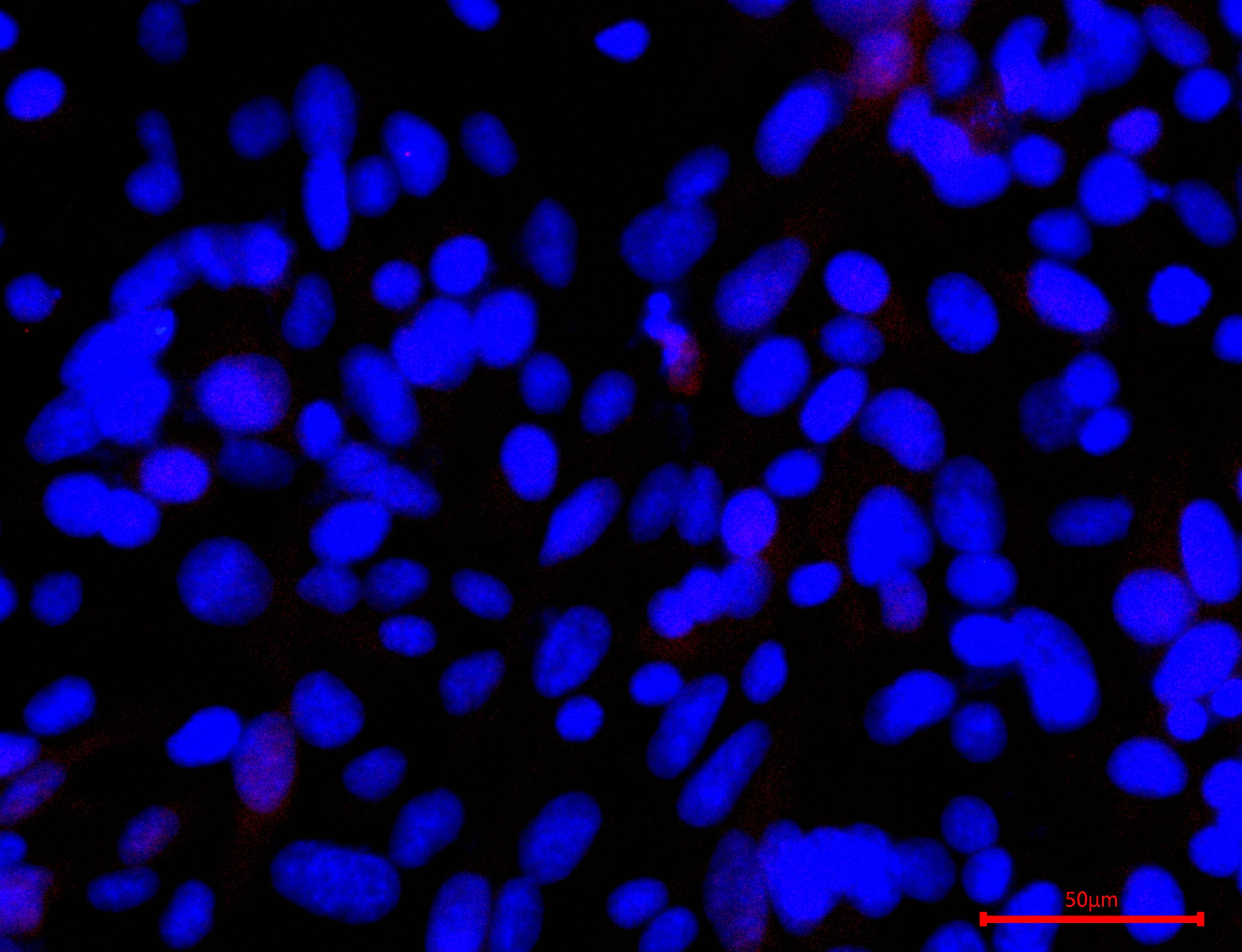

Supplement: Supplementary file 1 [file metabolites-16-00340-s001.zip › Figure S2 Uncropped microscopy images/Figure8/ASC/CTLmerge2(1).jpg]

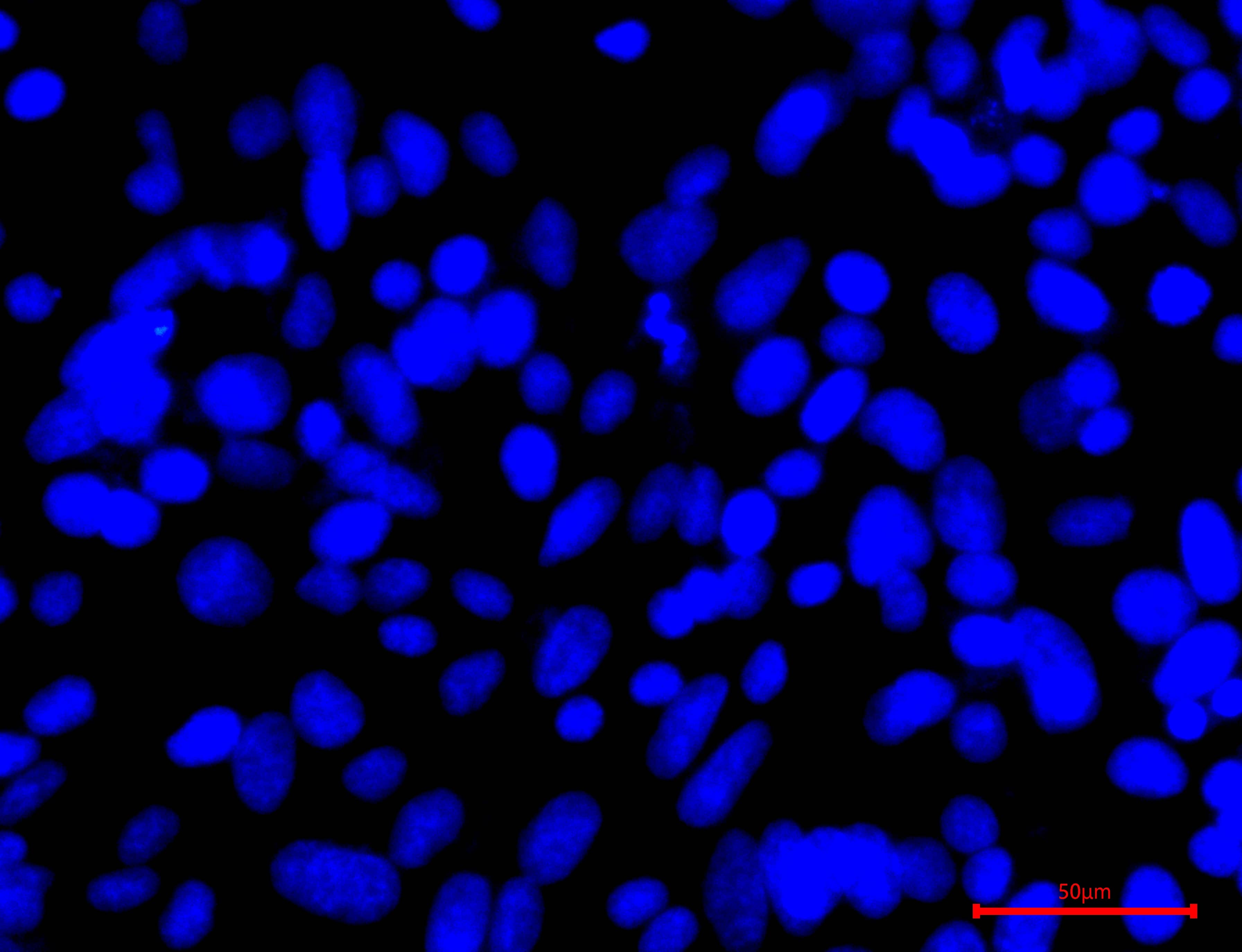

Supplement: Supplementary file 1 [file metabolites-16-00340-s001.zip › Figure S2 Uncropped microscopy images/Figure8/ASC/CTL核2(1).jpg]

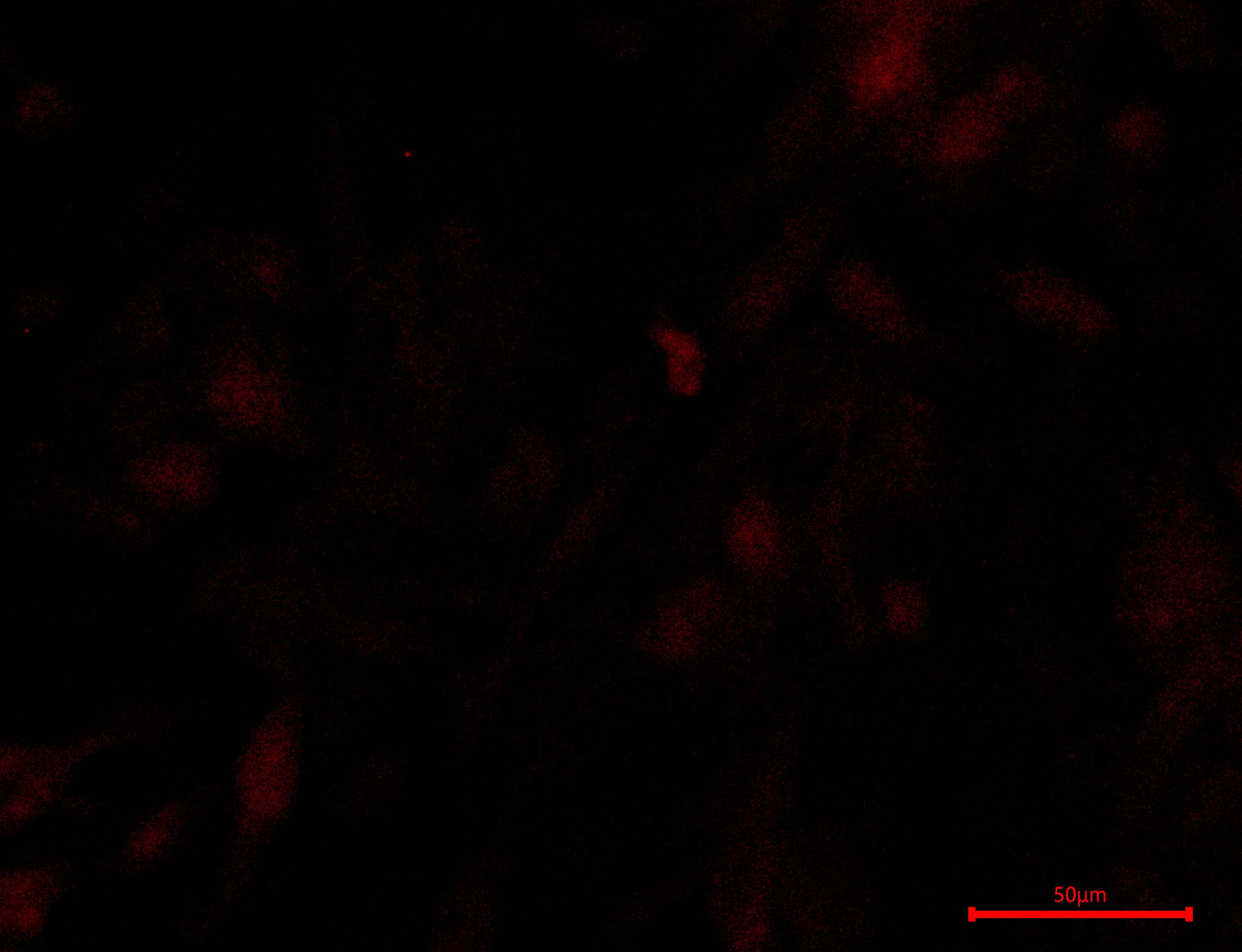

Supplement: Supplementary file 1 [file metabolites-16-00340-s001.zip › Figure S2 Uncropped microscopy images/Figure8/ASC/CTL红2(1).jpg]

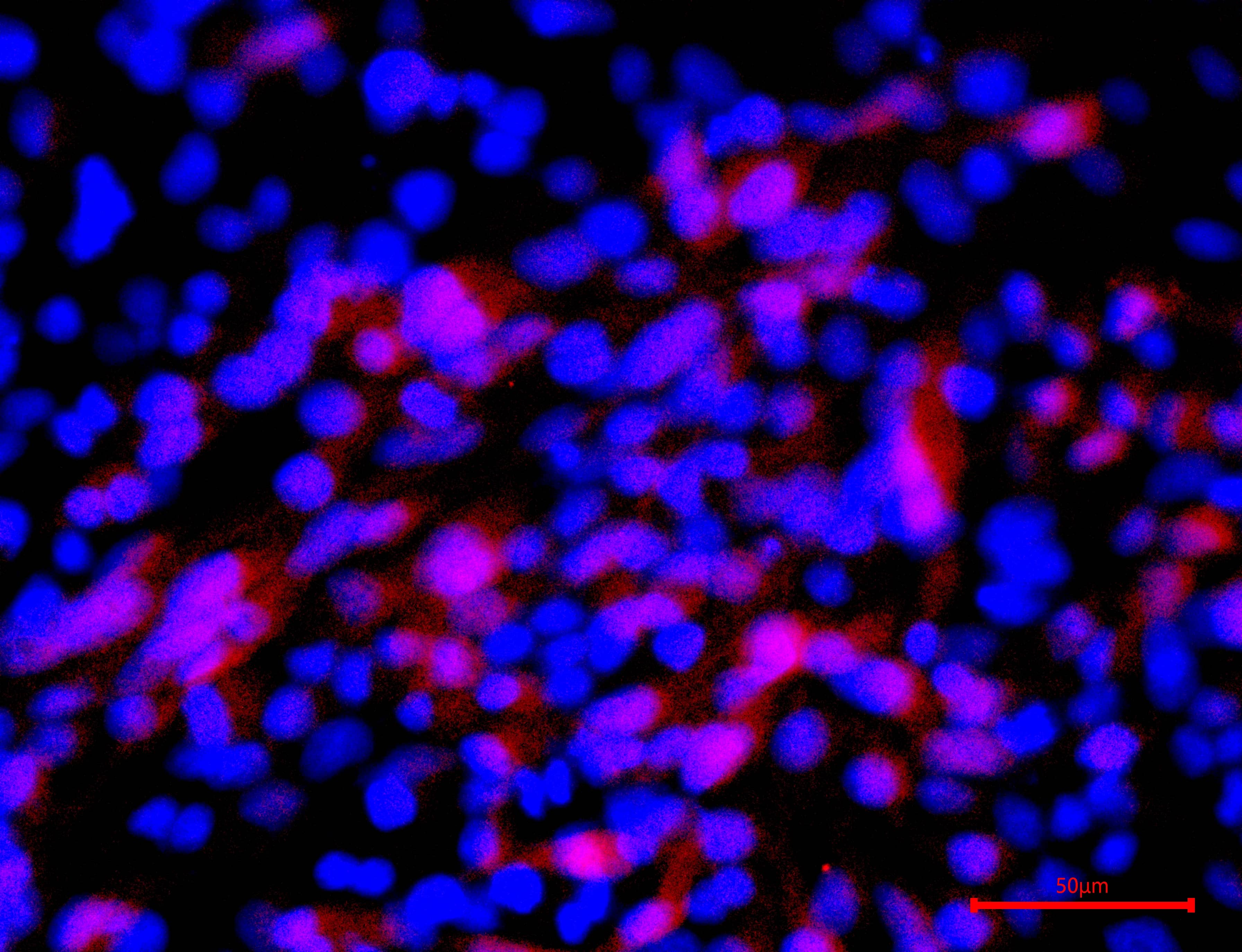

Supplement: Supplementary file 1 [file metabolites-16-00340-s001.zip › Figure S2 Uncropped microscopy images/Figure8/ASC/Nmerge3(1).jpg]

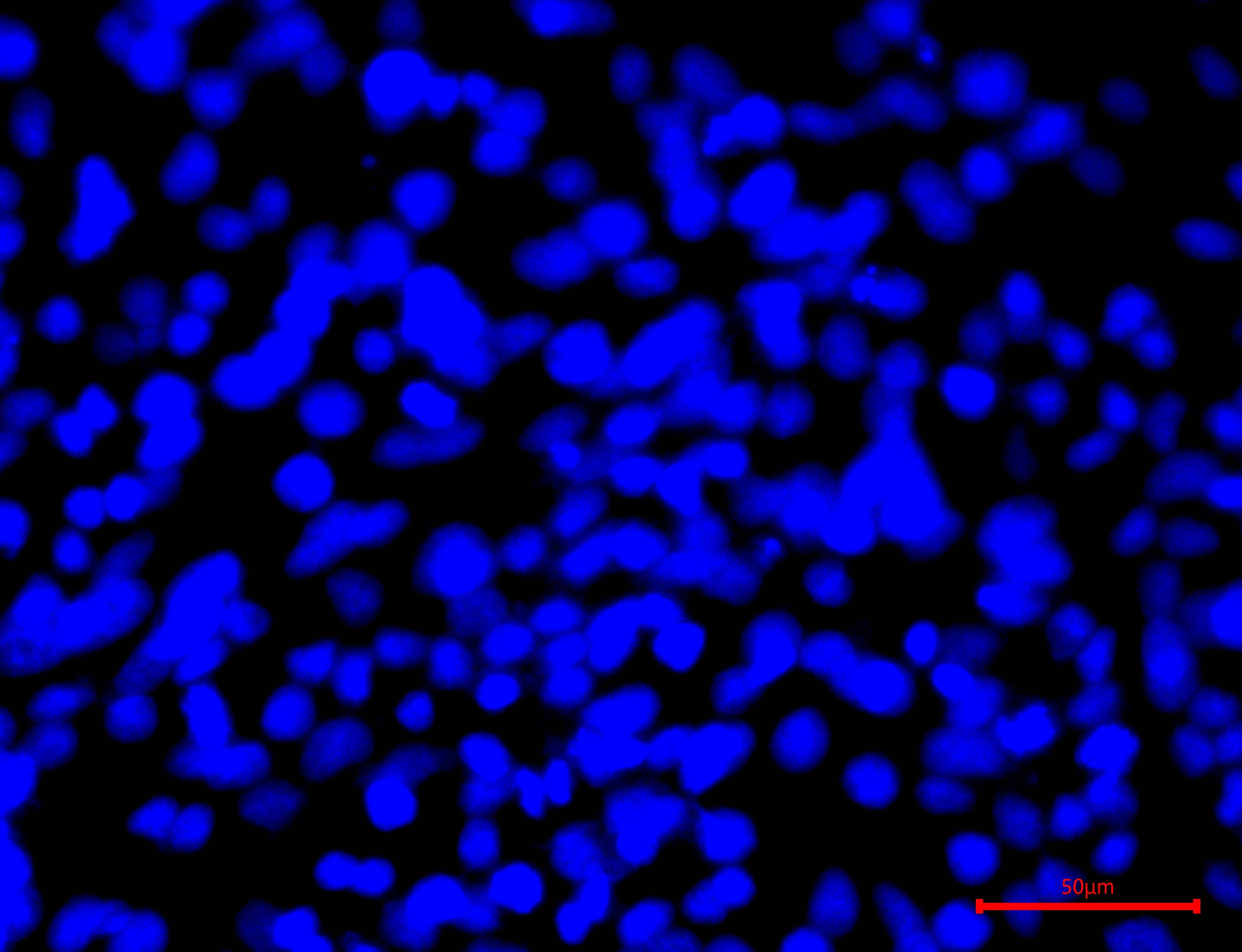

Supplement: Supplementary file 1 [file metabolites-16-00340-s001.zip › Figure S2 Uncropped microscopy images/Figure8/ASC/N核3(1).jpg]

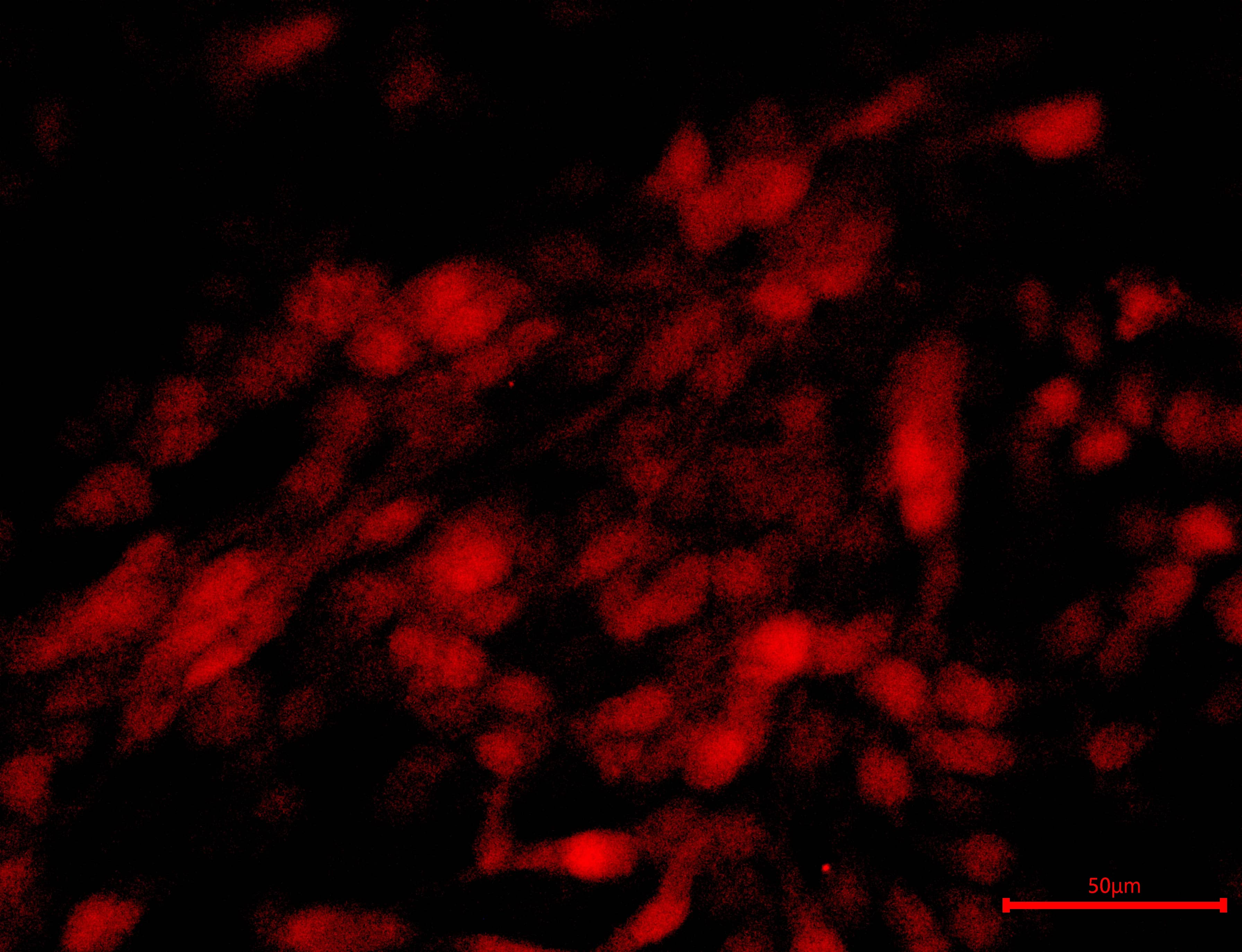

Supplement: Supplementary file 1 [file metabolites-16-00340-s001.zip › Figure S2 Uncropped microscopy images/Figure8/ASC/N红3(1).jpg]

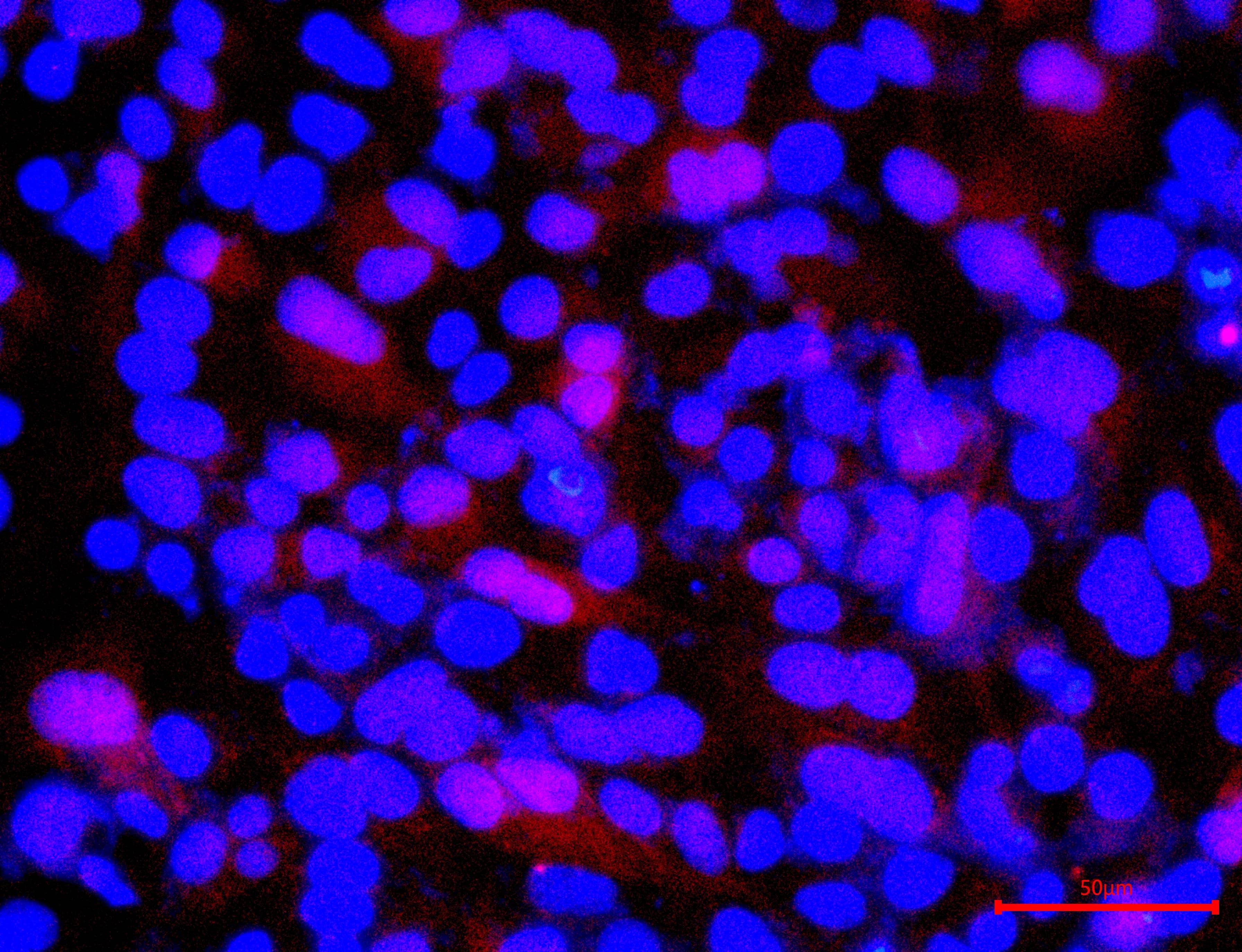

Supplement: Supplementary file 1 [file metabolites-16-00340-s001.zip › Figure S2 Uncropped microscopy images/Figure8/ASC/PAmerge4(1).jpg]

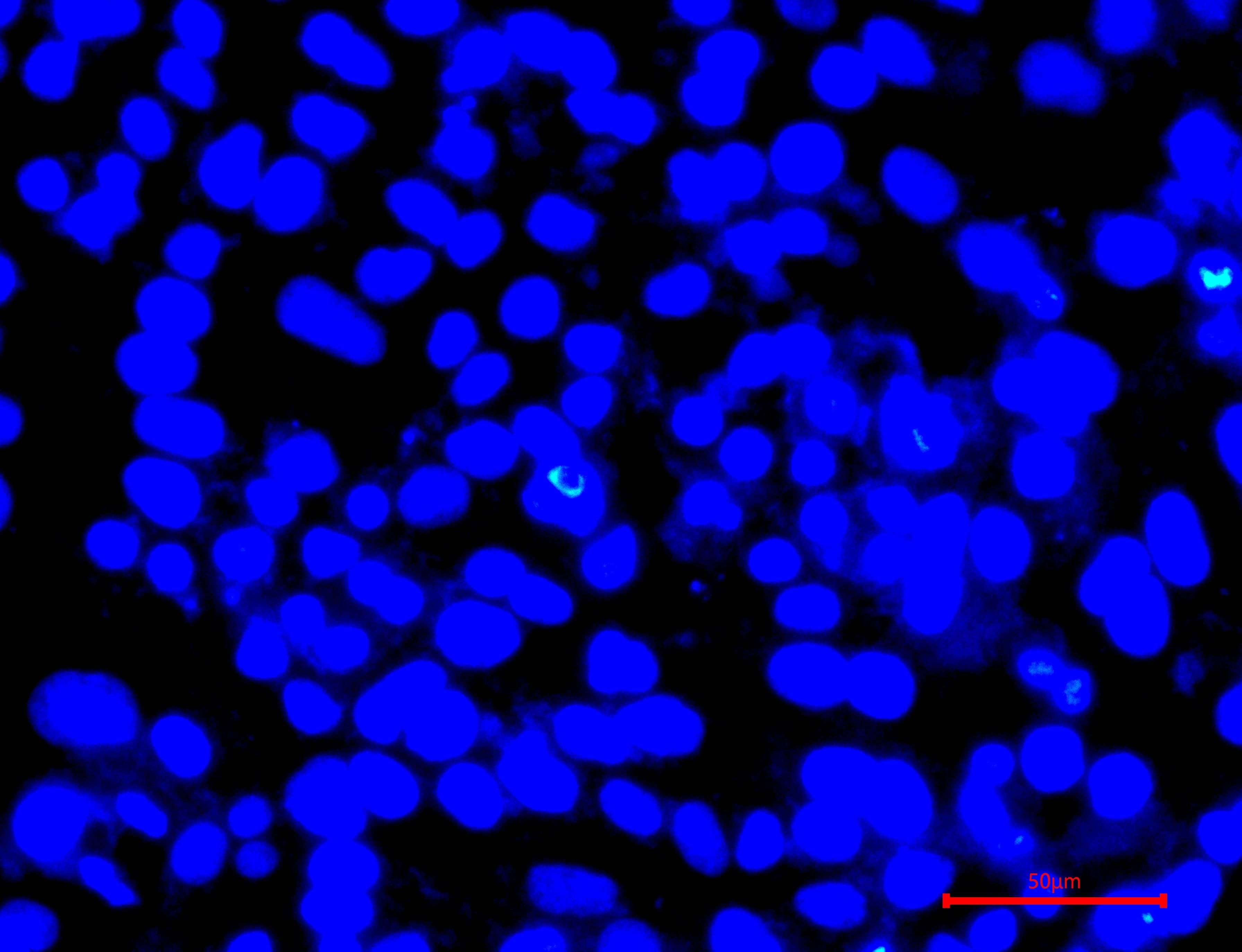

Supplement: Supplementary file 1 [file metabolites-16-00340-s001.zip › Figure S2 Uncropped microscopy images/Figure8/ASC/PA核4(1).jpg]

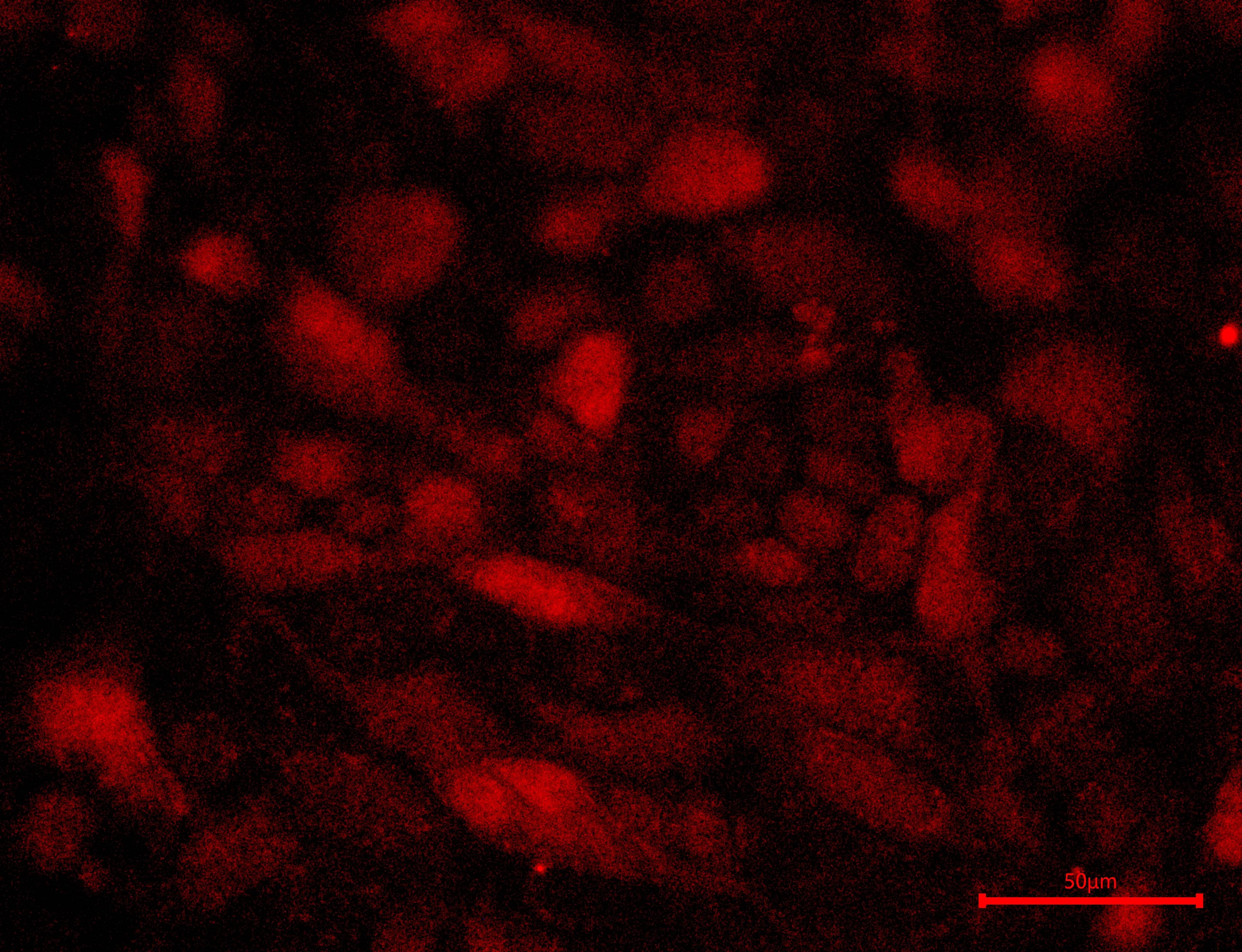

Supplement: Supplementary file 1 [file metabolites-16-00340-s001.zip › Figure S2 Uncropped microscopy images/Figure8/ASC/PA红4(1).jpg]

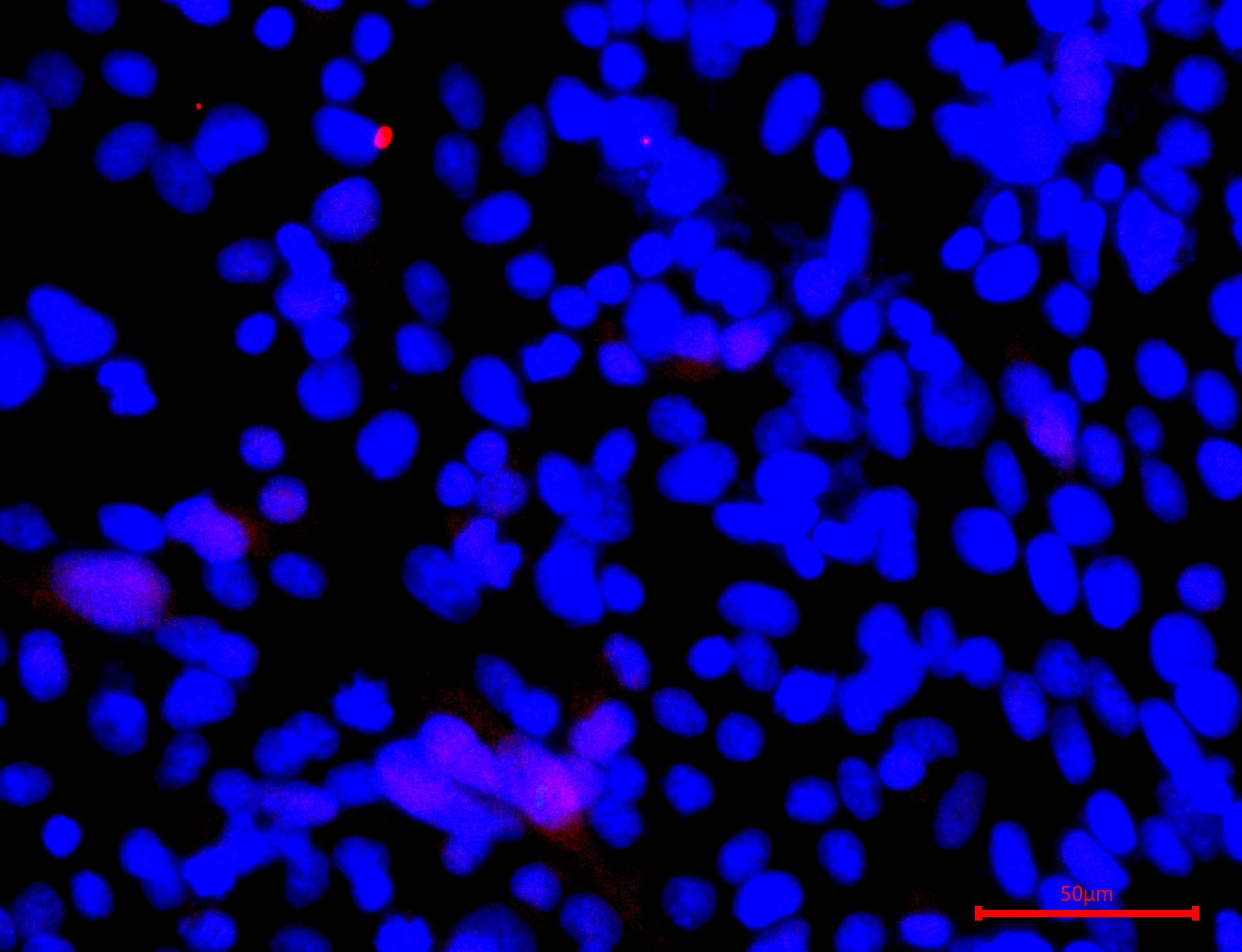

Supplement: Supplementary file 1 [file metabolites-16-00340-s001.zip › Figure S2 Uncropped microscopy images/Figure8/ASC/PQQmerge1(1).jpg]

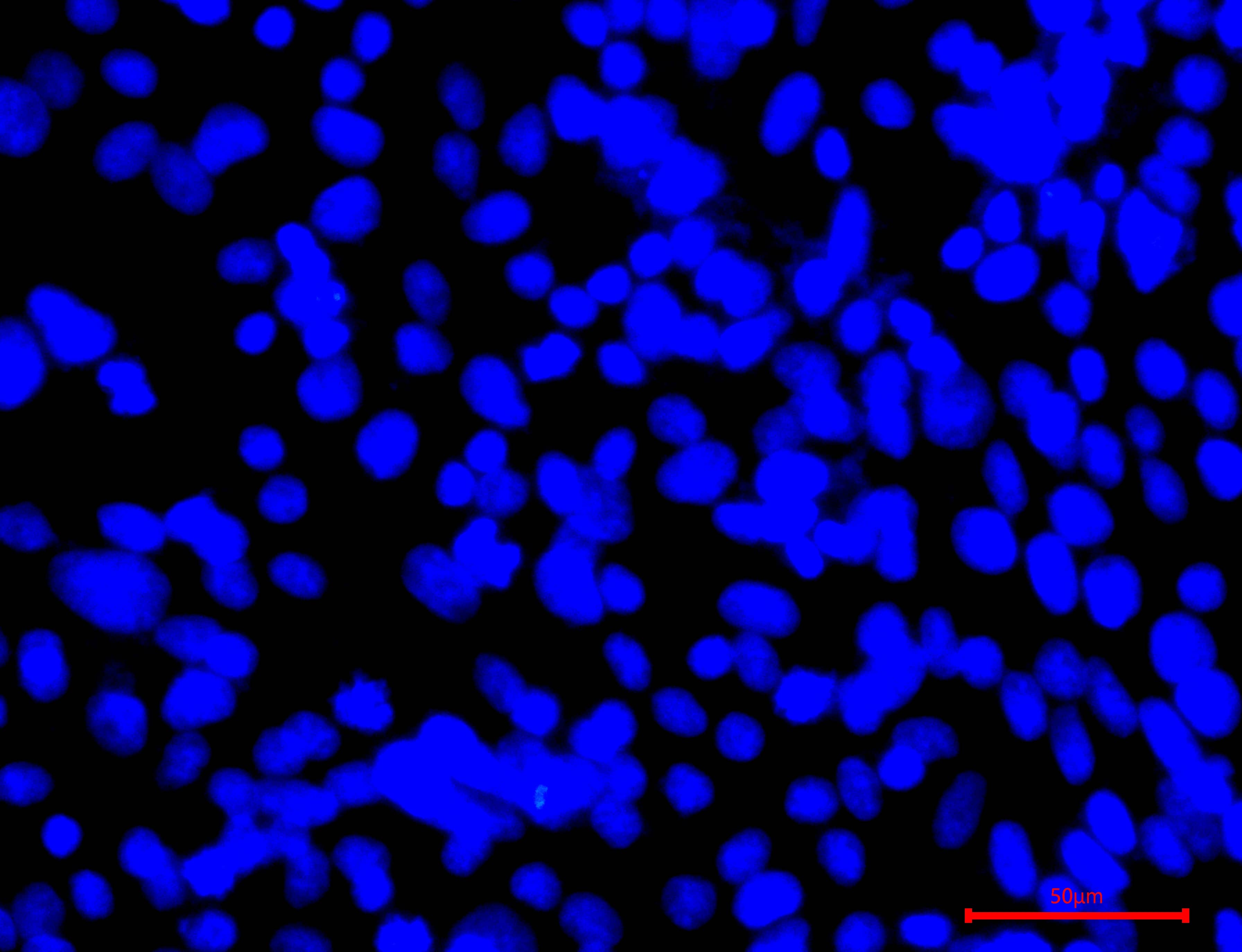

Supplement: Supplementary file 1 [file metabolites-16-00340-s001.zip › Figure S2 Uncropped microscopy images/Figure8/ASC/PQQ核1(1).jpg]

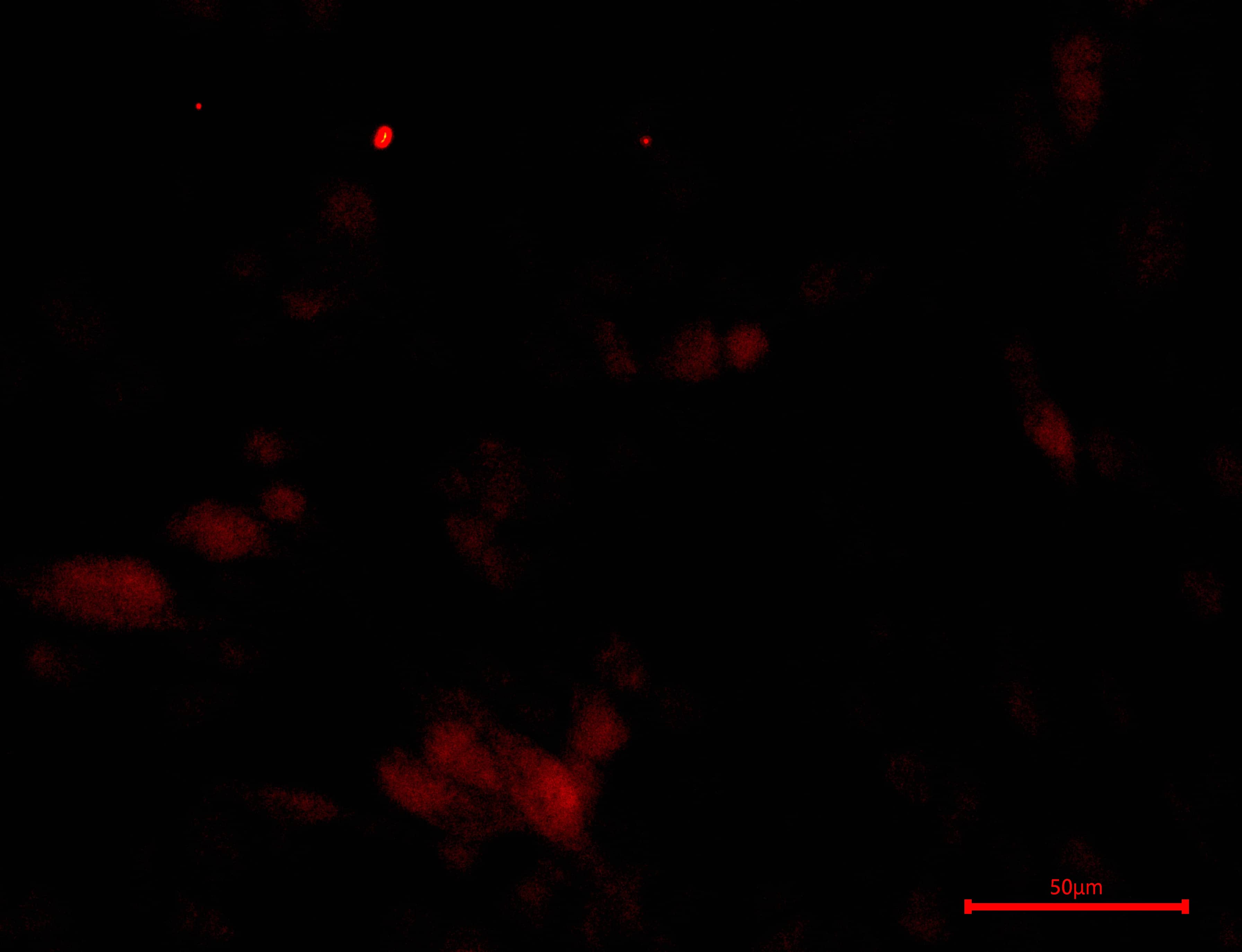

Supplement: Supplementary file 1 [file metabolites-16-00340-s001.zip › Figure S2 Uncropped microscopy images/Figure8/ASC/PQQ红1(1).jpg]

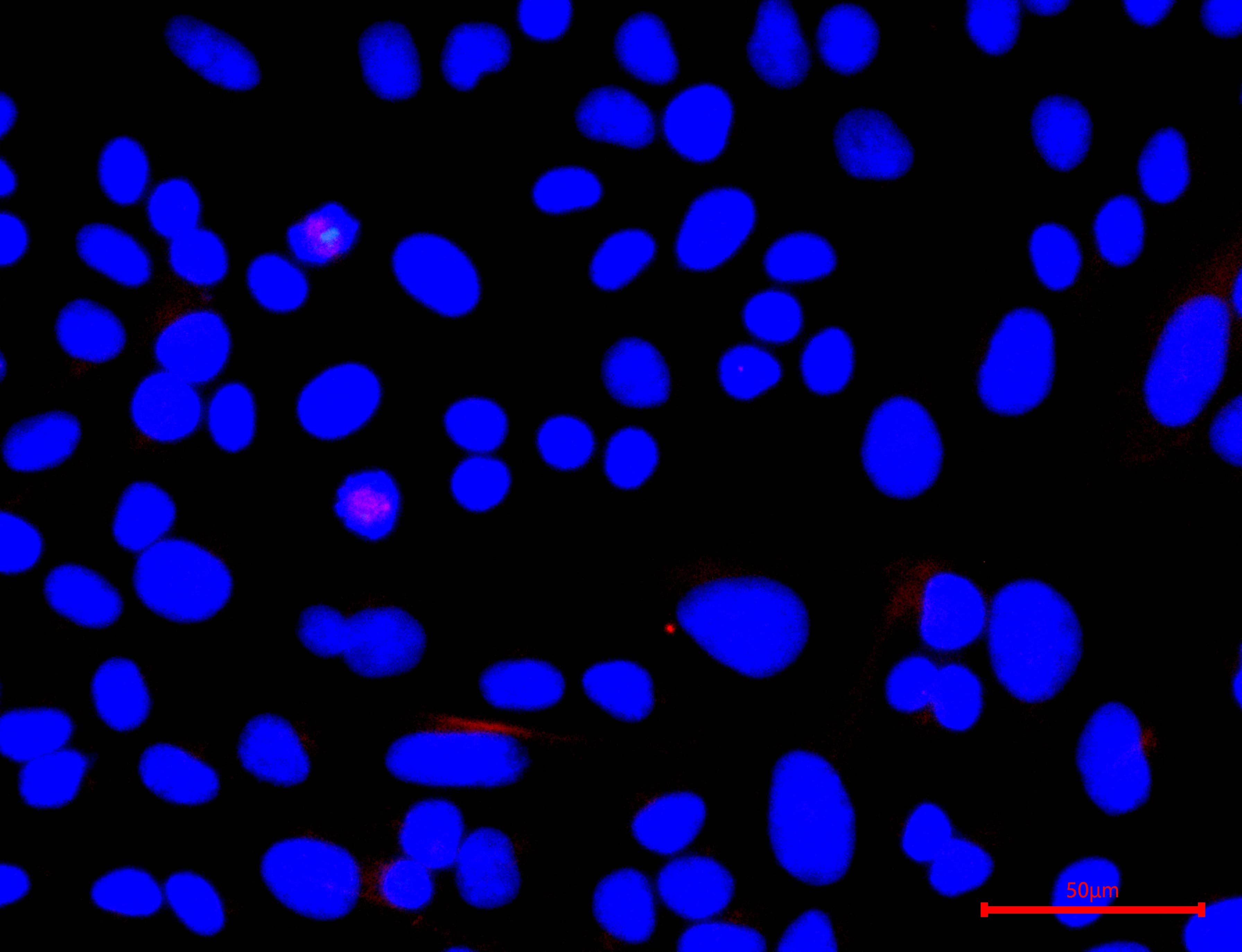

Supplement: Supplementary file 1 [file metabolites-16-00340-s001.zip › Figure S2 Uncropped microscopy images/Figure8/Caspase-1 cut/CTLmerge1(1).jpg]

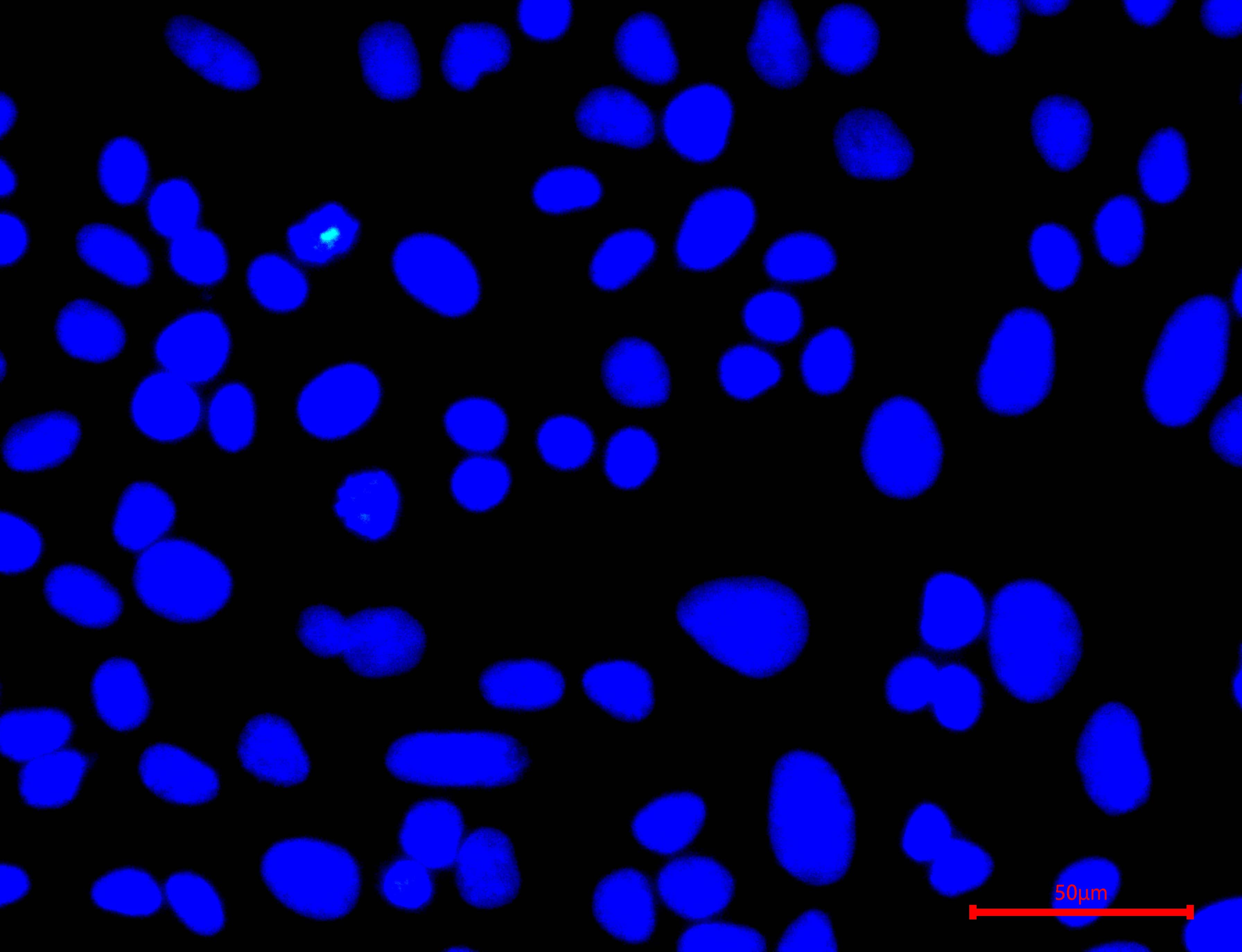

Supplement: Supplementary file 1 [file metabolites-16-00340-s001.zip › Figure S2 Uncropped microscopy images/Figure8/Caspase-1 cut/CTL核1(1).jpg]

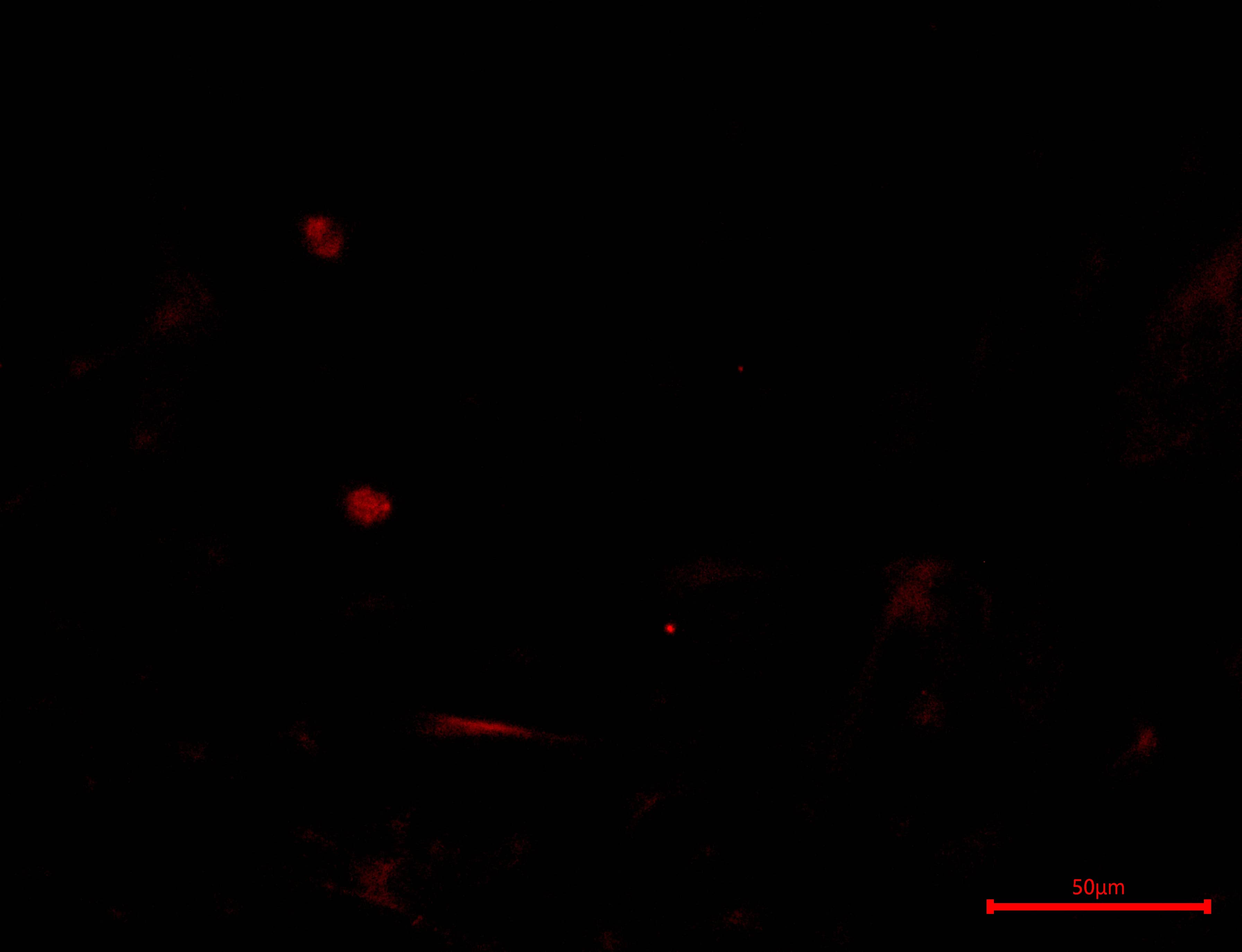

Supplement: Supplementary file 1 [file metabolites-16-00340-s001.zip › Figure S2 Uncropped microscopy images/Figure8/Caspase-1 cut/CTL红1(1).jpg]

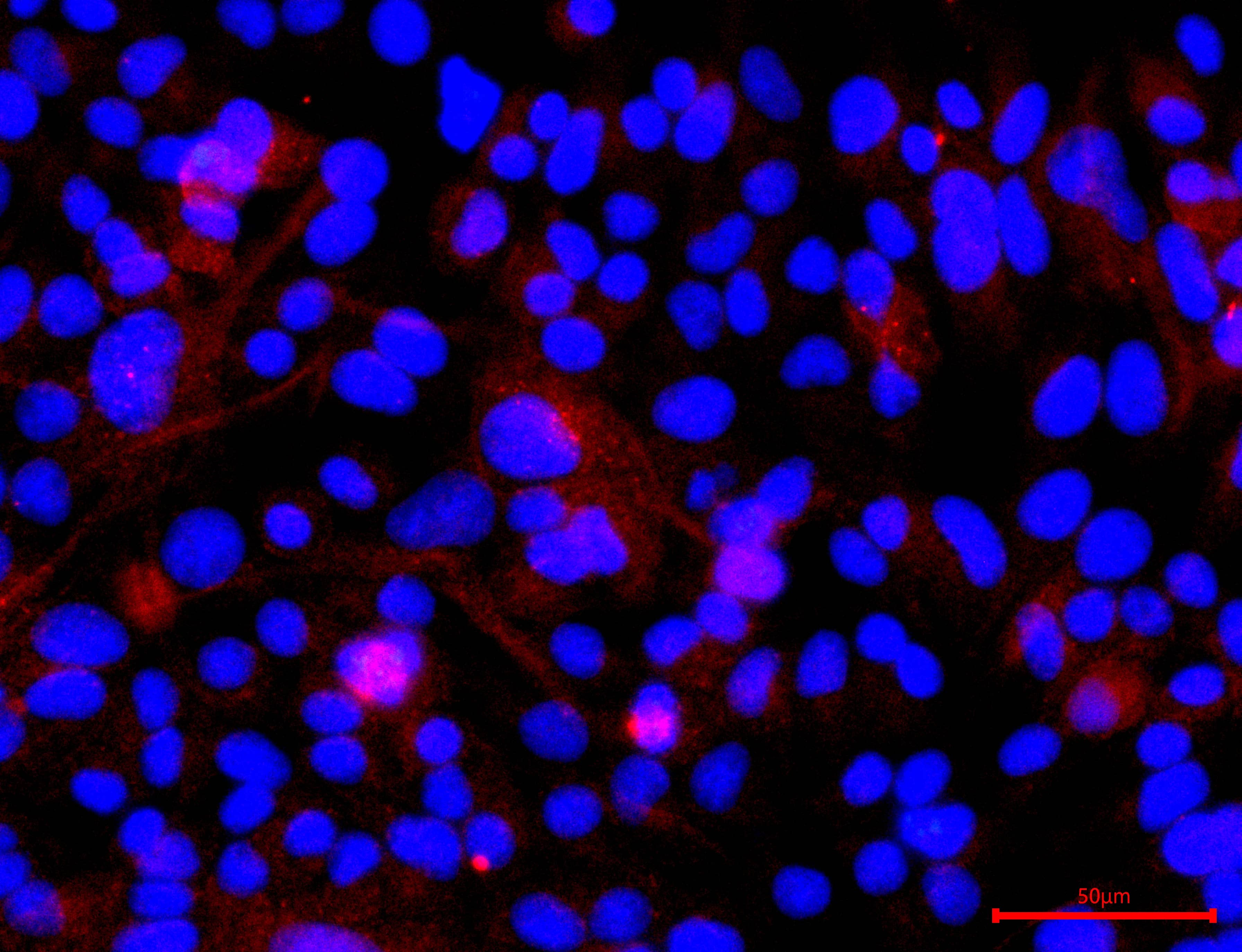

Supplement: Supplementary file 1 [file metabolites-16-00340-s001.zip › Figure S2 Uncropped microscopy images/Figure8/Caspase-1 cut/Nmerge2(1).jpg]

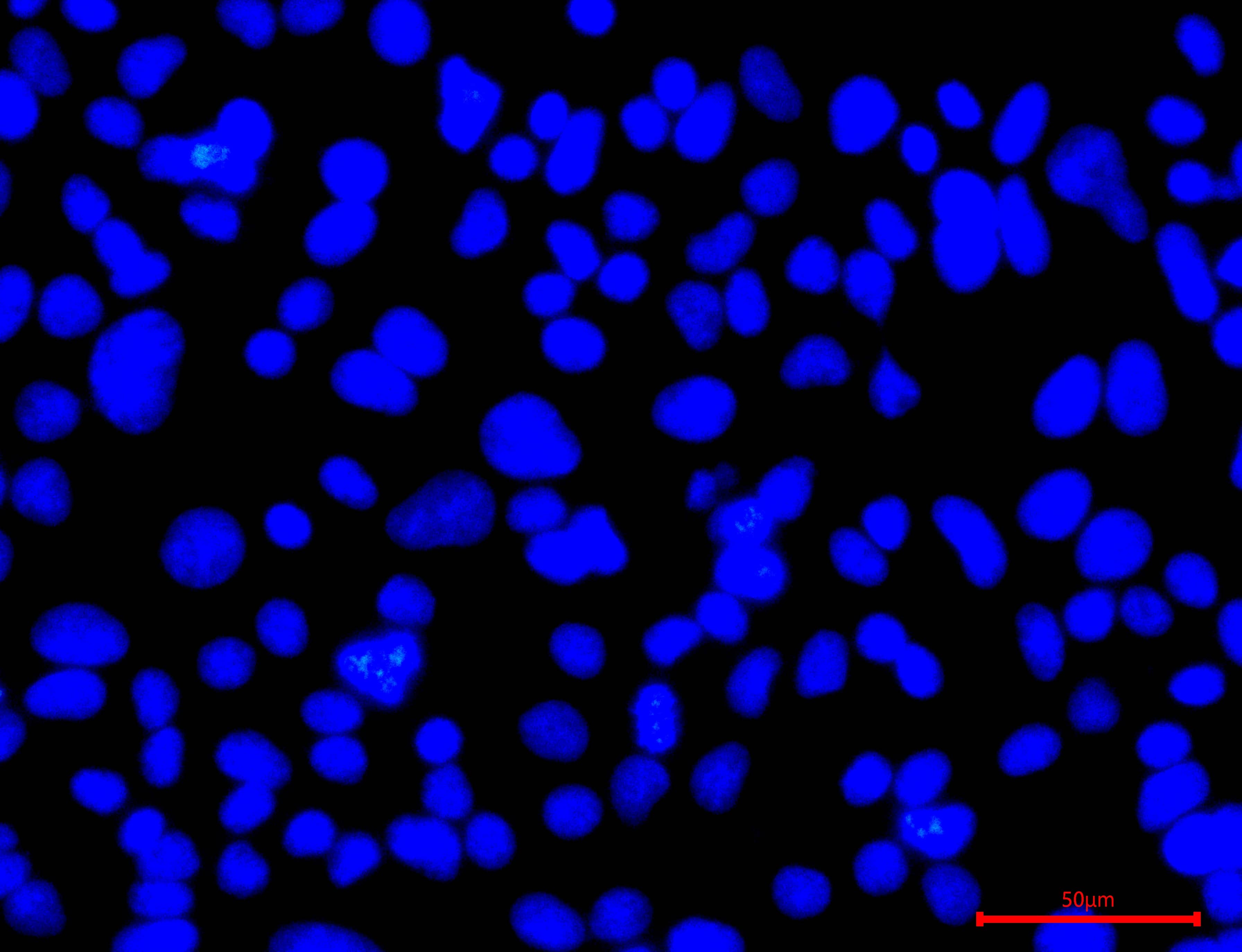

Supplement: Supplementary file 1 [file metabolites-16-00340-s001.zip › Figure S2 Uncropped microscopy images/Figure8/Caspase-1 cut/N核2(1).jpg]

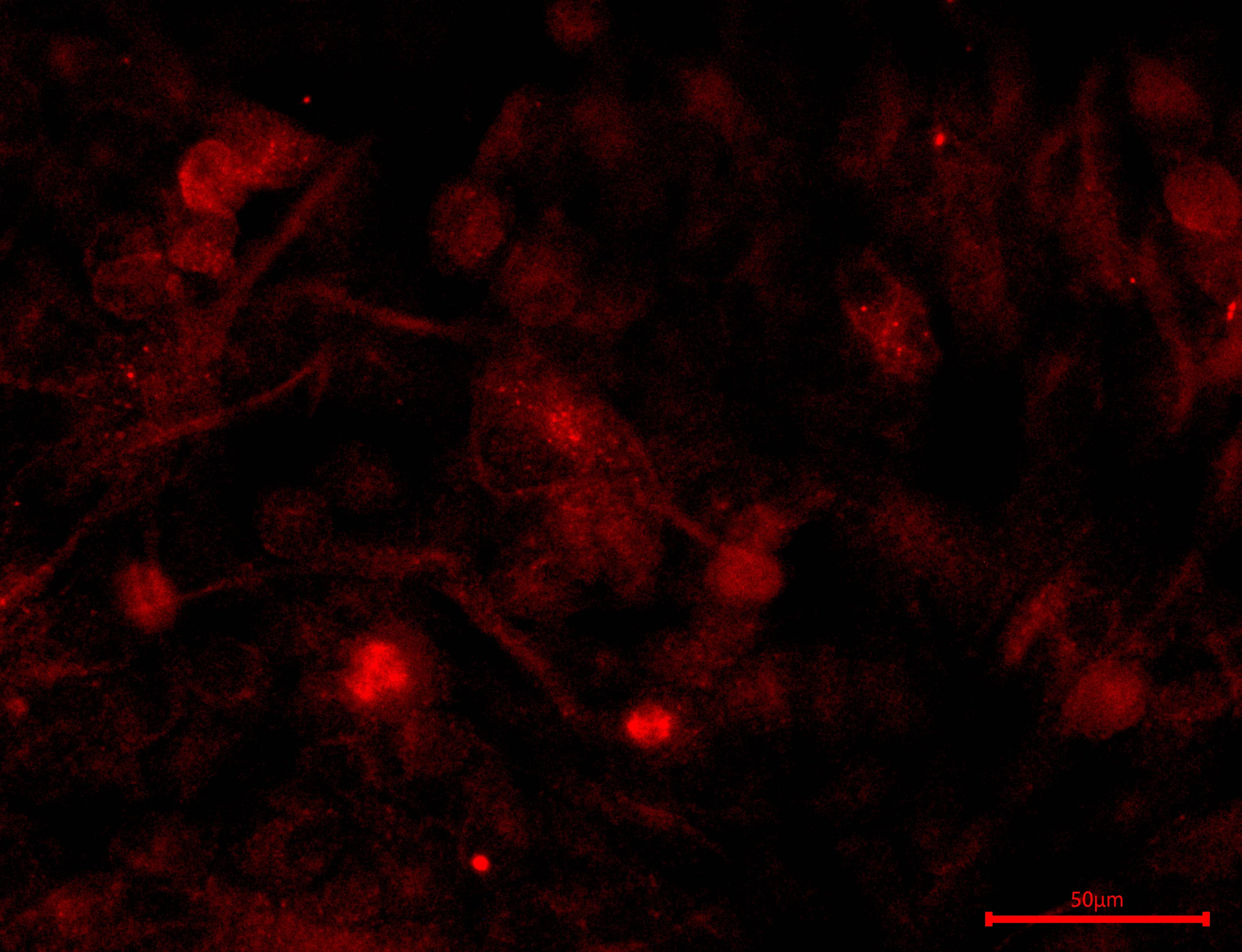

Supplement: Supplementary file 1 [file metabolites-16-00340-s001.zip › Figure S2 Uncropped microscopy images/Figure8/Caspase-1 cut/N红2(1).jpg]

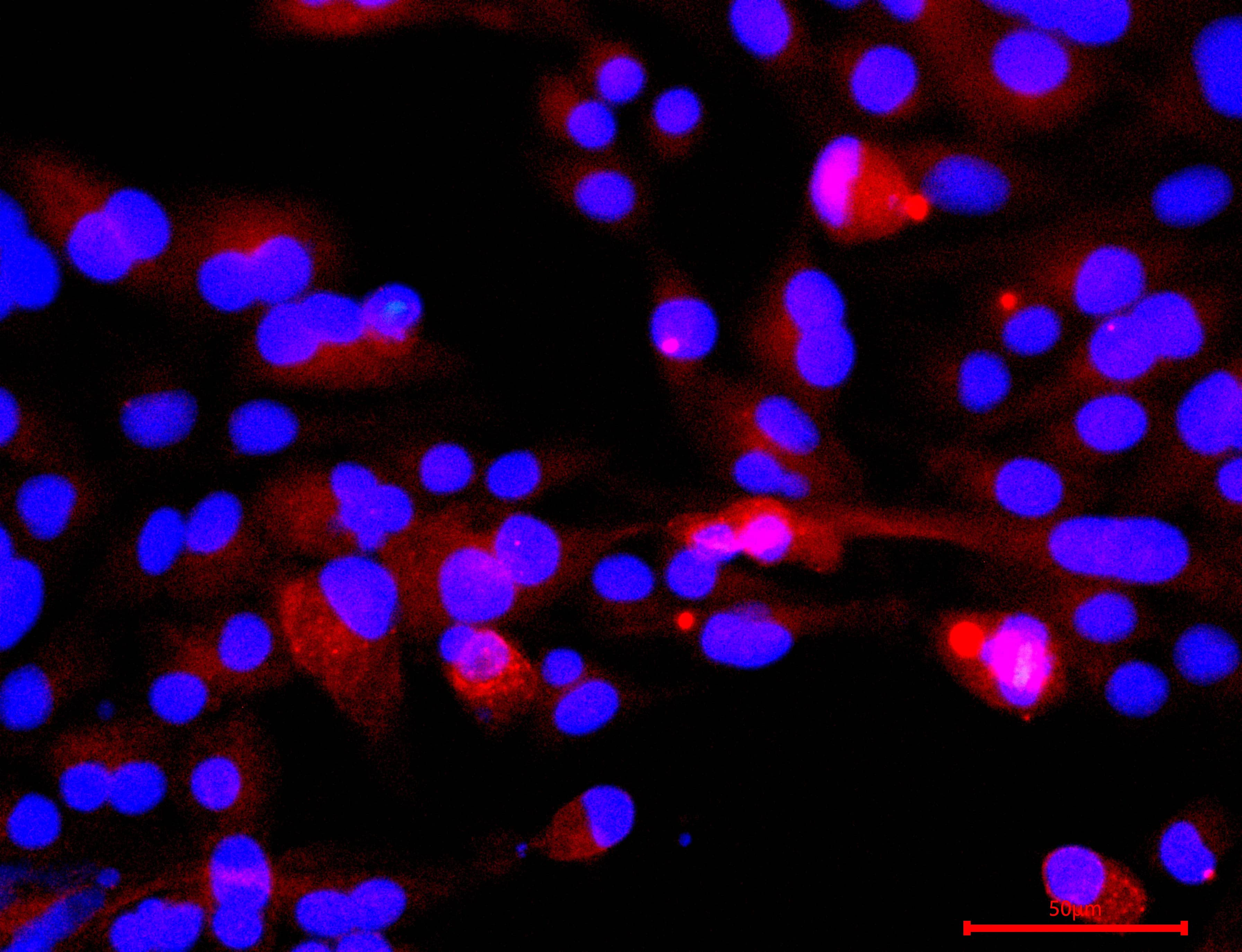

Supplement: Supplementary file 1 [file metabolites-16-00340-s001.zip › Figure S2 Uncropped microscopy images/Figure8/Caspase-1 cut/PAmerge1(1).jpg]

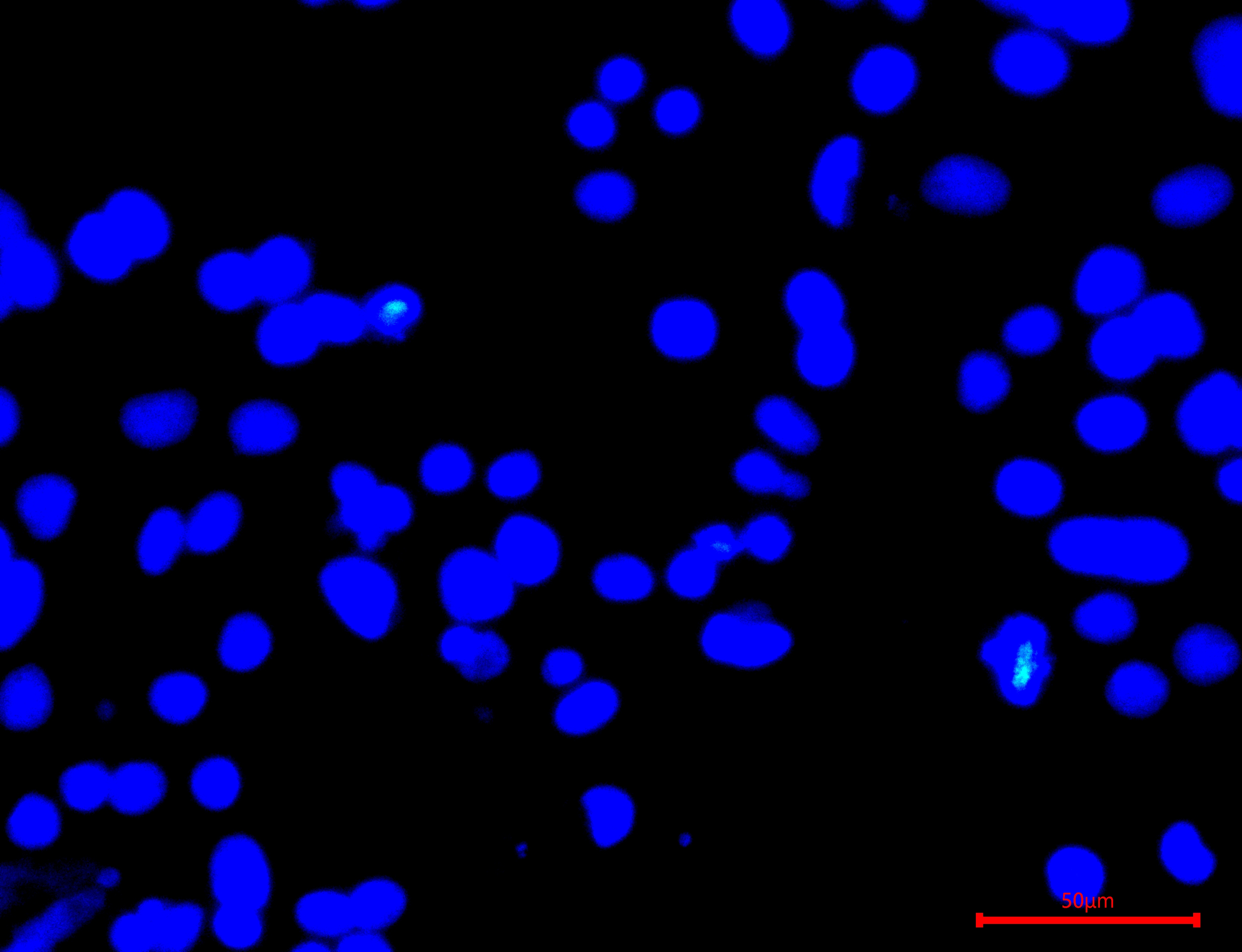

Supplement: Supplementary file 1 [file metabolites-16-00340-s001.zip › Figure S2 Uncropped microscopy images/Figure8/Caspase-1 cut/PA核1.jpg]

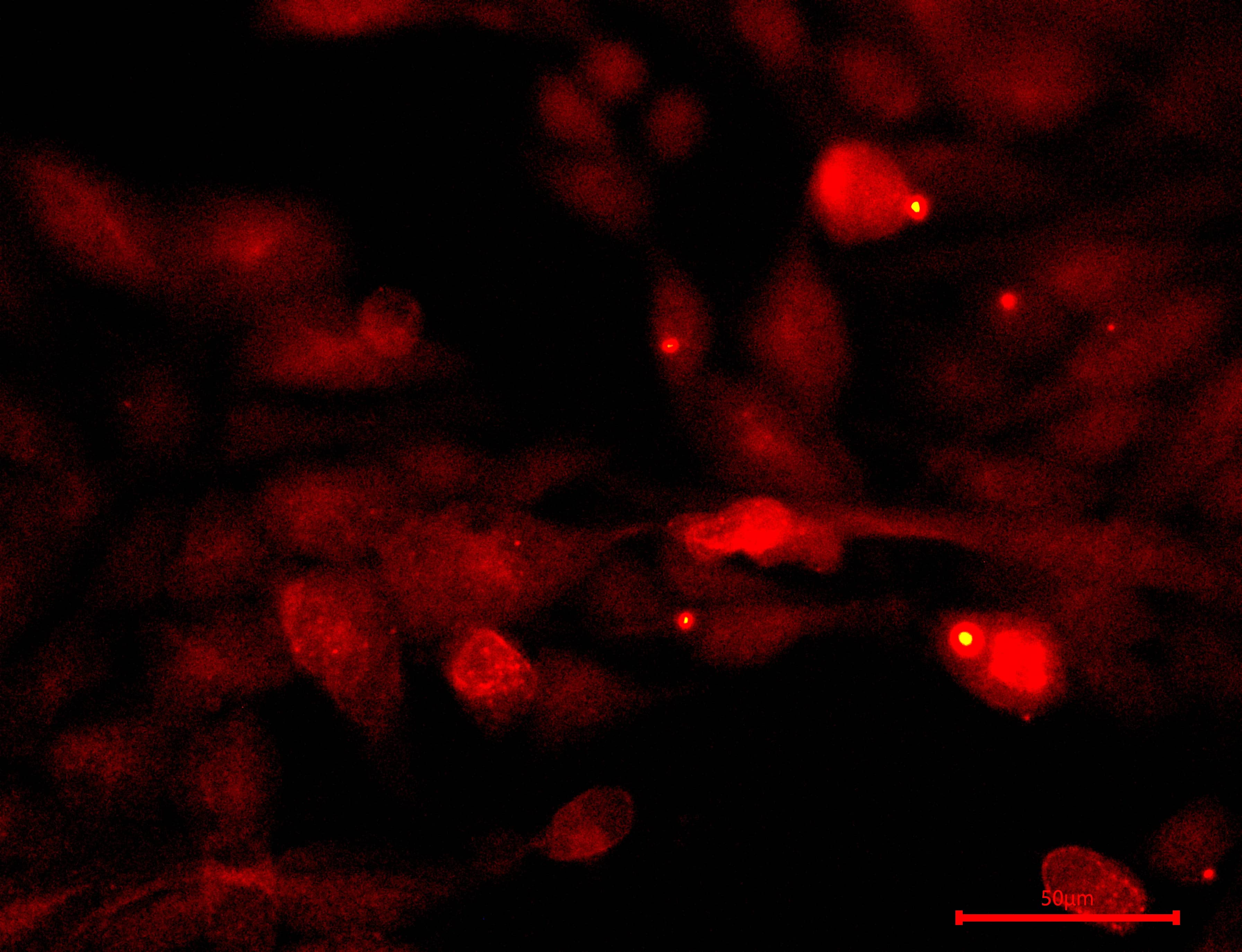

Supplement: Supplementary file 1 [file metabolites-16-00340-s001.zip › Figure S2 Uncropped microscopy images/Figure8/Caspase-1 cut/PA红1(1).jpg]

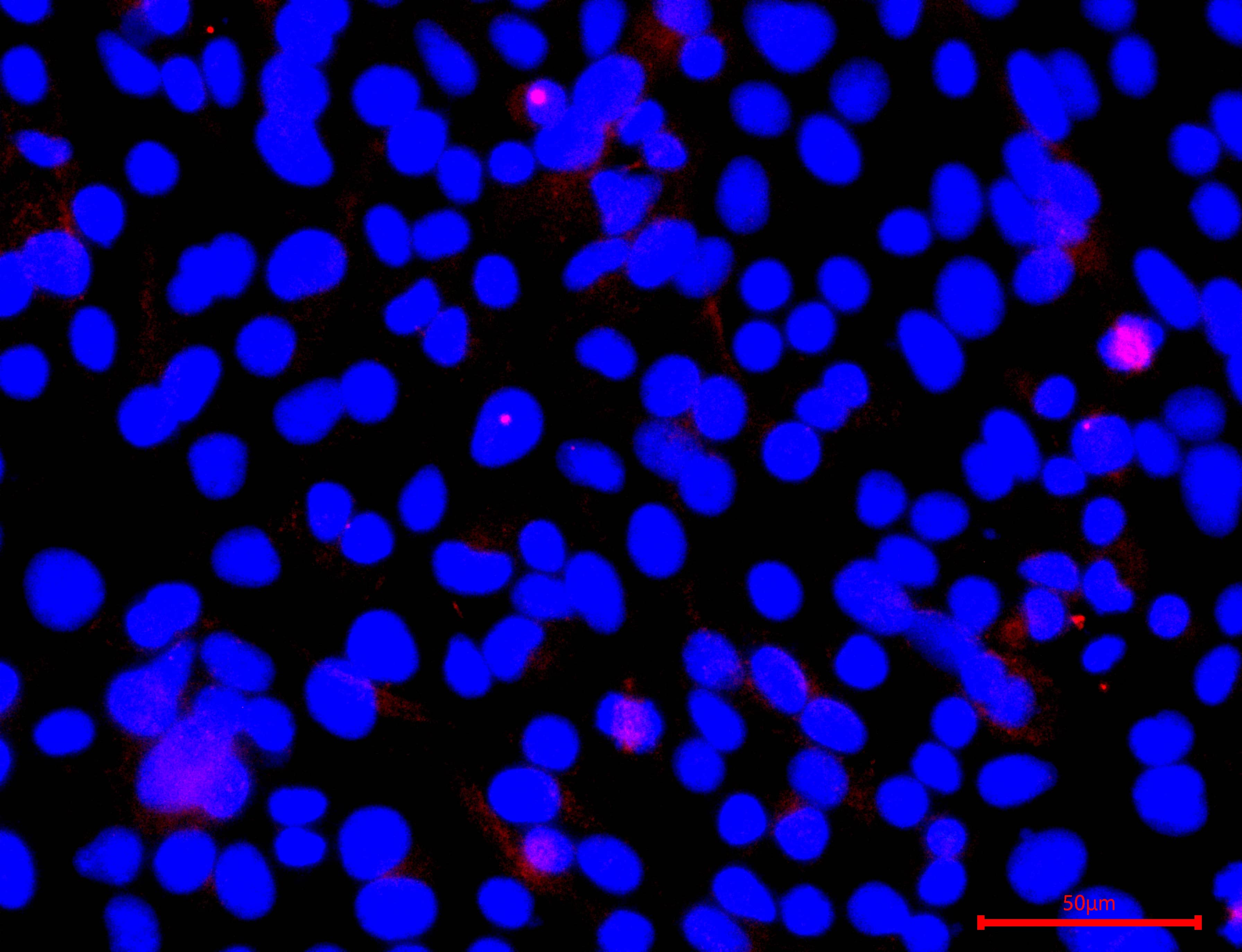

Supplement: Supplementary file 1 [file metabolites-16-00340-s001.zip › Figure S2 Uncropped microscopy images/Figure8/Caspase-1 cut/PQQmerge1(1).jpg]

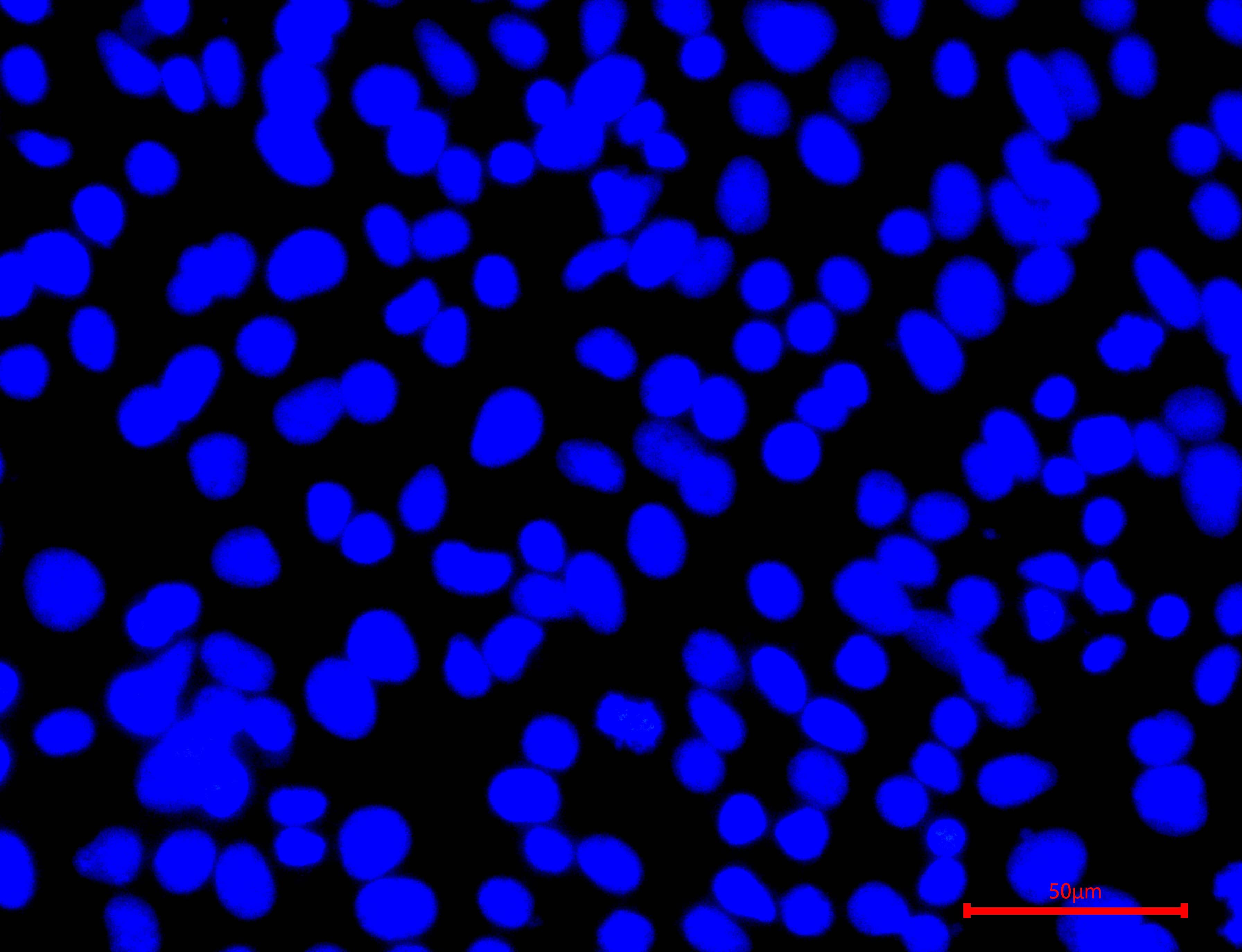

Supplement: Supplementary file 1 [file metabolites-16-00340-s001.zip › Figure S2 Uncropped microscopy images/Figure8/Caspase-1 cut/PQQ核1(1).jpg]

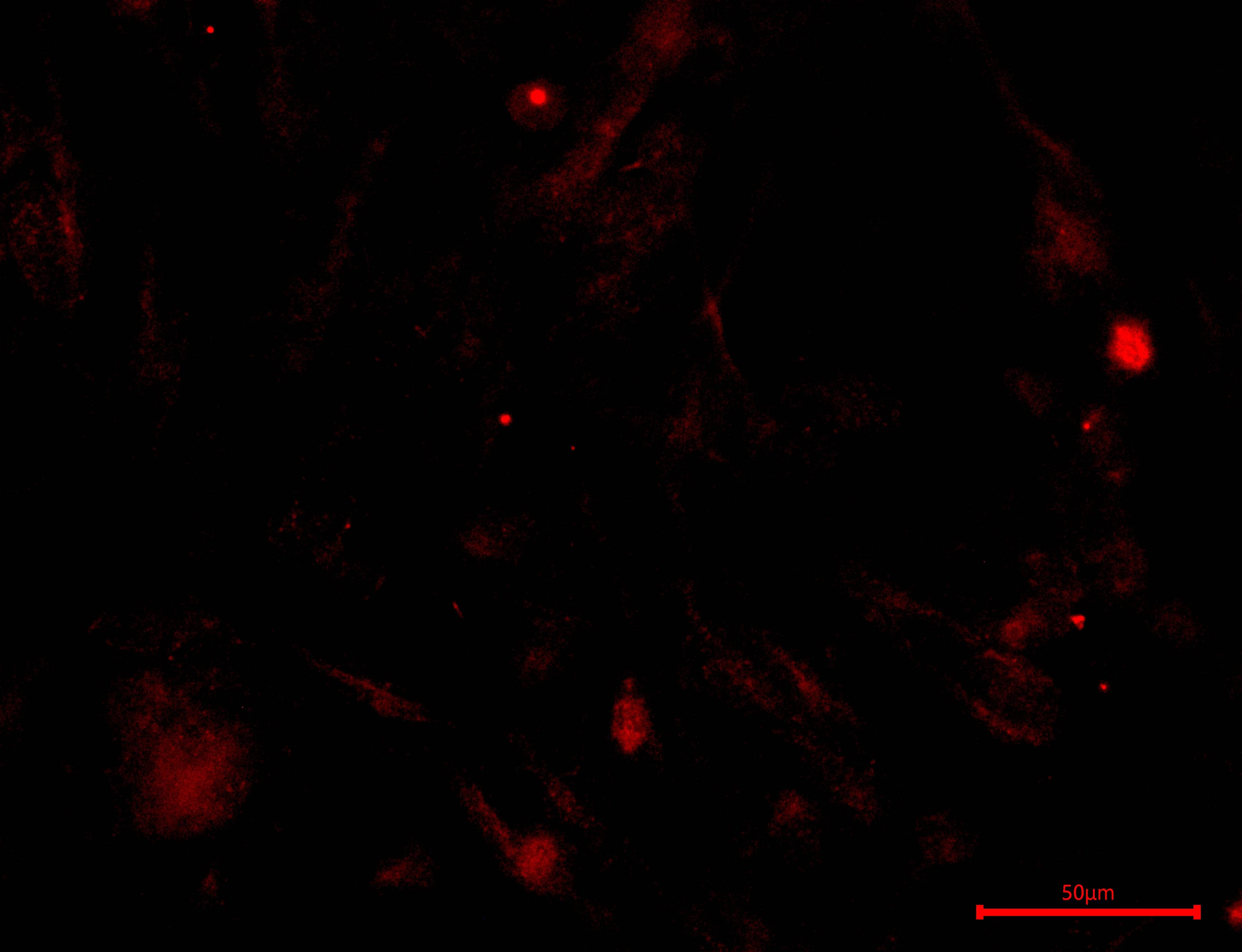

Supplement: Supplementary file 1 [file metabolites-16-00340-s001.zip › Figure S2 Uncropped microscopy images/Figure8/Caspase-1 cut/PQQ红1(1).jpg]

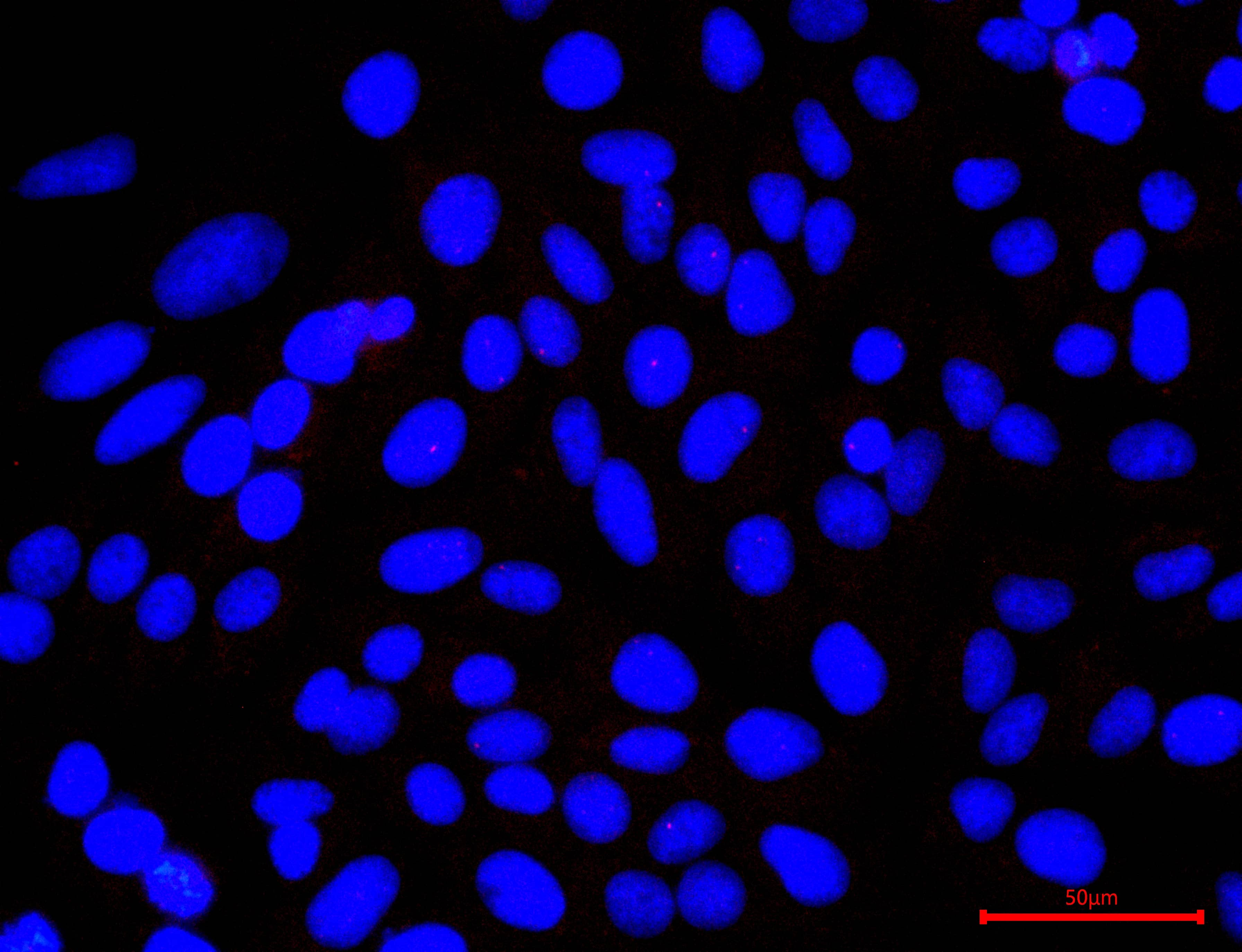

Supplement: Supplementary file 1 [file metabolites-16-00340-s001.zip › Figure S2 Uncropped microscopy images/Figure8/IL-1β/CTLmerge3(1).jpg]

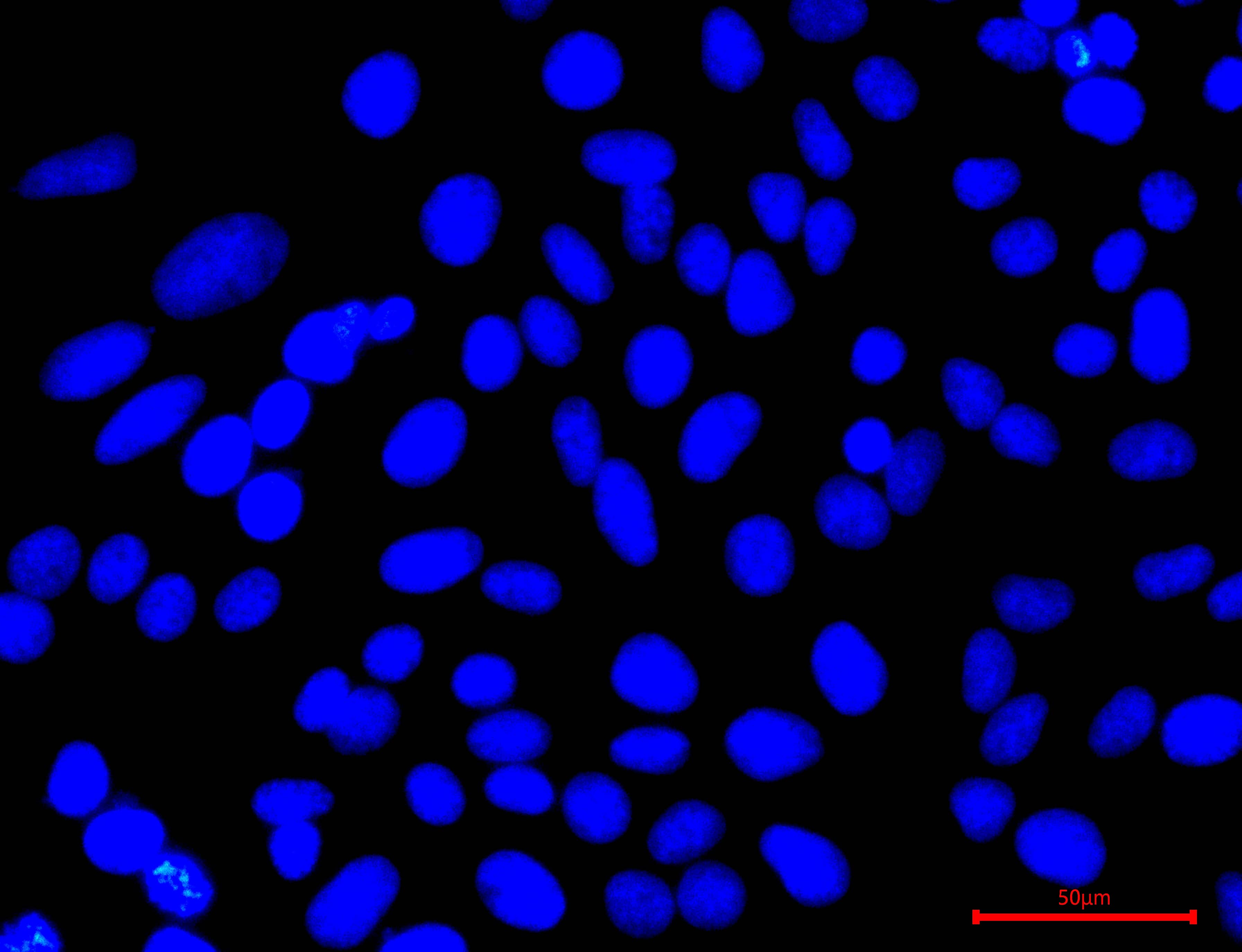

Supplement: Supplementary file 1 [file metabolites-16-00340-s001.zip › Figure S2 Uncropped microscopy images/Figure8/IL-1β/CTL核3(1).jpg]

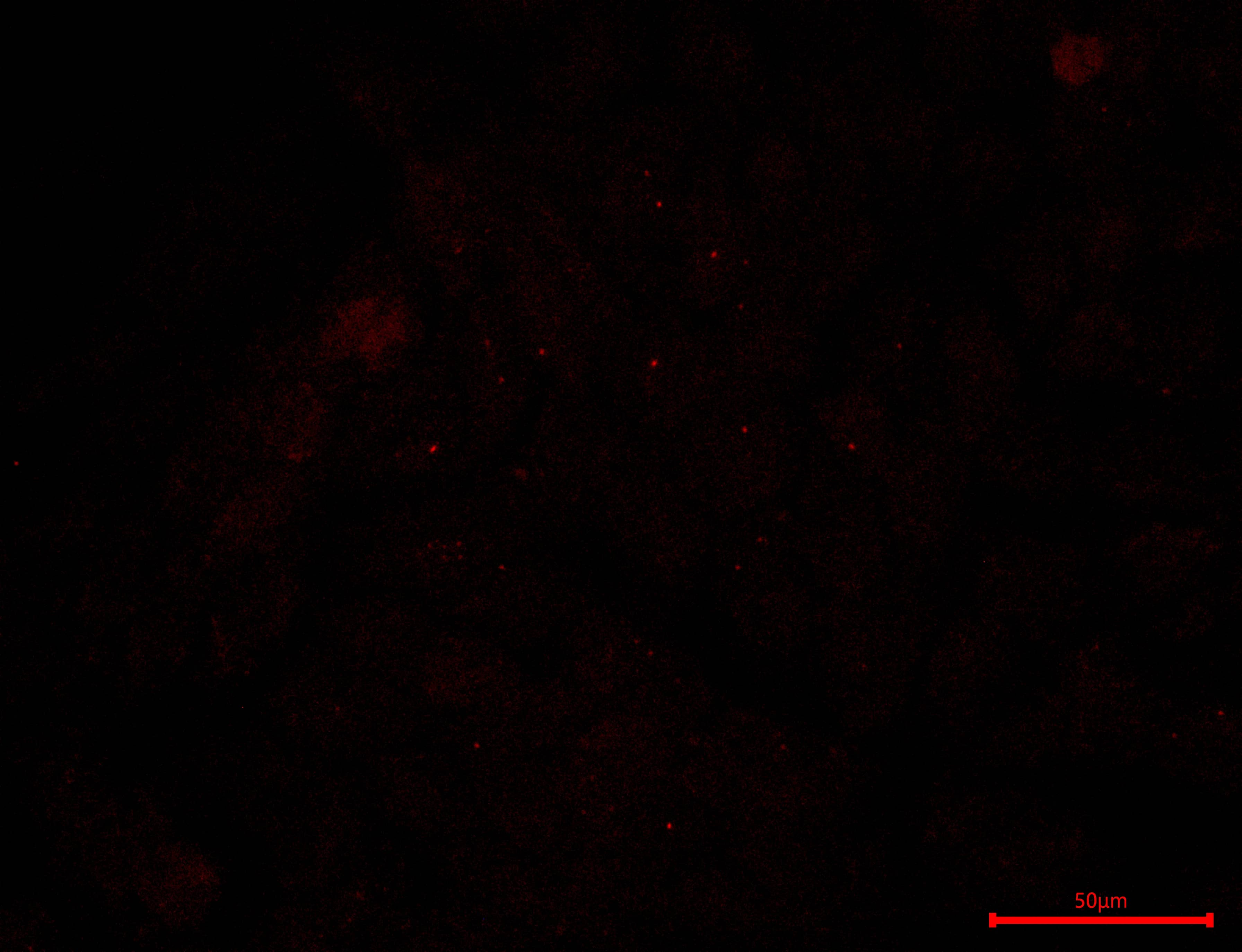

Supplement: Supplementary file 1 [file metabolites-16-00340-s001.zip › Figure S2 Uncropped microscopy images/Figure8/IL-1β/CTL红3(1).jpg]
